# Long-term effects of alcohol consumption on cognitive function: a systematic review and dose-response analysis of evidence published between 2007 and 2018

**DOI:** 10.1186/s13643-019-1220-4

**Published:** 2020-02-13

**Authors:** Sue E. Brennan, Steve McDonald, Matthew J. Page, Jane Reid, Stephanie Ward, Andrew B. Forbes, Joanne E. McKenzie

**Affiliations:** School of Public Health and Preventive Medicine, Monash University, Melbourne, Australia

**Keywords:** Alcohol, Systematic review, Cognition dose-response, Meta-analysis

## Abstract

**Background:**

Understanding the long-term health effects of low to moderate alcohol consumption is important for establishing thresholds for minimising the lifetime risk of harm. Recent research has elucidated the dose-response relationship between alcohol and cardiovascular outcomes, showing an increased risk of harm at levels of intake previously thought to be protective. The primary objective of this review was to examine (1) whether there is a dose-response relationship between levels of alcohol consumption and long-term cognitive effects, and (2) what the effects are of different levels of consumption.

**Methods:**

The review was conducted according to a pre-specified protocol. Eligible studies were those published 2007 onwards that compared cognitive function among people with different levels of alcohol consumption (measured ≥ 6 months prior to first follow-up of cognition). Major cognitive impairment was excluded. Searches were limited to MEDLINE, Embase and PsycINFO (January 2007 to April 2018). Screening, data extraction, and risk of bias assessment (ROBINS-I) were piloted by three authors, then completed by a single author and checked by a second. Analyses were undertaken to identify and characterise dose-response relationships between levels of alcohol consumption and cognition. Certainty of evidence was assessed using GRADE.

**Results:**

We included 27 cohort studies (from 4786 citations). Eighteen studies examined the effects of alcohol consumption at different levels (risk of bias 16 serious, 2 critical). Ten studies provided data for dose-response analysis. The pooled dose-response relationship showed a maximum standardised mean difference (SMD) indicating slightly better cognition among women with moderate alcohol consumption compared to current non-drinkers (SMD 0.18, 95%CI 0.02 to 0.34, at 14.4 grams/day; 5 studies, very low certainty evidence), and a trivial difference for men (SMD 0.05, 95% CI 0.00 to 0.10, at 19.4 grams/day; 6 studies, very low certainty evidence).

**Conclusions:**

Major limitations in the design and reporting of included studies made it impossible to discern if the effects of ‘lower’ levels of alcohol intake are due to bias. Further review of the evidence is unlikely to resolve this issue without meta-analysis of individual patient data from cohort studies that address biases in the selection of participants and classification of alcohol consumption.

## Background

Alcohol consumption is an established risk factor for a large number of health conditions, contributing to morbidity and premature death from cancers, cardiovascular disease, and liver disease [1, 2]. Governments have attempted to mitigate these health impacts by providing guidelines for lower risk consumption of alcohol. However, uncertainty around the effects of light to moderate alcohol consumption has made it challenging to establish thresholds for minimising the lifetime risk of harm [2]. While light to moderate alcohol consumption has been associated with a protective effect on some outcomes (e.g. all-cause mortality, cardiovascular disease, and dementia), there is mounting evidence that these findings are an artefact of study design [2–4]. Recent research has helped elucidate the dose-response relationship between alcohol consumption and some of these outcomes showing that, rather than having protective effect, light to moderate alcohol intake is associated with an increased risk of stroke, other cardiovascular disease subtypes (excluding myocardial infarction), and all-cause mortality [1, 2]. Comparable studies examining the dose-response relationship between alcohol consumption and long-term cognitive outcomes are lacking [5, 6].

Rehm and colleagues recently reported an overview of twenty systematic reviews (published 2000-March 2018) that had examined the relationship between alcohol use and dementia or cognitive impairment [6]. Only one of the twenty reviews reported a dose-response analysis. The analysis showed an elevated risk of dementia when 38 g of alcohol or more is consumed per day, and a lower risk of dementia with ‘modest’ alcohol consumption (between 6 and 12.5 g per day) compared to other levels of intake [7]. Studies measuring other cognitive outcomes were excluded from Xu et al. Although the findings from Xu et al. are consistent with earlier systematic reviews (e.g. [8–10]), the recent evidence against any protective effect of alcohol on cardiovascular outcomes signals the need to closely examine the association between light to moderate alcohol intake and cognition. In particular, a dose-response analysis considering other cognitive outcomes is needed, together with a detailed assessment of the extent to which observed results may be explained by bias.

The current systematic review aims to address evidence gaps, examining the dose-response relationship between alcohol and mild cognitive impairment. We focus on the cumulative effects of lower levels of alcohol exposure on cognitive function—those effects arising from drinking over time (not a single occasion). Although these effects may be most evident after a longer period of exposure (typically, later in life), there is also a need to examine the potential for long-term effects on cognition arising from drinking alcohol early in life (up to the age of 25). This is because of the concerns that exposure to alcohol during this period of brain development may bring an increased risk of cognitive impairment [11, 12]. The review was commissioned to inform an update of the 2009 Australian Guidelines to Reduce Health Risks from Drinking Alcohol (the Alcohol Guidelines) [13]. As such, it considers evidence published from 2007 onwards (i.e. subsequent to the evidence review conducted for the 2009 Alcohol Guideline).

## Objectives

The objectives of the review are to address the following questions.

1. Is there a dose-response relationship between levels of alcohol consumption and long-term cognitive effects for women and men? If so, what are the effects at different levels of consumption?

The different levels of alcohol consumption defined for the review were based on increments of a single standard Australian drink (10 g of alcohol). This standard is common to a number of other countries (e.g. France, Netherlands, New Zealand, Spain), with some countries having slightly lower (e.g. United Kingdom) or higher (e.g. Canada, United States) standards. The levels were the following:

- Never drinking or very low-level drinking (0 to < 10 g/week)
- ≥ 10 g/week and < 10 g/day
- ≥ 10 g/day and < 20 g/day
- ≥ 20 g/day and < 30 g/day
- ≥ 30 g/day and < 40 g/day
- ≥ 40 g/day and < 50 g/day
- ≥ 50 g/day

Secondary objectives

2. Is the effect of alcohol consumption on long-term cognition modified by age, co-morbidities, or drug use?
3. What studies are available comparing the long-term effects of different patterns of alcohol consumption on cognition for women and men? What questions are addressed by these studies (in terms of populations, alcohol consumption patterns, and outcomes)?

Different patterns of consumption were defined inclusively for the review. Examples include different levels of per-occasion consumption of alcohol (e.g. infrequent “heavy” or “binge” drinking versus regular drinking within lower risk levels), different frequency of drinking, and different patterns of consumption over time. Since the literature on the effects of different patterns of alcohol consumption covers diverse questions, examining non-comparable patterns of intake, among different populations, these studies were summarised to map available evidence.

## Methods

Methods for the review were pre-specified in a protocol, which was peer-reviewed prior to conducting the review (Additional file 1; Changes to protocol, Additional file 2, Appendix 1). The review was not registered on PROSPERO due to plans for public consultation prior to wider dissemination. The methods reported in this review are based on the Cochrane Handbook for Systematic Reviews of Interventions [14], with modifications for undertaking a review of exposures. The GRADE approach is used to summarise and assess the certainty of evidence arising from the review (see ‘Summary of findings tables and assessment of certainty of the body of evidence’ section for details). GRADE methods are widely used in guideline development to ensure a systematic, transparent and common approach to interpreting results [15]. The review is reported in accordance with the PRISMA statement [16, 17], with additional methods description based on the PRISMA-P statement [18, 19].

### Criteria for considering studies for this review

#### Types of participants

General population

Studies that were limited to one or more of the following subgroups were eligible for inclusion:

- People in specific age groups identified in the 2009 Alcohol guideline as potentially having a higher risk of harm from alcohol exposure than the general population. For example, children and young people (less than 18 years), young adults (18–25), older people (65 and over)
- Women or men

We planned to report data and analyses from studies that met other eligibility criteria for the following subgroups.

- People with existing health conditions (physical, mental or both)
- People using licit and/or illicit drugs
- People with a family history of alcohol dependence.

Studies restricted to one or more of these three subgroups were eligible only if the study explicitly aimed to examine the association between alcohol consumption and long-term cognition.

#### Types of exposure

Eligible studies were those examining different levels of alcohol consumption, patterns of alcohol consumption, or both.

##### Measurement methods and quantification

Studies were eligible irrespective of the methods used to measure alcohol exposure. We anticipated that these methods would vary across studies, but would include retrospective survey involving recall of alcohol consumption over different periods of life or intake diaries to measure current alcohol consumption. Single or repeated measures of exposure were eligible. Studies had to report alcohol consumption in units that allowed quantification of the average amount of alcohol consumed (e.g. grams or millilitres of pure alcohol) over a period of time (e.g. per day, week, month).

##### Timing of alcohol exposure measurement

The timing of measurement needed to match the study design features listed in ‘Types of studies’ section for a prospective design. Data collected on alcohol consumption, and used in analyses, had to be collected at least 6 months prior to the first follow-up measure of cognition. Concurrent measures of alcohol were accepted only in studies with multiple measures of alcohol over time, where the final measure was taken concurrently with a baseline (not follow-up) measure of cognition.

To account for differences in the methods used to measure alcohol exposure, we extracted data on the measurement methods and assessed potential biases that may arise through the method used.

#### Types of comparator exposure

For inclusion in the review, the comparator group must have been a different level or pattern of alcohol consumption.

For inclusion in the meta-analysis of different levels of alcohol consumption and the dose-response analysis, studies had to report results for either a ‘never’ drinker group or a ‘very low-level’ drinker group. We broadly defined ‘never’ drinkers as individuals that had never consumed a serve of alcohol (lifetime abstainers) or had consumed very little alcohol across their lifetime. Where lifetime consumption was not measured, we accepted current non-drinkers (e.g. based on consumption over the preceding 12 months), noting in data extraction and risk of bias assessment the potential for misclassification and contamination of a non-drinking group with former drinkers. A similar approach was taken to misclassification of occasional drinkers, where the recall period was such that occasional drinkers might be missed and incorrectly categorised as non-drinkers. We defined very low-level drinkers as those whose average alcohol consumption was 0 to < 10 g/week. The latter threshold reflects consumption of a single Australian standard drink (10 g of alcohol), and is common to a number of other countries (e.g. France, Netherlands, New Zealand, Spain).

We anticipated diversity across studies in the definition and composition of potentially eligible comparator groups (which may or may not be the referent group to which other categories of alcohol consumption were compared in each study) [20]. For example, across studies referent groups have been defined as never drinking [21], not drinking above a certain threshold (e.g. less than 1 unit of alcohol per week [22]), and not drinking over a defined period of time (e.g. less than 1 unit over the preceding 12 months [23]). Studies reporting a group with these or similarly low levels of alcohol consumption were eligible, irrespective of whether the group was used as the referent in the study.

#### Types of outcomes

Eligible studies were those that reported at least one measure of cognitive function (or performance), which is the primary outcome for this review. Studies must have assessed cumulative long-term effects of alcohol consumption on cognitive function (e.g. decline in function over time). We excluded studies that only examined acute effects (during intoxication or withdrawal), long-term effects arising from injury on a single drinking occasion (e.g. a traumatic brain injury sustained while intoxicated), and those where there was insufficient length of follow-up to examine the longer-term effects of cumulative exposure (< 6 months). While we did not set a minimum threshold for ‘long-term’, we considered the extent to which studies provided evidence of a sustained effect, and the duration of this effect, when interpreting results (see ‘Timing of outcome measurement’ section). We also excluded studies that only examined cognitive function as a predictor of alcohol-use behaviours (e.g. studies examining whether prior cognitive function led to heavy alcohol use).

Eligible outcomes were broadly categorised as follows.

Cognitive function

- Global cognitive function
- Domain-specific cognitive function (especially domains that reflect specific alcohol-related neuropathologies, such as psychomotor speed and working memory)

Clinical diagnoses of cognitive impairment

- Mild cognitive impairment (also referred to as mild neurocognitive disorders)

These conditions were ‘characterised by a decline from a previously attained cognitive level’ ([5], p2675).

Major cognitive impairment (also referred to as major neurocognitive disorders; including dementia) was excluded.

We expected that definitions and diagnostic criteria would vary across studies, so we accepted a range of definitions as noted under ‘Methods of outcome assessment’ section. Table 1 provides an example of specific domains of cognitive function used in the diagnosis of mild and major cognitive impairment in the Diagnostic and Statistical Manual of Mental Disorders, Fifth Edition (DSM-5) [24]).

**Table 1:** Domains used to diagnose major and mild neurocognitive disorders in the DSM-5

Domain	Cognitive abilities covered by the domain
Complex attention	Sustained attention, divided attention, selective attention, processing
Executive function	Planning, decision making, working memory, responding to feedback/error correction, overriding habits, mental flexibility
Learning and memory	Immediate memory, recent memory
Language	Expressive language and receptive language
Perceptual-motor ability	Construction and visual perception
Social cognition	Recognition of emotions, theory of mind, behavioural regulation

##### Methods of outcome assessment

Any measure of cognitive function was eligible for inclusion. The tests or diagnostic criteria used in each study should have had evidence of validity and reliability for the assessment of mild cognitive impairment, but studies were not excluded on this basis.

We anticipated that many different methods would be used to assess cognitive functioning across studies. These include the following.

Clinical diagnoses of

- Mild cognitive impairment using explicit criteria (e.g. [25], National Institute on Aging and the Alzheimer’s Association (United States; NIA-AA) criteria [26]; any of the definitions of mild cognitive impairment described in [27])

Neuropsychological tests used to assess global cognitive function, for example the:

- Mini-Mental State Examination (MMSE)
- Addenbrooke’s Cognitive Examination-Revised (ACE-R) which “incorporates the MMSE and assesses attention, orientation, fluency, language, visuospatial function, and memory, yielding subscale scores for each domain” [28]
- Montreal Cognitive Assessment (MOCA), which provides measures for specific cognitive abilities and may be more suitable for assessing mild cognitive impairment than the MMSE [28]

Neuropsychological tests for assessing domain-specific cognitive function, for example, tests of:

- Attention and processing speed, for example, the Trail making test (TMT-A)
- Memory, for example, the Hopkins verbal learning test (HVLT-R; immediate, delay)
- Visuospatial ability, for example the Block design test
- Executive function, for example, the Controlled Oral Word Association Test (COWAT)

Results could be reported as an overall test score that provides a composite measure across multiple areas of cognitive ability (i.e. global cognitive function), sub-scales that provide a measure of domain-specific cognitive function or cognitive abilities (e.g. processing speed, memory), or both.

##### Timing of outcome measurement

Studies with a minimum follow-up of 6 months were eligible, a time frame chosen to ensure that studies were designed to examine more persistent effects of alcohol consumption. This threshold was based on previous reviews examining the association between long-term cognitive impairment and alcohol consumption (e.g. Anstey 2009 specified 12 months [29]) and guidance from the Cochrane Dementia and Cochrane Improvement Group, which suggests a minimum follow-up of 9 months for studies examining progression from mild cognitive impairment to dementia [28]. We deliberately specified a shorter period to ensure studies reporting important long-term effects were not missed.

No restrictions were placed on the number of points at which the outcome was measured, but the length of follow-up and number of measurement points (including a baseline measure of cognition) was considered when interpreting study findings and in deciding which outcomes were similar enough to combine for synthesis. Since long-term cognitive impairment is characterised as a decline from a previous level of cognitive function and implies a persistent effect, studies with longer-term outcome follow up at multiple time points should provide the most direct evidence.

##### Selection of cognitive outcomes where multiple are reported

We anticipated that individual studies would report data for multiple cognitive outcomes.

Specifically, a single study may report results:

- For multiple constructs related to cognitive function, for example, global cognitive function and cognitive ability on specific domains (e.g. memory, attention, problem-solving, language);
- Using multiple methods or tools to measure the same or similar outcome, for example reporting measures of global cognitive function using both the MMSE and the MOCA;
- At multiple time points, for example, at 1, 5, and 10 years.

Where multiple cognition outcomes were reported, we selected one outcome for inclusion in analyses and for reporting the main outcomes (e.g. for GRADEing), choosing the result that provided the most complete information for analysis. Where multiple results remained, we listed all available outcomes (without results) and asked our content expert to independently rank these based on relevance to the review question, and the validity and reliability of the measures used. Measures of global cognitive function were prioritised, followed by measures of memory, then executive function. Methods for selecting results when there were multiple effect estimates and/or analyses are described in ‘Measures of association’ and ‘Summary of findings tables and assessment of certainty of the body of evidence’ sections.

##### Secondary outcomes

We planned to include studies that reported brain structure outcomes (as measured by neuroimaging) only if the study also reported a cognitive function outcome (i.e. studies reporting only a brain structure outcome with no measure of cognitive function were excluded).

##### Excluded outcomes

In line with recommendations from the Cochrane Dementia and Cognitive Improvement Group [30], surrogate outcomes were ineligible, for example:

- Brain structure and function, in the absence of a measure of cognitive function
- Biomarkers

#### Types of studies

Cohort studies and nested case-control studies were eligible for inclusion in the review.

Broadly, these types of designs can be described as follows.

- Cohort: “a study in which a defined group of people (the cohort) is followed over time, to examine associations between different … [exposures] and subsequent outcomes” [31].
- Nested case-control: a study in which “Individuals experiencing an outcome of interest are identified from within a defined cohort (for which some data have already been collected) and form a group of ‘cases’. Individuals, often matched to the cases, who did not experience the outcome of interest are also identified from within the defined cohort and form the group of ‘controls’.” Data characterising prior exposure “are collected retrospectively” [31]. Data on alcohol exposure should be collected from existing records, since those experiencing cognitive decline may not be able to provide sufficiently valid and reliable information about their prior exposure.

In line with current Cochrane guidance, decisions about study eligibility were based on the assessment of the study design features listed in Table 2 rather than labels (‘cohort’ or ‘case-control’) or broad definitions of each type of study.

**Table 2:** Design features for determining study eligibility and description (adapted from [31])

Study design feature	Prospective cohort	Retrospective cohort	Nested case-control
(1) A comparison between two or more groups of participants with different levels or patterns of alcohol consumption (‘yes’ = cohort or NCC)	Yes	Yes	Yes
(2a) Participants were allocated to groups based on different levels or patterns of alcohol exposure	Yes	Yes	No (based on outcome)
(2b) Participants were allocated to groups on the basis of outcomes	No	No	Yes
(3) The following parts of the study were prospective:			
a. Identification of participants	Yes	No	Yes
b. Assessment of alcohol consumption and allocation to alcohol consumption categories prior to follow-up measures of cognition	Yes	No	Yes (from existing records)
c. Assessment of outcomes (baseline cognition)	Yes	Possibly	Yes
d. Generation of hypotheses	Yes	Yes	Yes
Assessment of comparability of groups was based on:			
• Potential confounders	Possibly	Possibly	Possibly
• Outcome variables at baseline	Possibly	Possibly	No

##### Definition of study ‘baseline’

Prospective assessment of alcohol consumption (Table 2, design feature 3b) was judged to have occurred if data on alcohol consumption was collected at least 6 months prior to the first ‘follow-up’ measure of cognition. We defined the last point at which alcohol was measured as the ‘baseline’ for the study (an important consideration for studies with alcohol consumption data collected at multiple time points). A ‘baseline’ assessment of cognition may have been made at this point, but was not a requirement for inclusion in the review (Table 2, design feature 3c). Studies that collected alcohol data concomitantly with follow-up measures of cognition (i.e. beyond ‘baseline’) were excluded unless they reported an analysis based only on the alcohol measures taken prospectively. To avoid ambiguity when describing data collection points, we used a standardised nomenclature for each point (T0 being the first measurement point, then each subsequent point numbered sequentially: T1, T2, T3, etc.).

While eligible for this review, randomised trials examining the effects of different levels and/or patterns of alcohol exposure are unlikely to be conducted because of ethical concerns and the length of follow-up required to measure long-term cognitive outcomes.

##### Excluded designs

Case-control studies were excluded, except for nested case-controls. Case-control studies compare “people with a specific outcome of interest (‘cases’) with people from the same source population but without that outcome (‘controls’), to examine the association between the outcome and prior exposure” [31]. This design is unsuitable for addressing the objectives of this review since it is unlikely to be possible to obtain valid and reliable estimates of prior exposure to alcohol from individuals with the outcome of interest (cognitive impairment).

Studies using other designs (before-after comparisons, cross-sectional studies) were excluded since it is difficult (if not impossible) to attribute observed changes in outcomes to the exposure [31]. Studies that collected longitudinal data, but only presented analyses based on concomitant measures of alcohol and cognition, were also excluded on this basis.

##### Date and language restrictions

Studies published from 2007 onwards were eligible for inclusion. Studies published in languages other than English were excluded. A recent study has shown that the exclusion of studies in languages other than English rarely impacts the results and conclusion of a review [32], a finding that is consistent with an earlier study that found no evidence that English-language restriction introduces systematic bias in meta-analytic results [33].

### Search methods for identification of studies

Our approach combined searching for systematic reviews as well as primary studies. Searches were limited to bibliographic databases and checking the reference lists of eligible studies.

#### Systematic reviews

An independent evidence evaluation on the health effects of alcohol consumption commissioned by NHMRC [34] listed 13 systematic reviews (published between 2007 and 2016) that related to alcohol and cognitive impairment, and a further two systematic reviews were identified from an overview by Rehm et al [6]. From these reviews, we retrieved all primary studies that met the eligibility criteria. In addition, we searched MEDLINE and Embase for systematic reviews published since 2016 and ensured that any relevant primary studies included in these reviews were considered for inclusion.

#### Primary studies

The primary studies we identified from existing systematic reviews served as the initial source of studies. We used information about how these studies were indexed (i.e. thesaurus terms, text words) to help develop and validate the search strategy for primary studies. This technique (referred to as relative recall) is particularly useful when there are a reasonable number of studies (~20).

Independently of the search for systematic reviews, we searched for primary studies relevant to the review question published since January 2007. No language or geographic limitations were applied to the search. Searches were limited to MEDLINE, Embase, and PsycINFO.

The search strategy for Ovid MEDLINE was based on an assessment of the 2009 systematic review by Anstey [29] and the more recent 2017 meta-analysis by Xu [7]. The searches conducted for the Anstey review were very broad, generating over 33,000 citations, of which 15 were ultimately included in the meta-analysis. The MEDLINE search (see Additional file 2, Appendix 2) retrieved all the studies included in the Anstey review but is considerably more precise. This search also retrieved all seven additional studies included in the meta-analysis by Xu.

We decided not to include the text word ‘impairment’ as a stand-alone term since records retrieved using this text word (not already retrieved by the text words ‘cognition’ or ‘cognitive’) were mostly concerned with kidney or liver impairment, or some other impairment, and unrelated to cognition.

The MEDLINE search was translated for Embase and PsycINFO, incorporating each database’s relevant thesaurus terms for alcohol, dementia/cognitive impairment, and study design (see Additional file 2, Appendix 2).

Beyond database searching, we checked the reference lists of eligible studies for additional relevant publications.

### Data collection and analysis

#### Selection of studies

Citations identified from the literature searches and reference list checking were imported to EndNote and duplicates were removed. Three reviewers independently screened a sample of 109 citations to pre-test and refine coding guidance based on the inclusion criteria. Disagreements about eligibility were resolved through discussion. One reviewer (SB, JR, or SM) then each screened about a third of the remaining citations (grouped by year of publication) for inclusion in the review using the pre-tested coding guidance.

Full-text of all potentially eligible studies were retrieved. A sample of full-text studies was independently screened by two reviewers (SB and JR) until concordance was achieved (~15%; 37/228 of full-text studies screened). The remaining full-text studies were screened by one reviewer (SB or JR). All included studies, and those for which eligibility was uncertain, were screened by a second reviewer (JR or SB). Disagreements or uncertainty about eligibility were resolved through discussion, with advice from the review biostatisticians (JM, AF, or both) to confirm eligibility based on study design and analysis methods. Further information was sought from the authors of two studies (Piumatti 2018, Wardzala 2018) to clarify methods and interpretation of the analysis.

Citations that did not meet the inclusion criteria were excluded and the reason for exclusion was recorded at the full-text screening.

Cohort names, author names, and study locations, dates and samples characteristics were used to identify multiple reports arising from the same study (deemed to be a ‘cohort’). These reports were matched, and data extracted only from the report that provided the most relevant analysis and complete information for the review. In most cases, the decision was based on the outcome reported (global function was prioritised).

#### Data extraction and management

For each included study, one review author (SB, JR or JM) extracted data relating to study characteristics using a pre-tested data extraction and coding form. A second author (SB, JR, or JM) independently verified data relating to alcohol consumption categories (including conversions to grams per day) and outcome measures. One author extracted quantitative data (JM). Discrepancies were resolved through discussion, and advice sought from the review content expert (SW) or biostatistician (AF) if the agreement could not be reached or for more complex scenarios.

Pre-testing of the data extraction and coding form was done on two studies purposefully selected from the included studies to cover the diversity of data types anticipated in the review. Advice was sought from the review content expert (SW) and biostatisticians (JM or AF) to ensure data were extracted as planned. Revisions to the data extraction form were made as required to maximise the quality and consistency of data collection.

We extracted information relating to the characteristics of included studies and results as follows.

1. Study identifiers and characteristics of the study design

- Study references (multiple publications arising from the same study were matched to an index reference, which is the study from which results were selected for analysis or summary)
- Study or cohort name, location, and commencement date
- Study design (categorised as ‘prospective cohort study’, ‘nested case-control study’, or ‘other’ using the checklist of study design features developed by Reeves and colleagues, [31])
- Funding sources and funder involvement in the study

2. Characteristics of the exposure and comparator groups

- Levels of alcohol consumption as defined in the study, including details of how consumption was measured and categorised, and information required to convert data for reporting and analysis
  - Qualitative descriptors of each category, if used (e.g. never or non-drinker, abstainer, former drinker, low/moderate/heavy consumption)
  - Upper and lower boundaries of each category (e.g. 1 to 29 g per day; 5.1 to 10 units per week based on a standard drink in the UK)
  - Group used as referent category (comparator) in analyses and how defined
  - Units of measurement (e.g. standard units of alcohol per day and definition of unit)
  - Method of collecting alcohol consumption data (e.g. retrospective survey involving recall of alcohol consumption over different periods of life; intake diaries to measure current alcohol consumption); time points at which exposure data were collected
  - Sample size for each exposure group at each measurement point and included in analysis; number lost to follow up [these data were used in the analysis and risk of bias assessment]
  - Any additional parameters used to derive each category or exposure measure (e.g. alcohol consumption at each drinking occasion; frequency of drinking; recall period)
- Patterns of exposure
  - Any additional data not listed above that characterises and quantifies different patterns of alcohol exposure (e.g. consumption on heaviest drinking day; diagnosis of an alcohol-use disorder such as dependence or harmful drinking, and the method of assessment; definition of other frequency-based categories used to characterise patterns of drinking such as occasional drinking or infrequent consumption)
- Duration/length of exposure period at study baseline and follow-up (directly reported or data that can be used to calculate)
- Age at commencement of drinking (initial exposure)

3. Characteristics of participants

- Age at baseline and follow up, sex, ethnicity, co-morbidities, socio-economic status (including education), use of licit or illicit drugs, family history of alcohol dependence
- Other characteristics of importance within the context of each study
- Eligibility criteria used in the study

4. Outcomes assessed and results

- Outcomes domains (e.g. cognition, brain structure, function in daily life). We categorised specific domains of cognitive function by the domains used in the DSM-5 for diagnosis of cognitive impairment (Table 1).
- For cognition outcomes:
  - Measurement method (e.g. Montreal cognitive assessment) and time points
  - Potential confounders, co-exposures and other sources of bias mentioned in the paper [35]. Baseline statistics of the confounders to allow assessment of the comparability of the exposure groups.
  - Results including: summary statistics (means and standard deviations, or number of events for cognitive outcomes that have been dichotomised, and sample size) in each exposure category, unadjusted and adjusted estimates of the associations (e.g. mean differences, confidence intervals, t-values, p-values, or risk ratios/odds ratios for binary outcomes) overall and stratified by the specified subpopulations, where possible. For adjusted estimates, we extracted information on the analysis method, how confounding was adjusted, and which confounders were adjusted for.
  - Data required to assess risk of bias (see ‘Assessment of risk of bias of included studies’ section) and report the methods that influenced judgements [35]. In particular, we collected and summarised information about study design features that potentially introduced selection bias (e.g. a lag time between initiating drinking and enrolment to the study), or bias through misclassification of alcohol consumption status (e.g. measures that do not capture variation in patterns of drinking over time).

#### Assessment of risk of bias of included studies

One author (MP) assessed risk of bias for each included study using ROBINS-I (Risk Of Bias In Non-randomized Studies of Interventions) tool [36], and a second author (SB) independently verified the assessments and summarised study design features on which judgements were made. Discrepancies were resolved through discussion, with advice from a third reviewer (JM) if the agreement could not be reached, for more complex scenarios or judgements of critical risk of bias (see below). To ensure concordance, the assessment process was piloted by all assessors (JM, SB, and MP) on two included studies.

ROBINS-I was developed for “evaluating risk of bias in estimates of the comparative effectiveness (harm or benefit) of interventions” from non-randomised studies (i.e. where randomisation was not used to allocate individuals to comparison groups) [36]. While alcohol is generally considered an exposure, ROBINS-I has been successfully applied to equivalent studies (e.g. those examining the association between change in body size and mortality) and has advantages over checklist approaches in that it facilitates an overall judgement of RoB that can be incorporated in the analysis and the GRADE assessment [36, 37].

ROBINS-I requires assessment of the following seven domains:

1. Bias due to confounding (see below ‘Pre-specification of confounding factors and co-exposures’)
2. Bias in selection of participants into the study (e.g. we considered whether any lag between initiating drinking and enrolment into the study was likely to introduce bias)
3. Bias in classification of interventions (e.g. we considered whether the method of measuring alcohol consumption could lead to misclassification of the level of consumption due to problems with recall, underreporting, and not capturing variation in consumption over time)
4. Bias due to deviations from intended interventions (exposures)
5. Bias due to missing data
6. Bias in measurement of outcomes
7. Bias in selection of the reported result

It is recommended that users applying ROBINS-I should consider in advance the confounding factors and co-interventions that have the potential to lead to bias in included studies. These are listed at the end of this section.

Within each domain, we judged risk of bias as “low” (comparable to a well-performed randomised trial), “moderate” (sound for a non-randomised study), “serious” (there are some important problems) or “critical” (the study is too problematic to provide useful evidence).

We rated the overall risk of bias for each result based on the most serious risk of bias judgement across any of the seven domains (i.e. overall risk of bias is “serious” if at least one domain is rated “serious”). If we judged a result to be at “critical” risk of bias on the first domain (bias due to confounding), we did not assess other domains, since the overall risk of bias for the result would be “critical” by default. Studies that were judged to be at “critical” risk of bias overall were excluded from the summary and syntheses of results, and they do not contribute to our conclusions. For each study and result (outcome) assessed, we report our judgement of risk of bias by domain and provide a rationale for the judgment with supporting information about study methods.

##### Pre-specification of confounding factors and co-exposures

Confounding domains are “prognostic variables (factors that predict the outcome of interest)” that also predict the exposure at baseline [36]. ROBINS-I defines important confounding domains as those “for which, in the context of [a specific] study, adjustment is expected to lead to a clinically important change in the estimated effect of the [exposure]”. We considered the following confounding domains as important for most or all studies since they have been shown to be associated with alcohol consumption and are prognostic factors for cognitive impairment: age, sex, socioeconomic factors (especially education), smoking, and co-morbidities (especially diabetes, and obesity). Co-exposures were assessed on a study-by-study basis.

For GRADE assessments, it was necessary to summarise the risk of bias assessments across studies for each outcome. We followed recent GRADE guidance for making these judgements [37]. These summary assessments of risk of bias were used in determining the overall certainty of the body of evidence using GRADE, and the basis for each is reported as footnotes to the summary of findings tables.

#### Measures of association

Cognition was assessed using continuous measures with varying scales and neurocognitive tests across the studies. The standardised mean difference (SMD) was therefore used to standardise the associations so that they were comparable across studies. In some studies, the measures of cognition were dichotomised and analysed as binary outcomes. These studies reported odds ratios along with 95% confidence intervals. For these studies, we converted the odds ratios (ORs) and their confidence limits to SMDs using a simple approximation proposed by Chinn [38]. The accuracy of the resulting SMD variances was assessed, and where necessary, adjustments were made to these variances so that when they were back-transformed to the (log) OR scale, they yielded equivalent variances to the observed (log) OR variances. In the circumstance where results from multiple multivariable models were presented, we extracted associations from the most fully adjusted model, except in the case where an analysis adjusted for a possible intermediary along the causal pathway (i.e. post-baseline measures of prognostic factors (e.g. smoking, drug use, hypertension)) [39].

#### Unit of analysis issues

In this review, the unit of analysis issue that arose was multiple estimates of association calculated for different levels of alcohol consumption within the same study. These estimates are correlated since each level of alcohol consumption is compared against the same group of participants (i.e. current non-drinkers). Methods used to adjust for the correlation between the estimated associations are described in the ‘Data synthesis’ section.

#### Assessment of heterogeneity

We assessed heterogeneity through visual inspection of the study-specific dose-response curves, formal testing for heterogeneity using the *Χ*^2^ test (using a significance level of α=0.1), and quantified heterogeneity in the study-specific dose-response coefficients using the *I*^2^ statistic.

#### Assessment of reporting biases

We had planned to investigate the potential for small-study effects using contour-enhanced funnel plots and formal statistical tests for funnel plot asymmetry if there were at least 10 studies included in a synthesis. However, all syntheses included fewer than 10 studies.

#### Data synthesis

##### Investigation of the association between levels of alcohol consumption and cognition

In planning the review, we anticipated that there may be too little data to conduct a dose-response analysis. We, therefore, planned to undertake pair-wise comparisons of the effects of never drinking or very low-level drinking (0 to < 10 g/week) with different levels of alcohol consumption (≥ 10 g/week and < 10 g/day; ≥ 10 g/day and < 20 g/day; ≥ 20 g/day and < 30 g/day; ≥ 30 g/day and < 40 g/day; ≥ 40 g/day and < 50 g/day; ≥ 50 g/day).) We did not undertake these analyses since all studies that contributed data suitable for synthesis were able to be included in the dose-response analyses. The dose-response analyses provide a more complete understanding of the relationship between alcohol consumption and the size of the SMDs since all data are modelled in a single synthesis. Further, from these models, the size of any effect on cognition (SMDs) can be predicted at any level of alcohol consumption (within the observed range).

##### Investigation of the dose-response relationship between levels of alcohol consumption and cognition

Analyses were undertaken to identify and characterise dose-response relationships between levels of alcohol consumption and cognition. For each study, the relationship between the SMD of cognition (compared with abstainers) and alcohol consumption was modelled using a restricted cubic spline with three knots (at the 10th, 50th, and 90th percentiles of alcohol consumption), accounting for correlation amongst the SMDs. The estimated study-specific dose-response coefficients and their covariance matrices were combined using a random-effects multivariate model [40]. The between-study variance of the dose-response coefficients was obtained using restricted maximum likelihood. Studies assessed as at a critical risk of bias were not included in the dose-response analysis.

In studies that reported alcohol consumption in different units (e.g. millilitres or standard drinks per days), we converted these to grams per day using the relevant country’s standards [41]. For each category of alcohol consumption, we used the median or mean of alcohol consumption in grams per day when presented. When not presented, we assigned the midpoint of the category as the dose value. When the largest dose category was reported without an upper bound, the dose value assigned was calculated as the lower bound of the largest dose category plus the width of the previous (second-to-largest) category [42].

The combined dose-response curves, along with 95% confidence intervals, were presented graphically and in tabular form (presenting predicted standardised mean differences of cognition for different alcohol consumption levels).

We examined the robustness of the combined dose-response model to different locations of the knots. We had also planned to examine the robustness of the combined dose-response model to different numbers of knots, but we did not do this. For each dose-response analysis, we were limited to a maximum of three knots due to some studies only reporting three levels of alcohol consumption.

The dose-response models were fitted using the package dosresmeta in the statistical program R [43].

##### Subgroup analyses

We present the dose-response relationships for females and males separately where possible (i.e. where the study was undertaken with only one sex, or the results were reported separately by sex within a study). For other potential modifying factors (age, co-morbidities, drug-taking, or a family history of alcohol use), no studies were limited to a particular subpopulation, nor did they report associations separately by particular subpopulations within a study.

##### Sensitivity analyses

We had planned to undertake sensitivity analyses examining the robustness of the results to the method of alcohol measurement (intake over multiple time points versus once) and limiting to studies that reported results for ‘never’ drinkers. We did not undertake these sensitivity analyses due to only a small number of studies available for any of the dose-response analyses (i.e. a maximum of six studies).

##### Summary of results from single studies

For studies that were not able to be included in the dose-response analyses, we summarised the risk of bias assessment, the study characteristics, the reported associations (including 95% confidence intervals and *p* values where reported), and provided an interpretation. We had planned to present reported associations using forest plots, but because of incomplete reporting and the variability in the measures of association (e.g. linear trends, quadratic trends, hazard ratios, odds ratios) used across the studies, this was not possible.

#### Summary of findings tables and assessment of certainty of the body of evidence

We assessed the certainty of the evidence for results from the dose-response analysis using the GRADE approach. In accordance with the detailed GRADE guidance [15, 37], the following domains were assessed (as briefly summarised below) and a judgement made about whether there were serious, very serious or no concerns in relation to each domain.

1. Risk of bias. Based on the summary assessment across studies for each outcome reported for a comparison (see ‘Risk of bias’ section). The assessment was based on guidance for ROBINS-I [35] and GRADE [37].
2. Inconsistency. We assessed (1) whether there was heterogeneity in the observed effects across studies that suggested important differences in the effect of the exposure (based on visual inspection of data and statistical tests of heterogeneity), and (2) whether this could be explained (e.g. by variance in effects across subgroups if data were available).
3. Imprecision. We assessed whether the interpretation of the upper and lower confidence limits leads to conflicting interpretations about the effect of the exposure (e.g. benefit and appreciable harm).
4. Indirectness. We assessed whether there were differences between the characteristics of included studies (PECO of included studies) and the review question (in terms of the review PECO) such that the effects observed in the included studies were unlikely to apply directly to the review question. For example, studies with multiple measures of alcohol over time, and longer-term outcome follow up at multiple time points, were considered to provide the most direct evidence of the cognitive effects of life-long alcohol-use patterns. In general, this information was used to interpret results, rather than downgrade.
5. Publication bias. Our judgement of suspected publication bias was based on the assessment of reporting bias as described in ‘Assessment of reporting biases’ section. Evidence of small-study effects and the absence of a plausible alternative explanation for these effects indicate that publication bias should be suspected.
6. Upgrading domains (large effect size, dose-response gradient, opposing plausible residual confounding). Recent GRADE guidance is that observational studies may start as high certainty evidence when ROBINS-I is used for the risk of bias assessment [37]. Doing so alters the assessment of GRADE upgrading domains since these domains examine the likelihood that any observed association could be explained by residual confounding, and are typically used to upgrade observational studies from low to moderate or high certainty. In line with one of the options presented in recent GRADE guidance, we considered the upgrading domains when assessing confounding and selection bias using ROBINS-I.

GRADEpro GDT software (www.gradepro.org ) was used to record decisions and derive an overall GRADE (high, moderate, low, or very low) for the certainty of evidence for each outcome, using the GRADE rules in which observational studies assessed using ROBINS-I begin as ‘high’ certainty evidence (score=4) and can be downgraded by −1 for each domain with serious concerns or −2 for very serious concerns [37].

A summary of findings table (using the evidence profile format for guidelines) was prepared using the GRADEpro GDT software. For each result from the dose-response analysis, the evidence profile includes estimates of the effects of alcohol exposure reported as standardised mean differences, and the overall GRADE (rating of certainty). The evidence profile also includes (1) the study design(s), number of studies contributing data (the type and size of the evidence base), (2) our assessment of each of the domains (risk of bias, inconsistency, indirectness, imprecision, publication bias), and (3) a statement interpreting the evidence (clinical impact) for each outcome (by population subgroup). Footnotes are included to explain judgements made about downgrading the rating of the certainty of the evidence.

## Results

### Results of the search

#### Systematic reviews

The search of MEDLINE and Embase for systematic reviews published since the NHMRC evidence evaluation was conducted on 13 February 2018 and retrieved 251 records after duplicates were removed. Eleven systematic reviews were potentially eligible and we screened the included studies of these reviews, together with those from relevant systematic reviews from the 13 identified in the NHMRC overview report, to identify relevant primary studies. We did not identify any additional potentially eligible studies from these sources.

#### Primary studies

**Fig. 1:**
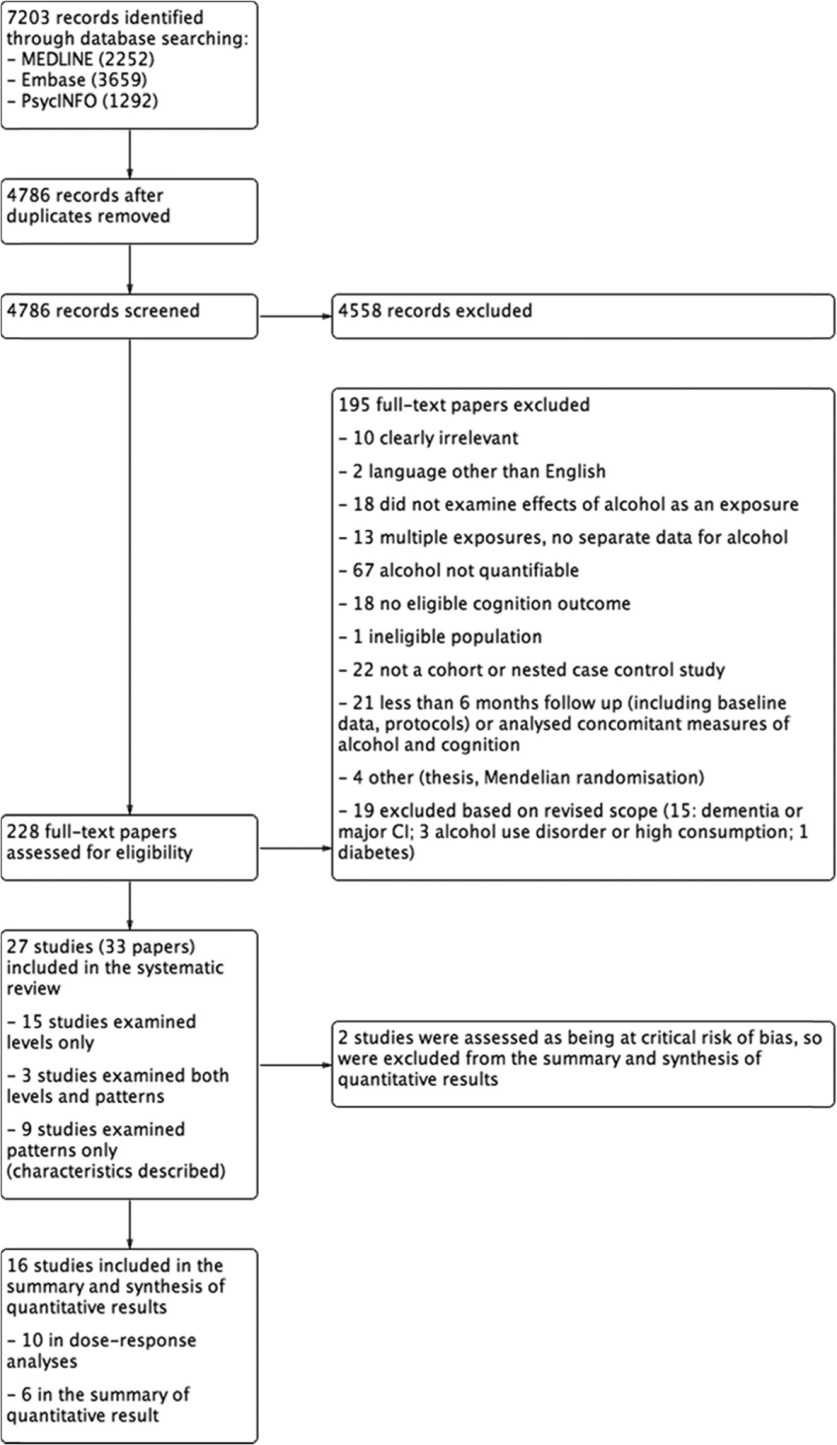
Study flow diagram

The searches of MEDLINE, Embase, and PsycINFO for primary studies were conducted on 9 April 2018. After removing duplicates, we screened 4786 records. Figure 1 shows the flow of references through the review. (See Additional file 2, Appendix 2 for the search results for each source.) The full-text of 228 papers were screened, from which 195 were excluded.

After screening and full-text review, we included 27 studies (reported in 33 papers). Of these, 15 studies examined the effects of different levels of alcohol consumption, three examined both different levels and patterns of alcohol consumption, and nine examined patterns only. Sixteen of 18 studies that examined the effects of different levels of alcohol intake were included in the summary and synthesis of quantitative results. Two of the 18 were assessed as at a critical risk of bias (Hassing 2018, McGuire 2007), excluding them from the summary and synthesis of quantitative results. Study characteristics are reported for these studies, and the nine studies examining patterns.

Included studies were assigned a unique identifier (first author family name and year of publication) which is used throughout the review. A list of included studies and references to all linked papers is in Additional file 2, Appendix 3.

### Description of studies

#### Included studies

##### Studies examining the effects of different levels of alcohol consumption

Characteristics of the 18 included studies that examined the effects of different levels of alcohol consumption are summarised in Table 3 and reported in more detail in Table 4.

**Table 3:** Comparison of characteristics of studies that examined the effect of different levels of alcohol consumption

	Study dates (years from T0; bold= ‘baseline’)										Alcohol category* (bold=referent)							Cognitive function (bold=selected result)								
Study ID (sample size at T0; % female)	Age (T0)	T0	T1	T2	T3	T4	T5	TX	Age final follow-up	Length of follow-up from b/l	0 to < 10 g/week	≥ 10 g/week to < 10 g/day	≥ 10 to < 20 g/day	≥ 20 to < 30 g/day	≥ 30 to < 40 g/day	≥ 40 to < 50 g/day	≥ 50 g/day	Global function	MCI diagnosis	Complex attention	Executive function	Learning and memory	Language	Perceptual motor ability	Social cognition	Outcome description (details of selected result)
Arntzen 2010 [44] (5033; 56%)**	58 (mean)	**A**	C (~7)						~65	~7	**X**	X								X		**X**				SCD: learning and memory (immediate and delayed recall)
Downer 2015 [45] (664; 56%)**	42 (mean)	**A**	C (~28)	C (~34)					75 (mean)	~34	**X**	X		X		X		**X**			X	X				GCF: Average of Z-scores on 11 tests (incl. memory, executive function, language, complex attention)
Hassing 2018 [46] (305; 56%) critical RoB	~56–66	**A**	C (~24)	C (~26)	C (~28)	C (~30)	C (~32)		83 (mean)	~32		X	X	X				**X**				X		X		GCF: MMSE score (change over time)
Heffernan 2016 [47] (821; 55%)**	70–90	**A, C**	C (~2)	C (~2)					~74–94	~4	**X**		X			X				X	X	**X**	X	X		SCD: learning and memory (delayed recall)
Hogenkamp 2014 [48] (652; 100%)	70	**A, C**	C (~7)						77	~7	**X**	X	X	X						X	**X**					SCD: executive function (TMT-B)
Horvat 2015 [49] (28,947; 55%)**	45–69	**A, C**	C (~4)						47–78	~4	**X**	WM	W	WM			M			X		**X**	X			SCD: learning and memory (delayed recall)
Kesse-Guyot 2012 [50] (3088; 46%)**	45–60	**A**	C (~13)						~58–73	~13	X	WM		**WM**		WM	WM	**X**			X	X	X			GCF: Average of T-scores on 6 tests (executive function; learning & memory; language)
Kitamura 2017 [51] (1814; 60%)**	44–79	**A**	C (~3)						~47–82	~3	**X**		X		X		X	**X**								GCF: cognitive impairment (MMSE <24)
Lang 2007 [52] (13,333; 57%)	≥ 65	**A**	C (~4)						≥ 69	~4	X	**X**		X	X			**X**								GCF: Binary “poor function” (bottom quintile for sum of scores on 3 tests)
McGuire 2007 [53] (2572; 66%) critical RoB	≥ 70 (mean 76)	A	**A, C** **(~2)**	C (~2)					~80	~2	**X**	X	X					**X**								GCF: Binary “low” or “high” (based on cut-off score on 2 tests)
Piumatti 2018 [54] (13,342; 55%)	40–73	**A, C**	C (~5)						~45–78	~5	continuous variable									**X**						SCD: complex attention (mean reaction time over 7 test trials)
Richard 2017 [55] (1334; 54%)**	55–84	**A**	C (~4)	C (~4)	C (~4)	C (~4)	C (~4)	C^†^ (~4)	85	14±8 (med-ian)	**X**	W	M	W	M	W	M	**X**								GCF: Binary “impaired” or “healthy” based on cut-off on Z-scores (age, sex, education adjusted MMSE)
Sabia 2011 [56] (4073; 0%)**	~45–55	A	A (1)	A (1)	A (1)	A (1)	A (1)	**A (T6-9)**, C (T10, 11)	~55–65	1	M	M	**M**	M	M					**X**						SCD: complex attention (digit symbol substitution test)
Sabia 2014 [57] (7153; 29%)**	35–55	A	A (~5)	**A, C (~5)**	C (~5)	C (~5)			55–80	~10	X	**W** **M**	W	W M		M		**X**			X	X				GCF: Average of Z-scores on 4 tests (executive function; learning & memory)
Samieri 2013 [58] (6174; 100%)	≥ 60	**A**	C(5.6)	C(~2)	C(~2)				~79 (mean)	~10	**X**	X		X				**X**				X				GCF: Average of Z-scores on 5 tests (TICS, learning & memory, language)
Solfrizzi 2007 [59] (1445; 44%)	65–84	**A, C**	C						~68–87	3.5	**X**	X		X	X				**X**							MCI: hazard ratio for incident MCI (Petersen diagnostic criteria)
Stott 2008 [60] (5,804; 52%)**	70–82	**A, C**	C (~1)	C (~1)	C (~1)	C (~1)			~73–85	3.2	**X**	X	M					**X**		X		X				GCF: MMSE score
Wardzala 2018 [61] (486; 75%)	≥ 80	**A, C**	C (~1)	C (~1)	C (~1)	C (~1)	C (~1)	C (~1)	~86–91	~5–7	**X**	W M		W M				**X**		X	X	X	X			GCF: MMSE score (change over time)

Abbreviations: *GCF*: Global cognitive function, *SCD*: Specific cognitive domain, *MCI*: Mild cognitive impairment, *A*: Measure of alcohol intake, *C*: Measure of cognition, *M:* Men, *W*: Women, *X*: Men and women. *DSST*: Digit symbol substitution test, *DSCT*: Digit symbol coding test, *TMT*: Trail making test, *MMSE*: Mini-Mental State Exam, *TICS*: Telephone Interview for Cognitive Status, *RoB*: Risk of bias

*Based on mean or median alcohol consumption, or midpoint of specified category (if average consumption for group not reported). For the largest dose category, if an upper bound was not specified then the assigned dose value was calculated as the lower bound of the largest dose category plus the width of the previous (second-to-largest) category. Alcohol category: Bolded entry (**X, M or W**) was used as referent in study

**Included in dose-response analysis for ‘females’ only, ‘males’ only, or ‘females and males’

^†^Follow up until age 86—number of follow-up measures depends on age at baseline

**Table 4:** Detailed characteristics of studies examining the effects of different levels of alcohol consumption

Study details	Sample	Alcohol exposure categories	Details of the included article	Study dates
Arntzen 2010 [44] Norway Cohort name: the Tromsø Study Serious risk of bias	Based on 5,033 men and women (56% female) with a mean age of 58 years at point of first alcohol measure (T0) and ~65 years at final cognitive assessment. Substudy of the Tromsø Study cohort which was established in 1974 to examine cardiovascular risk among people aged 25-85 years.	Teetotaller: not defined Category (referent): < 1 glass per fortnight for women or men (midpoint = 0.5 g/day) Category: 1–2 glasses per fortnight for women or men (midpoint = 1.4 g/day) Category: 3–4 glasses per fortnight for women or men (midpoint = 3.4 g/day) Category: > 5 glasses per fortnight for women or men (midpoint = 5.0 g/day) Grams per drink: Not reported. Assumed 12–15 g (RARHA 2015).	Observational cohort examining associations between different levels of alcohol consumption and cognitive function. Inclusion criteria: Eligible participants were aged 25–85 years at start of T0 (December 1994; 100% of those aged 55–74 and 5–10% of other birth cohorts were invited). Exclusion criteria: self-reported stroke; incomplete alcohol data; incomplete covariate data; no data for any of the 4 cognitive tests. Alcohol ascertainment: Current: self-report questionnaire asking about frequency (“how many times a month do you normally drink alcohol” and quantity “how many glasses of (beer/wine/spirits) do you normally drink” in a fortnight. Recall: not reported. Lifetime: “Are you a teetotaller”. Cognitive function: Learning and memory (immediate and delayed recall of 12 nouns), complex attention (Digit Symbol-Coding test from Wechsler adult intelligence scale (WAIS); Tapping test scores for dominant and non-dominant hand). Mean difference in raw scores. Higher score = better cognition.	Study period: 1994-2001 Alcohol exposure: single assessment at baseline (T0: 1994-95) Outcome measures: single assessments at ~7 year from T0. (T1: 2001) Length of outcome follow-up: ~ 7 years
Downer 2015 [45] United States Cohort name: Framingham Heart Study Offspring Cohort Serious risk of bias	Based on 664 men and women (56% female) mean age of 41.8 years at point of first alcohol measure (T0) and 74.8 at final measure of cognition (T2). Substudy of the Framingham Heart Study Offspring Cohort among those actively participating in the cohort when cognitive testing was first introduced (1999)	Abstainer (referent): 0 drinks per week Light: 1–6 drinks per week (mean = 5.6 g/day) Moderate: 7–14 drinks per week (mean = 20 g/day) Heavy: 15–34 drinks per week (mean = 41.6 g/day) Grams per drink: Not reported. Assumed 14 g based on US standard.	Observational cohort examining associations between different levels of alcohol consumption in midlife and cognitive function at late life. Inclusion criteria: Eligible participants were 60 years or older at first measure of cognition (T1). Exclusion criteria: stroke, Alzheimer’s disease, other dementia; did not receive cognitive testing or an MRI within 6 months, no APOE genotype data; history of consuming ≥ 5 drinks almost daily (based on screening question administered at T2). Alcohol ascertainment: Current: self-report questionnaire asking about quantity (“how many bottles/glasses/drinks of beer/wine/cocktails”) consumed per week. Recall: 12 months. Lifetime: screening question to exclude those who had drunk ≥ 5 drinks almost daily at any time of life. Cognitive function: Global cognitive function based on average of standardised individual scores from 11 tests measuring: language (Boston naming test), complex attention (TMT-A and B), perceptual motor (Hooper Visual Organisational test), learning and memory (tests of immediate and delayed recall assessing: visual memory, verbal memory, and learning), abstract reasoning. Test results converted to Z-scores ((individual score - sample mean)/SD). Higher scores = better cognition. Other outcomes reported: learning and memory, executive function, brain volume.	Study period: 1971-2008 Alcohol exposure: single assessment at baseline = ‘midlife’ (T0: 1971). (‘Late life’ measure exclude from the SR because analyses are cross-sectional) Outcome measures: two assessments, at ~ 6 year interval (range 1.5-8 years) (T1, T2: 1999-2002; ~2005-2008) Length of outcome follow-up: ~ 34 years from T0.
Hassing 2018 [46] Sweden Cohort name: none—data from Swedish Twin Registry Critical risk of bias	Based on 305 men and women (56% female) age ~ 56 to 66 years at point of first alcohol measure (T0) and mean age of 83 years at first measure of cognition (T1). Analysis of data from the Swedish Twin Registry (established late 1950s) and the OCTO-Twin study on cognitive ageing (started 1991-93)	Abstainer: excluded from analyses Occasional: < 1 drink per week Low: ~4 drinks per week (midpoint g/day, not estimable) Moderate: ~8 drinks per week (midpoint g/day, not estimable) Heavy: > 15 drinks per week (no heavy drinkers in the sample) Categories were reported for descriptive purposes only. Alcohol consumption was analysed as a continuous variable (grams per week). Grams per drink: 12 g	Observational cohort examining associations between different levels of alcohol consumption in midlife and cognitive function at late life. Inclusion criteria: Eligible participants were twins on The Swedish Twin Registry, aged ≥80 years at first measure of cognition (T1; birth years 1901-1911). Exclusion criteria: non-drinkers (at T0: no information on how abstention was measured), dementia diagnosis at T1 (first measure of cognition), missing cognition data (T1), missing alcohol data (T0). Alcohol ascertainment: Current: self-report questionnaire asking about frequency (whether drank alcohol or not; how often) and quantity (how much consumed on a typical occasion, by type). Recall: not reported. Lifetime: not reported. Cognitive function: Global cognitive function (MMSE). Raw scores converted to T-scores (mean=50; SD=10). Smaller change in mean score over time = less cognitive decline. Other outcomes reported: learning and memory (subscale of Wechsler adult intelligence scale (WAIS): prose recall; Thurstone’s picture recognition test; Information task), perceptual motor ability (Block design test).	Study period: 1967-2001 Alcohol exposure: single assessment ‘midlife’ (T0: 1967) Outcome measures: 5 assessments, at ~ 2 year intervals (T1-T5: 1991-93, 1993-95, 1995-97, 1997-99, 1999-2001) Length of outcome follow-up: ~ 32 years from T0 to T5 (final cognition measure )
Heffernan 2016 [47] Australia Cohort name: Sydney Memory and Ageing Study Serious risk of bias	Based on 821 men and women (55% female) aged 70-90 years at point of first alcohol measure (T0) and ~74-94 years at final cognitive assessment.	Abstainers (referent): no alcohol (last 12 months) Low risk: > 0 to ≤ 2 drinks per day for women; > 0 to ≤ 4 drinks per day for men (weighted midpoint based on proportion of women in low risk group = 15 g/day) Risky: > 2 drinks per day for women; > 4 drinks per day for men (weighted midpoint based on proportion of women in risky group = 43 g/day) Grams per drink: 10 g based on Australian standard. Data also re-analysed using NIAAA categories (results not presented in SR).	Observational cohort examining associations between different levels of alcohol consumption and cognitive decline in specific domains. Inclusion criteria: Eligible participants were aged 70-90 years at T0, community dwelling. Exclusion criteria: MMSE <24; health conditions (psychotic symptoms, dementia, schizophrenia, bipolar disorder, multiple sclerosis, motor neuron, developmental disability, progressive malignancy); learnt English after age 10; 2 or fewer valid scores for measured domains; no alcohol data; unknown APOE. Alcohol ascertainment: Current: self-report in interview asking about frequency of drinking (monthly, weekly, daily) and “amount of drinks per drinking session”. Recall: last 12 months. Lifetime: ever “drank more heavily than in the last 12 months”; if no alcohol in last 12 months “had they ever consumed“. Cognitive function: Learning and memory (immediate and delayed recall: Logical Memory Story A; Rey Auditory Verbal Learning; Benton Visual Retention), executive function (Controlled Oral Word Association; Trail making test B), complex attention (Digit Symbol-Coding; Trail making test A), language (Boston Naming, Semantic fluency - animals), perceptual motor ability (Block design test). Scores transformed to quasi-z scores (using baseline mean and SD of participants with cognition ≥1 SD from mean) and averaged across tests. Higher z score = better cognition (change from baseline > -1.0 SD = decline).	Study period: 2005-2011 Alcohol exposure: single assessment at baseline (T0: 2005-07) Outcome measures: baseline (T0) and 2 follow-up assessments at ~2 year intervals. (T0-T2: 2005-2007; 2007-2009; 2009-2011) Length of outcome follow-up: ~ 4 years (mean 38 months)
Hogenkamp 2014 [48] Sweden Cohort name: Uppsala Longitudinal Study of Adult Men (ULSAM) Serious risk of bias	Based on 652 men aged 70 years at point of first alcohol measure (T0). Substudy of the ULSAM cohort which was established to identify metabolic risk factors for CVD.	Non-drinker: 0 drinks per day Category: > 0 to ≤ 1 drinks per day (mean = 5.4 g/day) Category: > 1 to ≤ 2 drinks per day (mean = 16.7 g/day) Category: > 2 drinks per day (mean = 28.9 g/day) Alcohol analysed as continuous variable, examining linear trends, so no referent. Grams per drink: 12 g	Observational cohort examining associations between different levels of alcohol consumption and cognitive function in older men. Inclusion criteria: Eligible participants were healthy males aged 70 years (T0),. Exclusion criteria: Cognitively unhealthy (MMSE <25), missing data on alcohol intake. Alcohol ascertainment: Current: self-report of usual intake of types of alcohol per week. Recall: not reported. Lifetime: not measured. Cognitive function: Specific cognitive domains (2 outcomes). Executive function (Trail making test part B [TMT-B]) and complex attention (Trail making test part A [TMT-A]). Higher raw scores = worse cognition (these are timed tests).	Study period: 1990-2001 Alcohol exposure: single assessment (T0: 1990-1994) Outcome measures: baseline and follow-up assessment ~7 years later. (T0-T1: 1990-1994; 1997-2001) Length of outcome follow-up: ~7 years from baseline (T0)
Horvat 2015^†^ [49] Eastern Europe (Russia, Poland, Czech Republic) Cohort name: HAPIEE (Health, Alcohol, and Psychosocial Factors in Eastern Europe) prospective cohort study Serious risk of bias	Based on 28,947 men and women (54.7% female) aged 45–69 years at point of first alcohol measure (T0).	Non-drinker: 0 g/day Light (referent): < 5 g/day for women (midpoint = 2.5 g/day); < 10 g/day for men (midpoint = 5 g/day) Moderate: ≥ 5 to < 20 g/day for women (midpoint = 12.5 g/day); ≥ 10 to < 40 g/day for men (midpoint = 25 g/day) Heavy: ≥ 20 g/day for women (midpoint = 27.5 g/day); ≥ 40 g/day for men (midpoint = 55 g/day)	Observational cohort examining associations between different levels and patterns (frequency, binge, problem drinking) of alcohol consumption and cognitive function in older adults. Inclusion criteria: Eligible participants were aged 45–69 years (T0), randomly selected from population registers and electoral lists. Exclusion criteria: none reported. Alcohol ascertainment: Current: self-report graduated frequency questionnaire (GFQ) asking about frequency of consumption and number of drinks (by alcohol type; not specified whether asked in relation to a typical occasion/week/other). Recall: last 12 months. Lifetime: not measured. Cognitive function: Specific cognitive domains (4 outcomes). Learning and memory (immediate recall of words in 3 x 1 minutes trials; delayed recall of words after other tests administered), language (verbal fluency, number animals named in 1 minute), complex attention (letter cancelled test for attention, mental speed, concentration). Test results were converted to Z-scores (mean =0; SD = 1) using whole sample means and SDs. Higher scores = better cognition.	Study period: 2002-2008 Alcohol exposure: single assessment (T0: 2002-2005; second assessment made at follow-up, but not used in prospective analysis) Outcome measures: baseline and follow-up assessments at ~ 4 year intervals. (T0-T1: 2002-2008) Length of outcome follow-up: 4 years from baseline (T0)
Kesse-Guyot 2012 [50] France Cohort name: SU.VI.MAX 2 cohort Serious risk of bias	Based on 3,088 men and women (46% female) aged 45-60 years (mean 52) at point of first alcohol measure (T0). Observational follow-up of SU.VI.MAX randomised trial of dietary supplements for prevention of cancer, heart disease and mortality.	Non-drinker (referent): 0 g/day for women or men Category: ≥ 0.1 to ≤ 4.9 g/day for women or men (midpoint = 2.5 g/day) Category: ≥ 5.0 to ≤ 14.9 g/day for women or men (midpoint = 9.95 g/day) Category (referent): ≥ 15.0 to ≤ 29.9 g/day for women or men (midpoint = 22.45 g/day) Category: ≥ 30.0 to ≤ 59.9 g/day for women or men (midpoint = 44.95 g/day) Category: ≥ 60.0 g/day for women (midpoint = 74.95 g/day); ≥ 60.0 to ≤ 89.9 g/day for men (midpoint = 74.95 g/day) Category: ≥ 90.0 g/day for men (midpoint = 119.9 g/day)	Observational cohort examining associations between different levels of alcohol consumption in midlife and cognitive function 13 years later. Inclusion criteria: Eligible participants were healthy adults aged 45-60 years (T0), and agreed to participate in the observational follow-up SU.VI.MAX. Exclusion criteria: incomplete cognitive tests, < 3/12 dietary records, missing values for any covariables. Alcohol ascertainment: Current: 24 hour dietary record (bimonthly over 2 years, randomly assigned across 2 weekend days and 4 week days) asking about the number alcoholic drinks (by type) and portion size (validated photographs of 7 portion sizes, including 2 extreme). Recall: 24 hours. Lifetime: not measured. Cognitive function: Global cognitive function based on mean of standardised individual scores from 4 tools measuring: learning and memory (RI-48 test - a delayed cued recall test), language (verbal fluency, number animals named and number words beginning with P in 2 minutes), executive function (forward and backward digit span; Delis- Kaplan trail-making test). Test results converted to T scores (rescaled to SD = 10; 1 point difference in score = 1/10 difference in SD). Higher scores = better cognition. Also reported results for specific domains.	Study period: 1994-2009 Alcohol exposure: single assessment at baseline (T0: 1994-1996) Outcome measures: single assessment (T1: 2007-2009) Length of outcome follow-up: ~ 13 years from T0.
Kitamura 2017 [51] Japan Cohort name: Murakami Cohort Study Serious risk of bias	Based on 1,814 men and women (60% female) aged 44-79 years at point of first alcohol measure (T0). Substudy of the Murakami Cohort Study which was established to examine risk factors for age-related disease.	Non-drinker or rare drinker (referent): < 1 g of alcohol per week Category: 1–149 g of alcohol per week (midpoint = 11 g/day) Category: 150–299 g of alcohol per week (midpoint = 32 g/day) Category: 300–449 g of alcohol per week (midpoint = 54 g/day) Category: ≥ 450 g of alcohol per week (midpoint = 75 g/day)	Observational cohort examining association between different levels of alcohol consumption (and other lifestyle factors) and cognitive impairment Inclusion criteria: Eligible participants were those aged 44-79 at T0, and participating in the Murakami Cohort. No information on eligibility criteria for cohort. Exclusion criteria: None reported. Alcohol ascertainment: Current: self-report questionnaire asking about frequency of consumption and amount (by alcohol type). Lifetime: no information. Recall period: not reported. Cognitive function: Global cognitive function (MMSE). Results reported as binary outcome in which cognitive impairment was defined as score <24.	Study period: 2011-2016 Alcohol exposure: single assessment at baseline (T0: 2011-2013) Outcome measures: single assessment. (T1: 2014-2016) Length of outcome follow-up: not reported. Assumed to be ~ 3 years from baseline (T0)
Lang 2007 [52] United States, United Kingdom Cohort name: English Longitudinal Study of Ageing (ELSA); U.S Health and Retirement Study (HRS) Serious risk of bias	Based on 13,333 men and women (57% female) aged 65 years or above at point of first alcohol measure (T0).	Non-drinker: 0 drinks per day Category (referent): > 0–1 drinks per day for men or women (midpoint = 7 g/day) Category: > 1–2 drinks per day for men or women (midpoint = 21 g/day) Category: > 2 drinks per day for men or women (midpoint = 35 g/day) Grams per drink: not reported (assumed 14 g based on USA standard and [62])	Pooled data from two observational cohorts examining the association between different levels of alcohol consumption and cognitive function, and between alcohol consumption and physical disability, and among older people. Inclusion criteria: Eligible participants were aged 65 years or above (T0). Exclusion criteria: none reported. Alcohol ascertainment: Current: self-report questionnaire asking “how many days per week” they drank alcohol and number of drinks consumed “on average” on drinking days (HRS: last year; ELSA: last 3 months). Lifetime: no information (HRS); non-drinkers who had quit were asked if they had done so for health reasons (ELSA). Cognitive function: Global cognitive function based on the sum of scores on three tests, word recall (mean of immediate and delayed word recall scores, score out of 10), numeracy (score out of 4), and specifying the date (day, date, month, year; score out of 4). A score in the bottom quintile was assessed as “poor cognitive function”.	Study period: 1998-2002 Alcohol exposure: single assessment at baseline (T0: 1998) Outcome measures: single assessment. (T1: 2002) Length of outcome follow-up: ~ 4 years (median: 50 months for HSE, 45 months for ELSA)
McGuire 2007 [53] United States Cohort name: Second Longitudinal Study of Aging (LSOA II) Critical risk of bias	Based on 2,572 men and women (66% female) aged 70 years or above at point of first alcohol measure (T0; mean age 76). Substudy of the LSOA II cohort which was established to examine health and, and the causes and consequences of health events among older persons (9447 men and women).	Non-drinker (referent): zero drinks per day (past year) One drink per day or less: ≤ 1 drink/day men or women (≤ 12 g/day, midpoint = 6 g/day) More than one drink per day: > 1 drink/day for men or women (> 12 g/day, midpoint = unknown) Categories based on NIAAA guideline s[63]. Grams per drink: not reported (assumed 12 g based on NIAAA guidelines)	Observational cohort examining association between different levels of alcohol consumption and cognitive impairment among people 70 years and over. Inclusion criteria: Eligible participants were aged 70 years (T0), and community-dwelling. Exclusion criteria: Cognitively impaired (1.5 SD units less than the cohort mean at T1) on measures of cognitive function (below). Alcohol ascertainment: Current: self-report questionnaire asking “how many days they drank alcoholic beverages, on average, in the last year” and number of drinks consumed on drinking days. Lifetime: no information. Cognitive function: Global cognitive function based on the sum of scores on two tests, one of mental status (0-10 points: e.g. questions ‘who is the president’, ‘what is used to cut paper’; ‘what is desert plant’; ‘what is the day, date, month, year’; counting backward from 20 and 86) and one of immediate memory (0-10 points: 10 item list of concrete nouns). Function was dichotomised as low (score of 9.5-13) or high (score of 14-20).	Study period: 1994-2000 Alcohol exposure: two assessments, ~2 years apart (T0, T1: 1994, 1997-1998) Outcome measures: 2 assessments, baseline and ~ 2 years later. (T1, T2: 1997-1998, 2000) Length of outcome follow-up: ~2 years from baseline (T1)
Piumatti 2018 [54] United Kingdom Cohort name: UK Biobank prospective cohort Serious risk of bias	Based on 13,342 men and women (54.7% female) aged 40-73 years at point of first alcohol measure (T0). Substudy of the UK Biobank cohort involving those who had undergone a repeat assessment.	Alcohol consumption was treated as a continuous variable in analyses (mean grams of alcohol per day), so categories were not defined. The analysis was limited to ‘weekly drinkers’: those who consumed alcohol at least once per week.	Observational cohort examining associations between different levels of alcohol consumption and change in cognitive function in middle and older populations. Inclusion criteria: Eligible participants were aged 40-73 years (T0), from a population sample from those registered for the UK National Health Service and living within 40 km of a Biobank research centre. Exclusion criteria: Consumed alcohol less frequently than once a week, self-disclosed history of neurological disorder (e.g. stroke, head trauma), only one valid score (from 7 tests) at baseline or follow-up. Alcohol ascertainment: Current: self-report questionnaire asking about frequency of consumption and number of drinks consumed on average per week (by alcohol type). Recall: not reported. Lifetime: not measured. Cognitive function: Specific cognitive domains (2 outcomes). Complex attention (processing speed based on a ‘stop-go’ reaction time task). Results reported for reaction time (mean of completed test trials) and intra-individual variation (IIV; standard deviation of each participant’s reaction time over 7 trials). Lower scores = better cognition.	Study period: 2006-2015 Alcohol exposure: single assessment (T0: 2006-2010; second assessment made at follow-up, but not used in prospective analysis) Outcome measures: baseline and follow-up assessments at ~ 5 year intervals. (T0-T1: 2006-2010; 2011-2015) Length of outcome follow-up: ~ 5 years from baseline (T0; mean 4.31)
Richard 2017^†^ [55] United States Cohort name: The Rancho Bernardo Study Serious risk of bias	Based on 1334 men and women (54% female) aged 55-84 years at point of first alcohol measure (T0). Substudy of the Rancho Bernardo Study cohort which was established to examine heart disease risk factors.	Non-drinker (referent): ‘no past alcohol use’ or 'did not drink in last year' Moderate: ≤ 1 drink/day for men 65 and older and women; ≤ 2 drinks/day for men (midpoint = 6 g/day for women; midpoint = 12 g/day for men) Heavy: > 1–3 drinks/day for men age 65 and older and women; > 2–4 for men under 65 (midpoint = 24 g/day for women; midpoint = 36 g/day for men) Excessive: > 3 drinks/day for men age 65 and older and women; > 4 drinks/day for men under 65 (midpoint = 48 g/day for women; midpoint = 60 g/day for men) Grams per drink: 12 g; NIAAA guidelines	Observational cohort examining association between different levels and patterns (by frequency) of alcohol consumption and cognitively healthy longevity (survival to age 85). Inclusion criteria: Eligible participants were those with potential to reach 85 years during follow-up period (55-84 years at T0). Exclusion criteria: Those who did not have ‘intact cognitive function’ at any assessment prior to 85^th^ birthday (or had not had an assessment 2 years prior to birthday). Missing data on education status. Missing data on education status. Alcohol ascertainment: Current: self-report questionnaire asking about frequency of consumption and number of drinks (by alcohol type) in a typical week. Lifetime: asked about any ‘past alcohol use’. Cognitive function: Global cognitive function (MMSE). Raw scores converted to Z-scores (adjusted for sex, age, education) using normative data. Cognitive impairment: Z-scores below −1.5. Outcomes reported: Cognitively Healthy Longevity (CHL: survival to age 85 without cognitive impairment), Cognitively Impaired Longevity (CIL: survival to age 85 with cognitive impairment).	Study period: 1984-2009 Alcohol exposure: single assessment at baseline (T0: 1984-1987) Outcome measures: up to 6 assessments at ~ 4 year intervals. (T1-T6: 1988-2009) Length of outcome follow-up: median of 13.9 years from baseline (T0)
Sabia 2011^†^ [56] France Cohort name: GAZEL cohort study Serious risk of bias	Based on 4073 men aged ~45-55 years at point of first alcohol measure (T0) and 55-65 years at point of cognition measure (T10). Substudy of GAZEL cohort study which was established to examine disease and health-related factors among workers in France’s national electricity and gas company.	No-consumption: 0 drinks per week Occasional: 1–3 drinks per week (midpoint = 3 g/day) Light (referent): 4–14 drinks per week (midpoint = 14 g/day) Moderate: 15–21 drinks per week (midpoint = 28 g/day) Heavy: > 21 drinks per week (midpoint = 38 g/day) Grams per drink: reported as 10–12 g (11 g assumed in SR analyses)	Observational cohort examining association between average level of alcohol consumption (measured over 10 years) and cognitive function at age ≥ 55 years. Also examines the effect of the trajectory of consumption (decreasing, stable, or increasing over 10 years) on cognition. Inclusion criteria: Eligible participants were men aged ≥ 55 years at the time cognition was measured (T10) and working for the electricity and gas company in which the GAZEL cohort was based. Exclusion criteria: Women (due to small number in the GAZEL cohort: ~10%); had no measure of alcohol consumption from T0-T4, T5-T9, or both; did not have full covariate data; did not participate in cognitive tests (*n* = 4525, 48.2%). Alcohol ascertainment: Current: self-report questionnaire asking about frequency of consumption and number of drinks per day (by alcohol type) in last 7 days. Calculated mean consumption per week over 10 years using annual measures of consumption (T0-T9). Lifetime: no information. Cognitive function: Specific cognition domain - complex attention measured by the Digit Symbol Substitution Test (DSST; subtest of the Weschler Adult Intelligence Scale). Mean scores reported for number of correct responses on 93 items (score range 0-93; higher score=better cognition).	Study period: 1992-2004 Alcohol exposure: 10 assessments at ~ 1 year intervals (T0-T9: 1992-2001, or 1993-2002, or 1994-2003; period determined by year of cognitive testing) Outcome measures: single assessment (T10: 2002, or 2003, or 2004) Length of outcome follow-up: 12 months from baseline (T9)
Sabia 2014 [57] England Cohort name: Whitehall II cohort study Serious risk of bias	Based on 7153 men and women (29 % female) aged 35-55 years at point of first alcohol measure (T0) and 55-80 years at final cognition measure. Analysis from the Whitehall II cohort which was established to examine social determinants of health among British civil servants.	Alcohol abstainers in the last 10 years: 0 grams in last 12 months (T1, T2, and T3) Alcohol cessation in the last 10 years (quitters): 0 g in last 12 months (T2), > 0 grams at T0 or T1 Occasional drinkers: > 0 g in last 12 months, none in the last week (T0, T1 and T2) 0 to 70th percentile (referent): 0.1–9.9 g/day for women (median = 3.4 g/day); 0.1–19.9 g/day for men (median = 8.4 g/day) 70th to 90th percentile: 10–18.9 g/day for women (median = 13.3 g/day); 20–35.9 g/day for men (median = 26.3 g/day) > 90th percentile: 19–66 g/day for women (median = 23.8 g/day); 36–112 g/day for men (median = 46.9 g/day)	Observational cohort examining association between average level of alcohol consumption in midlife (mean age 44 years) and subsequent cognitive decline. Inclusion criteria: Eligible participants were British public servants age 35–55 years at cohort inception (T0). Exclusion criteria: Missing alcohol or covariate data. Did not participate in the any of the baseline or follow-up assessments of cognition. Alcohol ascertainment: Current: self-report questionnaire asking about frequency of consumption (last 12 months) and number of drinks (by alcohol type) in last 7 days. Calculated mean consumption over 10 years from data collected at T0, T1 and T2. Lifetime: no information. Cognitive function: Global cognitive function (average of scores on 4 tests, each standardised using the mean and SD of scores at T2). Tests were of executive function (Alice Heim 4-I timed test of inductive reasoning; recall of “S” words; recall of animal names), learning and memory (recall of 20 words). Higher GCF score = less cognitive decline (over 10 years). Other outcomes reported: executive function; learning and memory	Study period: 1985-2009 Alcohol exposure: multiple assessments; baseline and then 2 assessments at ~5 year intervals (T0-T2: 1985-88, 1991-93, 1997-99) Outcome measures: 3 assessments at ~5 year intervals (T2-T4: 1997-99, 2002-04, 2007-09) Length of outcome follow-up: ~ 10 years from baseline (T2)
Samieri 2013a [58] United States Women’s Health Study Serious risk of bias	Based on 6174 women aged ≥ 60 at point of first alcohol measure (T0). Observational substudy of Women’s Health Study randomised trial of aspirin and vitamin E for prevention of CVD and cancer.	Non-drinker (referent): 0–1 drinks per day (median = 0 g/day) Category: ≥ 1 to ≤ 14.9 g/day (median = 2.9 g/day; range 1.2–6.0) Category: ≥ 15 g/day (median = 25.4 g/day; range 16.8–37.8)	Observational cohort examining association between a Mediterranean diet and specific components (including different levels of alcohol consumption) and cognitive function over time. Inclusion criteria: Eligible participants were those aged ≥65 years at cognitive assessment (T1; ~60 at T0), Exclusion criteria: complete dietary data (‘complete’ was not defined). Alcohol ascertainment: Current: self-report food frequency questionnaire asking about frequency of consumption of foods and beverages, including alcohol, and portion size (no information reported). Recall period: last 12 months. Lifetime: no information. Cognitive function: Global cognitive function average of z-scores from 5 tests: Telephone Interview for Cognitive Status (overall, including delay recall of 10-word list), East Boston Memory Tests (immediate and delayed recall), category fluency test. Other outcomes reported: Learning and memory (average of z-scores on the 4 tests). Higher mean scores = better cognition (inferred, not reported). No information on SD for average of Z scores, so scores are difficult to interpret.	Study period: 1992-2004 Alcohol exposure: single assessment (T0: 1992-1995) Outcome measures: 3 assessments, first at T1 (average of 5.6 years from T0) and then at ~2 year intervals (T1-T3: 1998-2004) Length of outcome follow-up: ~10 years from T0.
Solfrizzi 2007 [59] Italy Cohort name: Italian Longitudinal Study on Aging (ILSA) Serious risk of bias	Based on 1445 men and women (44% female) aged 65 to 84 years at point of first alcohol measure (T0). Substudy of ILSA which aims to examine common chronic conditions in the older population, and identify risk and protective factors	None: zero in last 5 years (current abstainer; referent) (former= zero in last 5 years, but some over lifetime) Category: < 1 drink per day (midpoint = 7.5 g/day) Category: 1–2 drinks per day (midpoint = 22.5 g/day) Category: > 2 drinks per day (midpoint = 37.5 g/day) Grams per drink: 15 g of alcohol	Observational cohort examining association between different levels of alcohol consumption and incidence of mild cognitive impairment (also progression to dementia). Inclusion criteria: Eligible participants were 65-84 years at baseline (T0), independent or institutionalised. Exclusion criteria: Confirmed diagnosis of dementia at T0 (structured clinical assessment for all participants with score on MMSE <24), refusal to perform MMSE or other neuropsychological test, unknown level of education. Alcohol ascertainment: Current: self-report food frequency questionnaire asking about frequency of consumption (number of times per day/month/year) and number of drinks per day (by alcohol type; 3 portion sizes). Recall: last 12 months. Lifetime: asked ‘when they had begun to drink’ and ‘how much beer or wine per day ever since’ (to identify former drinkers, and changed patterns). Cognitive function: Incidence of mild cognitive impairment diagnosed by trained neurologist using diagnostic criteria based on Petersen [64] (did not require subjective memory impairment; allowed for neurocognitive disabilities and comorbidities). Other outcomes: progression from MCI to dementia.	Study period: 1992-1996 Alcohol exposure: single assessment at baseline (T0: 1992) Outcome measures: 2 assessments, baseline and then ~3.5 years later (T0, T1: 1995-1996) Length of outcome follow-up: 3.5 years from baseline alcohol measurement (T0).
Stott 2008 [60] United Kingdom, Netherlands Prospective Study of Pravastatin in the Elderly at Risk (Prosper) Serious risk of bias	Based on 5804 men and women (52 % female) aged 70-82 years at point of first alcohol measure (T0) and ~73–85 years at final cognition measure. Observational study using data collected from the PROSPER randomised trial of Pravastatin.	Non-drinker (referent): not defined. Assumed 0 to < 1 unit/week for men and women (midpoint = 0.6 g/day; ) Low intake: ≥ 1 to ≤ 3 units/week for women; ≥ 1 to ≤ 7 units/week for men (midpoint = 2.3 g/day for women; midpoint = 4.6 g/day for men) Moderate intake: > 3 units/week for women ; > 7 units/week for men (midpoint = 4.6 g/day for women; midpoint = 11.4 g/day for men) Grams per drink: not reported (assumed 8 g based on UK standard, but study includes participants from Netherlands where 10 g is a standard drink)	Observational cohort examining association between different levels of alcohol consumption and cognitive function over time. Inclusion criteria: Eligible participants were those aged 70-82 years (T0), with good cognitive function (see exclusion) and evidence of vascular disease or major vascular risk factors (hypertension, smoking, diabetes). Exclusion criteria: MMSE 24 or below at T0. Alcohol or drug abuse. Alcohol ascertainment: Current: very little information reported about the measurement of alcohol here or in the trial protocol or report except “alcohol intake was … quantified in terms of usual alcohol intake in units per week for the previous month”. Lifetime: no information; assume not collected. Cognitive function: Global cognitive function (MMSE; higher scores means better cognition). Mean scores are reported for MMSE and other measures (below). Other outcomes reported: complex attention (Stroop Color–Word test; Letter Digit Coding test); learning and memory (immediate and delayed recall on Picture-Word Recall test).	Study period: Dec 1997- Mar 2002 Alcohol exposure: single assessment at baseline (T0: Dec 1997 to ~May 1999) Outcome measures: 5 assessments, first at baseline then at ~1 year intervals (T0-T4: years not reported) Length of outcome follow-up: mean 3.2 years from baseline (T0).
Wardzala 2018 [61] United States Cohort name: Oregon Brain Aging Study (OBAS); Intelligent Systems for Assessing Aging Changes (ISAAC) study Serious risk of bias	Based on 486 men and women (75% female) aged ~80 years or above at point of first alcohol measure (T0). Substudy involving participants from OBAS and ISAAC cohorts that met eligibility criteria for the current study.	Rare/never-drinker (referent): zero drinks per week (for any period ≥ 3 month over lifetime) Moderate: for women: < 3 drinks/day and < 7 drinks per week (mean = 8 g/day); for men: < 4 drinks/day and < 14 drinks/week (mean = 9 g/day) Heavy: for women: ≥ 3 drinks/day and ≥ 7 drinks per week (mean = 27 g/day); for men: ≥ 4 drinks/day and ≥ 14 drinks/week (mean = 24 g/day) Categories based on NIAAA guidelines. Grams per drink: not reported (assumed 12 g based on NIAAA guideline s[63])	Observational cohort examining association between different levels of alcohol consumption and cognitive function among people ~80 years and over. Inclusion criteria: Eligible participants were aged ≥80 years at T0 (≥70 years for non-Caucasian, who comprised <10-20% of participants), living independently in the community with better than average health for age. Exclusion criteria: Cognitively impaired (Clinical Dementia Rating (CDR) of > 0.5 and a Mini-Mental State Examination (MMSE) score of ≤24). No alcohol data; missing outcome data. Alcohol ascertainment: Current: self-report questionnaire in interview asking about “frequency of drinking (days per week) and drinks per drinking day". Lifetime: asked if "ever consumed > 1 drink per week for > 3 months". Asked about drinking (same quantity/ frequency questions) at age ’40-current’, ’19-39’ and ‘0-18’ years. Cognitive function: Global cognitive function MMSE score. Specific cognitive domains: learning and memory (word list: delayed recall), executive function (Trail making test B), complex attention (Digit Symbol-Coding), language (Semantic fluency - animals). Results reported as change in mean score over time (smaller change = better outcome).	Study period: 2004 to ~2011 (OBAS); 2007 to ~2017 (ISAAC) Alcohol exposure: single assessments for most participants at baseline (T0: ~2004 OBAS; 2007-09 ISAAC) Outcome measures: not reported, ~6-7 annual assessments based on time in study (mean 6-8 years). (No information on time points. Assume-T0-T7: 2004 to ~2011 (OBAS); 2007 to ~2017 (ISAAC) Length of outcome follow-up: no information. ~5–7 years from alcohol measurement (T0)

*Content is replicated for studies that examined levels and patterns, except details of alcohol categories/ascertainment. ^†^Denotes a study that also contributed data on patterns of alcohol consumption. ^††^National Institute on Alcohol Abuse and Alcoholism (NIAAA), United States [63]

Six of the 18 studies were conducted in the United States (Downer 2015, Lang 2007 [also UK], McGuire 2007, Richard 2017, Samieri 2013, Wardzala 2018), four were in the United Kingdom (Lang 2007, Piumatti 2018, Sabia 2014, Stott 2008), and two each in Sweden (Hassing 2018, Hogenkamp 2014) and France (Kesse-Guyot 2012, Sabia 2011). Other studies were in Australia (Heffernan 2016), Eastern Europe (Horvat 2015), Japan (Kitamura 2017) and Norway (Arntzen 2010).

##### Ascertainment of alcohol exposure

The first point at which alcohol consumption was measured was at mid-life in seven studies (Arntzen 2010, Downer 2015, Hassing 2018, Horvat 2015, Kesse-Guyot 2012, Sabia 2011, Sabia 2014), late-life in eight studies (Heffernan 2016, Hogenkamp 2014, Lang 2007, McGuire 2007, Samieri 2013, Solfrizzi 2007, Stott 2008, Wardzala 2018) and spanned from mid-life (~age 40 to 60) to late-life (~age 65 to > 80) in three studies (Kitamura 2017, Piumatti 2018, Richard 2017).

Only three studies measured alcohol at multiple time points. McGuire 2007 measured alcohol twice, 2 years apart (McGuire 2007). In Sabia 2011 and Sabia 2014, multiple measures of alcohol consumption were taken over 10 years; ten annual measures were taken in Sabia 2011 (a minimum of 1 measure in each 5-year period was required) and in Sabia 2014, three measures were taken at 5-year intervals. Details of the measurement methods and how these were used to categorise consumption are reported in Table 4.

##### Measurement of cognition outcomes

Baseline measures of cognition were taken in eight of 18 studies (Heffernan 2016, Hogenkamp 2014 Horvat 2015, McGuire 2007, Piumatti 2018, Solfrizzi 2007, Stott 2008 and Wardzala 2018). Multiple follow-up measures of cognition were taken in eight studies (Downer 2015, Hassing 2018, Heffernan 2016, Sabia 2011, Sabia 2014, Samieri 2013a, Stott 2008, Wardzala 2018). Richard 2017 took multiple measures of cognition, but only to exclude those with cognitive impairment prior to age 85.

One of 18 studies reported a diagnosis of mild cognitive impairment, based on clinical exam and validated diagnostic criteria (Solfrizzi 2007). Eleven of 18 studies reported a measure of global cognitive function. Of these, six reported outcomes based on the MMSE (Downer 2015, Hassing 2018, Kitamura 2017, Richard 2017, Stott 2008, Wardzala 2018; see Table 3 and Table 4 for the metrics derived from the MMSE), and five reported composite measures of global cognitive function derived for tests of one or more specific cognitive domains (Kesse-Guyot 2012, Lang 2007, McGuire 2007, Sabia 2014, Samieri 2013). Six studies reported measures of function on specific cognitive domains, most reporting results for multiple domains from a battery of neurocognitive tests. The results selected for review from these studies were measures of learning and memory in three studies (Arntzen 2010, Heffernan 2016, Horvat 2015), executive function in one study (Hogenkamp 2014) and complex attention in two studies (Piumatti 2018, Sabia 2011).

##### Studies examining the effects of different patterns of alcohol consumption

Characteristics of the 12 included studies that examined the effects of different patterns of alcohol consumption are summarised in the Additional file 2, Appendix 4, Table 4.1. Six of these studies were among adolescents or university students, while the other six involved participants at mid- to late-life. The studies varied considerably in terms of the types of patterns considered. Three of 12 examined heavy drinking episodes (“binge” drinking), six examined changes in the pattern of consumption over time (levels and frequency) of which two focused on changes in binge drinking patterns, one examined the age of onset of first and weekly drinking, and two examined frequency of consumption only. Importantly, the analysis methods used in these studies have not been carefully reviewed, so it is possible that some studies may not meet the eligibility criterion for using only prospective measures of alcohol in the analysis.

#### Ongoing studies and studies awaiting assessment

We did not identify any ongoing studies, although many of the identified cohorts are ongoing, so may generate analyses eligible for updates of this review. There are no studies awaiting assessment.

#### Excluded studies

Reasons for excluding the 195 studies are described in the Additional file 2, Appendix 5 (Characteristics of excluded studies). An alphabetically sorted reference list of all studies excluded after full-text review is provided in the Additional file 2, Appendix 10.

Of the 195 studies, eight were coded as “near miss” because they met all eligibility criteria but measures of alcohol were collected concomitantly with measures of cognition and the authors modelled the association between alcohol consumption and cognition over time (Additional file 2, Appendix 5, Table 5.1). In many cases, this was done to provide a more reliable measure of alcohol intake over time; however, the approach rendered the studies ineligible because the analysis was not limited to prospective measures of alcohol, and hence do not enable causal inferences to be made about the effect of alcohol on cognition. For this dataset, it would have been possible for the study authors to have examined the association between alcohol consumption at a fixed time and future cognition.

A further 19 studies were excluded to narrow the scope of the review to a priority question that could be addressed within the required timeframe and resources. Since a recent systematic (Xu 2017) examined the effects of different levels of alcohol on dementia and presented a dose-response analysis, we excluded 15 studies for which the only eligible outcome was dementia or major cognitive impairment (Additional file 2, Appendix 5, Table 5.2). In addition, we excluded studies that examined the effects of alcohol among specific subgroups (two studies: alcohol use disorder or diabetes) or that only examined the effects of high levels of alcohol intake (Additional file 2, Appendix 5, Table 5.3).

The remaining 176 excluded studies were excluded based on one or more of the pre-specified eligibility criteria, as reported in Tables 5.4-5.12 of the Additional file 2 (Appendix 5).

### Risk of bias

The complete risk of bias assessment for each study, including the rationale for the judgement of each domain, is reported in the Additional file 2, Appendix 6 (Risk of bias assessment of included studies). Study methods that influenced each judgement are also summarised. The overall judgement is noted in Table 4 (Study characteristics).

All studies were assessed as being at serious risk of bias, except for two (Hassing 2018, McGuire 2007), which were judged to be at critical risk of bias. In addition to concerns identified across all studies about selection bias and bias arising from misclassification of alcohol consumption, these two studies were judged to be at a critical risk of bias due to missing outcome data. Neither study reported whether missing data were balanced across groups, nor did the analysis approach address potential biases arising from missing data.

Across all studies, there were serious concerns about the risk of selection bias. Most studies enrolled participants at mid-life (~40 to 60 years of age) or late-life (~65 to 80 years). The lag time between initiating drinking and the first measurement of alcohol intake means that those who previously experienced harmful outcomes associated with drinking may be excluded (because they died or were inaccessible, declined or were unable to participate). Further, some studies excluded less healthy people (e.g. those with pre-existing cognitive impairment). While difficult to avoid, these design features are likely to result in the exclusion of drinkers with poorer health caused or exacerbated by alcohol (including those with alcohol-related cognitive impairment or alcohol-related risk factors for impairment). This risks biasing the sample through the inclusion of healthy drinkers, potentially attenuating differences between drinking and non-drinking groups.

There were also serious concerns about the risk of bias arising from methods used to categorise participants’ alcohol consumption and the resulting potential for misclassification. All but three studies (Sabia 2011, Sabia 2014, McGuire 2007) used a single assessment of alcohol consumption to estimate consumption, so most studies are unlikely to capture drinking patterns over time. Related to this, almost all studies categorised alcohol intake based on current consumption (recall over the last 12 months or less), so contamination of non-drinking groups with former drinkers is likely. To account for this, some studies used a low- or moderate-level drinking group as the referent, and two studies included 10-year abstainers only (Sabia 2011, Sabia 2014). However, the problems with measurement of lifetime consumption, together with underestimation (through poor recall) or conscious under-reporting of intake, mean that misclassification is likely across most included studies.

Since former drinkers have been shown to have poorer self-reported health and higher levels of depression than current drinkers (both associated with cognition), misclassification has implications for the comparability of groups and confounding [20, 21]. Most studies adjusted for important confounding domains pre-specified for the review, but some residual confounding was likely.

No important conflicts of interest were identified for authors of any of the 18 included studies (Additional file 2, Appendix 4, Table 4.2). One study (Kesse-Guyot 2012) received partial funding from a food catering company, in addition to government and non-food industry funding (the proportion of funding from each source was not reported). The authors reported that the funders had no involvement in the study; however, a conflict of interest could not be completely ruled out. Of the 17 remaining studies, 14 appeared free of any conflict of interest (funding or other), and three appeared free of financial conflicts but provided insufficient information to judge other conflicts. Ethics approval was reported for 14 of 18 studies (Additional file 2, Appendix 4, Table 4.2).

### Effects of different levels of alcohol on cognition

#### Dose-response syntheses

In the following sections (‘Females’, ‘Males’, and, ‘Females and males’), the results from dose-response analyses are presented. For most studies, assumptions were required to calculate the doses of alcohol and the statistics used to compute the standardised mean differences (SMDs) (see Additional file 2, Appendix 7 for details). Therefore, while the estimated dose-response relationships may be indicative of the shape of the relationship, the presented estimates should be cautiously interpreted.

##### Females

**Fig. 2:**
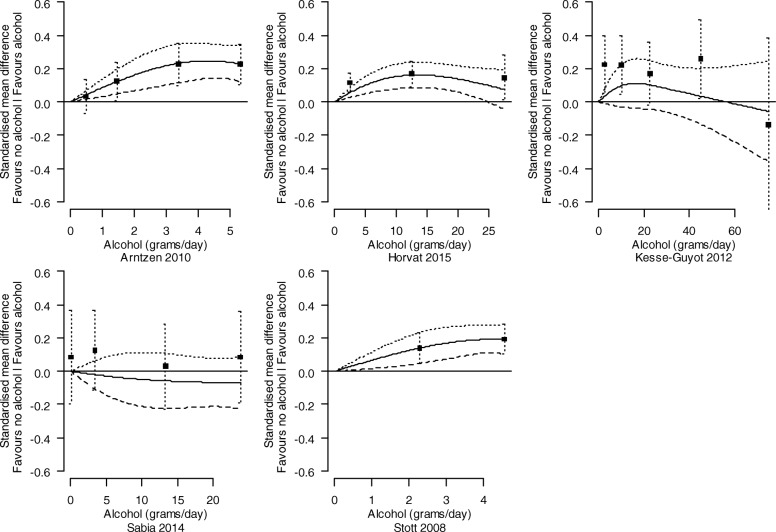
Study-specific standardised mean differences (SMDs) of cognition for increasing doses of alcohol (grams/day) for females. The relationship between the SMDs and cognition was modelled using a restricted cubic spline with three knots (located at 10th, 50th, and 90th percentiles of alcohol consumption observed across the studies). Black squares indicate a standardised mean difference and the whiskers indicate its 95% confidence interval. Solid lines represent the estimated dose-response curves, and the dashed lines the corresponding 95% confidence intervals. The current non-drinker served as the referent group

Five of 15 eligible studies for this analysis were able to be included in the investigation of the dose-response relationship between levels of alcohol consumption and cognition. Study-specific dose-response curves of standardised mean differences (SMDs) of cognition (compared with current non-drinkers) and alcohol consumption (grams/day) are displayed in Fig. 2. Three of the five studies reported measures of global cognitive function, derived by averaging standardised scores on tests of specific cognitive domains (Kesse-Guyot 2012; Sabia 2014), or from an MMSE score (Stott 2008). The other two studies reported measures of learning and memory (Arntzen 2010; Horvat 2015).

**Fig. 3:**
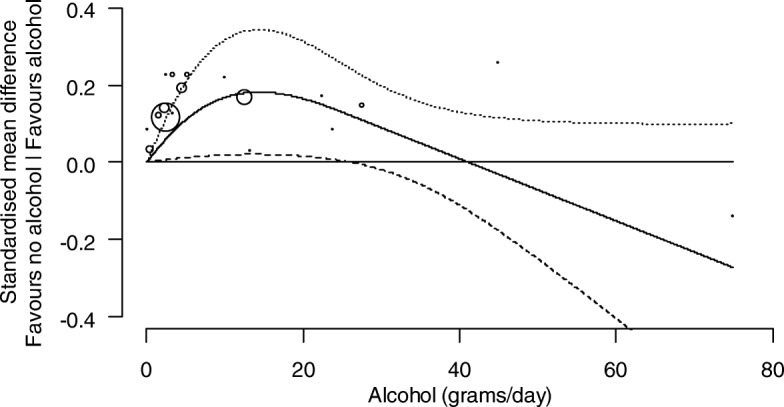
Pooled dose-response relationship between alcohol consumption (grams/day) and the standardised mean difference in cognition (solid line) for females. The study-specific relationships were modelled using restricted cubic splines and combined in a multivariate random-effects meta-analysis. The dashed lines represent the 95% confidence intervals for the combined spline model. The current non-drinker served as the referent group. Circles indicate study-specific observed SMDs, with the size of the bubbles proportional to precision (inverse of the variance) of the SMDs

The pooled dose-response relationship is displayed in Fig. 3 and tabulated in Table 5. For alcohol consumption less than 25.9 g alcohol/day (the point at which the predicted lower bound of the confidence interval crosses zero), cognition was slightly better in those consuming alcohol than current non-drinkers. However, the SMDs were small, with a maximum SMD of 0.18 (95%CI 0.02, 0.34), occurring at an intake of 14.4 g alcohol/day. Further, there was evidence of heterogeneity in the study-specific dose-response coefficients (*I*^*2*^ = 69.5%, *Q* test for heterogeneity *p* value = 0.001).

**Table 5:** Predicted SMDs from pooled dose-response relationships for varying levels of alcohol consumption (grams alcohol/day)

Alcohol consumption (grams/day)	Females only		Males only		Females and males	
	SMD	(95%CI)	SMD	(95%CI)	SMD	(95%CI)
5	0.11	(0.01, 0.21)	0.02	(0, 0.04)	0.08	(0, 0.15)
10	0.17	(0.02, 0.32)	0.04	(0.01, 0.08)	0.14	(0, 0.29)
15	0.18	(0.02, 0.34)	0.05	(0, 0.1)	0.2	(−0.01, 0.4)
20	0.16	(0.02, 0.31)	0.05	(0, 0.1)	0.23	(−0.01, 0.48)
25	0.13	(0, 0.26)	0.05	(−0.01, 0.11)	0.24	(−0.03, 0.51)
30	0.09	(−0.02, 0.2)	0.04	(−0.02, 0.1)	0.23	(−0.05, 0.51)
35			0.03	(−0.04, 0.1)	0.21	(−0.07, 0.49)
40			0.01	(−0.06, 0.09)	0.17	(−0.1, 0.45)
45			0	(−0.09, 0.09)	0.13	(−0.14, 0.4)
50			−0.02	(−0.13, 0.09)	0.08	(−0.2, 0.35)
55			−0.04	(−0.16, 0.09)	0.03	(−0.25, 0.31)

Results from the sensitivity analyses revealed that the shape of the dose-response model was not robust to different locations of the knots for higher levels of alcohol consumption (Additional file 2, Appendix 8, Figure 8.1). This was perhaps unsurprising since only one study (Kesse-Guyot 2012) contributed data for high levels of alcohol consumption. A further sensitivity analysis removing two SMDs associated with alcohol consumption greater than 30 g alcohol/day from Kesse-Guyot showed that the dose-response relationship at lower alcohol consumption levels was robust to the outlying observations (Additional file 2, Appendix 8, Figure 8.2).

##### Males

**Fig. 4:**
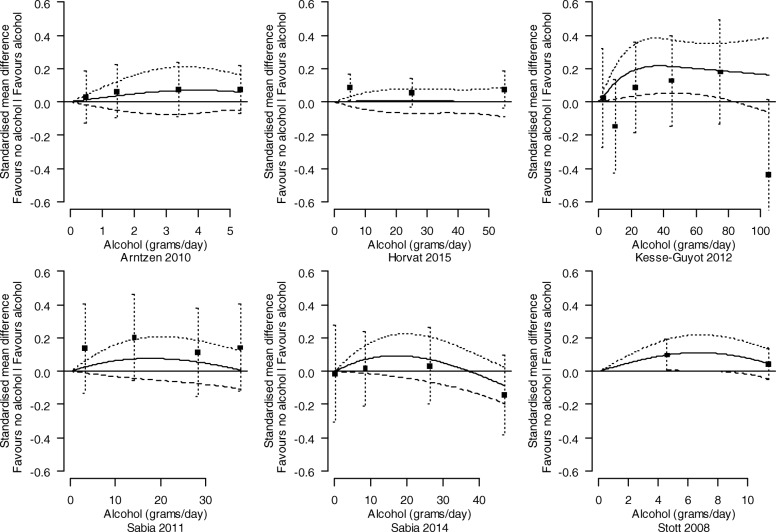
Study-specific standardised mean differences (SMDs) of cognition for increasing doses of alcohol (grams/day) for males. The relationship between the SMDs and cognition was modelled using a restricted cubic spline with three knots (located at 10th, 50th, and 90th percentiles of alcohol consumption observed across the studies). Black squares indicate a standardised mean difference and the whiskers indicate its 95% confidence interval. Solid lines represent the estimated dose-response curves, and the dashed lines the corresponding 95% confidence intervals. The current non-drinker served as the referent group

Six of 14 eligible studies for this analysis were able to be included in the investigation of the dose-response relationship between levels of alcohol consumption and cognition. Study-specific dose-response curves of standardised mean differences (SMDs) of cognition (compared with current non-drinkers) and alcohol consumption (grams/day) are displayed in Fig. 4. Three of the six studies reported measures of global cognitive function, derived by averaging standardised scores on tests of specific cognitive domains (Kesse-Guyot 2012; Sabia 2014), or from an MMSE score (Stott 2008). The other three studies reported measures of a specific cognitive domain; learning and memory (Arntzen 2010; Horvat 2015) or complex attention (Sabia 2011).

**Fig. 5:**
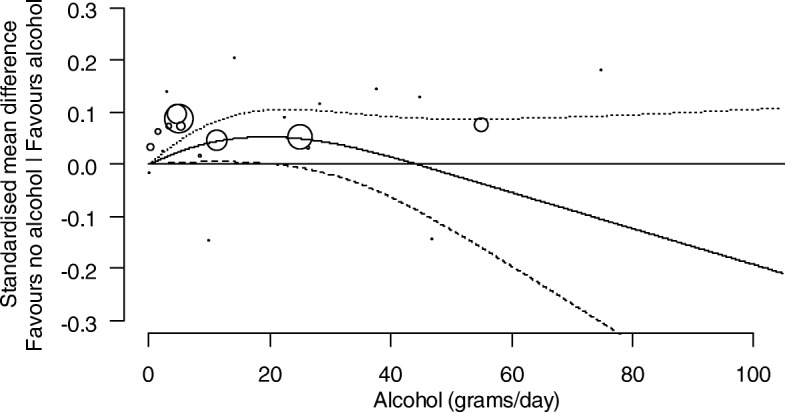
Pooled dose-response relationship between alcohol consumption (grams/day) and the standardised mean difference in cognition (solid line) for males. The study-specific relationships were modelled using restricted cubic splines and combined in a multivariate random-effects meta-analysis. The dashed lines represent the 95% confidence intervals for the combined spline model. The current non-drinker served as the referent group. Circles indicate study-specific observed SMDs, with the size of the bubbles proportional to precision (inverse of the variance) of the SMDs

The pooled dose-response relationship is displayed in Fig. 5 and tabulated in Table 5. The shape of the dose-response relationship for males was similar to that observed for females; however, the maximum SMD of 0.05 (95%CI 0.00, 0.10), occurring at an intake of 19.4 g alcohol/day, was very small. For all levels of alcohol consumption, the predicted lower bound of the confidence interval of the SMD indicated that cognition was similar or poorer as compared to current non-drinkers, but the SMDs were small for alcohol intakes less than 55 g/day (Table 5). There was evidence of heterogeneity in the study-specific dose-response coefficients (*I*^2^ = 56.6%, *Q* test for heterogeneity *p* value = 0.011).

Results from the sensitivity analyses revealed that the shape of the dose-response model was not robust to different locations of the knots for higher levels of alcohol consumption (Additional file 2, Appendix 8, Figure 8.3). This was perhaps unsurprising since only one study (Kesse-Guyot 2012) contributed data for high levels of alcohol consumption. A further sensitivity analysis removing two SMDs associated with alcohol consumption greater than 70 g alcohol/day from Kesse-Guyot showed that the dose-response relationship at lower alcohol consumption levels was robust to the outlying observations (Additional file 2, Appendix 8, Figure 8.4).

##### Females and males

**Fig. 6:**
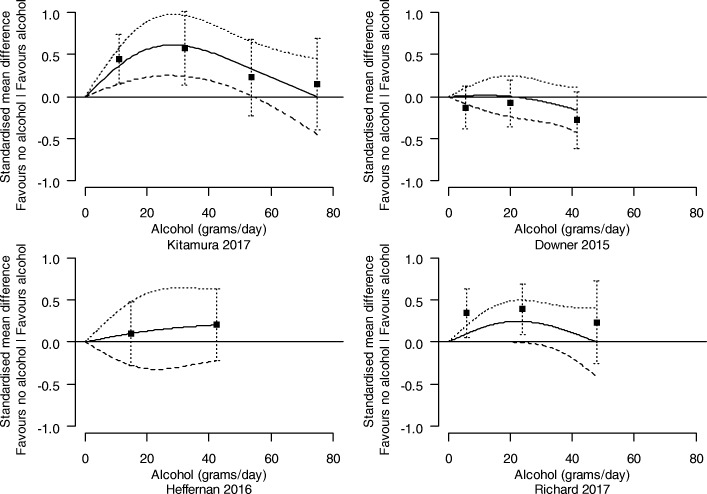
Study-specific standardised mean differences (SMDs) of cognition for increasing doses of alcohol (grams/day) for females and males. The relationship between the SMDs and cognition was modelled using a restricted cubic spline with three knots (located at 10th, 50th, and 90th percentiles of alcohol consumption observed across the studies). Black squares indicate a standardised mean difference and the whiskers indicate its 95% confidence interval. Solid lines represent the estimated dose-response curves, and the dashed lines the corresponding 95% confidence intervals. The current non-drinker served as the referent group

Four of 16 eligible studies for this analysis were able to be included in the investigation of the dose-response relationship between levels of alcohol consumption and cognition. Study-specific dose-response curves of standardised mean differences (SMDs) of cognition (compared with current non-drinkers) and alcohol consumption (grams/day) are displayed in Fig. 6. Three of the four studies reported measures of global cognitive function, derived by averaging standardised scores on tests of specific cognitive domains (Downer 2015), or from an MMSE score (Kitamura 2017; Richard 2017). The other study reported a measure of a specific cognitive domain, learning and memory (Heffernan 2016).

**Fig. 7:**
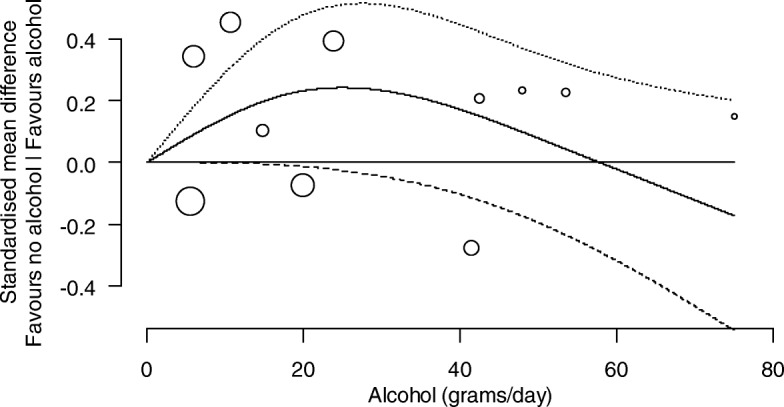
Pooled dose-response relationship between alcohol consumption (grams/day) and the standardised mean difference in cognition (solid line) for females and males. The study-specific relationships were modelled using restricted cubic splines and combined in a multivariate random-effects meta-analysis. The dashed lines represent the 95% confidence intervals for the combined spline model. The current non-drinker served as the referent group. Circles indicate study-specific observed SMDs, with the size of the bubbles proportional to precision (inverse of the variance) of the SMDs.

The pooled dose-response relationship is displayed in Fig. 7 and tabulated in Table 5. The shape of the dose-response relationships for females only and males only was similar to the dose-response shape for females and males. The maximum SMD of 0.24 (95%CI −0.03, 0.51) occurred at an intake of 25 g alcohol/day. For higher levels of alcohol consumption (e.g. > 55 g alcohol/day), there may be detrimental effects on cognition; however, this is where there is most uncertainty in the predictions (see sensitivity analyses). There was some evidence of heterogeneity in the study-specific dose-response coefficients (*I*^2^ = 47.2%, *Q* test for heterogeneity *p* value = 0.078).

Results from the sensitivity analyses revealed that the shape of the dose-response model was not robust to different locations of the knots for higher levels of alcohol consumption (Additional file 2, Appendix 8, Figure 8.5). This is likely due to only one study (Kitamura 2017) contributing data for high levels of alcohol consumption. A further sensitivity analysis removing one SMD associated with alcohol consumption greater than 55 g alcohol/day from Kitamura showed that the dose-response relationship at lower alcohol consumption levels was robust to the outlying observation (Additional file 2, Appendix 8, Figure 8.6).

#### Summary of results from single studies

Six studies (Solfrizzi 2007, Lang 2007a, Hogenkamp 2014, Samieri 2013a, Piumatti 2018, Wardzala 2018) that examined the association between levels of alcohol consumption and cognition were not able to be included in the dose-response analyses (see Additional file 2, Appendix 9 for reasons for exclusion). Study characteristics, reported associations, and interpretations are presented in Table 6. The results are briefly summarised here. The study authors’ interpretations seemed often to be based on statistical significance. In combination, results were often incompletely reported (e.g. missing effect estimates, no information about the range of a scale) precluding clinical interpretation of the observed associations.

**Table 6:** Results of single studies that examined effects of different levels of alcohol (ineligible for dose-response analysis)

Study details	Key study dates	Results	Interpretation
Hogenkamp 2014 Based on 652 men aged 70 years at point of first alcohol measure (T0: 1990).	Alcohol exposure: single assessment (T0: 1990-1994) Outcome measures: baseline (T0), then follow-up ~7 years later (T1)	Selected outcome: executive function (Trail making test part B) Difference in mean change from baseline from regression model with alcohol modelled as a continuous variable of grams/day. Linear term: −0.325; *p* value = 0.471	Interpretation of the linear term was that the decline in executive function over time (7 years) was less as the dosage of alcohol increased per day. However, this term was not statistically significant.
Lang 2007a Based on 13,333 men and women (57% female) aged 65 years or above at point of first alcohol measure (T0: 1998).	Alcohol exposure: single assessment at baseline (T0: 1998) Outcome measures: single assessment ~4 years from baseline (T1: 2002)	Selected outcome: global cognitive function (sum of scores on 3 tests of memory; bottom quintile = poor cognitive function) Odds ratios (ORs) from logistic regression with alcohol modelled as a categorical variable Non-drinkers: OR > 1; *p* value < 0.05 > 0 to ≤ 1 drink/day (referent): 1.00 > 1 to ≤ 2 drinks/day: OR 0.82 (95%CI 0.64, 1.05) > 2 drinks/day: OR < 1; *p* value > 0.05	No evidence of a difference in the odds of poor cognitive function in the alcohol consumption categories (> 1 to ≤ 2 drinks/day; > 2 drinks/day) compared with the referent category. Some evidence that non-drinkers had a greater odds of poor cognition compared with the referent category. The relationship was not modified by sex (specific results not reported in primary study).
Piumatti 2018 Based on 13,342 men and women (54.7% female) aged 40-73 years at point of first alcohol measure (T0: 2006).	Alcohol exposure: single assessment (T0: 2006-2010; 2nd assessment not used in prospective analysis) Outcome measures: baseline (T0), then follow-up ~5 years later. (T1: 2011-2015)	Selected outcome: complex attention (processing speed based on reaction time task. Log reaction time. Higher score = worse cognition) Predicted difference in log reaction time (milliseconds, ms) from a restricted cubic spline model with alcohol modelled as a continuous variable of log grams/day (outcome): Linear effect up to 10 g/day (spline 1): −0.048 (log ms)(95%CI −0.105, −0.030); *p* value < 0.001 Non-linear effect (spline 2): 0.035 (log ms) (95%CI 0.007, 0.059); *p* value = 0.013	Interpretation of the linear effect up to 16 g/day (spline 1) was that for every 1 standard deviation unit in log grams alcohol/day, there was a predicted -0.048 standard deviation decrease in log reaction time. That is, cognitive performance improved up to 16 g/day. However, cognitive performance started to decline as alcohol consumption increased beyond 16 g/day. The study authors concluded that the relationship was modified by age for the non-linear effect, but was not modified by sex (for either of the effects).
Samieri 2013a Based on 6174 women aged ≥ 60 at point of first alcohol measure (T0: 1992).	Alcohol exposure: single assessment (T0: 1992-1995) Outcome measures: 3 assessments—T1 (average of 5.6 years from T0) and then T2 and T3 at ~2-year intervals (T1-T3: 1998-2004) Total length of follow-up: ~10 years from T0.	Selected outcome: global cognitive function (average of z-scores on 5 tests: overall cognition, language and memory. Higher score=better cognition). Mean difference (MD) from regression model with alcohol modelled as a categorical variable: Non-drinker (referent): 0 > 0 to < 15 g/day (median 2.9): MD 0.01 (95%CI −0.03, 0.05) ≥ 15 g/day (median 25.4): MD −0.02 (95%CI −0.10, 0.05)	No evidence of a mean difference in global cognitive function between the different levels of alcohol consumption compared with the referent category of no alcohol. No information on the scale range or standard deviation of the global cognitive function outcome is provided, precluding clinical interpretation.
Solfrizzi 2007 Based on 1445 men and women (44% female) aged 65 to 84 years at point of first alcohol measure (T0: 1992).	Alcohol exposure: single assessment at baseline (T0: 1992) Outcome measures: baseline (T0), then follow-up ~3.5 years later (T1: 1995–1996)	Selected outcome: Incidence of mild cognitive impairment (MCI; Petersen diagnostic criteria [64]) Hazard ratios (HR) from Cox proportional hazards model with alcohol modelled as a categorical variable: Categorical model: No-alcohol (referent): 1.00 ≤ 1 drink/day: HR 0.67 (95%CI 0.37, 1.21) > 1 to ≤ 2 drinks/day: HR 1.27 (95%CI 0.65, 2.47) > 2 drinks/day: HR 0.85 (95%CI 0.40, 1.81) Hazard ratios from Cox proportional hazards models with alcohol modelled as a continuous variable of *(*assumed by review authors*)* drinks/day (linear only; and linear and quadratic terms): Linear model: Linear term: HR 1.08 (95%CI 0.94, 1.24) Polynomial (quadratic) model: Linear term: HR 1.06 (95%CI 0.87, 1.28) Quadratic term: HR 1.00 (95%CI 0.98, 1.02)	No evidence of a difference in the relative rates of MCI in any of the alcohol categories compared to no alcohol consumption, however, the confidence intervals were wide. The relationship was not modified by sex (specific results not reported in primary study). No evidence of a linear trend between alcohol consumption and the rate of MCI (linear model). No evidence of a linear and quadratic trend between alcohol consumption and the rate of MCI (polynomial model). The relationship was not modified by sex (specific results not reported in primary study).
Wardzala 2018 Based on 486 men and women (75% female) aged ~80 years or above at point of first alcohol measure (T0: 2004 or 2008–09 depending on cohort).	Alcohol exposure: single assessment for most participants at baseline (T0) Outcome measures: not reported, ~6–7 annual assessments based on time in study (mean 6–8 years). Total length of follow-up: no information. Assume ~5–7 years from T0	Selected outcome: global cognitive function (mini mental state exam (MMSE). Higher score = better cognition). Annual rate of change in MMSE from a linear mixed model with alcohol modelled as a categorical variable: Women: Rare/never drinkers (referent): annual rate of change < 0 (i.e. MMSE declining over time) Moderate drinkers: annual rate of change not statistically significantly different (compared with referent category); *p* value > 0.05 Heavy drinkers: annual rate of change not statistically significantly different (compared with referent category); *p* value > 0.05 Men: Rare/never drinkers (referent): annual rate of change < 0 (i.e. MMSE declining over time) Moderate drinkers: annual rate of change reduced (compared referent category); *p* value < 0.01 Heavy drinkers: annual rate of change not statistically significantly different (compared with referent category); *p* value > 0.05	In women, the annual decline in global cognitive function was not found to be statistically significantly different between alcohol consumption categories and the referent category. In men, the annual decline in global cognitive function was not found to be statistically significantly different between the heavy drinkers and rare/never drinkers; however, it was found to be statistically significantly different between the moderate drinkers and the rare/never drinkers. The rate of cognitive decline was less in moderate drinkers. The primary study authors provided no clinical interpretation of the results beyond concluding based on statistical significance. Results are depicted in a figure, with some reporting in the text.

*For completeness, a brief summary of some study characteristics is replicated in this table (sample, key study dates, alcohol categories)

Full details are reported in the table of study characteristics, including alcohol categories and conversion of each category to grams per day

Solfrizzi 2007 found no evidence of an association between alcohol consumption and cognition using two different analysis methods. The authors reported that the associations were not modified by sex. Lang 2007a found the odds of poor cognition were greater for non-drinkers compared with those drinking > 0 to ≤ 1 drink/day (referent category). The odds of poor cognition in higher drinking categories (> 1 to ≤ 2 drinks/day; > 2 drinks/day) were less (i.e. ORs < 1) than the referent category, but were not statistically significantly different. The authors reported that the relationship was not modified by sex. Hogenkamp 2014 examined the linear association between alcohol consumption and executive function and found that the decline in executive function over time was less as the dosage of alcohol increased per day; however, the linear association was not statistically significant. Samieri 2013a found no evidence of a mean difference in global cognitive function between different levels of alcohol consumption compared with the non-drinker referent category. Piumatti 2018 examined the relationship between log alcohol and log reaction time using restricted cubic splines and found that cognitive performance improved up to 16 g alcohol/day but started to decline beyond 16 g. The authors concluded that the relationship was modified by age (for the non-linear effect), but was not modified by sex. Wardzala 2018 found that in females, the annual decline in global cognitive function was not found to be statistically significantly different between alcohol consumption categories and rare/never drinkers (referent category). In men, the annual decline in global cognitive function was not found to be statistically significantly different between the heavy drinkers and rare/never drinkers; however, it was found to be statistically significantly different between the moderate drinkers and the rare/never drinkers, with the rate of cognitive decline being less than in moderate drinkers.

#### Summary of findings table and assessment of certainty of the evidence

The summary of findings table (using the evidence profile format) is presented in Table 7.

**Table 7:** Summary of findings of the effect of different levels of alcohol consumption compared to no or very low level (zero to <10 g/week) consumption on cognition

Certainty assessment							Impact	Certainty	Importance (of outcome)
№ of studies	Study design	Risk of bias	Inconsistency	Indirectness	Imprecision	Other considerations			
Cognition (women only) (follow up: range 3 years to 13 years; assessed with: various scales and tests (standardised mean difference))									
5	observational studies	very serious ^a^	serious ^b^	not serious	not serious ^c^	none	**There was no important difference in cognitive function between women who consumed one to two standard drinks (<20 grams of alcohol) per day and non-drinkers but the evidence is very uncertain**. For alcohol consumption less than 25.9 grams alcohol/day (the point at which the predicted lower bound of the confidence interval crosses zero; ~2.5 standard drinks), cognition was slightly better in those consuming alcohol than current non-drinkers. However, the SMDs were small, with a maximum SMD of 0.18 (95%CI 0.02, 0.34), occurring at an intake of 14.4 grams alcohol/day. The effects are particularly uncertain at higher levels of alcohol consumption (>30 grams per day), since only one study (Kesse-Guyot 2012) contributes data for these levels of intake.	⨁◯◯◯ VERY LOW	CRITICAL
Cognition (men only) (follow up: range 1 years to 13 years; assessed with: various scales and tests (standardised mean difference))									
6	observational studies	very serious ^a^	serious ^d^	not serious	not serious ^c^	none	**There was no important difference in cognitive function between men who consumed one to two standard drinks (<20 grams of alcohol) per day and non-drinkers but the evidence is very uncertain.** The maximum SMD of 0.05 (95%CI 0.00, 0.10), occurring at an intake of 19.4 grams alcohol/day, was very small. For all levels of alcohol consumption, the predicted lower bound of the confidence interval of the SMD indicated that cognition was similar or poorer as compared to current non-drinkers, but the SMDs were small for alcohol intakes less than 55 grams/day. The effects are particularly uncertain at higher levels of alcohol consumption (>30 grams per day), since only one study (Kesse-Guyot 2012) contributes data for these levels of intake.	⨁◯◯◯ VERY LOW	CRITICAL
Cognition (women and men) (follow up: range 3 years to 34 years; assessed with: various scales and tests (standardised mean difference))									
4	observational studies	very serious ^a^	serious ^e^	not serious	not serious ^c^	none	**There was no important difference in cognitive function between adults who consumed one to two standard drinks (<20 grams of alcohol) per day and non-drinkers but the evidence is very uncertain.** The maximum SMD of 0.24 (95%CI -0.03, 0.51) occurred at an intake of 25 grams alcohol/day. For higher levels of alcohol consumption (e.g. >55 grams alcohol/day) there may be detrimental effects on cognition, however, this is where there is most uncertainty in the predictions, since only one study (Kitamura 2017) contributes data for these levels of intake.	⨁◯◯◯ VERY LOW	CRITICAL
Cognition (young people up to age 25) (follow up: range 12 months to years; assessed by: any scale or test or diagnostic criteria)									
0							None of the included studies examined the effects of different levels of alcohol consumption on cognition among young people, or the effects of different levels of alcohol consumption up to age 25 on cognition over the life-course (any age). Note, studies examining only acute effects (intoxication or withdrawal) were ineligible for the review.	-	CRITICAL

Explanations: a. Downgrade for very serious risk of bias. All studies were at serious risk of selection bias (due to lag time between initiating drinking and first alcohol measurement) and serious risk of misclassification of alcohol consumption status (no lifetime measures or measures of variation in consumption over time; recall bias). Also moderate-serious concern about bias arising from residual confounding and missing outcome data. b. Downgraded for serious inconsistency. There was evidence of heterogeneity in the study-specific dose-response coefficients (I^2^ = 69.5%, Q-test for heterogeneity p-value = 0.001). There are important differences between studies that may account for the observed heterogeneity, but it was not possible to explore whether these differences explained the observed heterogeneity. c. Not downgraded for imprecision despite wide confidence interval, since interpretation of the upper and lower bound of the interval suggests small, probably unimportant effects with considerable uncertainty due to the risk of bias and inconsistency. d. Downgraded for serious inconsistency. There was evidence of heterogeneity in the study-specific dose-response coefficients (I^2^ = 56.6%, Q-test for heterogeneity p-value = 0.011). There are important differences between studies that may account for the observed heterogeneity, but it was not possible to explore whether these differences explained the observed heterogeneity. e. Downgraded for serious inconsistency. There was evidence of heterogeneity in the study-specific dose-response coefficients (I^2^ = 47.2%). Differences between studies may account for the observed heterogeneity, but it was not possible to explore whether these differences explained the observed heterogeneity.

## Discussion

### Summary of main results

This review included 18 studies that examined the effects of different levels of alcohol consumption on cognitive function, 16 of which contributed to the summary or synthesis of quantitative results. Ten studies were included in dose-response analyses (5 in the analysis for women, 6 in the analysis for men, and 4 in the analysis for men and women).

The pooled dose-response relationship for women showed that for alcohol consumption less than 25.9 g alcohol/day, cognition was slightly better in those consuming alcohol than current non-drinkers (very low certainty evidence). However, the effect sizes (reported as SMDs) were small, with the largest effect (SMD 0.18 (95%CI 0.02, 0.34) at an intake of 14.4 g alcohol/day (< 2 standard drinks per day, based on standards in Australia, France, the Netherlands, the United Kingdom and several other countries). For men, the pooled dose-response relationship was similar in shape to that observed for women; however, the maximum SMD of 0.05 (95%CI 0.00, 0.10), occurring at an intake of 19.4 g alcohol/day, was very small (very low certainty evidence). Limitations in the design of studies contributing to these analyses are such that the observed effects may be biased.

### Overall completeness and applicability of the evidence

The studies included for review of the effects of different levels of alcohol consumption included participants at mid- to late-life, limiting applicability to other age groups (discussed below). Many of the studies reported single measures of cognition and had short-term follow-up (some without baseline assessment), so do not provide evidence about the persistence of observed effects. Only one study measured mild cognitive impairment using validated diagnostic criteria. Several studies reported measures of global cognitive function derived from a comprehensive battery of neurocognitive tests; however, the majority reported more limited measures that may be less suited to detecting mild cognitive impairment (e.g. MMSE scores).

None of the included studies examined the effects of different levels of alcohol intake on cognition among young people (up to age 25) or had measures of alcohol consumption among these age groups. Potentially eligible studies among this age group examined patterns of consumption but did not report analyses of the effects of different levels of consumption, or data that could be used in dose-response analyses. The absence of data following people from or close to the initiation of drinking in studies on the effects of average consumption has multiple ramifications. First, evidence about the effects of different levels of alcohol consumption on cognitive function among young people is lacking. Second, those who experience alcohol-related harm early in life may be missing from studies that begin in mid- to late-life, potentially leading to underrepresentation of the least healthy drinkers, and those who may be at most risk of cognitive impairment. Third, without measures of alcohol consumption early in life, studies are unable to reliably assess variation in average alcohol consumption or consumption patterns over the life-course. Consequently, studies may fail to differentiate between those who have very different historic patterns of consumption. All three issues limit the completeness and applicability of evidence in this review.

None of the studies included in the dose-response analysis examined whether the effects of alcohol were modified by co-morbidities or the use of licit or illicit drugs. We identified one eligible study that examined the effects of different levels of alcohol consumption among people with diabetes, and no studies involving people with other co-morbidities.

Our consideration of studies examining the effects of different patterns of consumption was limited to summarising study characteristics. Quite different patterns were examined across studies, and it is unlikely that studies examined sufficiently similar patterns to be meta-analysed, although more detailed review of this evidence is warranted.

### Quality of the evidence

Overall, the evidence contributing to the dose-response analyses reported in this review is of very low quality. This is partly due to inconsistent findings across studies, but the main reason for uncertainty is the serious risk of bias arising from limitations in the design of all studies. Many of the study design limitations are difficult to address, largely because of ethical issues that prevent randomised trials of alcohol consumption. Whereas in a randomised trial known and unknown risk factors for cognitive impairment would be balanced across groups through randomisation, this is not the case in a cohort study. In observational studies of alcohol, participants have ‘selected’ to drink alcohol or not. Decisions to drink—or not drink—may be associated with a range of characteristics that may, in turn, be risk factors for cognitive function (e.g. those with ill health may be less likely to drink or may quit drinking as their health declines). Although most studies attempted to control for these factors, residual confounding is likely. Issues with confounding were exacerbated because very few studies controlled for biases arising from the misclassification of drinkers as non-drinkers. Consequently, those with potentially elevated risk for cognitive impairment were likely to have been included in non-drinking groups. Finally, the evidence contributing to the review derives entirely from cohort studies involving participants at mid- to late-life, potentially excluding less healthy drinkers, at higher risk of cognitive impairment related to alcohol consumption.

### Potential biases in the review process

The review was conducted according to a pre-specified protocol with the aim of minimising biases in the review process. We conducted a comprehensive search of literature published from 2007 onwards. To minimise bias and error, we performed independent screening on samples of citations and full-text articles to ensure concordance, and a second person checked extraction of quantitative data (including that used to quantify alcohol intake) and risk of bias assessments. However, this is a rapid review, which inherently requires some methodological compromises that may introduce bias.

Due to the size of the reviewed literature, we were unable to perform double screening of all references, and we performed checks rather than independent assessment of the risk of bias and data extraction. However, we were over-inclusive in decisions to screen the full text of studies (retrieving full text of 5% of all citations, i.e. 228 papers from 4786 citations), reasons for exclusion were recorded when screening citations to facilitate verification of decisions, and the final list of included studies was cross-checked against a recent overview of systematic reviews [6] to ensure no studies were missed. At least three authors read all included studies (SB, JM, MP, JR), and a second author reviewed all papers for which there was uncertainty over inclusion or interpretation. All quantitative data were extracted and analysed by an experienced biostatistician (JM).

We did not contact authors for further information or data (with two exceptions, as documented in the methods). This meant that we may have missed subsequent publications of some studies published only as conference abstracts. It also meant that we relied on published data for our assessment of study design, risk of bias and for analysis.

### Limitations of the review

For most studies, assumptions were required to standardise alcohol consumption (i.e. to calculate doses of alcohol in grams per day) and to calculate the statistics required to standardise effect measures (i.e. compute the standardised mean differences, SMDs). While these assumptions are not expected to bias results of the systematic review, limitations arise from making such assumptions. For example, where the authors did not specify the number of grams of alcohol in a standard drink, we standardised using published definitions of a standard drink for the country in which the study was conducted. It is possible that a different standard (or no standard) was used in these studies, which might have led to a slight over- or under-estimate of the level of alcohol intake. However, the alternative would have been not to standardise, making comparison across studies impossible. Importantly, standardising alcohol consumption and effect measures was a necessary step for enabling comparisons of findings across studies, irrespective of whether results were then pooled in a statistical analysis or not. Hence, any limitations arising from standardisation would have applied whether we reported standardised results from single studies, pooled results in pairwise meta-analyses (i.e. examining whether cognitive function differs for one level of alcohol consumption compared to another, for example < 10 g/week compared to ≥ 20 g/day to < 30 g/day), or pooled results in a dose-response analysis (i.e. examining whether cognitive function differs with increasing levels of alcohol consumption).

A further limitation of the review is that we did not report or synthesise results from studies that examined the effect of patterns of alcohol consumption. While dose-response analyses based on the average level of alcohol consumption provide important information, they do not account for the potentially harmful effects of different patterns of consumption and may mask such effects. In particular, the effects of irregular consumption above lower risk levels (e.g. weekly or monthly “binge” drinking) and the effects of drinking early on the life-course (e.g. less than 25 years of age) need to be examined. A simple, yet questionable, approach to considering results from studies examining different patterns of alcohol consumption would have been to report conclusions from the abstracts of included studies. However, given the known biases in the reporting of conclusions in the abstracts of non-randomised studies (see, for example [65]), and the number of analyses reported in each included study, it is unlikely that this would provide a valid summary of the evidence.

## Authors’ conclusions

### Implications for policy

We found that there is currently very low certainty evidence showing a very small, probably unimportant, beneficial effects on cognition at levels of alcohol consumption at or below those currently indicated as lower risk for women and men in the 2009 Australian Guidelines, and those of New Zealand, and a number of European countries including the United Kingdom (i.e. two standard drinks or < 20 g of alcohol per day). The extent to which this reflects a true effect or bias arising from limitations of studies included in the systematic review cannot be determined.

### Implications for research

Published research examining the effects of different levels of alcohol consumption on cognition has a number of limitations, some of which could be addressed through adherence to the STROBE guidelines for reporting observational studies [66, 67]. The reporting of key elements of study design was particularly problematic, with many studies omitting information, or reporting ambiguous information, about the timing of data collection for alcohol exposure and cognition outcomes. In several studies, it was impossible to determine whether cross-sectional or longitudinal data were collected and whether the alcohol data used in analyses were entirely prospective or collected concomitantly with follow-up measures of cognition. Other problematic reporting practices included not presenting baseline characteristics (including covariate data) for each of the alcohol categories for which results were reported (needed to examine baseline imbalance), and not summarising information about missing data by alcohol categories (needed to examine whether there was a differential loss to follow-up across groups). Collectively, these problematic reporting practices may have led to an unnecessary exclusion of some studies based on design or a more serious rating of risk of bias than necessary.

More challenging to address are study design limitations that may bias the observed effects of alcohol on cognition in observational studies. The methodological literature on alcohol epidemiology identifies numerous recommendations for the study design that were not widely implemented in the studies included in this review. For example, methodological studies have identified and provided empirical evidence about methods for measuring alcohol, and dealing with potential bias and confounding arising from misclassification of alcohol consumption (see for example, [20, 68–72]). These practices were rarely implemented in studies included in this review. Greater attention to applying these and other best-practice methods may increase the certainty of evidence arising from future research.

## Supplementary information

**Additional file 1.** Protocol for the systematic review

**Additional file 2.** Appendices 1 to 10

## Data Availability

Requests for data should be sent to the corresponding author.

## Abbreviations

95% CI: 95% confidence interval
ACE‐R: Addenbrooke’s Cognitive Examination ‐ Revised
AWC: Alcohol Working Committee
CI: Cognitive impairment
COWAT: Controlled Oral Word Association Test
CVD: Cardiovascular disease
DSCT: Digit symbol coding test
DSST: Digit symbol substitution test
g: grams
GCF: Global cognitive function
GFQ: Graduated frequency questionnaire
GRADE: Grading of Recommendations Assessment, Development and Evaluation
HR: Hazard ratio
HVLT‐R: Hopkins verbal learning test
MCI: Mild cognitive impairment
MD: Mean difference (usually based on a scale score or test)
MMSE: Mini Mental State Examination
MOCA: Montreal Cognitive Assessment
ms: milliseconds
NHMRC: National Health and Medical Research Council
NIAAA: National Institute on Alcohol Abuse and Alcoholism (United States)
NIA‐AA: National Institute on Aging and the Alzheimer’s Association (United States)
ONHMRC: Office of the National Health and Medical Research Council
OR: Odds ratio
PECO: Population, Exposure, Comparator, Outcome
PRISMA: Preferred Reporting Items for Systematic Reviews and Meta‐Analyses
PRISMA‐P: Preferred Reporting Items for Systematic Reviews and Meta‐Analyses ‐ protocols
RoB: Risk of bias
ROBINS‐I: Risk Of Bias In Non‐randomized Studies of Interventions
SCD: Specific cognitive domain
SD: Standard deviation
SE: Standard error
SMD: Standardised mean difference
SR: Systematic review
STROBE: Strengthening the Reporting of Observational Studies in Epidemiology
T0, T1, etc: Time 0: 1^st^ measurement point; time 1: 2^nd^ measurement point
TICS: Telephone Interview for Cognitive Status
TMT‐A & TMT‐B: Trail making test part A; Trail making test part B
