# Supplementary material for: Long-term effects of alcohol consumption on cognitive function: a systematic review and dose-response analysis of evidence published between 2007 and 2018
Source: Syst Rev. 2020 Feb 13;9:33. doi: 10.1186/s13643-019-1220-4 (PMC7020517; doi:10.1186/s13643-019-1220-4)
Supplement: Supplementary file 1 — Additional file 1. Protocol for the systematic review [file 13643_2019_1220_MOESM1_ESM.pdf]

Contents

Protocol for a systematic review of the association between different levels and patterns of alcohol consumption and long-term cognitive impairment 2

Authors and contributors to the protocol 2

Declarations of interest 2

1. Background 3

2. Objectives 3

3. Methods 4

3.1 Criteria for considering studies for this review 4

3.1.1 Types of participants 4

3.1.2 Types of exposure 4

3.1.3 Types of comparator exposure 5

3.1.4 Types of outcomes 5

3.1.5 Types of studies 8

3.2 Search methods for identification of studies 9

3.2.1 Systematic reviews 9

3.2.2 Primary studies 10

3.3 Data collection and analysis 12

3.3.1 Selection of studies 12

3.3.2 Data extraction and management 12

3.3.3 Assessment of risk of bias of included studies 14

3.3.4 Measures of association 15

3.3.5 Unit of analysis issues 16

3.3.6 Assessment of heterogeneity 16

3.3.7 Assessment of reporting biases 16

3.3.8 Data synthesis 16

3.3.9 Summary of findings tables and assessment of certainty of the body of evidence 18

4. References 20

Appendix: Database search strategies 23

Search strategy for Ovid Embase 23

Search strategy for Ovid PsycINFO 23

Protocol for a systematic review of the association between different levels and patterns of alcohol consumption and long-term cognitive impairment

Authors and contributors to the protocol

Sue Brennan	Senior Evidence Officer responsible for leading the review. Contributed to the design of the review. Wrote the protocol with contributions from other authors as described.
Joanne McKenzie	Drafted the review questions. Wrote the analysis plan and method for reporting treatment effects. Provided statistical advice on study appraisal and interpretation. Planned contributions to the analysis and interpretation.
Steve McDonald	Developed the search strategy and wrote the search methods. Critical review of the protocol.
Stephanie Ward	Provided clinical advice, especially in relation to eligibility criteria and identification of confounding domains. Critical review of the protocol.
Matthew Page	Wrote sections of the risk of bias assessment and provided advice on risk of bias assessment. Planned contributions to data extraction and appraisal of studies.
Andrew Forbes	Provided statistical advice for development of the analysis plan. Critical review of the protocol with planned contributions to the analysis and interpretation.
Sally Green	Critical review of the protocol and project oversight
Jane Reid	Named reviewer with planned contributions to the selection of studies (screening), data extraction, and appraisal of studies.
Emily Karahalios	Named reviewer with planned contributions to the analysis.

Declarations of interest

All authors declare they have no financial, personal or professional interests that could be construed to have influenced the conduct or results of this systematic review.

1. Background

The National Health and Medical Research Council (NHMRC), is updating the 2009 *Australian Guidelines to Reduce Health Risks from Drinking Alcohol (the Alcohol Guidelines)* to ensure the Guidelines are based on an evaluation of the latest and best scientific evidence on the health effects (risks and benefits) of alcohol consumption. The review team was contracted by the National Health and Medical Research Council (NHMRC) to design and undertake the systematic review described in this protocol.

The current systematic review aims to address identified gaps in existing review evidence about the association between levels and patterns of alcohol consumption and long-term cognitive impairment. It will consider evidence published from 2007 onwards (i.e. subsequent to the evidence review conducted for the 2009 *Alcohol Guideline*) about the association between alcohol consumption and long-term cognitive function. Findings from this review will need to be interpreted in combination with evidence published prior to 2007.

2. Objectives

The objectives of the review are to address the following questions.

1. What is the long-term effect of different levels of alcohol consumption compared to never drinking or very low level drinking (zero to < 1og/week) on cognition for women and men, where the levels are defined as:
    - ≥10 g/week and <10 g/day
    - ≥ 10 and <20 g/day
    - ≥ 20 g/day and <30 g/day
    - ≥ 30 and <40 g/day
    - ≥ 40 g/day and <50 g/day
    - ≥ 50 g/day
2. What are the long-term effects of different patterns of alcohol consumption compared to never (or very low level) drinking on cognition for women and men?
    - Patterns of alcohol consumption may include, irregular heavy drinking (episodic or “binge” drinking) or daily drinking at levels identified as “low risk” in the 2009 *Alcohol guideline*.
3. What is the long-term effect of one pattern of alcohol consumption compared to a different pattern of alcohol consumption on cognition for women and men?

Secondary objectives

4. Is there a dose-response relationship between levels of alcohol consumption and long-term cognitive effects for women and men?
5. Is the effect of alcohol consumption and long-term cognitive effects modified by age, co-morbidities, or drug use?

3. Methods

Methods reported in this protocol are based on the *Cochrane Handbook for Systematic Reviews of Interventions* (Higgins 2011), with modifications for undertaking a review of exposures. The GRADE approach will be used to summarise and assess the certainty of evidence arising from this review (see Section 3.3.9 for details). GRADE methods are widely used in guideline development to ensure a systematic, transparent and common approach to interpreting results (Schunemann 2013). The protocol is reported in accordance with the PRISMA-P statement (Moher 2015, Shamseer 2015).

3.1 Criteria for considering studies for this review

3.1.1 Types of participants

General population

Studies that are limited to one or more of the following subgroups will also be eligible for inclusion:

- People in specific age groups identified in the 2009 *Alcohol guideline* as potentially having a higher risk of harm from alcohol exposure than the general population. For example, children and young people (less than 18 years), young adults (18-25), older people (65 and over)
- Women or men

If available separately, data and analyses from studies that meet other eligibility criteria will be reported for the following subgroups.

- people with existing health conditions (physical, mental or both)
- people using licit and/or illicit drugs
- people with a family history of alcohol dependence.

Studies restricted to one or more of these three subgroups will be eligible only if the study explicitly aims to examine the association between alcohol consumption and long-term cognition.

3.1.2 Types of exposure

Eligible studies are those examining different levels of alcohol consumption, patterns of alcohol consumption, or both.

Studies must report alcohol consumption in units that allow quantification of the average amount of alcohol consumed (e.g. grams or millilitres of pure alcohol) over a period of time (e.g. per day, week, month).

Studies are eligible irrespective of the methods used to measure alcohol exposure. We anticipate that these methods will vary across studies, but may include retrospective survey involving recall of alcohol consumption over different periods of life or intake diaries to measure current alcohol consumption. Single or repeated measures of exposure may be collected. The timing of measurement should match that indicated in the study design features listed in section 3.1.5. To account for differences in the methods used to measure alcohol exposure, we will extract data on the measurement methods and assess potential biases and confounding that may arise through the method used.

3.1.3 Types of comparator exposure

For inclusion in the review, the comparator group must include a different level or pattern of alcohol consumption.

For inclusion in the meta-analysis of different levels of alcohol consumption and the dose response analysis, studies must report results for either a 'never' drinker group or a 'very low-level' drinker group. We broadly define 'never' drinkers as individuals that have never consumed a serve of alcohol (lifetime abstainers) or have consumed very little alcohol across their lifetime. We define very low-level drinkers as those whose average alcohol consumption is zero to <10g/week. The latter threshold reflects consumption of a single Australian standard drink (10 grams of alcohol).

We anticipate diversity across studies in the definition and composition of potentially eligible comparator groups (which may or may not be the referent group to which other categories of alcohol consumption are compared in each study) (Naimi 2017). For example, across studies referent groups have been defined as never drinking (Knott 2015), not drinking above a certain threshold (e.g. less than one unit of alcohol per week (Topiwala 2017)), and not drinking over a defined period of time (e.g. less than one unit over the preceding 12 months (Australian Institute of Health and Welfare 2017)). Studies reporting a group with these or similarly low levels of alcohol consumption will be eligible, irrespective of whether the group is used as the referent in the study.

For inclusion in the meta-analysis of patterns of drinking, eligible comparators are 'never drinking' or a different pattern of drinking. For example, comparison of irregular heavy drinking (episodic or "binge" drinking) with daily drinking at levels identified as "low risk".

We anticipate that some studies will use methods for measuring alcohol consumption that may result in misclassification of former drinkers as abstainers or very-low level drinkers. Specifically, in studies that only collect data on current rather than lifetime exposure (e.g. alcohol consumption over the preceding 12 months), former drinkers may be misclassified as abstainers (Naimi 2017). Since former drinkers have been shown to have poorer self-reported health and higher levels of depression than current drinkers (both associated with cognition), this misclassification has implications for the comparability of groups and confounding (Knott 2015, Naimi 2017). Rather than exclude studies that may have misclassified former drinkers, we will assess potential for confounding from imbalance in prognostic factors associated with cognition, and consider this in our interpretation of findings (Sterne 2016).

3.1.4 Types of outcomes

Eligible studies are those that report at least one measure of cognitive function (or performance), which is the primary outcome for this review. Studies must assess cumulative long-term effects of alcohol consumption on cognitive function. We will exclude studies that only examine acute effects (during intoxication or withdrawal), long-term effects arising from injury on a single drinking occasion (e.g. a traumatic brain injury sustained while intoxicated), and those where there is insufficient length of follow-up to examine the longer-term effects of cumulative exposure (< 6 months). While we have not set a minimum threshold for 'long-term', we will consider the extent to which studies provide evidence of a sustained effect, and the duration of this effect, when interpreting results (see *Timing of outcome measurement*).

Eligible outcomes may be broadly categorised as follows.

Cognitive function

- global cognitive function

- domain-specific cognitive function (especially domains that reflect specific alcohol-related neuropathologies, such as psychomotor speed and working memory)

Clinical diagnoses of cognitive impairment

- mild cognitive impairment (also referred to as mild neurocognitive disorders)
- major cognitive impairment (also referred to as major neurocognitive disorders; includes dementia)

These conditions are "characterised by a decline from a previously attained cognitive level" ((Livingston 2017), p2675).

We expect that definitions and diagnostic criteria will vary across studies, so will accept a range of definitions as noted under *Methods of outcome assessment*. Table 1 provides an example of specific domains of cognitive function used in the diagnosis of mild and major cognitive impairment in the Diagnostic and Statistical Manual of Mental Disorders, Fifth edition (DSM-5) (Black 2014)).

We will only include eligible studies reporting all-cause dementia if they provide relevant data not assessed in the systematic review by Xu et al, since that review provides an up-to-date synthesis (Xu 2017).

Table 1. Domains used to diagnose major and mild neurocognitive disorders in the DSM-5

Domain	Cognitive abilities covered by the domain
Complex attention	sustained attention, divided attention, selective attention, processing
Executive function	planning, decision making, working memory, responding to feedback/error correction, overriding habits, mental flexibility
Learning and memory	immediate memory, recent memory
Language	expressive language and receptive language
Perceptual-motor ability	construction and visual perception
Social cognition	recognition of emotions, theory of mind, behavioural regulation

Methods of outcome assessment

Any measure of cognitive function is eligible for inclusion. The tests or diagnostic criteria used in each study should have evidence of validity and reliability for the assessment of mild cognitive impairment, major cognitive impairment, or both (according to the study question).

We anticipate that many different methods will be used to assess cognitive functioning across studies. These include the following.

Clinical diagnoses of

- mild cognitive impairment using explicit criteria (e.g. (Winblad 2004); any of the definitions of mild cognitive impairment described in (Matthews 2008))

- major cognitive impairment using validated criteria (e.g. DSM-V, DSM-IV, ICD-10, NINCDS ADRDA criteria)

Neuropsychological tests used to assess global cognitive function, for example the:

- Mini Mental State Examination (MMSE)
- Addenbrooke's Cognitive Examination - Revised (ACE-R) which "incorporates the MMSE and assesses attention, orientation, fluency, language, visuospatial function, and memory, yielding subscale scores for each domain" (Davis 2013)
- Montreal Cognitive Assessment (MOCA), which provides measures for specific cognitive abilities and may be more suitable for assessing mild cognitive impairment than the MMSE (Davis 2013)

Neuropsychological tests for assessing domain-specific cognitive function, for example tests of:

- attention and processing speed, for example the Trail making test (TMT-A)
- memory, for example the Hopkins verbal learning test (HVLT-R; immediate, delay)
- visuospatial ability, for example the Block design test
- executive function, for example the Controlled Oral Word Association Test (COWAT)

Results may be reported as an overall test score that provides a composite measure across multiple areas of cognitive ability (i.e. global cognitive function), sub-scales that provide a measure of domain-specific cognitive function or cognitive abilities (e.g. processing speed, memory), or both.

Timing of outcome measurement

Studies with a minimum follow up of 6 months will be eligible. This threshold is based on previous reviews examining the association between long-term cognitive impairment and alcohol consumption (e.g. Anstey 2009 specified 12 months (Anstey 2009)) and guidance from the Cochrane Dementia and Cochrane Improvement Group, which suggests a minimum follow-up of nine months for studies examining progression from mild cognitive impairment to dementia (Davis 2013). We have deliberately specified a shorter period to ensure studies reporting important long-term effects are not missed.

No restrictions will be placed on the number of points at which the outcome is measured, but the length of follow up and number of measurement points will be considered when interpreting study findings (especially in the GRADE assessment of indirectness) and in deciding which outcomes are similar enough to combine for synthesis. Since long-term cognitive impairment is characterised as a decline from a previous level of cognitive function and implies a persistent effect, studies with longer-term outcome follow up at multiple time points should provide the most direct evidence.

Selection of cognitive outcomes where multiple are reported

We anticipate that individual studies may report data for multiple cognitive outcomes.

Specifically, a single study may report results:

- for *multiple constructs* related to cognitive function, for example global cognitive function and cognitive ability on specific domains (e.g. memory, attention, problem-solving, language);

- using *multiple methods or tools to measure* the same or similar outcome, for example reporting measures of global cognitive function using both the MMSE and the MOCA;
- at *multiple time points*, for example at one, five and 10 years.

We will select one outcome for inclusion in analyses and for reporting the main outcomes (e.g. for GRADEing), choosing the result that provides the most complete information for analysis. Where multiple results remain, we will list all available outcomes (without results) and ask our content expert to independently rank these based on relevance to the review question, and the validity and reliability of the measures used. Methods for selecting results when there are multiple effect estimates and/or analyses are described in Sections 3.3.4 and 3.3.9.

Secondary outcomes

Brain structure outcomes (as measured by neuroimaging) will be included from studies that report a cognitive function outcome (i.e. studies reporting only a brain structure outcome with no measure of cognitive function will be excluded).

Excluded outcomes

In line with recommendations from the Cochrane Dementia and Cognitive Improvement Group (Harrison 2016), surrogate outcomes are ineligible, for example:

- brain structure and function, in the absence of a measure of cognitive function
- biomarkers

3.1.5 Types of studies

Cohort studies and nested case-control studies are eligible for inclusion in the review.

Broadly, these types of designs can be described as follows.

- *Cohort*: "a study in which a defined group of people (the cohort) is followed over time, to examine associations between different … [exposures] and subsequent outcomes" (Reeves 2011).
- *Nested case-control*: a study in which "Individuals experiencing an outcome of interest are identified from within a defined cohort (for which some data have already been collected) and form a group of 'cases'. Individuals, often matched to the cases, who did not experience the outcome of interest are also identified from within the defined cohort and form the group of 'controls'." Data characterising prior exposure "are collected retrospectively". (Reeves 2011). Data on alcohol exposure should be collected from existing records, since those experiencing cognitive decline may not to be able to provide sufficiently valid and reliable information about their prior exposure.

In line with current Cochrane guidance, decisions about study eligibility will be based on assessment of the study design features listed in Table 2 rather than labels ('cohort' or 'case-control') or broad definitions of each type of study.

Table 2. Design features for determining study eligibility (adapted from (Reeves 2011))

Study design feature	Prospective cohort	Retrospective cohort	Nested case-control
A comparison between two or more groups of participants with different levels or patterns of alcohol exposure (one of which is no or very low-level exposure)	Yes	Yes	Yes

Study design feature	Prospective cohort	Retrospective cohort	Nested case-control
Participants were allocated to groups based on different levels or patterns of alcohol exposure	Yes	Yes	No (based on outcome)
The following parts of the study were prospective:			
• identification of participants	Yes	No	Yes
• assessment of baseline and allocation to exposure group	Yes	No	Yes
• assessment of outcomes	Yes	Possibly	Yes
• generation of hypotheses	Yes	Yes	Yes
Assessment of comparability of groups was based on:			
• potential confounders	Possibly	Possibly	Possibly
• outcome variables at baseline	Possibly	Possibly	No

While eligible for this review, randomised trials examining the effects of different levels and/or patterns of alcohol exposure are unlikely to be conducted because of ethical concerns and the length of follow-up required to measure long-term cognitive outcomes.

Excluded designs. Case-control studies will be excluded, except for nested case-controls. Case control studies compare "people with a specific outcome of interest ('cases') with people from the same source population but without that outcome ('controls'), to examine the association between the outcome and prior exposure" (Reeves 2011). This design is unsuitable for addressing the objectives of this review, since it is unlikely to be possible to obtain valid and reliable estimates of prior exposure to alcohol from individuals with the outcome of interest (cognitive impairment).

Studies using other designs (before-after comparisons, cross sectional studies) will be excluded since it is difficult (if not impossible) to attribute observed changes in outcomes to the exposure (Reeves 2011).

Date and language restrictions. Studies published from 2007 onwards are eligible for inclusion. Studies published in languages other than English will be excluded. A recent study has shown that the exclusion of studies in languages other than English rarely impacts on the results and conclusion of a review (Hartling 2017); a finding that is consistent with an earlier study that found no evidence that English-language restriction introduces systematic bias in meta-analytic results (Morrison 2012).

3.2 Search methods for identification of studies

Our approach combines searching for systematic reviews as well as primary studies. These searches will be limited to bibliographic databases and checking the reference lists of eligible studies.

3.2.1 Systematic reviews

The independent evidence evaluation on the health effects of alcohol consumption commissioned by NHMRC (NHMRC Clinical Trials Centre 2017) listed 13 systematic reviews (published between 2007 and 2016) that related to alcohol and cognitive impairment. From these reviews we will retrieve primary studies that meet the eligibility criteria. In addition, we have identified systematic reviews published since 2016, and will ensure that any relevant primary studies included in these reviews are retrieved and considered for inclusion. The systematic reviews search will be limited to MEDLINE (see

Table 3).

Table 3. Search strategy for systematic reviews (Ovid MEDLINE)

#	Search statement
1	exp Alcohol drinking/
2	exp Alcoholic Beverages/
3	Alcoholic intoxication/
4	Alcoholism/
5	exp Alcohol-Related Disorders/
6	(alcohol$ or drinking or wine).tw.
7	or/1-6
8	exp Dementia/
9	exp Cognitive Dysfunction/
10	(dementia or cognition or cognitive).tw.
11	or/8-10
12	(meta-analysis or review).pt.
13	(systematic$ and (review$ or overview$)).tw.
14	(meta?analy$ or meta analy$).tw.
15	or/12-14
16	7 and 11 and 15
17	(2017$ or 2018$).dt.[1]
18	16 and 17

[1] added to MEDLINE in 2017-2018, irrespective of year of publication

3.2.2 Primary studies

The primary studies we identify from existing systematic reviews will serve as the initial source of studies. We have also used information about how these studies are indexed (i.e. thesaurus terms, text words) to help develop and validate the search strategy for primary studies. This technique, referred to as capture-recapture, is particularly useful when a reasonable number of studies (~20) are involved.

Independently of the search for systematic reviews, we will search for primary studies relevant to the review question published since January 2007. No language or geographic limitations will be applied. Searches will be limited to MEDLINE, Embase and PsycINFO.

The search strategy for Ovid MEDLINE (including In-Process & Other Non-Indexed Citations) (Table 4) is based on an assessment of the 2009 systematic review by Anstey (Anstey 2009) and the more recent 2017 meta-analysis by Xu (Xu 2017). The searches conducted for the Anstey review were very broad, generating over 33,000 citations, of which 15 were ultimately included in the meta-analysis. The MEDLINE search below (line 19) retrieves all the studies

included in the Anstey review but is considerably more precise. This search also retrieves all seven additional studies included in the meta-analysis by Xu.

We decided not to include the text word ‘impairment’ as a stand-alone term since records retrieved using this text word (not already retrieved by the text words ‘cognition’ or ‘cognitive’) are mostly concerned with kidney or liver impairment, or some other impairment, and are unrelated to cognition.

The MEDLINE search has been translated for Embase and PsycINFO, incorporating each database’s relevant thesaurus terms for alcohol, dementia/cognitive impairment and study design (Appendix).

Table 4. Search strategy for primary studies (Ovid MEDLINE)

#	Search statement
1	exp Alcohol Drinking/
2	exp Alcohol-Related Disorders/
3	exp Alcoholic Beverages/
4	(alcohol$ or drinking or wine).tw.
5	or/1-4
6	exp Dementia/
7	exp Cognition Disorders/
8	(dementia or cognition or cognitive or neurocognit$ or neuro-cognit$ or alzheimer$).tw.
9	or/6-8
10	exp Cohort Studies/
11	Controlled Clinical Trial.pt.
12	exp Case-Control Studies/
13	Risk Factors/
14	(cohort$ or longitudinal or follow-up or “follow up”).tw.
15	(case$ adj3 control$).tw.
16	or/10-15
17	5 and 9 and 16
18	Animals/ not Humans/
19	17 not 18
20	limit 19 to yr=”2007 –Current”

Beyond database searching, we will check the reference lists of eligible studies for additional relevant publications.

3.3 Data collection and analysis

3.3.1 Selection of studies

Citations identified from the literature searches and reference list checking will be imported to EndNote and duplicates removed. Citations may then be imported to Covidence (www.covidence.org), an online tool that streamlines the screening stage of a systematic review. Three reviewers will independently screen a sample of citations to pre-test and refine coding guidance based on the inclusion criteria. Disagreements about eligibility will be resolved through discussion, and further studies screened until concordance is achieved. One reviewer (SB, JR or SM) will then screen remaining citations (titles and abstracts) for inclusion in the review using the pre-tested coding guidance.

Full-text of all potentially eligible studies will be retrieved and independently screened by two reviewers (SB, JR or SM), with disagreements resolved using the same approach as for citation screening. Advice may be sought from the review content expert (SW) to confirm eligibility based on PECO or biostatistician (JM or AF) to confirm eligibility based on study design. If a study does not contain sufficient information for a decision to be made about its eligibility, further information may be sought from the study's authors. Citations that do not meet the inclusion criteria will be excluded and the reason for exclusion will be recorded at full-text screening.

Cohort names, author names, and study titles, locations and dates will be used to identify multiple reports arising from the same study.

3.3.2 Data extraction and management

For each included study, one review author (SB, SM, or JR) will extract data relating to study characteristics using a pre-tested data extraction and coding form, and a second author (SB, SM, or JR) will independently verify the data. Two authors will independently extract quantitative data (MP, SB, SM). Discrepancies will be resolved through discussion, and advice sought from the review content expert (SW) or biostatistician (JM, AF) if agreement cannot be reached or for more complex scenarios.

Pre-testing of the data extraction and coding form will be done on three studies purposefully selected from the included studies to cover the diversity of data types anticipated in the review. Advice will be sought from the review content expert (SW) and biostatisticians (JM or AF) to ensure data are extracted as planned. Revisions to the data extraction form will be made as required to maximise the quality and consistency of data collection.

We will extract information relating to the characteristics of included studies and results as follows.

1. Study identifiers and characteristics of the study design

    - Study references (multiple publications arising from the same study will be matched to an index reference)

    - Study or cohort name, location and commencement date

- Study design (categorised as 'prospective cohort study', 'retrospective cohort study', 'nested case-control study', or 'other' using the checklist of study design features developed by Reeves and colleagues, (Reeves 2017))
- Funding sources and funder involvement in study.

2. Characteristics of the exposure and comparator groups

- Levels of exposure as defined in the study, including details of how exposure was measured and categorised, and information required to convert data for reporting and analysis
    - qualitative descriptors of each category, if used (e.g. never or non-drinker, abstainer, former drinker, low/moderate/heavy consumption)
    - upper and lower boundaries of each category (e.g. 1 to 29 grams per day; 5.1-10 units per week based on a standard drink in the UK)
    - group used as referent category (comparator) in analyses and how defined
    - units of measurement (e.g. standard units of alcohol per day and definition of unit)
    - method of collecting exposure data (e.g. retrospective survey involving recall of alcohol consumption over different periods of life; intake diaries to measure current alcohol consumption); time points at which exposure data were collected
    - sample size for each exposure group at each measurement point and included in analysis; number lost to follow up
    - any additional parameters used to derive each category or exposure measure (e.g. alcohol consumption at each drinking occasion; frequency of drinking; recall period)
- Patterns of exposure
    - Any additional data not listed above that characterises and quantifies different patterns of alcohol exposure (e.g. consumption on heaviest drinking day; diagnosis of an alcohol use disorder such as dependence or harmful drinking, and the method of assessment; definition of other frequency-based categories used to characterise patterns of drinking such as occasional drinking or infrequent consumption).
- Duration/length of exposure period at study baseline and follow-up (directly reported or data that can be used to calculate)
- Age at commencement of drinking (initial exposure)

3. Characteristics of participants

- Age at baseline and follow up
- Sex
- Ethnicity

- Co-morbidities
- Socio-economic status (including education)
- Use of licit or illicit drugs
- Family history of alcohol dependence
- Other characteristics of importance within the context of each study

4. Outcomes assessed and results
   - Outcomes domains (e.g. cognition, brain structure, function in daily life)
   - For cognition outcomes:
     - Measurement method (e.g. Montreal cognitive assessment) and time points
     - Potential confounders, co-exposures and other sources of bias mentioned in the paper (Sterne 2016). Baseline statistics of the confounders to allow assessment of the comparability of the exposure groups.
     - Results including: summary statistics (means and standard deviations, or number of events for cognitive outcomes that have been dichotomised, and sample size) in each exposure category, unadjusted and adjusted estimates of the associations (e.g. mean differences, confidence intervals, t-values, p-values, or risk ratios/odds ratios for binary outcomes) overall and stratified by the specified subpopulations, where possible. For adjusted estimates, we will extract information on the analysis method, how confounding was adjusted, and which confounders were adjusted for.
     - Data required to assess risk of bias (see Section 3.3.3) (Sterne 2016)

3.3.3 Assessment of risk of bias of included studies

One author (MP, SB, SM or JR) will assess risk of bias for each included study using the ROBINS-I (Risk Of Bias In Non-randomized Studies of Interventions) tool (Sterne 2016), and a second author (MP, SB, SM, or JR) will independently verify the assessments. Discrepancies will be resolved through discussion, with advice from a third reviewer (JM or AF) if agreement cannot be reached or for more complex scenarios. To ensure concordance, the assessment process will be piloted by all assessors (MP, SB, SM, JR, JM) on two included studies, with further piloting if required.

ROBINS-I was developed for "evaluating risk of bias in estimates of the comparative effectiveness (harm or benefit) of interventions" from non-randomised studies (i.e. where randomisation was not used to allocate individuals to comparison groups) (Sterne 2016). While alcohol is generally considered an exposure, ROBINS-I has been successfully applied to equivalent studies (e.g. those examining the association between change in body size and mortality) and has advantages over checklist approaches in that it facilitates an overall judgement of RoB that can be incorporated in the analysis and the GRADE assessment (Sterne 2016, Schunemann 2018). However, if infeasible to apply ROBINS-I due to inclusion of a large number of studies, we will assess studies using the Agency for Health Research and Quality (AHRQ) Item Bank for Assessing Risk of Bias and Confounding for Observational Studies of Interventions or Exposures (Viswanathan 2013)

ROBINS-I requires assessment of the following seven domains:

1. Bias due to confounding
2. Bias in selection of participants into the study
3. Bias in classification of interventions (exposure)
4. Bias due to deviations from intended interventions (exposures)
5. Bias due to missing data
6. Bias in measurement of outcomes
7. Bias in selection of the reported result

It is recommended that users applying ROBINS-I should consider in advance the confounding factors and co-interventions that have the potential to lead to bias in included studies. These are listed at the end of this section.

Within each domain, we will judge risk of bias as "low" (comparable to a well performed randomized trial), "moderate" (sound for a non-randomized study), "serious" (there are some important problems) or "critical" (the study is too problematic to provide useful evidence).

We will rate the *overall risk of bias* for each result based on the most serious risk of bias judgement across any of the seven the domains (i.e. overall risk of bias is "serious" if at least one domain is rated "serious"). If we judge a result to be at "critical" risk of bias on the first domain (bias due to confounding), we will not assess other domains, since the overall risk of bias for the result will be "critical" by default. We will exclude results of studies at "critical" risk of bias overall from the syntheses and they will not contribute to our conclusions. For each study and result (outcome) assessed, we will report our judgment of risk of bias by domain and provide a rationale for the judgment with supporting information. Our risk of bias judgments will be described in the characteristics of included studies table.

Pre-specification of confounding factors and co-exposures

Confounding domains are "prognostic variables (factors that predict the outcome of interest)" that also predict the exposure at baseline (Sterne 2016). ROBINS-I defines important confounding domains as those "for which, in the context of [a specific] study, adjustment is expected to lead to a clinically important change in the estimated effect of the [exposure]". We consider the following confounding domains as important for most or all studies since they have been shown to be associated with alcohol consumption and are prognostic factors for cognitive impairment: age, sex, socio-economic factors (especially education), smoking, and co-morbidities (especially diabetes, and obesity). Co-exposures will be assessed on a study-by-study basis.

For GRADE assessments it will be necessary to summarise risk of bias assessments across studies for each outcome. We will follow recent GRADE guidance for making these judgements (Schunemann 2018).These summary assessments of risk of bias will be used in determining the overall certainty of the body of evidence using GRADE, and the basis for each will be reported as footnotes to the summary of findings tables.

3.3.4 Measures of association

We expect that cognition will be measured using long ordinal scales, with varying measurement instruments used across studies. The standardised mean difference (SMD) will therefore be used as the measure of association. In the circumstance where results from multiple multivariable models are presented, we will extract associations from the most fully adjusted model, except in the case where an analysis adjusts for a possible intermediary along the causal

pathway (i.e. post baseline measures of prognostic factors (e.g. smoking, drug use, hypertension)) (Karahalios 2017). For ordinal measures of cognition that have been dichotomised and analysed as binary outcomes in the primary studies, we will re-express reported, or calculated, odds ratios as SMDs (Chinn 2000).

3.3.5 Unit of analysis issues

In this review, the unit of analysis issue is likely to arise from multiple estimates of association calculated for different levels of alcohol consumption within the same study. These estimates will be correlated since they will each compare against the same group of participants (e.g. 'never drinkers' in the comparisons 'high dose alcohol versus never drinkers' and 'moderate dose alcohol versus never drinkers'). Methods to adjust for the correlation between the estimated associations are described in the *Data synthesis* section.

3.3.6 Assessment of heterogeneity

We will assess heterogeneity visually by inspecting the overlap of confidence intervals on the forest plots, formally test for heterogeneity using the χ^2 test (using a significance level of $\alpha=0.1$), and quantify heterogeneity using the I^2 statistic (Higgins 2002).

3.3.7 Assessment of reporting biases

We will investigate the potential for small study effects (if there are at least 10 studies) using contour-enhanced funnel plots and formal statistical tests for funnel plot asymmetry. Contour-enhanced funnel plots aid in determining if funnel plot asymmetry is due to publication bias or other factors (Peters 2008). We will use the statistical test proposed by Egger et al to test for small-study effects (Egger 1997), and assess the potential impact of small-study effects using cumulative meta-analysis.

3.3.8 Data synthesis

Investigation of the association between levels and patterns of alcohol consumption and cognition

Separate comparisons will be set up by categories reflecting average levels of alcohol consumption (≥10 g/week and < 10 g/day; ≥10 g/day and < 20 g/day; ≥20 g/day and < 30 g/day; ≥30 g/day and < 40 g/day; ≥40 g/day and < 50 g/day; ≥ 50 g/day) versus never drinkers or very low level drinkers (zero to <1 g/day); patterns of alcohol consumption (e.g. occasional heavy drinking) versus 'never' drinkers or very low-level drinkers (zero to <10 g/week); and one pattern of alcohol consumption versus a different pattern (e.g. occasional heavy drinking compared to regular low risk drinking). The increments are based on an Australian standard drink (10 grams of alcohol), with lower levels based on the 2009 *Australian Guidelines*. If insufficient data are available, we will collapse categories. If sufficient data are available, these comparisons will be separated by sex. In studies that report alcohol consumption in different units (e.g. millilitres or standard drinks per days), we will convert these to grams per day using the relevant country's standards (Stockwell 2000). For studies that report alcohol consumption in categories that do not adequately fit those specified, the approach outlined under 'Investigation of dose response' will be applied to categorise average levels of intake.

Within each comparison (where possible), estimates of association between alcohol consumption and cognition will be combined using a random effects model with inverse-variance weighting. We will use the restricted maximum likelihood between-study variance estimator (Raudenbush 2009) with the Knapp and Hartung adjustment (Knapp 2003). Studies assessed as at a critical risk of bias will not be included in the meta-analysis (or considered in drawing conclusions). If a study contributes multiple estimates per comparison (e.g. alcohol consumption category), these estimates and their variances will first be averaged (Lopez-Lopez

In press). Or, if estimates are presented separately for non-overlapping subgroups, these will first be combined using a fixed effect meta-analysis.

If meta-analysis is not possible, and for studies that cannot be included in a meta-analysis, we will present available estimates of association (95% confidence intervals, p-values), along with risk of bias assessments and other exposure characteristics, in tables structured by comparison and subpopulation (e.g. sex, age categories). Forest plots will be used to visually depict estimates of association, even when these estimates are not meta-analysed.

These analyses will be undertaken using the suite of meta-analysis packages in the statistical package Stata and the `metafor` package in the statistical program R (Viechtbauer 2010).

Subgroup analyses

We will investigate if the associations between levels and patterns of alcohol consumption are modified by age, co-morbidities (any physical or mental health condition), drug taking (legal or otherwise), or a family history of alcohol use. However, we expect there will be limited ability to undertake these analyses since they rely upon studies being undertaken within a limited population or, separate reporting of associations by particular subpopulations within a study.

Sensitivity analyses

Many cohort studies may have only measured alcohol consumption at baseline. The risk of misclassification of intake in these studies is likely to be greater than those that measure alcohol intake over multiple time points (Jayasekara 2014). Given this, we plan to undertake sensitivity analyses (for the primary comparisons) removing studies that only have one measure of intake. Similarly, misclassification of former drinkers as very low-level drinkers may impact on observed effects. We plan to undertake sensitivity analyses (for the primary comparisons) limiting to studies that report data for ‘never’ drinkers.

Investigation of the dose response relationship between levels of alcohol consumption and cognition

An analysis will be undertaken to identify and characterise any dose-response relationship between levels of alcohol consumption and cognition, stratified by sex and age (where possible). For each study, the relationship between the SMD of cognition (compared with abstainers) and alcohol consumption will be modelled using a restricted cubic spline with three knots, accounting for correlation amongst the SMDs. The estimated study-specific dose-response coefficients and their covariance matrices will be combined using a random effects multivariate model (Crippa 2016). The between-study variance of the dose-response coefficients will be obtained using restricted maximum likelihood. Studies assessed as at a critical risk of bias will not be included in the dose-response analysis.

For each category of alcohol consumption, we will use the median or mean of alcohol consumption in grams per day when presented. When not presented, we will assign the midpoint of the category as the dose value. For the largest dose category, which may not have an upper bound, the assigned dose value will be calculated as the lower bound of the largest dose category plus the width of the previous (second-to-largest) category (Il'yasova 2005).

The combined does-response curve, along with 95% confidence interval, will be presented graphically and in tabular form (presenting predicted standardised mean differences of cognition for different alcohol consumption levels).

We will examine the robustness of the combined dose-response model to different locations of the knots and the number of knots.

The dose-response models will be fitted using the package `dosresmeta` in the statistical program R (Crippa 2016).

3.3.9 Summary of findings tables and assessment of certainty of the body of evidence

For each comparison and outcome domain, we will assess the certainty of the evidence using the GRADE approach. In accordance with the detailed GRADE guidance (Schunemann 2013, Schunemann 2018), the following domains will be assessed (as briefly summarised below) and a judgement made about whether there are serious, very serious or no concerns in relation to each domain.

1. Risk of bias. Based on the summary assessment across studies for each outcome reported for a comparison (see 'Risk of bias' section). The assessment will be based on guidance for ROBINS-I (Sterne 2016) and GRADE (Schunemann 2018).

2. Inconsistency. We will assess (1) whether there is heterogeneity in the observed effects across studies that suggests important differences in the effect of the exposure (based on point estimates, overlap in confidence intervals, and statistical tests of heterogeneity), and (2) whether this can be explained (e.g. by variance in effects across sub groups). Where a single study contributes data for a comparison and outcome, inconsistency will not be rated.

3. Imprecision. We will assess (1) whether interpretation of the upper and lower confidence limits leads to conflicting interpretations about whether the exposure has an important effect, and (2) whether the optimal information size is met (whether the total number of participants included in a meta-analysis is equal to or greater than the number required for an adequately powered study).

4. Indirectness. We will assess whether there are important differences between the review questions and the characteristics of included studies that may lead to important differences in the exposure effects (i.e. the applicability of the evidence). For example, studies with longer-term outcome follow up at multiple time points will be assessed as providing the most direct evidence

5. Publication bias. Our judgement of suspected publication bias will be based on assessment of reporting bias as described in section 3.3.8. Evidence of small study effects and the absence of a plausible alternative explanation for these effects indicates that publication bias should be suspected.

6. Upgrading domains (large effect size, dose response gradient, opposing plausible residual confounding). Recent GRADE guidance is that observational studies may start as high certainty evidence when ROBINS-I is used for risk of bias assessment (Schunemann 2018). Doing so alters the assessment of GRADE upgrading domains, since these domains examine the likelihood that any observed association could be explained by residual confounding, and are typically used to upgrade observational studies from low to moderate or high certainty. In line with one of the options presented in recent GRADE guidance, we will consider these GRADE domains when assessing confounding in ROBINS-I.

GRADEpro GDT software (www.gradepro.org) will be used to record decisions and derive an overall GRADE (high, moderate, low or very low) for the certainty of evidence for each outcome, using the GRADE rules in which observation studies assessed using ROBINS-I begin as 'high' certainty evidence (score=4) and can be downgraded by -1 for each domain with serious concerns or -2 for very serious concerns (Schunemann 2018).

Summary of findings tables (evidence profiles or evidence statements) will be prepared using the GRADEpro GDT software. For each comparison and outcome, the evidence profile will include estimates of the effects of alcohol exposure reported as standardised mean differences,

and the overall GRADE (rating of certainty). The evidence profiles will also include (1) the study design(s), number of studies and number of participants contributing data (the type and size of the evidence base), (2) our assessment of each of the domains (risk of bias, inconsistency, indirectness, imprecision, other considerations including publication bias), and (3) a plain language statement interpreting the evidence (clinical impact) for each comparison and outcome. Footnotes will be used to explain judgements made about downgrading or upgrading the rating of the certainty of the evidence.

4. References

Anstey, K. J., H. A. Mack and N. Cherbuin (2009). "Alcohol consumption as a risk factor for dementia and cognitive decline: meta-analysis of prospective studies." Am J Geriatr Psychiatry **17**(7): 542-555.

Australian Institute of Health and Welfare (2017). National Drug Strategy Household Survey 2016: detailed findings. Drug Statistics series no. 31. Cat. no. PHE 214. Canberra, AIHW.

Black, D. W. (2014). DSM-5 guidebook : Chapter 17 Neurocognitive Disorders. DSM-5 guidebook : the essential companion to the Diagnostic and statistical manual of mental disorders, fifth edition. J. E. Grant. Arlington, VA, American Psychiatric Publishing.

Chinn, S. (2000). "A simple method for converting an odds ratio to effect size for use in meta-analysis." Stat Med **19**(22): 3127-3131.

Crippa, A. and N. Orsini (2016). "Dose-response meta-analysis of differences in means." BMC Med Res Methodol **16**: 91.

Crippa, A. and N. Orsini (2016). "Multivariate Dose-Response Meta-Analysis: The dosresmeta R Package." 2016 **72**(Code Snippet 1): 15.

Davis, D. H., S. T. Creavin, A. Noel-Storr, T. J. Quinn, N. Smailagic, C. Hyde, C. Brayne, R. McShane and S. Cullum (2013). "Neuropsychological tests for the diagnosis of Alzheimer's disease dementia and other dementias: a generic protocol for cross-sectional and delayed-verification studies." Cochrane Database Syst Rev(3).

Egger, M., G. Davey Smith, M. Schneider and C. Minder (1997). "Bias in meta-analysis detected by a simple, graphical test." BMJ **315**(7109): 629-634.

Harrison, J. K., A. H. Noel-Storr, N. Demeyere, E. L. Reynish and T. J. Quinn (2016). "Outcomes measures in a decade of dementia and mild cognitive impairment trials." Alzheimers Res Ther **8**(1): 48.

Hartling, L., R. Featherstone, M. Nuspl, K. Shave, D. M. Dryden and B. Vandermeer (2017). "Grey literature in systematic reviews: a cross-sectional study of the contribution of non-English reports, unpublished studies and dissertations to the results of meta-analyses in child-relevant reviews." BMC Medical Research Methodology **17**(1): 64.

Higgins, J. and S. Green, Eds. (2011). Cochrane Handbook for Systematic Reviews of Interventions Version 5.1.0 [updated March 2011]. The Cochrane Collaboration.

Higgins, J. P. and S. G. Thompson (2002). "Quantifying heterogeneity in a meta-analysis." Stat Med **21**(11): 1539-1558.

Il'yasova, D., I. Hertz-Picciotto, U. Peters, J. A. Berlin and C. Poole (2005). "Choice of exposure scores for categorical regression in meta-analysis: a case study of a common problem." Cancer Causes Control **16**(4): 383-388.

Jayasekara, H., D. R. English, R. Room and R. J. MacInnis (2014). "Alcohol consumption over time and risk of death: a systematic review and meta-analysis." Am J Epidemiol **179**(9): 1049-1059.

Karahalios, A., D. R. English and J. A. Simpson (2017). "Change in body size and mortality: a systematic review and meta-analysis." Int J Epidemiol **46**(2): 526-546.

Knapp, G. and J. Hartung (2003). "Improved tests for a random effects meta-regression with a single covariate." Stat Med **22**(17): 2693-2710.

Knott, C. S., N. Coombs, E. Stamatakis and J. P. Biddulph (2015). "All cause mortality and the case for age specific alcohol consumption guidelines: pooled analyses of up to 10 population based cohorts." BMJ **350**: h384.

Livingston, G., A. Sommerlad, V. Orgeta, S. G. Costafreda, J. Huntley, D. Ames, C. Ballard, S. Banerjee, A. Burns, J. Cohen-Mansfield, C. Cooper, N. Fox, L. N. Gitlin, R. Howard, H. C. Kales, E. B. Larson, K. Ritchie, K. Rockwood, E. L. Sampson, Q. Samus, L. S. Schneider, G. Selbaek, L. Teri and N. Mukadam (2017). "Dementia prevention, intervention, and care." Lancet **390**(10113): 2673-2734.

Lopez-Lopez, J. A., M. J. Page, M. Lipsey and J. P. T. Higgins (In press). "Dealing with multiplicity of effect sizes in systematic reviews and meta-analyses." Research Synthesis Methods.

Matthews, F. E., B. C. Stephan, I. G. McKeith, J. Bond, C. Brayne and Medical Research Council Cognitive Function and Ageing Study (2008). "Two-year progression from mild cognitive impairment to dementia: to what extent do different definitions agree?" J Am Geriatr Soc **56**(8): 1424-1433.

Moher, D., L. Shamseer, M. Clarke, D. Ghersi, A. Liberati, M. Petticrew, P. Shekelle, L. A. Stewart and P.-P. Group (2015). "Preferred reporting items for systematic review and meta-analysis protocols (PRISMA-P) 2015 statement." Syst Rev **4**(1): 1.

Morrison, A., J. Polisena, D. Husereau, K. Moulton, M. Clark, M. Fiander, M. Mierzwinski-Urban, T. Clifford, B. Hutton and D. Rabb (2012). "The effect of English-language restriction on systematic review-based meta-analyses: a systematic review of empirical studies." Int J Technol Assess Health Care **28**(2): 138-144.

Naimi, T. S., T. Stockwell, J. Zhao, Z. Xuan, F. Dangardt, R. Saitz, W. Liang and T. Chikritzhs (2017). "Selection biases in observational studies affect associations between 'moderate' alcohol consumption and mortality." Addiction **112**(2): 207-214.

NHMRC Clinical Trials Centre (2017). Evaluating the evidence on the health effects of alcohol consumption: evidence evaluation report commission by the Office of the National Health and Medical Research Council Sydney, The University of Sydney.

Peters, J. L., A. J. Sutton, D. R. Jones, K. R. Abrams and L. Rushton (2008). "Contour-enhanced meta-analysis funnel plots help distinguish publication bias from other causes of asymmetry." J Clin Epidemiol **61**(10): 991-996.

Raudenbush, S. W. (2009). Analyzing effect sizes: Random-effects models. The Handbook of Research Synthesis and Meta-Analysis. H. Cooper, L. V. Hedges and J. C. Valentine, Russell Sage Foundation**:** 295-315.

Reeves, B., J. Deeks, J. Higgins and G. Wells (2011). Chapter 13: Including non-randomized studies. . Cochrane Handbook for Systematic Reviews of Interventions. Version 5.1.0 [updated March 2011]. Available from www.cochrane-handbook.org. J. Higgins and S. Green, The Cochrane Collaboration.

Reeves, B. C., G. A. Wells and H. Waddington (2017). "Quasi-experimental study designs series-paper 5: a checklist for classifying studies evaluating the effects on health interventions-a taxonomy without labels." J Clin Epidemiol **89**: 30-42.

Schunemann, H. J., J. Brozek, G. Guyatt and A. D. Oxman, Eds. (2013). Handbook for grading the quality of evidence and the strength of recommendations using the GRADE approach. Accessed 5 July 2016. Hamilton, Canada, McMaster University.

Schunemann, H. J., C. Cuello, E. A. Akl, R. A. Mustafa, J. J. Meerpohl, K. Thayer, R. L. Morgan, G. Gartlehner, R. Kunz, S. V. Katikireddi, J. Sterne, J. P. Higgins, G. Guyatt and G. W. Group (2018). "GRADE Guidelines: 18. How ROBINS-I and other tools to assess risk of bias in non-randomized studies should be used to rate the certainty of a body of evidence." J Clin Epidemiol.

Shamseer, L., D. Moher, M. Clarke, D. Ghersi, A. Liberati, M. Petticrew, P. Shekelle, L. A. Stewart and P.-P. Group (2015). "Preferred reporting items for systematic review and meta-analysis protocols (PRISMA-P) 2015: elaboration and explanation." BMJ **349**: g7647.

Sterne, J., J. Higgins, R. Elbers, B. Reeves and and the development group for ROBINS-I. (2016). "Risk Of Bias In Non-randomized Studies of Interventions (ROBINS-I): detailed guidance, updated 12 October 2016. Available from http://www.riskofbias.info Accessed 25 March 2018."

Sterne, J. A., M. A. Hernan, B. C. Reeves, J. Savovic, N. D. Berkman, M. Viswanathan, D. Henry, D. G. Altman, M. T. Ansari, I. Boutron, J. R. Carpenter, A. W. Chan, R. Churchill, J. J. Deeks, A. Hrobjartsson, J. Kirkham, P. Juni, Y. K. Loke, T. D. Pigott, C. R. Ramsay, D. Regidor, H. R. Rothstein, L. Sandhu, P. L. Santaguida, H. J. Schunemann, B. Shea, I. Shrier, P. Tugwell, L. Turner, J. C. Valentine, H. Waddington, E. Waters, G. A. Wells, P. F. Whiting and J. P. Higgins (2016). "ROBINS-I: a tool for assessing risk of bias in non-randomised studies of interventions." BMJ **355**: i4919.

Stockwell, T. and T. Chikritzhs (2000). International Guide for Monitoring Alcohol Consumption and Related Harm. Geneva, Switzerland, Department of Mental Health and Substance Dependence, Noncommunicable Diseases and Mental Health Cluster, World Health Organization.

Topiwala, A., C. L. Allan, V. Valkanova, E. Zsoldos, N. Filippini, C. Sexton, A. Mahmood, P. Fooks, A. Singh-Manoux, C. E. Mackay, M. Kivimäki and K. P. Ebmeier (2017). "Moderate alcohol consumption as risk factor for adverse brain outcomes and cognitive decline: Longitudinal cohort study." BMJ (Online) **357**.

Viechtbauer, W. (2010). "Conducting Meta-Analyses in R with the metafor." Journal of Statistical Software **36**(3).

Viswanathan, M., N. D. Berkman, D. M. Dryden and L. Hartling (2013). AHRQ Methods for Effective Health Care. Assessing Risk of Bias and Confounding in Observational Studies of Interventions or Exposures: Further Development of the RTI Item Bank. Rockville (MD), Agency for Healthcare Research and Quality (US).

Winblad, B., K. Palmer, M. Kivipelto, V. Jelic, L. Fratiglioni, L. O. Wahlund, A. Nordberg, L. Backman, M. Albert, O. Almkvist, H. Arai, H. Basun, K. Blennow, M. de Leon, C. DeCarli, T. Erkinjuntti, E. Giacobini, C. Graff, J. Hardy, C. Jack, A. Jorm, K. Ritchie, C. van Duijn, P. Visser and R. C. Petersen (2004). "Mild cognitive impairment--beyond controversies, towards a consensus: report of the International Working Group on Mild Cognitive Impairment." J Intern Med **256**(3): 240-246.

Xu, W., H. Wang, Y. Wan, C. Tan, J. Li, L. Tan and J.-T. Yu (2017). "Alcohol consumption and dementia risk: a dose-response meta-analysis of prospective studies." European journal of epidemiology **32**(1): 31-42.

Appendix: Database search strategies

Search strategy for Ovid Embase

#	Search statement
1	exp drinking behavior/
2	exp alcoholism/
3	exp alcoholic beverage/
4	(alcohol$ or drinking or wine).tw.
5	or/1-4
6	exp dementia/
7	exp cognitive defect/
8	(dementia or cognition or cognitive or neurocognit$ or neuro-cognit$ or alzheimer$).tw.
9	or/6-8
10	exp cohort analysis/
11	exp case control study/
12	exp risk factor/
13	exp longitudinal study/
14	(cohort$ or longitudinal or follow-up or "follow up").tw.
15	(case$ adj3 control$).tw.
16	or/10-15
17	5 and 9 and 16
18	exp animal/ not human/
19	17 not 18
20	limit 19 to yr="2007 –Current"

Search strategy for Ovid PsycINFO

#	Search statement
1	exp alcoholism/
2	exp alcohol drinking patterns/
3	exp drinking behavior/
4	exp binge drinking/

5	exp alcohol abuse/
6	exp alcoholic beverages/
7	(alcohol$ or drinking or wine).tw.
8	or/1-7
9	exp dementia/
10	exp cognitive impairment/
11	(dementia or cognition or cognitive or neurocognit$ or neuro-cognit$ or alzheimer$).tw.
12	or/9-11
13	exp risk factors/
14	exp longitudinal studies/
15	exp followup studies/
16	(cohort$ or longitudinal or follow-up or “follow up”).tw.
17	(case$ adj3 control$).tw.
18	or/13-17
19	8 and 12 and 18
20	limit 19 to yr=”2007 –Current”
