# Supplementary material for: Long-term effects of alcohol consumption on cognitive function: a systematic review and dose-response analysis of evidence published between 2007 and 2018
Source: Syst Rev. 2020 Feb 13;9:33. doi: 10.1186/s13643-019-1220-4 (PMC7020517; doi:10.1186/s13643-019-1220-4)
Supplement: Supplementary file 2 — Additional file 2. Appendices 1 to 10 [file 13643_2019_1220_MOESM2_ESM.pdf]

Contents

Appendix 1. Changes to protocol 2

Appendix 2. Database search strategies 3

Appendix 3. References to included studies 6

Appendix 4. Characteristics of included studies 9

Table 4.1. Characteristics of studies that examined the effects of different patterns of alcohol consumption* 9

Table 4.2. Funding sources, potential conflicts of interest, and ethics approval for studies that examined different levels of alcohol consumption 16

Appendix 5. Characteristics of excluded studies – reasons for exclusion 18

Appendix 6. Risk of bias assessment of included studies examining the effects of different levels of alcohol 32

Arntzen 2010 32

Downer 2015 33

Hassing 2018 34

Heffernan 2016 35

Hogenkamp 2014 36

Horvat 2015 37

Kesse-Guyot 2012 38

Kitamura 2017 39

Lang 2007 40

McGuire 2007 41

Piumatti 2018 42

Richard 2017 43

Sabia 2011 44

Sabia 2014 45

Samieri 2013 46

Solfrizzi 2007 47

Stott 2007 48

Wardzala 2018 49

Appendix 7. Data manipulation for each study 50

Appendix 8. Results from sensitivity analyses 53

Appendix 9. Reasons for exclusion of studies from the dose-response analyses 56

Appendix 10. Alphabetical reference list of all studies excluded following full text review57

Appendix 11. Abbreviations 69

References 70

Appendix 1. Changes to protocol

Section	Protocol	Review
Review objectives	We planned to examine the effects of different patterns of alcohol consumption on cognition.	The size and complexity of the review necessitated several changes to scope to ensure review completion within resources and required timeframe. For this reason, we limited our review of studies examining patterns to a summary and synthesis of study characteristics. For these studies, risk of bias was not assessed and results were not extracted or reported.
Eligibility criteria	No changes, but some criteria were revised to clarify study eligibility.	Criteria for study design were refined to clarify the definition of prospective alcohol exposure assessment and to explicitly exclude studies that used concomitant measures of alcohol and follow-up cognition in analyses (despite having longitudinal data that would have be suitable for analyses based on prospective alcohol assessment).
Study selection	We planned to have two reviewers independently screen citations and full text.	The large number of studies retrieved for full text screening, together with the complexity of screening, meant that we were unable to independently double screen all studies. A samples of studies was double screened until concordance in screening was achieved. All included studies and any for which screening decisions were uncertain were double screened.
Summary and synthesis	We planned to extract, summarise and undertake synthesis of results from studies examining different patterns of consumption.	We extracted and summarised information about the characteristics of studies that examined the effect of different patterns of alcohol consumption, but did not extract or perform any summary or synthesis of results.<br>This was a decision taken to manage the size of the review, based on the large number of studies initially contributing to the question about levels of alcohol consumption.
Synthesis	We planned to undertake meta-analyses of pairwise comparisons of different levels of alcohol consumption versus never drinkers or very low level drinkers (zero to <1 g/day).	We did not undertake these analyses since all studies that contributed data suitable for synthesis were able to be included in the dose-response analyses. The dose-response analyses provide a more complete understanding of the relationship between alcohol consumption and the size of the SMDs, since all data are modelled in a single synthesis. Further, from these models, SMDs at any level of alcohol consumption (within the observed range) can be predicted.

Appendix 2. Database search strategies

Search strategy for systematic reviews (Embase and MEDLINE)

Embase Classic+Embase 1947 to 2018 February 12 and Ovid MEDLINE(R) Epub Ahead of Print, In-Process & Other Non-Indexed Citations, Ovid MEDLINE(R) Daily, Ovid MEDLINE and Versions(R) 1946 to February 07, 2018

#	Search Statement	Results
1	exp Alcohol drinking/	106261
2	exp Alcoholic Beverages/	45653
3	Alcoholic intoxication/	18410
4	Alcoholism/	193695
5	exp Alcohol-Related Disorders/	228502
6	(alcohol$ or drinking or wine).tw.	876461
7	or/1-6	955932
8	exp Dementia/	451881
9	exp Cognitive Dysfunction/	419113
10	(dementia or cognition or cognitive).tw.	868483
11	or/8-10	1134251
12	(meta-analysis or review).pt.	4706858
13	(systematic$ and (review$ or overview$)).tw.	342078
14	(meta?analy$ or meta analy$).tw.	281997
15	or/12-14	4957423
16	7 and 11 and 15	4677
17	(2017$ or 2018$).dc.[1]	2004172
18	16 and 17	213
19	(2017$ or 2018$).dt.[1]	1353297
20	16 and 19	107
21	18 or 20	320
22	remove duplicates from 21	251

[1] added to EMBASE/MEDLINE in 2017-2018, irrespective of year of publication

Search strategy for primary studies (MEDLINE)

Ovid MEDLINE(R) Epub Ahead of Print, In-Process & Other Non-Indexed Citations, Ovid MEDLINE(R) Daily, Ovid MEDLINE and Versions(R) <1946 to April 04 2018>

#	Search Statement	Results
1	exp Alcohol drinking/	61992
2	exp Alcohol-Related Disorders/	106648
3	exp Alcoholic Beverages/	17829
4	(alcohol$ or drinking or wine).tw.	367876
5	or/1-4	406690
6	exp Dementia/	144901
7	exp Cognition Disorders/	79912
8	(dementia or cognition or cognitive or neurocognit$ or neuro-cognit$ or alzheimer$).tw.	448982
9	6 or 7 or 8	494437
10	exp Cohort Studies/	1725961
11	Controlled Clinical Trial.pt.	92290
12	exp Case-Control Studies/	905866

13	Risk Factors/	716307
14	(cohort$ or longitudinal or follow-up or "follow up").tw.	1327253
15	(case$ adj3 control$).tw.	143218
16	or/10-15	3104279
17	5 and 9 and 16	3733
18	Animals/ not Humans/	4407379
19	17 not 18	3708
20	limit 19 to yr="2007 -Current"	2252

Search strategy for primary studies (Embase)

Embase Classic+Embase <1947 to 2018 April 06>

#	Search Statement	Results
1	exp drinking behavior/	45554
2	exp alcoholism/	123560
3	exp alcoholic beverage/	28377
4	(alcohol$ or drinking or wine).tw.	519135
5	or/1-4	558699
6	exp dementia/	320588
7	exp cognitive defect/	426609
8	(dementia or cognition or cognitive or neurocognit$ or neuro-cognit$ or alzheimer$).tw.	623351
9	or/6-8	745932
10	exp cohort analysis/	361560
11	exp case control study/	142011
12	exp risk factor/	866500
13	exp longitudinal study/	111824
14	(cohort$ or longitudinal or follow-up or "follow up").tw.	2050163
15	(case$ adj3 control$).tw.	193423
16	or/10-15	2960836
17	5 and 9 and 16	4918
18	exp animal/ not human/	5173603
19	17 not 18	4825
20	limit 19 to yr="2007 -Current"	3659

Search strategy for primary studies (PsycINFO)

PsycINFO <1806 to April Week 1 2018>

#	Search Statement	Results
1	exp alcoholism/	29323
2	exp alcohol drinking patterns/	62837
3	exp drinking behavior/	68306
4	exp binge drinking/	2067
5	exp alcohol abuse/	45823
6	exp alcoholic beverages/	2638
7	(alcohol$ or drinking or wine).tw.	133581
8	or/1-7	136953
9	exp dementia/	69024

10	exp cognitive impairment/	32035
11	(dementia or cognition or cognitive or neurocognit$ or neuro-cognit$ or alzheimer$).tw.	480326
12	or/9-11	484443
13	exp risk factors/	70031
14	exp longitudinal studies/	15920
15	exp followup studies/	12359
16	(cohort$ or longitudinal or follow-up or "follow up").tw.	241129
17	(case$ adj3 control$).tw.	13725
18	or/13-17	314203
19	8 and 12 and 18	1927
20	limit 19 to yr="2007 -Current"	1292

Appendix 3. References to included studies

* paper from which data was extracted for studies with multiple eligible reports

Arntzen 2010

Arntzen KA, Schirmer H, Wilsgaard T, Mathiesen EB: Moderate wine consumption is associated with better cognitive test results: a 7 year follow up of 5033 subjects in the Tromso Study. Acta Neurologica Scandinavica 2010, Supplementum:23-29.

Boelema 2015

Boelema SR, Harakeh Z, van Zandvoort MJE, Reijneveld SA, Verhulst FC, Ormel J, Vollebergh WAM: Adolescent heavy drinking does not affect maturation of basic executive functioning: Longitudinal findings from the TRAILS study. PLoS ONE [Electronic Resource] 2015, 10:e0139186.

Carbia 2017

Carbia C, Cadaveira F, Lopez-Caneda E, Caamano-Isorna F, Rodriguez Holguin S, Corral M: Working memory over a six-year period in young binge drinkers. Alcohol 2017, 61:17-23.

Carbia 2018

Carbia C, Corral M, Doallo S, Caamano-Isorna F: The dual-process model in young adults with a consistent binge drinking trajectory into adulthood. Drug & Alcohol Dependence 2018, 186:113-119.

Downer 2015

Downer B, Jiang Y, Zanjani F, Fardo D: Effects of alcohol consumption on cognition and regional brain volumes among older adults. American Journal of Alzheimer's Disease & Other Dementias 2015, 30:364-374.

*Downer B, Zanjani F, Fardo DW: The relationship between midlife and late life alcohol consumption, APOE e4 and the decline in learning and memory among older adults. Alcohol & Alcoholism 2014, 49:17-22.

Gross 2011

Gross AL, Rebok GW, Ford DE, Chu AY, Gallo JJ, Liang K-Y, Meoni LA, Shihab HM, Wang N-Y, Klag MJ: Alcohol consumption and domain-specific cognitive function in older adults: longitudinal data from the Johns Hopkins Precursors Study. Journals of Gerontology Series B-Psychological Sciences & Social Sciences 2011, 66:39-47.

Hassing 2018

Hassing LB: Light alcohol consumption does not protect cognitive function: A longitudinal prospective study. Frontiers in Aging Neuroscience 2018, 10:81.

Heffernan 2016

Heffernan M, Mather KA, Xu J, Assareh AA, Kochan NA, Reppermund S, Draper B, Trollor JN, Sachdev P, Brodaty H: Alcohol consumption and incident dementia: Evidence from the Sydney Memory and Ageing Study. Journal of Alzheimer's Disease 2016, 52:529-538.

Hoang 2014

Hoang TD, Byers AL, Barnes DE, Yaffe K: Alcohol consumption patterns and cognitive impairment in older women. American Journal of Geriatric Psychiatry 2014, 22:1663-1667.

Hogenkamp 2014

Hogenkamp PS, Benedict C, Sjogren P, Kilander L, Lind L, Schioth HB: Late-life alcohol consumption and cognitive function in elderly men. Age 2014, 36:243-249.

Horvat 2015

Horvat P, Richards M, Kubinova R, Pajak A, Malyutina S, Shishkin S, Pikhart H, Peasey A, Marmot MG, Singh-Manoux A, Bobak M: Alcohol consumption, drinking patterns, and cognitive function in older Eastern European adults. Neurology 2015, 84:287-295.

Jacobus 2013

Jacobus J, Squeglia LM, Bava S, Tapert SF: White matter characterization of adolescent binge drinking with and without co-occurring marijuana use: a 3-year investigation. Psychiatry Research 2013, 214:374-381.

Kesse-Guyot 2014

*Kesse-Guyot E, Andreeva VA, Jeandel C, Ferry M, Touvier M, Hercberg S, Galan P: Alcohol consumption in midlife and cognitive performance assessed 13 years later in the SU.VI.MAX 2 cohort. PLoS ONE [Electronic Resource] 2012, 7:e52311.

Kesse-Guyot E, Andreeva VA, Lassale C, Hercberg S, Galan P: Clustering of midlife lifestyle behaviors and subsequent cognitive function: a longitudinal study. American Journal of Public Health 2014, 104:e170-177.

Kitamura 2017

Kitamura K, Watanabe Y, Nakamura K, Takahashi A, Takachi R, Oshiki R, Kobayashi R, Saito T, Tsugane S, Sasaki A: Weight loss from 20 years of age is associated with cognitive impairment in middle-aged and elderly individuals. PLoS ONE [Electronic Resource] 2017, 12:e0185960.

Lang 2007

Herring D, Paulson D: Moderate alcohol use and apolipoprotein E-4 (ApoE-4): Independent effects on cognitive outcomes in later life. Journal of Clinical & Experimental Neuropsychology: Official Journal of the International Neuropsychological Society 2018, 40:326-337.

*Lang I, Guralnik J, Wallace RB, Melzer D: What level of alcohol consumption is hazardous for older people? Functioning and mortality in U.S. and English national cohorts. Journal of the American Geriatrics Society 2007a, 55:49-57.

Lang I, Wallace RB, Huppert FA, Melzer D: Moderate Alcohol consumption in older adults is associated with better cognition and well-being than abstinence. Age & Ageing 2007b, 36:256-261.

McGuire 2007

McGuire LC, Ajani UA, Ford ES: Cognitive functioning in late life: the impact of moderate alcohol consumption. Annals of Epidemiology 2007, 17:93-99.

Mota 2013

Mota N, Parada M, Crego A, Doallo S, Caamano-Isorna F, Rodriguez Holguin S, Cadaveira F, Corral M: Binge drinking trajectory and neuropsychological functioning among university students: a longitudinal study. Drug & Alcohol Dependence 2013, 133:108-114.

Ngandu 2007

Ngandu T, Helkala E-L, Soininen H, Winblad B, Tuomilehto J, Nissinen A, Kivipelto M: Alcohol drinking and cognitive functions: findings from the Cardiovascular Risk Factors Aging and Dementia (CAIDE) Study. Dementia & Geriatric Cognitive Disorders 2007, 23:140-149.

Nguyen-Louie 2017

Nguyen-Louie TT, Matt GE, Jacobus J, Li I, Cota C, Castro N, Tapert SF: Earlier alcohol use onset predicts poorer neuropsychological functioning in young adults. Alcoholism: Clinical and Experimental Research 2017, 41:2082-2092.

Piumatti 2018

Piumatti G, Moore SC, Berridge DM, Sarkar C, Gallacher J: The relationship between alcohol use and long-term cognitive decline in middle and late life: a longitudinal analysis using UK Biobank. Journal of Public Health 2018:09.

Richard 2017

Richard EL, Kritz-Silverstein D, Laughlin GA, Fung TT, Barrett-Connor E, McEvoy LK: Alcohol Intake and Cognitively Healthy Longevity in Community-Dwelling Adults: The Rancho Bernardo Study. Journal of Alzheimer's Disease 2017, 59:803-814.

Sabia 2011

Sabia S, Gueguen A, Berr C, Berkman L, Ankri J, Goldberg M, Zins M, Singh-Manoux A: High alcohol consumption in middle-aged adults is associated with poorer cognitive performance only in the low socio-economic group. Results from the GAZEL cohort study. Addiction 2011, 106:93-101.

Sabia 2014

Sabia S, Nabi H, Kivimaki M, Shipley MJ, Marmot MG, Singh-Manoux A: Health behaviors from early to late midlife as predictors of cognitive function: The Whitehall II study. American Journal of Epidemiology 2009, 170:428-437.

* Sabia S, Elbaz A, Britton A, Bell S, Dugravot A, Shipley M, Kivimaki M, Singh-Manoux A: Alcohol consumption and cognitive decline in early old age. Neurology 2014, 82:332-339.

Topiwala A, Allan CL, Valkanova V, Zsoldos E, Filippini N, Sexton C, Mahmood A, Fooks P, Singh-Manoux A, Mackay CE, et al: Moderate alcohol consumption as risk factor for adverse brain outcomes and cognitive decline: longitudinal cohort study. BMJ 2017, 357:j2353.

Samieri 2013a

Samieri C, Grodstein F, Rosner BA, Kang JH, Cook NR, Manson JE, Buring JE, Willett WC, Okereke OI: Mediterranean diet and cognitive function in older age. Epidemiology 2013, 24:490-499.

Solfrizzi 2007

Solfrizzi V, D'Introno A, Colacicco AM, Capurso C, Del Parigi A, Baldassarre G, Scapicchio P, Scafato E, Amodio M, Capurso A, et al: Alcohol consumption, mild cognitive impairment, and progression to dementia. Neurology 2007, 68:1790-1799.

Stott 2008

Stott DJ, Falconer A, Kerr GD, Murray HM, Trompet S, Westendorp RGJ, Buckley B, de Craen AJM, Sattar N, Ford I: Does low to moderate alcohol intake protect against cognitive decline in older people? Journal of the American Geriatrics Society 2008, 56:2217-2224.

Wardzala 2018

Wardzala C, Murchison C, Loftis JM, Schenning KJ, Mattek N, Woltjer R, Kaye J, Quinn JF, Wilhelm CJ: Sex differences in the association of alcohol with cognitive decline and brain pathology in a cohort of octogenarians. Psychopharmacology 2018, 235:761-770.

Appendix 4. Characteristics of included studies

The following tables provide supplementary information to that reported in the Systematic review report. Table 4.1 summarises the PECO and study design characteristics of studies that examined different patterns of alcohol consumption. Table 4.2 summarises funding sources and potential conflict of interest for studies that examined different levels of alcohol consumption.

*Table 4.1. Characteristics of studies that examined the effects of different patterns of alcohol consumption**

Study details	Sample	Alcohol exposure categories (patterns)	Details of the included article	Study dates
**Boelema 2015**<br>Netherlands<br>Cohort name: Tracking Adolescents' Individual Lives Survey (TRAILS)	Based on 2230 adolescents (50.8% female) aged 10-12 at T0 and 18-20 at final follow-up (T3)<br><br>Substudy of original large, ongoing cohort	Group assignment according to consumption (average quantity / frequency since last follow-up) at T2 & T3<br>**Non-drinkers** 'did not consume alcohol' at T2 & T3<br>**Light-drinkers** < '6 glasses on a weekend day for boys and 5 glasses for girls' at T2 & T3<br>**Infrequent heavy drinkers** ≥ '6 glasses on a weekend day for boys and 5 glasses for girls' at T2 OR T3<br>**Increased heavy drinkers ≥ '**6 glasses on a weekend day for boys and 5 glasses for girls' and drinking regularly (last month prevalence ≥ 4 times drinking, i.e., weekly drinking) at T3<br>**Decreased heavy drinkers** ≥ **'**6 glasses on a weekend day for boys and 5 glasses for girls' and drinking regularly (last month prevalence ≥ 4 times drinking, i.e., weekly drinking) at T2<br>**Chronic heavy drinkers** ≥ '6 glasses on a weekend day for boys and 5 glasses for girls' and drinking regularly (last month prevalence ≥ 4 times drinking, i.e., weekly drinking) at T2 AND T3<br><br>Grams per glass = 10 grams	Observational cohort reporting associations between patterns (quantity / frequency and change in consumption over time) of alcohol consumption and cognitive function among adolescents.<br>Inclusion criteria: children living in 5 municipalities in the North of the Netherlands; born between 1 October 1989 and 30 September 1990 in 2 of the municipalities, and between 1 October 1990 and 30 September 1991 in the other 3. Participants recruited when age 10-12.<br>Exclusion criteria: serious health or language problems.<br>Alcohol ascertainment: Current (quantity, change over time): self-report questionnaire asking about frequency of weekly consumption (how many days do you drink alcohol) and typical intake (how much alcohol (glasses, cans, bottles)). Recall: since last follow-up (2-3 years). Lifetime: measured. Problem drinking: not measured.<br>Cognitive function: Specific cognitive domains (4 outcomes). Four basic executive functions (Amsterdam Neuropsychological Tasks): inhibition, working memory, shift attention, and sustained attention. Change scores calculated by computing z-scores for T0 and T3 measures and subsequently subtracting these scores from each other (T0-T3).	**Study period:** 2001-2010<br>**Alcohol exposure**: multiple assessments - baseline and then 3 measures at ~ 2-3 year intervals (T0-T3: 2001-2010) (only T2-T3 used in the analysis)<br>**Outcome measures**: baseline and final follow-up (T0 & T3: 2001 & 2010)<br>**Length of outcome follow-up**: ~ 8 years
**Carbia 2017**<br>Spain<br>Cohort name: not provided	Based on 155[1] university students (50.1% female) aged 18-19 years at baseline and 24-25 at final follow-up<br>1. 155 at baseline, 40 at final follow-up.	The classification criteria were based on responses to two questions: the third item of the AUDIT, and one question related to the rate of consumption (drinks per hour). Binge drinking (BD): ≥ 6 drinks per occasion (monthly or weekly) at a rate of ≥3 drinks per hour (heaviest consumption); Non binge drinking (non-BD): < 6	Observational cohort reporting on the relationship between binge drinking trajectory and working memory in Caucasian university students recruited from different faculties of the University of Santiago de Compostela.<br>Inclusion criteria: healthy university students with no other relevant risk factors, such as psychiatric comorbidity or family history of alcoholism.<br>Exclusion criteria: consumption of any other drugs (e.g., opiates, hallucinogens, cocaine, amphetamines, or medically prescribed psychoactive substances), except nicotine and cannabis; diagnosis of alcohol-use disorders; severe non-	**Study period**: not reported<br>**Alcohol exposure**: multiple assessments - baseline and then 3 measures at ~ 2 year intervals (T0-T3)

Study details	Sample	Alcohol exposure categories (patterns)	Details of the included article	Study dates
	Probably same sample involved in Mota 2013	drinks per occasion (monthly or weekly) at a rate of ≤ 2 drinks per hour (heaviest consumption)<br>Patterns used in the analyses:<br>**Ex-binge drinkers** had abandoned the BD pattern at the 2nd, 3rd, or 4th evaluation<br>**Stable non-binge drinkers** maintained non-binge drinking throughout follow-up<br>**Stable binge drinkers** maintained non-binge drinking throughout follow-up	corrected motor or sensory deficits; family history of major mental disorder; history of alcoholism in first-and second-degree relatives; history of psychopathology (DSM-IV-TR), such as attention-deficit hyperactivity disorder; conduct disorder or previous diagnosis of depression or anxiety; and current psychopathological symptoms as assessed by the Symptom Checklist-90-R (SCL-90-R). Abstainers were not included in the study.<br><u>Alcohol ascertainment</u>: The questionnaire included the Alcohol Use Disorders Identification Test (AUDIT) and questions related to alcohol use (rate of consumption, age of onset, etc.). Recall: no information. Lifetime: not measured. Problem drinking: not specifically assessed, but AUDIT (a test for alcohol disorders) used and 'alcohol-use disorder' was an exclusion criteria<br><u>Cognitive function</u>: specific cognitive domains (1 outcome; 16 measures) Executive function - working memory was assessed using the Self-Ordered Pointing Test, abstract design version (SOPT). The score was categorized into four values ranging from -1 to 1, to deal with negative values.	**Outcome measure**: multiple assessments - baseline and then 3 measures at ~ 2 year intervals (T0-T3)<br>**Length of outcome follow-up**: ~6 years
**Carbia 2018**<br>Spain<br>Cohort name: the Compostela Cohort	Based on 63 university students (49.2% female) aged 18 years at baseline (point of first alcohol measure) and 29 years when cognition measured<br><br>Substudy of original large cohort	**Non-binge drinkers** continuous low alcohol consumption trajectory (AUDIT-C [first three questions] score <4 in all assessments)<br>**Binge drinkers** previous high alcohol consumption trajectory (AUDIT-C [first three questions] score ≥4 at least in three assessments)	Observational cohort examining the relationship between binge drinking and the reflective system (executive functions) in young Caucasian adults recruited from the University of Santiago de Compostela.<br><u>Inclusion criteria</u>: continuous high alcohol consumption trajectories or continuous low alcohol consumption trajectories at the final follow-up. On the day of testing, the subjects self-reported abstinence from alcohol for at least 48 hr and slept well the night before.<br><u>Exclusion criteria</u>: severe motor or sensory deficits; history of any neurological or psychiatric disorders; medication that affects cognitive functions; and family history of alcoholism in first degree relatives.<br><u>Alcohol ascertainment</u>: measured with the AUDIT: AUDIT total scores analysed and AUDIT-C used to classify participants as binge drinkers or non-binge drinkers. Recall: no information. Lifetime: not measured. Problem drinking: AUDIT used: none of the participants scored > 20 (usually considered a cut-off for alcohol dependence).<br><u>Cognitive function</u>: specific cognitive domains (2 domains, 3 outcomes). Executive function (working memory) was measured with the Self-Ordered Pointing Test (SOPT); cognitive flexibility was measured with the Verbal fluency task and with the Trail Making Test (TMT).Test results appear to be analysed as raw scores.	**Study period**: 2005-2016<br>**Alcohol exposure**: multiple assessments - baseline and then 5 measures at ~ 2-3 year intervals (T0-T5: 2005-2016)<br>**Outcome measure**: single assessment at final follow-up (T5: 2016)<br>**Length of outcome follow-up**: ~11 years
**Gross 2011**<br>United States<br>Cohort name: Johns Hopkins Precursors Study	Based on 588 medical graduates (8% female) aged 55 years at baseline (point of first alcohol measure)	**Frequency** (last year): ≤ 2 times per month (includes those who reported drinking 'rarely), 1-2 times per week, 3-4 times per week, daily or almost daily (<u>referent</u>).	Observational cohort reporting associations between patterns (frequency, change in consumption over time) of alcohol consumption and cognitive function among older adults.<br><u>Inclusion criteria</u>: medical students who graduated from The Johns Hopkins Medical School between 1948 and 1964, and were alive and consented to cognitive testing over the telephone in 2005.	**Study period**: 1986-2005<br>**Alcohol exposure**: multiple assessments - baseline and then 4 measures at ~ 3-4 year

Study details	Sample	Alcohol exposure categories (patterns)	Details of the included article	Study dates
	and 60-86 years when cognition measured.<br><br>Substudy of original cohort.	**Change in average consumption** based on average daily intake measured at T0-T5 (recall at each time point: past year)<br>**Problem drinking.** CAGE questionnaire score <2 (referent), score 2+	Exclusion criteria: none reported<br>Alcohol ascertainment: Current (frequency, change over time): self-report questionnaire asking about frequency of consumption ("daily or almost every day", "3–4×/week", "1–2×/week", "1–2×/month", or "rarely") and typical weekly intake (by type of alcohol; grams of alcohol per drink not reported). Recall: last 12 months. Lifetime: not measured. Problem drinking: CAGE questionnaire.<br>Cognitive function: Global cognitive function (telephone interview for cognitive status (TICS)); specific cognitive domains (4 outcomes). Learning and memory (Hopkins verbal learning test), language (verbal fluency tests, number animals named; number of F, A, and S words), complex attention (Brief Test of Attention). Test results appear to be analysed as raw scores. Higher scores = better cognition.	intervals (T0-T5: 1986-2003)<br>**Outcome measures**: single assessment at follow-up (T6: 2005)<br>**Length of outcome follow-up**: ~ 2-19 years (depending on analysis)
**Hoang 2014**<br>United States<br>Cohort name: Women Cognitive Impairment Study of Exceptional Aging (WISE)	Based on 1309 community-dwelling women aged ≥65 years at baseline (point of first alcohol measure) and ≥85 years when cognition measured<br><br>An ancillary study of the Study of Osteoporotic Fractures (SOF)	The study measured average level of consumption per week (see below), but the analysis was based on average change in amount consumed over 16 year period<br>**Average decrease** (referent): between 0 to 0.5 drinks per week<br>**Decrease** average decrease >0.5 drinks per week<br>**Increase** average increase >0 drinks per week<br><br>Average level of consumption at each measurement point was categorised as:<br>Non-drinker: 0 drinks per week<br>Light: >0 to <3 drinks per week<br>Moderate: ≥ 3 to ≤ 7 drinks per week<br>Heavy: > 7 drinks per week<br>Possible binge drinking: >4 drinks on one occasion.	Observational cohort reporting long-term relationship between changes in alcohol use and cognitive impairment in older community-dwelling American women recruited from population-based listings<br>Inclusion criteria: aged 65 years and older at baseline.<br>Exclusion criteria: a previous diagnosis of dementia at baseline; less than 2 completed visits; no cognitive evaluation; incomplete alcohol use data.<br>Alcohol ascertainment: Current (quantity, change over time): self-report questionnaire asking about the frequency and amount. Recall: past 30 days. Lifetime: not measured. Problem drinking: possible binge drinking.<br>Cognitive function: Clinically significant cognitive impairment (mild cognitive impairment and dementia) (2 outcomes). Outcomes determined using 2 step process. First step: screening process using (1) Modified Mini-Mental State Examination; (2) California Verbal Learning Test delayed recall; (3) Informant Questionnaire on Cognitive Decline in the Elderly; (4) previous dementia diagnosis; or (5) nursing home residence, to identify at risk women. Second step adjudication of cognitive status using panel of clinical experts: diagnosis of dementia was made based on DSM-IV criteria and mild cognitive impairment was diagnosed using a modified Petersen.	**Study period:** 1986-2008<br>**Alcohol exposure:** multiple assessments - baseline and then at 2-6 year intervals (T0-T4: 1986-2004)<br>**Outcome measures**: baseline and final follow-up (T0 &T5: 1986 & 2008)<br>**Length of outcome follow-up**: 20 years from first alcohol measurement (T0)
**Horvat 2015**† Eastern Europe (Russia, Poland, Czech Republic)<br>Cohort name: HAPIEE (Health, Alcohol, and Psychosocial Factors in Eastern	Based on 28,947 men and women (54.7% female) aged 45-69 years at point of first alcohol measure (T0).	**Frequency** (last year): never, < 1 time per month (referent), 1-3 times per month, 1-4 times per week, ≥ 5 times per week)<br>**Binge drinking** (last year): non-drinker (0 grams per day), non-binger (on one occasion at least monthly: <60 grams for women, <100 grams per day for men; referent), binge drinker (on one occasion at least monthly: ≥60 grams for women, ≥100 grams per day for men).	Observational cohort examining associations between different levels and patterns (frequency, binge, problem drinking) of alcohol consumption and cognitive function in older adults.<br>Inclusion criteria: Eligible participants were aged 45-69 years (T0), randomly selected from population registers and electoral lists.<br>Exclusion criteria: none reported.<br>Alcohol ascertainment: Current: self-report graduated frequency questionnaire (GFQ) asking about frequency of consumption and number of drinks (by alcohol	**Study period**: 2002-2008<br>**Alcohol exposure**: single assessment at baseline (T0: 2002-2005; second assessment made at follow-up, but not

Study details	Sample	Alcohol exposure categories (patterns)	Details of the included article	Study dates
Europe) prospective cohort study		**Problem drinking: n**on-drinker (0 grams per day), CAGE score <2 (referent), score 2+<br>**Change in average consumption** (average of daily intake T0 and T1, recall 12 months prior): stable non-drinkers (consistently abstained), ex-drinkers (abstained T1 not T0), stable drinkers (stable consumption; referent), reduced drinking (higher consumption T0), increased drinking (higher consumption T1), and those who started drinking (abstained at T0 but not T1).	type; not specified whether asked in relation to a typical occasion/week/other). Recall: last 12 months. Lifetime: not measured.<br>Cognitive function: Specific cognitive domains (4 outcomes). **Learning and memory** (immediate recall of words in 3 x 1 minutes trials; delayed recall of words after other tests administered), **language** (verbal fluency, number animals named in 1 minute), **complex attention** (letter cancelled test for attention, mental speed, concentration). Test results were converted to Z-scores (mean =0; SD = 1) using whole sample means and SDs. Higher scores = better cognition.	used in prospective analysis)<br>**Outcome measures**: baseline and follow-up assessments at ~ 4 year intervals. (T0-T1: 2002-2008)<br>**Length of outcome follow-up**: 4 years from baseline (T0)
**Jacobus 2013**<br>United States<br>Cohort name: not reported	Based on 54[2] adolescents (40.7% female) aged 16-19 years at baseline and aged 19–22 years at final follow-up<br>2. 21 in alcohol + marijuana group	**Controls (CON)** consistent minimal alcohol (and marijuana) use (no binge episodes) since last follow-up<br>**Binge Drinking (BG)** engaging in heavy episodic alcohol use (≥4 drinks on one occasion for females and ≥5 drinks for males) and at least 3 binge episodes since last follow-up<br>**Binge Drinkers with Heavy Marijuana Use (BDHM)** (not relevant to this review)	Observational cohort reporting on prolonged patterns of alcohol (and marijuana use) on white matter integrity and neurocognitive functioning in late adolescents recruited from local high schools.<br>Inclusion criteria: consistent substance use (or non-use) over the 3 year follow-up period (i.e., reported the same pattern of use over 3 years). At project enrolment, binge drinkers were required to have < 10 lifetime marijuana use episodes. Controls were required to have < 20 lifetime alcohol use episodes and binge drinkers were required to have < 150 lifetime alcohol use episodes.<br>Exclusion criteria: history of: a lifetime DSM-IV Axis I disorder (other than cannabis or alcohol abuse or dependence), learning disability, neurological disorder or head trauma with loss of consciousness >2 minutes, serious physical health problem, complicated or premature birth including prenatal substance use; un-correctable sensory impairments; left handedness; MRI contraindications, and use of psychoactive medications (at T0).<br>Alcohol ascertainment: Lifetime and current (quantity/binge, frequency, change): the Customary Drinking and Drug Use Record, and the Timeline Follow-back asking about quantity and frequency. Recall: lifetime and last 28 days. Lifetime: measured. Problem drinking: binge drinking.<br>Cognitive function: **Global cognitive function** (calculated the average 5 domain scores at each time point). Specific cognitive domains: **complex attention, processing speed, verbal memory, visuospatial functioning**, and **executive functioning**. Multiple tests from which a composite score was calculated for each of the 5 domains Each measure was standardized for age and sex, and then converted to z-scores.	**Study period**: not reported<br>**Alcohol exposure**: multiple assessments - baseline and then 2 measures at 1.5 year intervals (T0-T2)<br>**Outcome measures**: multiple assessments - baseline and then 2 measures at 1.5 year intervals (T0-T2)<br>**Length of outcome follow-up**: 3 years
**Mota 2013**<br>Spain<br>Cohort name: not provided	Based on 89[3] university students (53.9% female) aged 18-19 years at baseline and 20-21 at follow-up	The classification criteria were based on responses to two questions: the third item of the AUDIT, and one question related to the rate of consumption (drinks per hour). Binge drinking (BD): ≥ 6 drinks per occasion (monthly or weekly) at a rate of ≥3 drinks per hour (heaviest consumption); Non binge drinking (non-BD): < 6	Observational cohort reporting on the relationship between binge drinking trajectory over university years and neuropsychological functioning in university students recruited from the University of Santiago de Compostela.<br>Inclusion criteria: healthy university students with no other relevant risk factors, such as psychiatric comorbidity or family history of alcoholism. Participants	**Study period**: not reported<br>**Alcohol exposure**: baseline and final follow-up (T0 &T1)

Study details	Sample	Alcohol exposure categories (patterns)	Details of the included article	Study dates
	3. 143 at baseline; 89 at follow-up and included in the analysis<br><br>Probably same sample involved in Carbia 2017	drinks per occasion (monthly or weekly) at a rate of ≤ 2 drinks per hour (heaviest consumption)<br>Patterns used in the analyses:<br>**Ex-binge drinkers** were classified as binge drinkers at baseline but at follow-up<br>**Non-binge drinkers** did not report a binge drinking pattern at baseline or follow-up<br>**Binge drinkers** reported a binge drinking pattern at baseline and follow-up	were required to not take alcohol or any other drug the day of the assessment, and to attend rested and on good health condition.<br>Exclusion criteria: history of neurological disorders (including loss of consciousness > 20 min); history of psychopathology (DSM-IV-TR Axis I and II); current psychopathological symptoms as assessed by the Symptom Checklist-90-R (SCL-90-R). Regular consumption of other drugs (e.g., opiates, hallucinogens, cocaine, amphetamines, or medically prescribed psychoactive substances), except nicotine and cannabis; alcohol-use disorders; severe non-corrected motor or sensory deficits; family history of major mental disorder; history of alcoholism in first-and second-degree relatives.<br>Alcohol ascertainment: The questionnaire included the Alcohol Use Disorders Identification Test (AUDIT) and questions related to alcohol use (rate of consumption, age of onset, etc.). Recall: no information. Lifetime: not measured. Problem drinking: not specifically assessed, but AUDIT (a test for alcohol disorders) used and 'alcohol-use disorder' was an exclusion criteria. .<br>Cognitive function: **specific cognitive domains** (2 outcomes; 18 measures). **Learning and memory** (episodic memory measured using: Rey-Auditory Verbal Learning Test; Logical Memory I and II; Family Pictures I and II). **Executive function** (Digits span backward subtest; the Spatial Location backward subtest of WAIS-III; the Self-Ordered and the Pointing Test (SOPT); Zoo Map and Key Search subtests of the Behavioural Assessment of Dysexecutive Syndrome (BADS)). Test results appear to be analysed as raw scores.	**Outcome measure**: baseline and final follow-up (T0 &T1)<br>**Length of outcome follow-up**: ~2 years
**Ngandu 2007**<br>Finland<br>Cohort name: the Cardiovascular Risk Factors Aging and Dementia (CAIDE) study	Based on 1341[4] (62.3% female) aged 50.2 years (mean) at baseline and aged 65-79 years at follow-up<br>4. 62 excluded from analysis (missing data)<br><br>2 cohorts (baseline dates):<br>1. 1972/1977 (n = 966)<br>2. 1982/1987 (n = 313)	1972/1977 cohort<br>**Non-drinker** never drank alcohol<br>**infrequent drinker** drank less frequently than once per month<br>**Frequent drinker** drank once per month or more often<br>1982/1987 cohort<br>For each alcohol type (beer, wine, spirit)<br>**Non-drinker** did not consume alcohol<br>**Low drinker** consumed amounts in lower half (at the median)<br>**high drinker** consumed amounts in upper half (at the median)	Observational cohort reporting associations between different patterns (frequency, quantity, type) of alcohol consumption in midlife and cognitive function in non-demented elderly persons in Eastern Finland derived from random population-based samples.<br>Inclusion criteria: aged from 65 to 79 years in 1997; non-demented.<br>Exclusion criteria: missing alcohol information.<br>Alcohol ascertainment: (different for each cohort) 1972/1977: current (frequency, change over time): self-report questionnaire, at baseline and follow-up, asking about frequency of consumption (never; < once a month; ≥once month). Change in drinking between midlife and late life was undertaken by creating 9 possible groups, i.e., never drinker in midlife and never drinker in late life etc. Recall: not reported. Lifetime: not measured. Problem drinking: not measured. 1982/1987: Current (quantity): self-report questionnaire, at baseline and follow-up, asking about quantity of beer, wine, and spirits; total weekly alcohol intake (g/ week) was determined, but not reported. 3 categories created (non-drinkers, drinkers divided into two groups at the median). At follow-up questions regarding quantity of cider, and if ceased drinking, were added. Recall: last week. Lifetime: not measured. Problem drinking: not measured.<br>Cognitive function: (for both cohorts) Global cognitive function (MMSE); specific cognitive domains (6 outcomes): episodic memory, semantic memory,	**Study period**: 1972-1998<br>**Alcohol exposure**: baseline and follow-up (T0 & T1: 1972/1977; 1982/1987 & 1998)<br>**Outcome measure**: single assessment at follow-up (T1: 1998)<br>**Length of outcome follow-up**: 21 years (mean)

Study details	Sample	Alcohol exposure categories (patterns)	Details of the included article	Study dates
			subjective memory, prospective memory, executive function, and psychomotor speed. Test results appear to be analysed as raw scores. Higher score = better cognition, except for subjective memory and executive function, where lower score = better cognition.	
**Nguyen-Louie 2017**<br>United States<br>Cohort name: not provided	Based on 215[5] adolescents (41% female) aged 12-15 years at baseline and aged ~20 years (mean) at final follow-up<br>5. 127 in AWDO group<br><br>Substudy of original large cohort (R01 AA13419)	**Age of first drinking onset (AFDO)** the age of first consuming at least 1 standard drink; analysed as a continuous variable<br>**Age of weekly drinking onset (AWDO)** the age when transitioned into weekly alcohol use; analysed as a continuous variable	Observational cohort examining the influence of age of first drinking onset and age of weekly alcohol use onset on neuropsychological performance in adolescents attending San Diego area public middle schools. Parents were also involved in the study and completed questionnaires.<br>Inclusion criteria: between ages 12 and 15 years at baseline; only participants who consumed at least 1 full drink during follow-up (determined retrospectively).<br>Exclusion criteria: prenatal alcohol (2 or more drinks a given week) or illicit drug exposure; birth prior to 35th gestational week; history of any neurological or DSM-IV Axis I disorder, head trauma or loss of consciousness (>2 minutes), chronic medical illness, learning or intellectual disability, psychoactive medication use; inadequate English comprehension; and non-correctable sensory problems. Potential participants were also excluded if they had ≥10 total lifetime drinking days, ≥3 lifetime experiences with marijuana, ≥5 lifetime cigarette uses, and history of other intoxicant use. All participants were asked not to use alcohol and other recreational drugs for at least 24 hours prior to the study, confirmed with breath alcohol concentration and urine drug screen in the laboratory.<br>Alcohol ascertainment: Age of onset, current (quantity, frequency) were assessed using the Customary Drinking and Drug Use Record and the Timeline Follow-back. Recall: lifetime and last 30 days. Lifetime: measured. Problem drinking: withdrawal symptoms and DSM-IV/DSM-5 SUD criteria were assessed after initiation of use.<br>Cognitive function: Specific cognitive domains (6 outcomes): verbal learning and memory, cognitive inhibition, psychomotor speed, working memory, visual attention, and visuospatial ability. Various neuropsychological assessments were undertaken which produced 26 variables. These variables were subjected to principal components analysis yielding 6 latent factors/outcomes – as above. Raw scores were transformed into z-scores. All outcomes were further transformed to ensure higher scores = better cognition.	**Study period**: 2003-2016<br>**Alcohol exposure**: multiple assessments - baseline and then annually<br>**Outcome measures**: multiple assessments - baseline and then annually<br>**Length of outcome follow-up**: 6.8 years (mean)
**Richard 2017**[†]<br>United States<br>Cohort name: The Rancho Bernardo Study	Based on 1,334 men and women (54% female) aged 55-84 years at baseline (point of first alcohol measure).	**Non-drinker (referent)**: 'no past alcohol use' or 'did not drink in last year'<br>**Infrequent drinking**: < 2 times per month<br>**Weekly drinking**: 1-4 times per week<br>**Near daily drinking**: 5-7 times per week	Observational cohort reporting association between different levels and patterns (by frequency) of alcohol consumption and cognitively healthy longevity (survival to age 85).<br>Inclusion criteria: Eligible participants were those with potential to reach 85 years during follow-up period (55-84 years at baseline), and assessed as having intact cognitive function prior to 85th birthday (or an assessment 2 years prior).<br>Exclusion criteria: Missing data on education status.	**Study period**: 1984-2009<br>**Alcohol exposure**: single assessment at baseline (T0: 1984-1987)

Study details	Sample	Alcohol exposure categories (patterns)	Details of the included article	Study dates
	Substudy of cohort examining heart disease risk factors.		Alcohol ascertainment: Current: self-report questionnaire asking about frequency of consumption and number of drinks (by alcohol type) in a typical week. Item and response options: "how often they consumed alcohol in an average week" (daily/almost daily; 3–4 times/week, 1–2 times/week, 1–2 times/month, or once/month). Lifetime: asked about any 'past alcohol use'.<br>Cognitive function: **Global cognitive function** (MMSE). Raw scores converted to Z-scores (adjusted for sex, age, education) using normative data. Cognitive impairment: Z-scores below −1.5. Outcomes reported: Cognitively Healthy Longevity (CHL: survival to age 85 without cognitive impairment), Cognitively Impaired Longevity (CIL: survival to age 85 with cognitive impairment).	**Outcome measures**: up to 6 assessments at ~ 4 year intervals. (T1-T6: 1988-2009)<br>**Length of outcome follow-up**: median of 13.9 years from baseline alcohol measurement
**Sabia 2011**† France<br>Cohort name: GAZEL cohort study	Based on 4,073 men aged ~45-55 years at point of first alcohol measure (T0) and 55-65 years at point of cognition measure (T10).<br><br>Substudy of GAZEL cohort study which was established to examine various diseases and health-related factors among workers in France's national electricity and gas company.	**Large decrease** (change in alcohol intake over 10 year period): ≥11 drinks fewer per week<br>**Small decrease** (change in alcohol intake over 10 year period): 4 to 10 fewer drinks per week<br>**Stable** (change in alcohol intake over 10 year period): 3 fewer to 4 more drinks per week<br>**Small increase** (change in alcohol intake over 10 year period): 5 to 11 more drinks per week<br>**Large increase** (change in alcohol intake over 10 year period): ≥12 more drinks per week<br><br>Grams per drink: reported as 10-12 grams	Observational cohort examining association between the trajectory of alcohol consumption (change in consumption over 10 years) and cognition at age ≥55 years. Also examines association between average level of alcohol consumption (based mean of annual measures over time) and cognitive function.<br>Inclusion criteria: Eligible participants were men aged ≥55 years at the time cognition was measured (T10) and working for the electricity and gas company in which the GAZEL cohort was based.<br>Exclusion criteria: Women (due to small number in the GAZEL cohort: ~10%); had no measure of alcohol consumption from T0-T4, T5-T9, or both; did not have full covariate data; did not participate in cognitive tests (n=4525, 48.2%).<br>Alcohol ascertainment: Current: self-report questionnaire asking about frequency of consumption and number of drinks per day (by alcohol type) in last 7 days. Calculated mean change in consumption over 10 year period (based on annual measures of consumption, T0-T9). Lifetime: no information.<br>Cognitive function: Specific cognition domain - **complex attention** measured by the Digit Symbol Substitution Test (DSST; subtest of the Weschler Adult Intelligence Scale). Mean scores reported for number of correct responses on 93 items (score range 0-93; higher score=better cognition).	**Study period**: 1992-2004<br>**Alcohol exposure**: 10 assessments at ~ 1 year intervals (T0-T9: 1992-2001, or 1993-2002, or 1994-2003; period determined by year of cognitive testing)<br>**Outcome measures**: single assessment (T10: 2002, or 2003, or 2004)<br>**Length of outcome follow-up**: 12 months from baseline (T9)

* For completeness, content is replicated for studies that examined both levels and patterns. Only information pertaining to alcohol categories and ascertainment differs, being limited to either patterns (as reported here) or levels (as reported in the corresponding table).
† Denotes a study that also contributed data on levels of alcohol consumption

Table 4.2. Funding sources, potential conflicts of interest, and ethics approval for studies that examined different levels of alcohol consumption

Study ID	Funding sources	Funders	Review authors' judgment of potential conflicts	Ethics approval
Arntzen 2010	Government<br>Not for profit organisation (including academic)	No information reported in this paper.<br>Various funders identified in Jacobsen 2012 (linked paper), all government or not-for-profit: for example, the National Screening Services, the Research Council of Norway, Northern Norway Regional Health Authority, Norwegian Council on Cardiovascular Diseases and Norwegian Foundation for Health and Rehabilitation	No conflicts identified	Yes
Downer 2015	No direct funding for study	None reported	No conflicts identified	Not reported
Hassing 2018	Government<br>Not for profit organisation (including academic)	The Bank of Sweden Tercentenary Foundation, the Alcohol Research Council of the Swedish Alcohol Retailing Monopoly, National Institute of Aging, National Institutes of Health, and The Swedish Research Council for Health, Working Life and Welfare—Forte	No conflicts identified	Yes
Heffernan 2016	Government	Australian National Health and Medical Research Council	No conflicts identified	Yes
Hogenkamp 2014	Government<br>Not for profit organisation (including academic)<br>Industry (not alcohol)	Swedish Research Council, Åhlens stiftelse, Swedish Brain Research Foundation, Tore Nilsons Foundation, Fredrik och Ingrid Thurings Foundation, Brain Foundation, Åke Wiberg Foundation, and Novo Nordisk Foundation.	No conflicts identified	Yes
Kesse-Guyot 2012	Government<br>Industry	French National Research Agency (nuANR-05-PNRA-010), the French Ministry of Health, Mederic (insurance agency), Sodexo (food catering company), Ipsen (pharmaceutical company), MGEN (insurance agency) and Pierre Fabre (pharmaceutical company).	Author has potentially conflicting interests (some industry funding from a food catering company; authors indicated this is not a conflict). No funder or sponsor involvement in study, but there is some industry funding (food catering company).	Yes
Kitamura 2017	Government<br>Not for profit organisation (including academic)	JSPS KAKENHI Grants, National Cancer Center Research and Development Fund	No conflicts identified	Yes
Lang 2007	Government	National Institutes of Health (NIH), Intramural Research Program, National Institute on Aging, NIH	Insufficient information reported to judge.	Not reported
McGuire 2007	Government	Funder not specified, but the study was conducted by the Centers for Disease Control and Prevention (CDCP), so it is likely CDCP funded the study.	Insufficient information reported to judge.	Not reported
Nooyens 2014	Government<br>Not for profit organisation (including academic)	This subtudy: Internationale Stichting Alzheimer Onderzoek.<br>Main cohort study: Ministry of Public Health, Welfare and Sport of The Netherlands, the National Institute for Public Health and the Environment, Europe Against Cancer programme of the European Commission (early phase).<br>Personal funding: European Commission: Public Health and Consumer Protection Directorate, Ministry of Public Health, Welfare and Sport of The Netherlands.	No conflicts identified	Yes

Study ID	Funding sources	Funders	Review authors' judgment of potential conflicts	Ethics approval
Piumatti 2018	Government<br>Not for profit organisation (including academic)	Economic and Social Research Council, Medical Research Council, Alcohol Research UK	No conflicts identified	Yes
Richard 2017	Government<br>Not for profit organisation (including academic)	National Institute on Alcohol Abuse and Alcoholism, National Institute of Aging, National Institute of Diabetes and Digestive and Kidney Diseases	No conflicts identified	Yes
Sabia 2011	Government<br>Can't tell if all the organisations listed are government.	EDF-GDF and INSERM, the 'Cohortes Santé TGIR Program', Agence nationale de la recherché (ANR) and Agence française de sécurité sanitaire de l'environnement et du travail (AFSSET), and the clinical examinations were funded by the Cnamts.	No conflicts identified	Yes
Sabia 2014	Government<br>Not for profit organisation (including academic)	British Medical Research Council, British Heart Foundation; National Heart, Lung, and Blood Institute, US NIH National Institute on Aging	No conflicts identified	Yes
Samieri 2013a	Government<br>Not for profit organisation (including academic)	National Institutes of Health (NIH), Pôle de Recherche et d'Enseignement Supérieur (PRES) Université de Bordeaux (France)	No conflicts identified	Yes
Solfrizzi 2007	Government<br>Not for profit organisation (including academic)	Italian National Research Council–CNR-Targeted Project on Aging, AFORIGE (Associazione per la Formazione e la Ricerca in Geriatria).	Insufficient information reported to judge.	Not reported
Stott 2008	Not reported	Not reported	No conflicts identified	Yes
Wardzala 2018	Government	VA Merit Review Award, United States Department of Veterans Affairs Biomedical Laboratory Research and Development, National Institutes of Health	No conflicts identified	Yes

Appendix 5. Characteristics of excluded studies – reasons for exclusion

195 studies were excluded from the review based on full-text screening against eligibility criteria.

Of these 195 studies, eight were coded as “near miss” because they met all eligibility criteria but measures of alcohol were collected concomitantly with measures of cognition and the authors modelled the association between alcohol consumption and cognition over time (Table 5.1). In many cases this was done to provide a more reliable measure of alcohol intake over time; however, the approach rendered the studies ineligible because the analysis were not limited to prospective measures of alcohol. For this dataset, it would have been possible to have examined the association between alcohol consumption at a fixed time and future cognition.

A further 19 studies were excluded to narrow the scope of the review to a priority question that could be addressed within the required timeframe and resources. Since a recent systematic (Xu 2017) examined the effects of different levels of alcohol on dementia, and presented a dose response analysis, we excluded 15 studies for which the only eligible outcome was dementia or major cognitive impairment (Table 5.2). In addition, we excluded studies that examined the effects of alcohol among specific subgroups (2 studies: alcohol use disorder or diabetes) or that only examined the effects of high levels of alcohol intake (Table 5.3).

The remaining 176 excluded studies were excluded based on one or more of the pre-specified eligibility criteria, as reported in Tables 5.4-5.12.

Table 5.1 Near miss studies: those that met all criteria, but analysed concomitant measures of alcohol intake and cognition at follow-up (8 studies)

	Reference	Reason for exclusion
1	Beydoun, M. A., A. A. Gamaldo, H. A. Beydoun, T. Tanaka, K. L. Tucker, S. A. Talegawkar, L. Ferrucci and A. B. Zonderman (2014). "Caffeine and ALCOHOL intakes and overall nutrient adequacy are associated with longitudinal cognitive performance among U.S. adults." Journal of Nutrition 144(6): 890-901.	Analysis based on concomitant measures of alcohol and cognition outcomes at follow-up
2	Hagger-Johnson, G., S. Sabia, E. J. Brunner, M. Shipley, M. Bobak, M. Marmot, M. Kivimaki and A. Singh-Manoux (2013). "Combined impact of smoking and heavy ALCOHOL use on cognitive decline in early old age: Whitehall II prospective cohort study." British Journal of Psychiatry 203(2): 120-125.	Analysis based on concomitant measures of alcohol and cognition outcomes at follow-up
3	Jurk, S., E. Mennigen, T. Goschke and M. N. Smolka (2016). "Low-level alcohol consumption during adolescence and its impact on cognitive control development." Addiction Biology: No-Specified.	Analysis based on concomitant measures of alcohol and cognition outcomes at follow-up
4	Klaming, R., J. Annese, D. J. Veltman and H. C. Comijs (2017). "Episodic memory function is affected by lifestyle factors: a 14-year follow-up study in an elderly population." Aging Neuropsychology & Cognition 24(5): 528-542.	Analysis based on concomitant measures of alcohol and cognition outcomes at follow-up
5	Lo, A. H. Y., R. J. Woodman, N. A. Pachana, G. J. Byrne and P. S. Sachdev (2014). "Associations between lifestyle and cognitive function over time in women aged 40-79 years." Journal of Alzheimer's Disease 39(2): 371-383.	Analysis based on concomitant measures of alcohol and cognition outcomes at follow-up
6	Nooyens, A. C. J., H. B. Bueno-de-Mesquita, B. M. van Gelder, M. P. J. van Boxtel and W. M. M. Verschuren (2014). "Consumption of alcoholic beverages and cognitive decline at middle age: the Doetinchem Cohort Study." British Journal of Nutrition 111(4): 715-723.	Analysis based on concomitant measures of alcohol and cognition outcomes at follow-up
7	Samieri, C., O. I. Okereke, E. E Devore and F. Grodstein (2013). "Long-term adherence to the Mediterranean diet is associated with overall cognitive status, but not cognitive decline, in women." Journal of Nutrition 143(4): 493-499.	Analysis based on concomitant measures of alcohol and cognition outcomes at follow-up
8	Zanjani, F., B. G. Downer, T. M. Kruger, S. L. Willis and K. W. Schaie (2013). "ALCOHOL effects on cognitive change in middle-aged and older adults." Aging & Mental Health 17(1): 12-23.	Analysis based on concomitant measures of alcohol and cognition outcomes at follow-up

Table 5.2 Dementia or major cognitive impairment is the only eligible outcome (15 studies; studies in Xu 2017 as indicated)

	Reference	Included in Xu 2017
1	Almeida, O. P., G. J. Hankey, B. B. Yeap, J. Golledge and L. Flicker (2014). "ALCOHOL consumption and cognitive impairment in older men: a mendelian randomization study." Neurology 82(12): 1038-1044.	No - not identified in list of excluded studies
2	Handing, E. P., R. Andel, P. Kadlecova, M. Gatz and N. L. Pedersen (2015). "Midlife Alcohol Consumption and Risk of Dementia Over 43 Years of Follow-Up: A Population-Based Study From the Swedish Twin Registry." Journals of Gerontology Series A-Biological Sciences & Medical Sciences 70(10): 1248-1254.	Yes
3	Heymann, D., Y. Stern, S. Cosentino, O. Tatarina-Nulman, J. N. Dorrejo and Y. Gu (2016). "The Association Between Alcohol Use and the Progression of Alzheimer's Disease." Current Alzheimer Research 13(12): 1356-1362.	No: post-dates search
4	Lobo, E., C. Dufouil, G. Marcos, B. Quetglas, P. Saz, E. Guallar, A. Lobo and Z. Workgroup (2010). "Is there an association between low-to-moderate alcohol consumption and risk of cognitive decline?" American Journal of Epidemiology 172(6): 708-716.	No - not identified in list of excluded studies
5	Mehlig, K., I. Skoog, X. Guo, M. Schutze, D. Gustafson, M. Waern, S. Ostling, C. Bjorkelund and L. Lissner (2008). "Alcoholic beverages and incidence of dementia: 34-year follow-up of the prospective population study of women in Goteborg." American Journal of Epidemiology 167(6): 684-691.	Yes
6	Nordstrom, P., A. Nordstrom, M. Eriksson, L. O. Wahlund and Y. Gustafson (2013). "Risk factors in late adolescence for young-onset dementia in men: A nationwide cohort study." JAMA Internal Medicine 173(17): 1612-1618.	No - not identified in list of excluded studies
7	Paganini-Hill, A., C. H. Kawas and M. M. Corrada (2016). "Lifestyle Factors and Dementia in the Oldest-old: The 90+ Study." Alzheimer Disease & Associated Disorders 30(1): 21-26.	Yes
8	Reijs, B. L. R., S. J. B. Vos, H. Soininen, J. Lotjonen, J. Koikkalainen, M. Pikkarainen, A. Hall, R. Vanninen, Y. Liu, S.-K. Herukka, Y. Freund-Levi, G. B. Frisoni, L. Frolich, F. Nobili, M. O. Rikkert, L. Spiru, M. Tsolaki, A. K. Wallin, P. Scheltens, F. Verhey and P. J. Visser (2017). "Association Between Later Life Lifestyle Factors and Alzheimer's Disease Biomarkers in Non-Demented Individuals: A Longitudinal Descriptive Cohort Study." Journal of Alzheimer's Disease 60(4): 1387-1395.	No: post-dates search
9	Stephan, B. C. M., C. Tzourio, S. Auriacombe, H. Amieva, C. Dufouil, A. Alperovitch and T. Kurth (2015). "Usefulness of data from magnetic resonance imaging to improve prediction of dementia: population based cohort study." BMJ 350: h2863.	No - not identified in list of excluded studies
10	Unverzagt, F. W., L. T. Guey, R. N. Jones, M. Marsiske, J. W. King, V. G. Wadley, M. Crowe, G. W. Rebok and S. L. Tennstedt (2012). "ACTIVE cognitive training and rates of incident dementia." Journal of the International Neuropsychological Society 18(4): 669-677.	No - not identified in list of excluded studies
11	Virta, J. J., T. Jarvenpaa, K. Heikkila, M. Perola, M. Koskenvuo, I. Raiha, J. O. Rinne and J. Kaprio (2010). "Midlife alcohol consumption and later risk of cognitive impairment: A twin followup study." Journal of Alzheimer's Disease 22(3): 939-948.	No - not identified in list of excluded studies
12	Weyerer, S., M. Schaufele, B. Wiese, W. Maier, F. Tebarth, H. van den Bussche, M. Pentzek, H. Bickel, M. Luppa, S. G. Riedel-Heller and g. German AgeCoDe Study (2011). "Current alcohol consumption and its relationship to incident dementia: results from a 3-year follow-up study among primary care attenders aged 75 years and older." Age & Ageing 40(4): 456-463.	Yes
13	Xu, G., X. Liu, Q. Yin, W. Zhu, R. Zhang and X. Fan (2009). "Alcohol consumption and transition of mild cognitive impairment to dementia." Psychiatry & Clinical Neurosciences 63(1): 43-49.	No - not identified in list of excluded studies
14	Xue, H., Q. Sun, L. Liu, L. Zhou, R. Liang, R. He and H. Yu (2017). "Risk factors of transition from mild cognitive impairment to Alzheimer's disease and death: A cohort study." Comprehensive Psychiatry 78: 91-97.	No: post-dates search
15	Zhou, S., R. Zhou, T. Zhong, R. Li, J. Tan and H. Zhou (2014). "Association of smoking and ALCOHOL drinking with dementia risk among elderly men in China." Current Alzheimer Research 11(9): 899-907.	Yes

Table 5.3 Study involves a specific subgroup (4 studies)

	Reference	Reason for exclusion
1	Durazzo, T. C., D. L. Pennington, T. P. Schmidt and D. J. Meyerhoff (2014). "Effects of cigarette smoking history on neurocognitive recovery over 8 months of abstinence in ALCOHOL-dependent individuals." Alcoholism: Clinical & Experimental Research 38(11): 2816-2825.	Subgroup: alcohol use disorder or high level consumption
2	Elwood, P., J. Galante, J. Pickering, S. Palmer, A. Bayer, Y. Ben-Shlomo, M. Longley and J. Gallacher (2013). "Healthy lifestyles reduce the incidence of chronic diseases and dementia: evidence from the Caerphilly cohort study." PLoS ONE [Electronic Resource] 8(12): e81877.	Subgroup: alcohol use disorder or high level consumption
3	Miguez-Burbano, M. J., M. Nair, J. E. Lewis and J. Fishman (2009). "The role of alcohol on platelets, thymus and cognitive performance among HIV-infected subjects: are they related?" Platelets 20(4): 260-267.	Subgroup: alcohol use disorder or high level consumption
4	Townsend, M. K., E. Devore, J. H. Kang and F. Grodstein (2009). "The relation between moderate alcohol consumption and cognitive function in older women with type 2 diabetes." Diabetes Research & Clinical Practice 85(3): 322-327.	Subgroup: women with diabetes

The excluded studies that follow (Table 5.4 to 5.12) are listed by the first criterion on which they were excluded. Other reasons for exclusion may apply. An alphabetical list of studies if provided in Appendix 9. Studies coded as clearly irrelevant are listed in the Appendix 9 only.

Table 5.4 Language other than English (2 studies)

	Reference	Reason for exclusion
1	Chanraud, S. and C. Bernard (2015). "Neuroimaging and alcoholism." Annales Medico-Psychologiques 173(3): 249-254.	Language other than English
2	Tang, H. D., Y. H. Yao, R. F. Xu, S. D. Chen and Q. Cheng (2008). "Analysis of cognitive impairment and associated factors of the elderly in Shanghai suburbs." Chinese Journal of Contemporary Neurology and Neurosurgery 8(4): 318-322.	Language other than English

Table 5.5 Does not examine effects of alcohol as an exposure (18 studies)

	Reference	Reason for exclusion
1	Brumback, T., D. Cao, P. McNamara and A. King (2017). "Alcohol-induced performance impairment: a 5-year re-examination study in heavy and light drinkers." Psychopharmacology 234(11): 1749-1759.	Does not examine effects of alcohol as an exposure.
2	Ceccanti, M., D. Hamilton, G. Coriale, V. Carito, L. Aloe, G. Chaldakov, M. Romeo, M. Ceccanti, A. Iannitelli and M. Fiore (2015). "Spatial learning in men undergoing alcohol detoxification." Physiology & Behavior 149: 324-330.	Does not examine effects of alcohol as an exposure.
3	Choi, I.-G., S.-I. Woo, H. J. Kim, D.-J. Kim, B. L. Park, H. S. Cheong, C. F. A. Pasaje, T. J. Park, J. S. Bae, Y. G. Chai and H. D. Shin (2010). "Lack of association between PRNP M129V polymorphism and multiple sclerosis, mild cognitive impairment, alcoholism and schizophrenia in a Korean population." Disease Markers 28(5): 315-321.	Does not examine effects of alcohol as an exposure.
4	Contador, I., F. Bermejo-Pareja, V. Puertas-Martin and J. Benito-Leon (2015). "Childhood and Adulthood Rural Residence Increases the Risk of Dementia: NEDICES Study." Current Alzheimer Research 12(4): 350-357.	Does not examine effects of alcohol as an exposure.
5	Czapla, M., J. J. Simon, B. Richter, M. Kluge, H. C. Friederich, S. Herpertz, K. Mann, S. C. Herpertz and S. Loeber (2015). "The impact of cognitive impairment and impulsivity on relapse of alcohol-dependent patients: Implications for psychotherapeutic treatment." Addiction Biology.	Does not examine effects of alcohol as an exposure.
6	Dingwall, K. M., P. Maruff and S. Cairney (2011). "Similar profile of cognitive impairment and recovery for Aboriginal Australians in treatment for episodic or chronic alcohol use." Addiction 106(8): 1419-1426.	Does not examine effects of alcohol as an exposure.
7	Marceau, E. M., J. Lunn, J. Berry, P. J. Kelly and N. Solowij (2016). "The Montreal Cognitive Assessment (MoCA) is Sensitive to Head Injury and Cognitive Impairment in a Residential Alcohol and Other Drug Therapeutic Community." Journal of Substance Abuse Treatment 66: 30-36.	Does not examine effects of alcohol as an exposure.

	Reference	Reason for exclusion
8	Maurage, F., P. de Timary, J. M. Tecco, S. Lechantre and D. Samson (2015). "Theory of mind difficulties in patients with alcohol dependence: beyond the prefrontal cortex dysfunction hypothesis." Alcoholism: Clinical & Experimental Research 39(6): 980-988.	Does not examine effects of alcohol as an exposure.
9	Nemoto, Y., T. Saito, S. Kanamori, T. Tsuji, K. Shirai, H. Kikuchi, K. Maruo, T. Arao and K. Kondo (2017). "An additive effect of leading role in the organization between social participation and dementia onset among Japanese older adults: the AGES cohort study." BMC Geriatrics 17(1): 297.	Does not examine effects of alcohol as an exposure.
10	Park, K.-Y., H.-S. Hwang, Y.-P. Kim and H.-K. Park (2017). "Risk factors for cognitive decline associated with gait speed in community-dwelling elderly Koreans with MMSE scores of 30." Aging-Clinical & Experimental Research 29(2): 183-189.	Does not examine effects of alcohol as an exposure.
11	Pelletier, S., B. Nalpas, R. Alarcon, H. Rigole and P. Perney (2016). "Investigation of Cognitive Improvement in Alcohol-Dependent Inpatients Using the Montreal Cognitive Assessment (MoCA) Score." Journal of Addiction Print 2016: 1539096.	Does not examine effects of alcohol as an exposure.
12	Pitel, A. L., J. Rivier, H. Beaunieux, F. Vabret, B. Desgranges and F. Eustache (2009). "Changes in the episodic memory and executive functions of abstinent and relapsed alcoholics over a 6-month period." Alcoholism: Clinical & Experimental Research 33(3): 490-498.	Does not examine effects of alcohol as an exposure.
13	Quaglino, V., E. De Wever and P. Maurage (2015). "Relations Between Cognitive Abilities, Drinking Characteristics, and Emotional Recognition in Alcohol Dependence: A Preliminary Exploration." Alcoholism: Clinical & Experimental Research 39(10): 2032-2038.	Does not examine effects of alcohol as an exposure.
14	Ritz, L., L. Coulbault, C. Lannuzel, C. Boudehent, S. Segobin, F. Eustache, F. Vabret, A. L. Pitel and H. Beaunieux (2016). "Clinical and Biological Risk Factors for Neuropsychological Impairment in Alcohol Use Disorder." PLoS ONE [Electronic Resource] 11(9): e0159616.	Does not examine effects of alcohol as an exposure.
15	Ros-Cucurull, E., R. F. Palma-Alvarez, C. Cardona-Rubira, E. Garcia-Raboso, C. Jacas, L. Grau-Lopez, A. C. Abad, L. Rodriguez-Cintas, S. Ros-Montalban, M. Casas, J. A. Ramos-Quiroga and C. Roncero (2018). "Alcohol use disorder and cognitive impairment in old age patients: A 6 months follow-up study in an outpatient unit in Barcelona." Psychiatry Research 261: 361-366.	Does not examine effects of alcohol as an exposure.
16	Vachon, D. D., R. F. Krueger, D. E. Irons, W. G. Iacono and M. McGue (2017). "Are Alcohol Trajectories a Useful Way of Identifying At-Risk Youth? A Multiwave Longitudinal-Epidemiologic Study." Journal of the American Academy of Child & Adolescent Psychiatry 56(6): 498-505.	Does not examine effects of alcohol as an exposure.
17	Yamamoto, N., G. Yamanaka, E. Takasugi, M. Ishikawa, T. Yamanaka, S. Murakami, T. Hanafusa, K. Matsubayashi and K. Otsuka (2009). "Lifestyle intervention reversed cognitive function in aged people with diabetes mellitus: two-year follow up." Diabetes Research & Clinical Practice 85(3): 343-346.	Does not examine effects of alcohol as an exposure.
18	Yen, C.-H., Y.-W. Yeh, C.-S. Liang, P.-S. Ho, S.-C. Kuo, C.-C. Huang, C.-Y. Chen, M.-C. Shih, K.-H. Ma, G.-S. Peng, R.-B. Lu and S.-Y. Huang (2015). "Reduced Dopamine Transporter Availability and Neurocognitive Deficits in Male Patients with Alcohol Dependence." PLoS ONE [Electronic Resource] 10(6): e0131017.	Does not examine effects of alcohol as an exposure.

Table 5.6 Examines multiple exposures; **no separate outcome data for alcohol** (N=13)

	Reference	Reason for exclusion
1	Assmann, K. E., C. Lassale, V. A. Andreeva, C. Jeandel, S. Hercberg, P. Galan and E. Kesse-Guyot (2015). "A healthy dietary pattern at midlife, combined with a regulated energy intake, is related to increased odds for healthy aging." Journal of Nutrition 145(9): 2139-2145.	Multiple exposures; does not report separate outcome data for alcohol
2	Bates, M. E., J. F. Buckman, G. T. Voelbel, D. Eddie and J. Freeman (2013). "The mean and the individual: Integrating variable-centered and person-centered analyses of cognitive recovery in patients with substance use disorders." Frontiers in Psychiatry 4.	Multiple exposures; does not report separate outcome data for alcohol
3	Gelber, R. P., H. Petrovitch, K. H. Masaki, R. D. Abbott, G. W. Ross, L. J. Launer and L. R. White (2012). "Lifestyle and the risk of dementia in Japanese-american men." Journal of the American Geriatrics Society 60(1): 118-123.	Multiple exposures; does not report separate outcome data for alcohol

	Reference	Reason for exclusion
4	Kimm, H., P. H. Lee, Y. J. Shin, K. S. Park, J. Jo, Y. Lee, H. C. Kang and S. H. Jee (2011). "Mid-life and late-life vascular risk factors and dementia in Korean men and women." Archives of Gerontology & Geriatrics 52(3): e117-122.	Multiple exposures; does not report separate outcome data for alcohol
5	Latvala, A., A. Tuulio-Henriksson, D. M. Dick, E. Vuoksimaa, J. Suvisaari, R. J. Viken, J. Kaprio and R. J. Rose (2009). "Cognitive functioning and alcohol dependence symptoms in young adulthood: Investigating the association in finnish twins." Behavior Genetics 39(6): 666.	Multiple exposures; does not report separate outcome data for alcohol
6	Lu, D., S. Ren, J. Zhang and D. Sun (2016). "Vascular risk factors aggravate cognitive impairment in first-ever young ischaemic stroke patients." European Journal of Neurology 23(5): 940-947.	Multiple exposures; does not report separate outcome data for alcohol
7	Lyu, J., S. H. Lee and H.-Y. Kim (2016). "Associations between healthy lifestyles and health outcomes among older Koreans." Geriatrics & gerontology international 16(6): 663-669.	Multiple exposures; does not report separate outcome data for alcohol
8	Norton, M. C., J. Dew, H. Smith, E. Fauth, K. W. Piercy, J. C. S. Breitner, J. Tschanz, H. Wengreen, K. Welsh-Bohmer and I. Cache County (2012). "Lifestyle behavior pattern is associated with different levels of risk for incident dementia and Alzheimer's disease: the Cache County study." Journal of the American Geriatrics Society 60(3): 405-412.	Multiple exposures; does not report separate outcome data for alcohol
9	Pearson, K. E., V. G. Wadley, L. A. McClure, J. M. Shikany, F. W. Unverzagt and S. E. Judd (2016). "Dietary patterns are associated with cognitive function in the REasons for Geographic And Racial Differences in Stroke (REGARDS) cohort." Journal of Nutritional Science 5: e38.	Multiple exposures; does not report separate outcome data for alcohol
10	Sabia, S., A. Singh-Manoux, G. Hagger-Johnson, E. Cambois, E. J. Brunner and M. Kivimaki (2012). "Influence of individual and combined healthy behaviours on successful aging." CMAJ Canadian Medical Association Journal 184(18): 1985-1992.	Multiple exposures; does not report separate outcome data for alcohol
11	Su, P., C.-C. Hsu, H.-C. Lin, W.-S. Huang, T.-L. Yang, W.-T. Hsu, C.-L. Lin, C.-Y. Hsu, K.-H. Chang and Y.-C. Hsu (2017). "Age-related hearing loss and dementia: a 10-year national population-based study." European Archives of Oto-Rhino-Laryngology 274(5): 2327-2334.	Multiple exposures; does not report separate outcome data for alcohol
12	Theadom, A., V. Parag, T. Dowell, K. McPherson, N. Starkey, S. Barker-Collo, K. Jones, S. Ameratunga, V. L. Feigin and B. R. Group (2016). "Persistent problems 1 year after mild traumatic brain injury: a longitudinal population study in New Zealand." British Journal of General Practice 66(642): e16-23.	Multiple exposures; does not report separate outcome data for alcohol
13	Voortman, T., J. C. Kiefte-de Jong, M. A. Ikram, B. H. Stricker, F. J. A. van Rooij, L. Lahousse, H. Tiemeier, G. G. Brusselle, O. H. Franco and J. D. Schoufour (2017). "Adherence to the 2015 Dutch dietary guidelines and risk of non-communicable diseases and mortality in the Rotterdam Study." European Journal of Epidemiology 32(11): 993-1005.	Multiple exposures; does not report separate outcome data for alcohol

Table 5.7 Alcohol not quantifiable (e.g. qualitative descriptors; alcohol/no alcohol; other) (67 studies)

Of the 67 studies excluded on this criterion, three examined patterns of drinking. These three studies were also ineligible based on other criteria

	Reference	Reason for exclusion
1	Aguirre-Acevedo, D. C., F. Lopera, E. Henao, V. Tirado, C. Munoz, M. Giraldo, S. I. Bangdiwala, E. M. Reiman, P. N. Tariot, J. B. Langbaum, Y. T. Quiroz and F. Jaimes (2016). "Cognitive Decline in a Colombian Kindred With Autosomal Dominant Alzheimer Disease: A Retrospective Cohort Study." JAMA Neurology 73(4): 431-438.	Alcohol not quantifiable
2	Barnes, D. E., J. A. Cauley, L.-Y. Lui, H. A. Fink, C. McCulloch, K. L. Stone and K. Yaffe (2007). "Women who maintain optimal cognitive function into old age." Journal of the American Geriatrics Society 55(2): 259-264.	Alcohol not quantifiable
3	Booker, A., L. E. Jacob, M. Rapp, J. Bohlken and K. Kostev (2016). "Risk factors for dementia diagnosis in German primary care practices." International Psychogeriatrics 28(7): 1059-1065.	Alcohol not quantifiable
4	Boot, B. P., C. F. Orr, J. E. Ahlskog, T. J. Ferman, R. Roberts, V. S. Pankratz, D. W. Dickson, J. Parisi, J. A. Aakre, Y. E. Geda, D. S. Knopman, R. C. Petersen	Alcohol not quantifiable

	Reference	Reason for exclusion
	and B. F. Boeve (2013). "Risk factors for dementia with Lewy bodies: a case-control study." Neurology 81(9): 833-840.	
5	Bos, I., S. J. Vos, L. Frolich, J. Kornhuber, J. Wiltfang, W. Maier, O. Peters, E. Ruther, S. Engelborghs, E. Niemantsverdriet, E. E. De Roeck, M. Tsolaki, Y. Freund-Levi, P. Johannsen, R. Vandenberghe, A. Lleo, D. Alcolea, G. B. Frisoni, S. Galluzzi, F. Nobili, S. Morbelli, A. Drzezga, M. Didic, B. N. van Berckel, E. Salmon, C. Bastin, S. Dauby, I. Santana, I. Baldeiras, A. de Mendonca, D. Silva, A. Wallin, A. Nordlund, P. M. Coloma, A. Wientzek, M. Alexander, G. P. Novak, M. F. Gordon, I. Alzheimer's Disease Neuroimaging, A. K. Wallin, H. Hampel, H. Soininen, S.-K. Herukka, P. Scheltens, F. R. Verhey and P. J. Visser (2017). "The frequency and influence of dementia risk factors in prodromal Alzheimer's disease." Neurobiology of Aging 56: 33-40.	Alcohol not quantifiable
6	Brion, M., F. D'Hondt, A.-L. Pitel, B. Lecomte, M. Ferauge, P. de Timary and P. Maurage (2017). "Executive functions in alcohol-dependence: A theoretically grounded and integrative exploration." Drug & Alcohol Dependence 177: 39-47.	Alcohol not quantifiable
7	Ceccanti, M., D. Hamilton, G. Coriale, V. Carito, L. Aloe, G. Chaldakov, M. Romeo, M. Ceccanti, A. Iannitelli and M. Fiore (2015). "Spatial learning in men undergoing alcohol detoxification." Physiology & Behavior 149: 324-330.	Alcohol not quantifiable
8	Chen, L. Y., Y. H. Wu, C. Y. Huang, L. K. Liu, A. C. Hwang, L. N. Peng, M. H. Lin and L. K. Chen (2017). "Predictive factors for dementia and cognitive impairment among residents living in the veterans' retirement communities in Taiwan: Implications for cognitive health promotion activities." Geriatrics and Gerontology International 17(Supplement 1): 7-13.	Alcohol not quantifiable
9	Chen, X., Y. Huang and H. G. Cheng (2012). "Lower intake of vegetables and legumes associated with cognitive decline among illiterate elderly Chinese: a 3-year cohort study." Journal of Nutrition, Health & Aging 16(6): 549-552.	Alcohol not quantifiable
10	Chen, Y., A. R. Sillaire, J. Dallongeville, E. Skrobala, D. Wallon, B. Dubois, D. Hannequin, F. Pasquier and Y. O. D. s. g. Lille (2017). "Low Prevalence and Clinical Effect of Vascular Risk Factors in Early-Onset Alzheimer's Disease." Journal of Alzheimer's Disease 60(3): 1045-1054.	Alcohol not quantifiable
11	Cherbuin, N., C. Reglade-Meslin, R. Kumar, P. Jacomb, S. Easteal, H. Christensen, P. Sachdev and K. J. Anstey (2009). "Risk factors of transition from normal cognition to mild cognitive disorder: the PATH through Life Study." Dementia & Geriatric Cognitive Disorders 28(1): 47-55.	Alcohol not quantifiable
12	Chiang, C.-J., P.-K. Yip, S.-C. Wu, C.-S. Lu, C.-W. Liou, H.-C. Liu, C.-K. Liu, C.-H. Chu, C.-S. Hwang, S.-F. Sung, Y.-D. Hsu, C.-C. Chen, S.-I. Liu, S.-H. Yan, C.-S. Fong, S.-F. Chang, S.-L. You and C.-J. Chen (2007). "Midlife risk factors for subtypes of dementia: a nested case-control study in Taiwan." American Journal of Geriatric Psychiatry 15(9): 762-771.	Alcohol not quantifiable
13	Fluharty, M. E., J. Heron and M. R. Munafo (2017). "Longitudinal associations of social cognition and substance use in childhood and early adolescence: findings from the Avon Longitudinal Study of Parents and Children." European Child & Adolescent Psychiatry: 20.	Alcohol not quantifiable
14	Fung, A. W. T., G. T. Y. Leung and L. C. W. Lam (2011). "Modulating factors that preserve cognitive function in healthy ageing." East Asian Archives of Psychiatry 21(4): 152-156.	Alcohol not quantifiable
15	Ganguli, M., B. Fu, B. E. Snitz, F. W. Unverzagt, D. A. Loewenstein, T. F. Hughes and C.-C. H. Chang (2014). "Vascular risk factors and cognitive decline in a population sample." Alzheimer Disease & Associated Disorders 28(1): 9-15.	Alcohol not quantifiable
16	Ganguli, M., B. Fu, B. E. Snitz, T. F. Hughes and C.-C. H. Chang (2013). "Mild cognitive impairment: incidence and vascular risk factors in a population-based cohort." Neurology 80(23): 2112-2120.	Alcohol not quantifiable
17	Ganguli, M., C.-W. Lee, B. E. Snitz, T. F. Hughes, E. McDade and C.-C. H. Chang (2015). "Rates and risk factors for progression to incident dementia vary by age in a population cohort." Neurology 84(1): 72-80.	Alcohol not quantifiable

	Reference	Reason for exclusion
18	Gow, A. J., W. Johnson, A. Pattie, M. C. Whiteman, J. Starr and I. J. Deary (2008). "Mental ability in childhood and cognitive aging." Gerontology 54(3): 177-186.	Alcohol not quantifiable
19	Hai, S., B. Dong, Y. Liu and Y. Zou (2012). "Occurrence and risk factors of mild cognitive impairment in the older Chinese population: a 3-year follow-up study." International Journal of Geriatric Psychiatry 27(7): 703-708.	Alcohol not quantifiable
20	Hajek, A. and H.-H. Konig (2016). "Longitudinal Predictors of Functional Impairment in Older Adults in Europe--Evidence from the Survey of Health, Ageing and Retirement in Europe." PLoS ONE [Electronic Resource] 11(1): e0146967.	Alcohol not quantifiable
21	Hao, L., X. Wang, L. Zhang, Y. Xing, Q. Guo, X. Hu, B. Mu, Y. Chen, G. Chen, J. Cao, X. Zhi, J. Liu, X. Li, L. Yang, J. Li, W. Du, Y. Sun, T. Wang, Z. Liu, Z. Liu, X. Zhao, H. Li, Y. Yu, X. Wang, J. Jia and Y. Han (2017). "Prevalence, Risk Factors, and Complaints Screening Tool Exploration of Subjective Cognitive Decline in a Large Cohort of the Chinese Population." Journal of Alzheimer's Disease 60(2): 371-388.	Alcohol not quantifiable
22	Harvanko, A. M., B. L. Odlaug, L. R. N. Schreiber and J. E. Grant (2012). "Cognitive task performance and frequency of ALCOHOL usage in young adults." Journal of Addiction Medicine 6(2): 106-111.	Alcohol not quantifiable
23	Heward, J., L. Stone, S.-M. Paddick, S. Mkenda, W. K. Gray, C. L. Dotchin, J. Kissima, C. Collingwood, B. Swai and R. W. Walker (2018). "A longitudinal study of cognitive decline in rural Tanzania: rates and potentially modifiable risk factors." International Psychogeriatrics: 1-11.	Alcohol not quantifiable
24	Holst, C., J. S. Tolstrup, H. J. Sorensen and U. Becker (2017). "Alcohol dependence and risk of somatic diseases and mortality: a cohort study in 19002 men and women attending alcohol treatment." Addiction 112(8): 1358-1366.	Alcohol not quantifiable
25	Hsu, W.-C., A. C. Tsai, Y.-C. Chen and J.-Y. Wang (2017). "Predicted factors for older Taiwanese to be healthy octogenarians: Results of an 18-year national cohort study." Geriatrics & gerontology international 17(12): 2579-2585.	Alcohol not quantifiable
26	Huang, C.-C., J.-D. Lee, D.-C. Yang, H.-I. Shih, C.-Y. Sun and C.-M. Chang (2017). "Associations Between Geriatric Syndromes and Mortality in Community-Dwelling Elderly: Results of a National Longitudinal Study in Taiwan." Journal of the American Medical Directors Association 18(3): 246-251.	Alcohol not quantifiable
27	Huntley, J., A. Corbett, K. Wesnes, H. Brooker, R. Stenton, A. Hampshire and C. Ballard (2018). "Online assessment of risk factors for dementia and cognitive function in healthy adults." International Journal of Geriatric Psychiatry 33(2): e286-e293.	Alcohol not quantifiable
28	Jacob, L., J. Bohlken and K. Kostev (2017). "Risk Factors for Mild Cognitive Impairment in German Primary Care Practices." Journal of Alzheimer's Disease 56(1): 379-384.	Alcohol not quantifiable
29	Kalapatapu, R. K., K. L. Delucchi, S. Wang, J. D. Harbison, E. E. Nelson and J. H. Kramer (2016). "Substance use history in behavioral-variant frontotemporal dementia versus primary progressive aphasia." Journal of Addictive Diseases 35(1): 36-41.	Alcohol not quantifiable
30	Kim, M. and J.-M. Park (2017). "Factors affecting cognitive function according to gender in community-dwelling elderly individuals." Epidemiology and health 39: e2017054.	Alcohol not quantifiable
31	Kim, S., Y. Kim and S. M. Park (2016). "Association between alcohol drinking behaviour and cognitive function: results from a nationwide longitudinal study of South Korea." BMJ Open 6(4): e010494.	Alcohol not quantifiable
32	Kimura, S., T. Ogata, J. Watanabe, T. Inoue and Y. Tsuboi (2017). "Does cerebral large-artery disease contribute to cognitive impairment?" eNeurologicalSci 8: 5-8.	Alcohol not quantifiable
33	Kitamura, K., Y. Watanabe, K. Nakamura, K. Sanpei, M. Wakasugi, A. Yokoseki, O. Onodera, T. Ikeuchi, R. Kuwano, T. Momotsu, I. Narita and N. Endo (2016). "Modifiable Factors Associated with Cognitive Impairment in	Alcohol not quantifiable

	Reference	Reason for exclusion
	1,143 Japanese Outpatients: The Project in Sado for Total Health (PROST)." Dementia and Geriatric Cognitive Disorders Extra 6(2): 341-349.	
34	Kuzma, E., D. J. Llewellyn, K. M. Langa, R. B. Wallace and I. A. Lang (2014). "History of ALCOHOL use disorders and risk of severe cognitive impairment: a 19-year prospective cohort study." American Journal of Geriatric Psychiatry 22(10): 1047-1054.	Alcohol not quantifiable
35	Lambert, M. E. (2016). "Differences in neurocognitive functioning associated with alcohol consumption in a multiethnic rural cohort: A Project FRONTIER study." Applied Neuropsychology Adult 23(5): 372-378.	Alcohol not quantifiable
36	Langballe, E. M., H. Ask, J. Holmen, E. Stordal, I. Saltvedt, G. Selbaek, A. Fikseaunet, S. Bergh, P. Nafstad and K. Tambs (2015). "Alcohol consumption and risk of dementia up to 27 years later in a large, population-based sample: the HUNT study, Norway." European Journal of Epidemiology 30(9): 1049-1056.	Alcohol not quantifiable
37	Latvala, A., A. E. Castaneda, J. Perala, S. I. Saarni, T. Aalto-Setala, J. Lonnqvist, J. Kaprio, J. Suvisaari and A. Tuulio-Henriksson (2009). "Cognitive functioning in substance abuse and dependence: a population-based study of young adults." Addiction 104(9): 1558-1568.	Alcohol not quantifiable
38	Lee, H., S. Park, K. Lim, K. Lim, Y. Park and J. Jang (2016). "Association between lifestyle and cognitive impairment among women aged 65 years and over in the Republic of Korea." Educational Gerontology 42(3): 198-208.	Alcohol not quantifiable
39	Luck, T., M. Luppa, S. Briel, H. Matschinger, H.-H. Konig, S. Bleich, A. Villringer, M. C. Angermeyer and S. G. Riedel-Heller (2010). "Mild cognitive impairment: incidence and risk factors: results of the leipzig longitudinal study of the aged." Journal of the American Geriatrics Society 58(10): 1903-1910.	Alcohol not quantifiable
40	Lyu, J. and S. H. Lee (2014). "ALCOHOL consumption and cognitive impairment among Korean older adults: does gender matter?" International Psychogeriatrics 26(2): 335-340.	Alcohol not quantifiable
41	McCallum, J., L. A. Simons, J. Simons and Y. Friedlander (2007). "Delaying dementia and nursing home placement: the Dubbo study of elderly Australians over a 14-year follow-up." Annals of the New York Academy of Sciences 1114: 121-129.	Alcohol not quantifiable
42	Nguyen-Louie, T. T., A. N. Simmons, L. M. Squeglia, M. Alejandra Infante, J. P. Schacht and S. F. Tapert (2018). "Earlier alcohol use onset prospectively predicts changes in functional connectivity." Psychopharmacology 235(4): 1041-1054.	Alcohol not quantifiable
43	Nguyen-Louie, T. T., A. Tracas, L. M. Squeglia, G. E. Matt, S. Eberson-Shumate and S. F. Tapert (2016). "Learning and Memory in Adolescent Moderate, Binge, and Extreme-Binge Drinkers." Alcoholism: Clinical & Experimental Research 40(9): 1895-1904.	Alcohol not quantifiable
44	Nguyen-Louie, T. T., N. Castro, G. E. Matt, L. M. Squeglia, T. Brumback and S. F. Tapert (2015). "Effects of Emerging Alcohol and Marijuana Use Behaviors on Adolescents' Neuropsychological Functioning Over Four Years." Journal of Studies on Alcohol & Drugs 76(5): 738-748.	Alcohol not quantifiable
45	Niu, M.-J., F.-Z. Yin, L.-X. Liu, Y. Fang, X.-M. Xuan and G.-F. Wu (2013). "Non-high-density lipoprotein cholesterol and other risk factors of mild cognitive impairment among Chinese type 2 diabetic patients." Journal of Diabetes & its Complications 27(5): 443-446.	Alcohol not quantifiable
46	Nowakowska, K., K. Jablkowska and A. Borkowska (2007). "[Cognitive dysfunctions in patients with alcohol dependence]." Psychiatria Polska 41(5): 693-702.	Alcohol not quantifiable
47	Ormstad, H., T. A. Rosness, A. L. M. Bergem, E. Bjertness and B. H. Strand (2016). "Alcohol consumption in the elderly and risk of dementia related death - A Norwegian prospective study with a 17-year follow-up." International Journal of Neuroscience 126(2): 135-144.	Alcohol not quantifiable
48	Park, B., J. Park, J. K. Jun, K. S. Choi and M. Suh (2013). "Gender differences in the association of smoking and drinking with the development of cognitive impairment." PLoS ONE [Electronic Resource] 8(10): e75095.	Alcohol not quantifiable

	Reference	Reason for exclusion
49	Parrish, K. H., O. E. Atherton, A. Quintana, R. D. Conger and R. W. Robins (2016). "Reciprocal relations between internalizing symptoms and frequency of alcohol use: Findings from a longitudinal study of Mexican-origin youth." Psychology of Addictive Behaviors 30(2): 203-208.	Alcohol not quantifiable
50	Peters, R., N. Beckett, M. Geneva, M. Tzekova, F. H. Lu, R. Poulter, N. Gainsborough, B. Williams, M.-C. de Vernejoul, A. Fletcher and C. Bulpitt (2009). "Sociodemographic and lifestyle risk factors for incident dementia and cognitive decline in the HYVET." Age & Ageing 38(5): 521-527.	Alcohol not quantifiable
51	Schwarzinger, M., B. G. Pollock, O. S. M. Hasan, C. Dufouil, J. Rehm and G. QalyDays Study (2018). "Contribution of alcohol use disorders to the burden of dementia in France 2008-13: a nationwide retrospective cohort study." The lancet Public Health 3(3): e124-e132.	Alcohol not quantifiable
52	Schwarzinger, M., S. P. Thiebaut, S. Baillot, V. Mallet and J. Rehm (2017). "Alcohol use disorders and associated chronic disease - a national retrospective cohort study from France.[Erratum appears in BMC Public Health. 2017 Sep 22;17 (1):736; PMID: 28938882]." BMC Public Health 18(1): 43.	Alcohol not quantifiable
53	Squeglia, L. M., A. D. Spadoni, M. A. Infante, M. G. Myers and S. F. Tapert (2009). "Initiating moderate to heavy alcohol use predicts changes in neuropsychological functioning for adolescent girls and boys.[Erratum appears in Psychol Addict Behav. 2010 Mar;24(1):118]." Psychology of Addictive Behaviors 23(4): 715-722.	Alcohol not quantifiable
54	Stephens, C., J. Spicer, C. Budge, B. Stevenson and F. Alpass (2015). "Accounting for differences in cognitive health between older adults in New Zealand and the USA." International Psychogeriatrics 27(4): 591-600.	Alcohol not quantifiable
55	Subramaniam, M., E. Abdin, J. A. Vaingankar and S. A. Chong (2013). "Gender differences in disability in a multiethnic Asian population: the Singapore Mental Health Study." Comprehensive Psychiatry 54(4): 381-387.	Alcohol not quantifiable
56	Takahashi, P. Y., C. R. Caldwell and P. V. Targonski (2011). "Effect of alcohol and tobacco use on vascular dementia: a matched case control study." Vascular Health & Risk Management 7: 685-691.	Alcohol not quantifiable
57	Toda, A., Y. Tagata, T. Nakada, M. Komatsu, N. Shibata and H. Arai (2013). "Changes in Mini-Mental State Examination score in Alzheimer's disease patients after stopping habitual drinking." Psychogeriatrics:The Official Journal of the Japanese Psychogeriatric Society 13(2): 94-98.	Alcohol not quantifiable
58	Tremolizzo, L., E. Bianchi, E. Susani, E. Pupillo, P. Messina, A. Aliprandi, A. Salmaggi, M. Cosseddu, A. Pilotto, B. Borroni, A. Padovani, C. Bonomini, O. Zanetti, I. Appollonio, E. Beghi and C. Ferrarese (2017). "Voluptuary Habits and Risk of Frontotemporal Dementia: A Case Control Retrospective Study." Journal of Alzheimer's Disease 60(2): 335-340.	Alcohol not quantifiable
59	Vaillant, G. E., O. I. Okereke, K. Mukamal and R. J. Waldinger (2014). "Antecedents of intact cognition and dementia at age 90 years: a prospective study." International Journal of Geriatric Psychiatry 29(12): 1278-1285.	Alcohol not quantifiable
60	van der Heide, I., U. Gehring, G. H. Koppelman and A. H. Wijga (2016). "Health-Related Factors Associated with Discrepancies between Children's Potential and Attained Secondary School Level: A Longitudinal Study." PLoS ONE [Electronic Resource] 11(12): e0168110.	Alcohol not quantifiable
61	Vincze, G., P. Almos, K. Boda, P. Dome, N. Bodi, G. Szlavik, E. Magloczki, M. Pakaski, Z. Janka and J. Kalman (2007). "Risk factors of cognitive decline in residential care in Hungary." International Journal of Geriatric Psychiatry 22(12): 1208-1216.	Alcohol not quantifiable
62	Virag, M., K. Janacsek, A. Horvath, Z. Bujdoso, D. Fabo and D. Nemeth (2015). "Competition between frontal lobe functions and implicit sequence learning: evidence from the long-term effects of alcohol." Experimental Brain Research 233(7): 2081-2089.	Alcohol not quantifiable
63	Vos, S. J. B., M. P. J. van Boxtel, O. J. G. Schiepers, K. Deckers, M. de Vugt, I. Carriere, J.-F. Dartigues, K. Peres, S. Artero, K. Ritchie, L. Galluzzo, E. Scafato, G. B. Frisoni, M. Huisman, H. C. Comijs, S. F. Sacuiu, I. Skoog, K. Irving, C. A. O'Donnell, F. R. J. Verhey, P. J. Visser and S. Kohler (2017). "Modifiable Risk	Alcohol not quantifiable

	Reference	Reason for exclusion
	Factors for Prevention of Dementia in Midlife, Late Life and the Oldest-Old: Validation of the LIBRA Index." Journal of Alzheimer's Disease 58(2): 537-547.	
64	Wadley, V. G., L. A. McClure, V. J. Howard, F. W. Unverzagt, R. C. Go, C. S. Moy, M. R. Crowther, C. R. Gomez and G. Howard (2007). "Cognitive status, stroke symptom reports, and modifiable risk factors among individuals with no diagnosis of stroke or transient ischemic attack in the REasons for Geographic and Racial Differences in Stroke (REGARDS) Study." Stroke 38(4): 1143-1147.	Alcohol not quantifiable
65	Wang, T., S. Xiao, K. Chen, C. Yang, S. Dong, Y. Cheng, X. Li, J. Wang, M. Zhu, F. Yang, G. Li, N. Su, Y. Liu, J. Dai and M. Zhang (2017). "Prevalence, Incidence, Risk and Protective Factors of Amnestic Mild Cognitive Impairment in the Elderly in Shanghai." Current Alzheimer Research 14(4): 460-466.	Alcohol not quantifiable
66	Weber, E., E. E. Morgan, J. E. Iudicello, K. Blackstone, I. Grant, R. J. Ellis, S. L. Letendre, S. Little, S. Morris, D. M. Smith, D. J. Moore, S. P. Woods and T. Group (2013). "Substance use is a risk factor for neurocognitive deficits and neuropsychiatric distress in acute and early HIV infection." Journal of Neurovirology 19(1): 65-74.	Alcohol not quantifiable
67	Yen, C.-H., C.-J. Yeh, C.-C. Wang, W.-C. Liao, S.-C. Chen, C.-C. Chen, J. Liang, T.-J. Lai, H.-S. Lin, S.-H. Lee and M.-C. Lee (2010). "Determinants of cognitive impairment over time among the elderly in Taiwan: results of the national longitudinal study." Archives of Gerontology & Geriatrics 50 Suppl 1: S53-57.	Alcohol not quantifiable

Table 5.8 Does not report an eligible outcome. (18 studies: no measure of global cognitive function, domain-specific cognitive function, diagnosis of cognitive impairment)

	Reference	Reason for exclusion
1	Bell, C. L., R. Chen, K. Masaki, P. Yee, Q. He, J. Grove, T. Donlon, J. D. Curb, D. C. Willcox, L. W. Poon and B. J. Willcox (2014). "Late-life factors associated with healthy aging in older men." Journal of the American Geriatrics Society 62(5): 880-888.	Not cognitive function OR diagnosis of cognitive impairment
2	Berntsen, S., J. Kragstrup, V. Siersma, G. Waldemar and F. B. Waldorff (2015). "Alcohol consumption and mortality in patients with mild Alzheimer's disease: a prospective cohort study." BMJ Open 5(12): e007851.	Not cognitive function OR diagnosis of cognitive impairment
3	Britton, A., M. Shipley, A. Singh-Manoux and M. G. Marmot (2008). "Successful aging: the contribution of early-life and midlife risk factors." Journal of the American Geriatrics Society 56(6): 1098-1105.	Cognition part of composite outcome only
4	Cartier, J. L., S. C. Kukreja and E. Barengolts (2017). "LOWER SERUM 25-HYDROXYVITAMIN D IS ASSOCIATED WITH OBESITY BUT NOT COMMON CHRONIC CONDITIONS: AN OBSERVATIONAL STUDY OF AFRICAN AMERICAN AND CAUCASIAN MALE VETERANS." Endocrine Practice 23(3): 271-278.	Cognition part of composite outcome only
5	Ellingson, J. M., K. A. Fleming, A. Verges, B. D. Bartholow and K. J. Sher (2014). "Working memory as a moderator of impulsivity and ALCOHOL involvement: testing the cognitive-motivational theory of ALCOHOL use with prospective and working memory updating data." Addictive Behaviors 39(11): 1622-1631.	Not cognition as an outcome (predictor variable)
6	Feng, Q., J. Son and Y. Zeng (2015). "Prevalence and correlates of successful ageing: a comparative study between China and South Korea." European Journal of Ageing 12(2): 83-94.	Not cognitive function OR diagnosis of cognitive impairment
7	Harper, J., S. M. Malone and W. G. Iacono (2017). "Testing the effects of adolescent alcohol use on adult conflict-related theta dynamics." Clinical Neurophysiology 128(11): 2358-2368.	Not cognitive function OR diagnosis of cognitive impairment
8	Hatchard, T., A. M. Smith, R. E. Halchuk, C. A. Longo, P. A. Fried, M. J. Hogan and I. Cameron (2015). "Effects of low-level alcohol use on cognitive interference: an fMRI study in young adults." Alcohol 49(1): 7-13.	Not cognition as an outcome (predictor variable)
9	Heikkinen, N., E. Niskanen, M. Kononen, T. Tolmunen, V. Kekkonen, P. Kivimaki, H. Tanila, E. Laukkanen and R. Vanninen (2017). "Alcohol	Not cognitive function OR diagnosis of cognitive impairment

	Reference	Reason for exclusion
	consumption during adolescence is associated with reduced grey matter volumes." Addiction 112(4): 604-613.	
10	Langberg, J. M., M. R. Dvorsky, K. L. Kipperman, S. J. Molitor and L. D. Eddy (2015). "Alcohol Use Longitudinally Predicts Adjustment and Impairment in College Students with ADHD: The Role of Executive Functions." Psychology of Addictive Behaviors 29(2): 444-454.	Not cognition as an outcome (predictor variable)
11	Lopez-Caneda, E., F. Cadaveira, A. Crego, A. Gomez-Suarez, M. Corral, M. Parada, F. Caamano-Isorna and S. Rodriguez Holguin (2012). "Hyperactivation of right inferior frontal cortex in young binge drinkers during response inhibition: a follow-up study." Addiction 107(10): 1796-1808.	Not cognitive function OR diagnosis of cognitive impairment
12	Pfefferbaum, A., T. Rohlfing, K. M. Pohl, B. Lane, W. Chu, D. Kwon, B. Nolan Nichols, S. A. Brown, S. F. Tapert, K. Cummins, W. K. Thompson, T. Brumback, M. J. Meloy, T. L. Jernigan, A. Dale, I. M. Colrain, F. C. Baker, D. Prouty, M. D. De Bellis, J. T. Voyvodic, D. B. Clark, B. Luna, T. Chung, B. J. Nagel and E. V. Sullivan (2016). "Adolescent Development of Cortical and White Matter Structure in the NCANDA Sample: Role of Sex, Ethnicity, Puberty, and Alcohol Drinking." Cerebral Cortex 26(10): 4101-4121.	Not cognitive function OR diagnosis of cognitive impairment
13	Postuma, R. B., A. Iranzo, B. Hogl, I. Arnulf, L. Ferini-Strambi, R. Manni, T. Miyamoto, W. Oertel, Y. Dauvilliers, Y.-E. Ju, M. Puligheddu, K. Sonka, A. Pelletier, J. Santamaria, B. Frauscher, S. Leu-Semenescu, M. Zucconi, M. Terzaghi, M. Miyamoto, M. M. Unger, B. Carlander, M.-L. Fantini and J. Y. Montplaisir (2015). "Risk factors for neurodegeneration in idiopathic rapid eye movement sleep behavior disorder: a multicenter study." Annals of Neurology 77(5): 830-839.	Cognition part of composite outcome only
14	Rist, P. M., J. R. Marden, B. D. Capistrant, Q. Wu and M. M. Glymour (2015). "Do physical activity, smoking, drinking, or depression modify transitions from cognitive impairment to functional disability?" Journal of Alzheimer's Disease 44(4): 1171-1180.	Not cognitive function OR diagnosis of cognitive impairment
15	Sawyer, K. S., M. Oscar-Berman, S. Mosher Ruiz, D. A. Galvez, N. Makris, G. J. Harris and E. M. Valera (2016). "Associations Between Cerebellar Subregional Morphometry and Alcoholism History in Men and Women." Alcoholism: Clinical & Experimental Research 40(6): 1262-1272.	Not cognitive function OR diagnosis of cognitive impairment
16	Srinivasa, R. N., H. C. Rossetti, M. K. Gupta, R. N. Rosenberg, M. F. Weiner, R. M. Peshock, R. W. McColl, L. S. Hynan, R. T. Lucarelli and K. S. King (2016). "Cardiovascular Risk Factors Associated with Smaller Brain Volumes in Regions Identified as Early Predictors of Cognitive Decline." Radiology 278(1): 198-204.	Not cognition as an outcome (predictor variable)
17	Sun, Q., M. K. Townsend, O. I. Okereke, E. B. Rimm, F. B. Hu, M. J. Stampfer and F. Grodstein (2011). "Alcohol consumption at midlife and successful ageing in women: a prospective cohort analysis in the nurses' health study." PLoS Medicine / Public Library of Science 8(9): e1001090.	Cognition part of composite outcome only
18	Wang, R., L. Fratiglioni, E. J. Laukka, M. Lovden, G. Kalpouzos, L. Keller, C. Graff, A. Salami, L. Backman and C. Qiu (2015). "Effects of vascular risk factors and APOE epsilon4 on white matter integrity and cognitive decline." Neurology 84(11): 1128-1135.	Not cognition as an outcome (predictor variable)

Table 5.9 Ineligible population (1 study)

	Reference	Reason for exclusion
1	Silverberg, N. D., W. Panenka, G. L. Iverson, J. R. Brubacher, J. R. Shewchuk, M. K. S. Heran, G. C. S. Oh, W. G. Honer and R. T. Lange (2016). "Alcohol Consumption Does not Impede Recovery from Mild to Moderate Traumatic Brain Injury." Journal of the International Neuropsychological Society 22(8): 816-827.	People recovering from a traumatic brain injury

Table 5.10 Clearly not a cohort or nested case control study (e.g. cross sectional, case control, other) (22 studies)

	Reference	Reason for exclusion
1	Au Yeung, S. L., C. Jiang, W. Zhang, T. H. Lam, K. K. Cheng, G. M. Leung and C. M. Schooling (2010). "Moderate alcohol use and cognitive function in the Guangzhou Biobank Cohort study." Annals of Epidemiology 20(12): 873-882.	Not a cohort or nested case control study
2	Banz, B. C. (2015). "An evaluation of executive functions, cognitive control and a neurocognitive profile of college binge." Dissertation Abstracts International: Section B: The Sciences and Engineering 76(1-B(E)): No-Specified.	Not a cohort or nested case control study
3	Braun, A. (2015). "Binge drinking's cognitive and emotional correlates: A multi-definitional investigation." Dissertation Abstracts International: Section B: The Sciences and Engineering 76(1-B(E)): No-Specified.	Not a cohort or nested case control study
4	Buttaro, M. A. (2008). "Vascular risk factors and cognitive functioning in normal elderly." Dissertation Abstracts International: Section B: The Sciences and Engineering 68(8-B): 5600.	Not a cohort or nested case control study
5	Byeon, H., Y. Lee, S. Y. Lee, K. S. Lee, S. Y. Moon, H. Kim, C. H. Hong, S. J. Son and S. H. Choi (2015). "Association of alcohol drinking with verbal and visuospatial memory impairment in older adults: Clinical Research Center for Dementia of South Korea (CREDOS) study." International Psychogeriatrics 27(3): 455-461.	Not a cohort or nested case control study
6	Cairney, S., A. Clough, M. Jaragba and P. Maruff (2007). "Cognitive impairment in Aboriginal people with heavy episodic patterns of alcohol use." Addiction 102(6): 909-915.	Not a cohort or nested case control study
7	Cations, M., B. Draper, L.-F. Low, K. Radford, J. Trollor, H. Brodaty, P. Sachdev, P. Gonski, G. A. Broe and A. Withall (2018). "Non-Genetic Risk Factors for Degenerative and Vascular Young Onset Dementia: Results from the INSPIRED and KGOW Studies." Journal of Alzheimer's Disease 62(4): 1747-1758.	Not a cohort or nested case control study
8	Fama, R., E. V. Sullivan, S. A. Sassoon, A. Pfefferbaum and N. M. Zahr (2016). "Impairments in Component Processes of Executive Function and Episodic Memory in Alcoholism, HIV Infection, and HIV Infection with Alcoholism Comorbidity." Alcoholism: Clinical & Experimental Research 40(12): 2656-2666.	Not a cohort or nested case control study
9	Fan, X., A. O'Donnell, S. P. Singh, R. Pungan and L. C. Perlmuter (2008). "Light to moderate alcohol drinking is associated with higher cognitive function in males with type 2 diabetes." Experimental Aging Research 34(2): 126-137.	Not a cohort or nested case control study
10	Fein, G. and S. McGillivray (2007). "Cognitive performance in long-term abstinent elderly alcoholics." Alcoholism: Clinical & Experimental Research 31(11): 1788-1799.	Not a cohort or nested case control study
11	Franken, I. H. A., M. Luijten, F. M. van der Veen and J. W. van Strien (2017). "Cognitive control in young heavy drinkers: An ERP study." Drug & Alcohol Dependence 175: 77-83.	Not a cohort or nested case control study
12	Garcia, A. M., N. Ramon-Bou and M. Porta (2010). "Isolated and joint effects of tobacco and alcohol consumption on risk of Alzheimer's disease." Journal of Alzheimer's Disease 20(2): 577-586.	Not a cohort or nested case control study
13	Harwood, D. G., A. Kalechstein, W. W. Barker, S. Strauman, P. St George-Hyslop, C. Iglesias, D. Loewenstein and R. Duara (2010). "The effect of alcohol and tobacco consumption, and apolipoprotein E genotype, on the age of onset in Alzheimer's disease." International Journal of Geriatric Psychiatry 25(5): 511-518.	Not a cohort or nested case control study
14	Hawkins, L. A., S. Kilian, A. Firek, T. M. Kashner, C. J. Firek and H. Silvet (2012). "Cognitive impairment and medication adherence in outpatients with heart failure." Heart & Lung 41(6): 572-582.	Not a cohort or nested case control study
15	Houston, R. J., J. L. Derrick, K. E. Leonard, M. Testa, B. M. Quigley and A. Kubiak (2014). "Effects of heavy drinking on executive cognitive functioning in a community sample." Addictive Behaviors 39(1): 345-349.	Not a cohort or nested case control study

	Reference	Reason for exclusion
16	Hurstak, E., J. K. Johnson, L. Tieu, D. Guzman, C. Ponath, C. T. Lee, C. W. Jamora and M. Kushel (2017). "Factors associated with cognitive impairment in a cohort of older homeless adults: Results from the HOPE HOME study." Drug & Alcohol Dependence 178: 562-570.	Not a cohort or nested case control study
17	Muller-Oehring, E. M., Y.-C. Jung, A. Pfefferbaum, E. V. Sullivan and T. Schulte (2015). "The Resting Brain of Alcoholics." Cerebral Cortex 25(11): 4155-4168.	Not a cohort or nested case control study
18	Ritchie, S. J., T. C. Bates, J. Corley, G. McNeill, G. Davies, D. C. Liewald, J. M. Starr and I. J. Deary (2014). "ALCOHOL consumption and lifetime change in cognitive ability: a gene x environment interaction study." Age 36(3): 9638.	Not a cohort or nested case control study
19	Smith, K., L. Flicker, A. Dwyer, D. Atkinson, O. P. Almeida, N. T. Lautenschlager and D. LoGiudice (2010). "Factors associated with dementia in Aboriginal Australians." Australian & New Zealand Journal of Psychiatry 44(10): 888-893.	Not a cohort or nested case control study
20	Son, S. J., K. S. Lee, B. H. Oh and C. H. Hong (2012). "The effects of head circumference (HC) and lifetime ALCOHOL consumption (AC) on cognitive function in the elderly." Archives of Gerontology & Geriatrics 54(2): 343-347.	Not a cohort or nested case control study
21	Valls-Serrano, C., A. Verdejo-Garcia and A. Caracuel (2016). "Planning deficits in polysubstance dependent users: Differential associations with severity of drug use and intelligence." Drug & Alcohol Dependence 162: 72-78.	Not a cohort or nested case control study
22	Yamawaki, M., K. Wada-Isoe, M. Yamamoto, S. Nakashita, Y. Uemura, Y. Takahashi, T. Nakayama and K. Nakashima (2015). "Association of cerebral white matter lesions with cognitive function and mood in Japanese elderly people: a population-based study." Brain and Behavior 5(3): e00315.	Not a cohort or nested case control study

Table 5.11 Protocols, reports of baseline data, or studies with less than 6 months follow-up (13 studies)

	Reference	Reason for exclusion
1	(2016). ""Cognitive, emotion control, and motor performance of adolescents in the NCANDA study: Contributions from alcohol consumption, age, sex, ethnicity, and family history of addiction": Correction to Sullivan et al. (2016).[Erratum for Neuropsychology. 2016 May;30(4):449-73; PMID: 26752122]." Neuropsychology 30(7): 829.	Protocol or baseline data only; no eligible paper reporting follow-up data identified
2	Brown, S. A., T. Brumback, K. Tomlinson, K. Cummins, W. K. Thompson, B. J. Nagel, M. D. De Bellis, S. R. Hooper, D. B. Clark, T. Chung, B. P. Hasler, I. M. Colrain, F. C. Baker, D. Prouty, A. Pfefferbaum, E. V. Sullivan, K. M. Pohl, T. Rohlfing, B. N. Nichols, W. Chu and S. F. Tapert (2015). "The National Consortium on Alcohol and NeuroDevelopment in Adolescence (NCANDA): A Multisite Study of Adolescent Development and Substance Use." Journal of Studies on Alcohol & Drugs 76(6): 895-908.	Protocol or baseline data only; no eligible paper reporting follow-up data identified
3	Corley, J., X. Jia, C. E. Brett, A. J. Gow, J. M. Starr, J. A. M. Kyle, G. McNeill and I. J. Deary (2011). "Alcohol intake and cognitive abilities in old age: the Lothian Birth Cohort 1936 study." Neuropsychology 25(2): 166-175.	< 6 months f/up for all outcome measures
4	Davis, B. J. K., J.-S. Vidal, M. Garcia, T. Aspelund, M. A. van Buchem, M. K. Jonsdottir, S. Sigurdsson, T. B. Harris, V. Gudnason and L. J. Launer (2014). "The ALCOHOL paradox: light-to-moderate ALCOHOL consumption, cognitive function, and brain volume." Journals of Gerontology Series A-Biological Sciences & Medical Sciences 69(12): 1528-1535.	< 6 months f/up for all outcome measures
5	Deckers, K., S. Kohler, M. van Boxtel, F. Verhey, C. Brayne, J. Fleming and C. C. s. collaboration (2017). "Lack of associations between modifiable risk factors and dementia in the very old: findings from the Cambridge City over-75s cohort study." Aging & Mental Health: 1-7.	< 6 months f/up for all outcome measures
6	Dimitrov, I., I. Milanov, N. Deleva and B. Ivanov (2011). "Risk factors for dementia in a community-based sample of the Bulgarian urban population." Archives of the Balkan Medical Union 46(2): 147-149.	< 6 months f/up for all outcome measures

	Reference	Reason for exclusion
7	Groot, R. H. M., M. L. van Dijk and P. A. Kirschner (2015). "Cohort profile of the GOALS study: A large-scale research of physical activity in Dutch students." British Journal of Educational Technology 46(5): 947-952.	Protocol or baseline data only; no eligible paper reporting follow-up data identified
8	Levy, B., E. Manove and R. D. Weiss (2012). "Recovery of cognitive functioning in patients with co-occurring bipolar disorder and ALCOHOL dependence during early remission from an acute mood episode." Annals of Clinical Psychiatry 24(2): 143-154.	< 6 months f/up for all outcome measures
9	Peres, K., F. Matharan, M. Allard, H. Amieva, I. Baldi, P. Barberger-Gateau, V. Bergua, I. Bourdel-Marchasson, C. Delcourt, A. Foubert-Samier, A. Fourrier-Reglat, M. Gaimard, S. Laberon, C. Maubaret, V. Postal, C. Chantal, M. Rainfray, N. Rascle and J.-F. Dartigues (2012). "Health and aging in elderly farmers: the AMI cohort." BMC Public Health 12: 558.	Protocol or baseline data only; no eligible paper reporting follow-up data identified
10	Reas, E. T., G. A. Laughlin, D. Kritz-Silverstein, E. Barrett-Connor and L. K. McEvoy (2016). "Moderate, Regular Alcohol Consumption is Associated with Higher Cognitive Function in Older Community-Dwelling Adults." Jpad 3(2): 105-113.	< 6 months f/up for all outcome measures
11	Roberts, R. O., Y. E. Geda, J. R. Cerhan, D. S. Knopman, R. H. Cha, T. J. H. Christianson, V. S. Pankratz, R. J. Ivnik, B. F. Boeve, H. M. O'Connor and R. C. Petersen (2010). "Vegetables, unsaturated fats, moderate alcohol intake, and mild cognitive impairment." Dementia & Geriatric Cognitive Disorders 29(5): 413-423.	< 6 months f/up for all outcome measures
12	Steinberg, S. I., M. D. Sammel, B. T. Harel, A. Schembri, C. Policastro, H. R. Bogner, S. Negash and S. E. Arnold (2015). "Exercise, sedentary pastimes, and cognitive performance in healthy older adults." American Journal of Alzheimer's Disease and Other Dementias 30(3): 290-298.	Protocol or baseline data only; no eligible paper reporting follow-up data identified
13	Sullivan, E. V., T. Brumback, S. F. Tapert, R. Fama, D. Prouty, S. A. Brown, K. Cummins, W. K. Thompson, I. M. Colrain, F. C. Baker, M. D. De Bellis, S. R. Hooper, D. B. Clark, T. Chung, B. J. Nagel, B. N. Nichols, T. Rohlfing, W. Chu, K. M. Pohl and A. Pfefferbaum (2016). "Cognitive, emotion control, and motor performance of adolescents in the NCANDA study: Contributions from alcohol consumption, age, sex, ethnicity, and family history of addiction." Neuropsychology 30(4): 449-473.	Protocol or baseline data only; no eligible paper reporting follow-up data identified

Table 5.12 Other (4 studies)

	Reference	Reason for exclusion
1	Au Yeung, S. L., C. Q. Jiang, K. K. Cheng, B. Liu, W. S. Zhang, T. H. Lam, G. M. Leung and C. M. Schooling (2012). "Evaluation of moderate ALCOHOL use and cognitive function among men using a Mendelian randomization design in the Guangzhou biobank cohort study." American Journal of Epidemiology 175(10): 1021-1028.	Design - Mendelian randomisation
2	Eisenstein, A. R. (2012). "Individual and additive effects of lifestyle behaviors on cognition: A longitudinal study." Dissertation Abstracts International: Section B: The Sciences and Engineering 73(2-B): 914.	Thesis
3	Jones, S. B. (2016). "Association of mid-life alcohol consumption with stroke and cognitive decline in the Atherosclerosis Risk in Communities Study." Dissertation Abstracts International: Section B: The Sciences and Engineering 77(1-B(E)): No-Specified.	Thesis
4	Larsson, S. C., M. Traylor, R. Malik, M. Dichgans, S. Burgess, H. S. Markus and o. b. o. t. I. G. o. A. s. P. CoStream Consortium (2017). "Modifiable pathways in Alzheimer's disease: Mendelian randomisation analysis." BMJ 359: j5375.	Design - Mendelian randomisation

Appendix 6. Risk of bias assessment of included studies examining the effects of different levels of alcohol

Arntzen 2010

Study ID<br>Country	Sample	Confounders measured (*adjusted for)	Ascertainment of alcohol consumption	Ascertainment of cognitive function outcome	Data & analysis (noting analysis to account for missing data)
**Arntzen 2010**<br>Norway<br>Cohort name: the Tromsø Study	Participants were recruited at age 58 years (mean). Those with incomplete alcohol and covariate data were excluded, as were those who reported a stroke.	**Age:** yes*. **Sex**: yes. **SES**: education* (years grouped by primary/part secondary, secondary, high school/A-level, college/university). **Smoking:** yes* (current).<br>**Co-morbidities**: coronary heart disease*, self-report diabetes*, depression*, blood pressure*, BMI*. **Baseline cognition**: no. Other: physical activity*; HDL-cholesterol*	**Measurement**: Self-report data from questionnaire, single assessment at baseline (T0) used in current study. Current: asked "are you a teetotaller", and then "How many times a month do you normally drink alcohol", and "How many glasses of (beer/wine/spirits) do you normally drink in a fortnight" (3 items by type). Participants instructed not to "count low alcohol beer" and to "put 0 if less than once a month". Recall: not reported. Lifetime: no information.<br>**Categories**: No alcohol ("teetotaller"), 4 drinking categories defined (<1, 1-2, 3-4, >5 drinks per fortnight; mean/median intake not reported by group).	No information on who administered the neuropsychological tests. Comparable method of assessment for all participants. No information about whether those who administered the test were aware of alcohol consumption status (blinding), but unlikely that this was the case since alcohol was measured by self-report questionnaire at separate visits.	
**Overall risk of bias**<br>**Serious** due to selection of participants into the study and classification of alcohol consumption	RoB in selection of participants into the study: **Serious**<br>The lag time between initiating drinking and enrolment to the study means that those who previously experienced harmful outcomes associated with drinking may be excluded (because they died or were inaccessible, declined or were unable to participate, or did not meet health-related study eligibility criteria). Potential to bias through exclusion of drinkers with poorer health caused or exacerbated by alcohol consumption.	RoB due to confounding: **Moderate**.<br>(i) Confounding expected. All known important confounding domains appropriately controlled for.<br>(ii) No important concerns about the timing, or validity and reliability, of measurement of confounding domains, such that we do not expect serious residual confounding.	RoB in classification of alcohol consumption: **Serious.**<br>Use of a single assessment to categorise levels of drinking brings a risk of misclassifying consumption (i.e. variation in drinking patterns over time are missed). Contamination of the non-drinking group with occasional/former drinkers is likely (e.g. no lifetime measure; categorisation based on current drinking). Underestimation (through recall) or conscious under-reporting may amplify problems with misclassification.<br>RoB due to deviations from exposure as categorised through intervention: **Low**<br>Although plausible, intervention during alcohol measurement (e.g.an interviewer noting high alcohol intake), is unlikely to have an important effect on behaviour.	RoB in measurement of outcomes: **Low**<br>(i) The methods of outcome assessment were comparable across alcohol consumption groups.<br>(ii) The outcome assessors were likely to be unaware of alcohol consumption status of study participants.<br>(iii) Any error in measuring cognition is likely to be unrelated to alcohol consumption status.	RoB due to missing outcome data: **Moderate**<br>(i) Proportions of and reasons for missing participants differ slightly across groups. (ii) The analysis is unlikely to have addressed the risk of bias arising from the missing data.<br>RoB in selection of the reported result: **No Information**<br>No protocol (or statistical analysis plan) identified from which to determine if measures or analyses reported were selected on the basis of results. Hence there is too little information to make a judgement.

Downer 2015

Study ID<br>Country	Sample	Confounders measured (*adjusted for)	Ascertainment of alcohol consumption	Ascertainment of cognitive function outcome	Data & analysis(noting analysis to account for missing data)
**Downer 2015**<br>United States<br>Cohort name: Framingham Heart Study Offspring Cohort	Participants had alcohol measures at age 42 years (mean), and were then invited for baseline cognitive testing age ≥60 (T1). At T1, those with major cognitive impairment, stroke or who did not receive cognitive testing were excluded, as were heavy drinkers (≥ 5 drinks almost daily at T2).	**Age:** yes*. **Sex**: yes*. **SES**: education* (assessed at T1; < high school, high school degree, some college, college degree; WRAT-3* measure of reading/ education quality). **Smoking:** yes* (current, never, former). **Co-morbidities**: no (explicitly stated that did not control for diabetes, hypertension, heart disease, depression). **Baseline cognition**: no.<br>Other: APOE e4 allele status*	**Measurement**: Self-report data from questionnaire, single assessment at baseline (T0; only midlife assessment eligible for SR). Current: asked "how many bottles/glasses/drinks of beer/wine/cocktails" consumed per week (portion size defined for each type). Recall: 12 months. Lifetime: asked "have you ever drunk ≥ 5 drinks almost daily at any time of life" (this group excluded from analysis). **Categories**: Abstainers (0 per week, last 12 months), 3 drinking categories defined (1-6, 7-14, 15-34 drinks per week; mean intake reported by group).	No information on who administered the neurocognitive tests. Appears to be same method of assessment for all participants. No information about whether those who administered the test were aware of alcohol consumption status (blinding), but unlikely that this was the case since alcohol was measured by self-report questionnaire at separate visits.	
**Overall risk of bias**<br>**Serious** due to confounding, selection of participants into the study and classification of alcohol consumption	RoB in selection of participants into the study: **Serious**<br>The lag time between initiating drinking and enrolment to the study means that those who previously experienced harmful outcomes associated with drinking may be excluded (because they died or were inaccessible, declined or were unable to participate, or did not meet health-related study eligibility criteria). Potential to bias through exclusion of drinkers with poorer health caused or exacerbated by alcohol consumption.	RoB due to confounding: **Serious**<br>Co-morbidities were not controlled for.	RoB in classification of alcohol consumption: **Serious**<br>Use of a single assessment to categorise levels of drinking brings a risk of misclassifying consumption (i.e. variation in drinking patterns over time are missed). Inclusion of a lifetime measure of heavy drinking may lessen the risk of misclassification of heavy drinkers, but not other past drinkers. Underestimation (through recall) or conscious under-reporting may amplify problems with misclassification.<br>RoB due to deviations from exposure as categorised through intervention: **Low.** Although plausible, intervention during alcohol measurement (e.g.an interviewer noting high alcohol intake), is unlikely to have an important effect on behaviour.	RoB in measurement of outcomes: **Low**.<br>(i) The methods of outcome assessment were comparable across alcohol consumption groups.<br>(ii) The outcome assessors were likely to be unaware of alcohol consumption status of study participants.<br>(iii) Any error in measuring cognition is likely to be unrelated to alcohol consumption status.	RoB due to missing outcome data: **Moderate**.<br>(i) Proportions of and reasons for missing participants are likely to have differed across groups.<br>(ii) The analysis is unlikely to have addressed the risk of bias arising from the missing data.<br>RoB in selection of the reported result: **No Information**.<br>No protocol (or statistical analysis plan) identified from which to determine if measures or analyses reported were selected on the basis of results. Hence there is too little information to make a judgement.

Hassing 2018

Study ID<br>Country	Sample	Confounders measured (*adjusted for)	Ascertainment of alcohol consumption	Ascertainment of cognitive function outcome	Data & analysis (noting analysis to account for missing data)
**Hassing 2018**<br>Sweden<br>Cohort name: none – data from Swedish Twin Registry	Participants were ~ 56 to 66 years of age at first alcohol measurement (T0). Those with incomplete alcohol or covariate data, a dementia diagnosis at the baseline measure of cognition (T1), and non-drinkers were excluded.	**Age:** yes*. **Sex**: yes*. **SES**: education (years); socioeconomic position* (low, middle, high). **Smoking:** yes* (% ever smoked). **Co-morbidities**: clinical review of medical records: diabetes*, vascular disease* (hypertension, myocardial infarction, heart failure, stroke); BMI*. **Baseline cognition**: yes.<br>All covariates, except BMI, measured ≥20 years from alcohol measurement.	**Measurement**: Self-report data from questionnaire, single assessment at T0 (34 years prior to first cognition measure). Current: Asked whether they drank, how often, and how much on a typical occasion (by type). Items not reported, or whether response categories/portion sizes were defined. Recall: not reported. Lifetime: not measured.<br>**Categories**: Abstainers were excluded. Alcohol consumption analysed as a continuous variable (g/week). No categories, except for descriptive purposes and these were incompletely defined (no upper/lower bound).	No information about who administered cognitive tests (here or in earlier reported referenced for testing methods). Assume same method of assessment for all participants. No information about whether those who administered the test were aware of alcohol consumption status (blinding), but unlikely given the interval between alcohol measurement and cognitive testing.	
**Overall risk of bias**<br>**Critical** due to missing data and Serious risk of bias due to confounding, selection into the study, and classification of alcohol consumption	RoB in selection of participants into the study: **Serious**.<br>The lag time between initiating drinking and enrolment to the study means that those who previously experienced harmful outcomes associated with drinking may be excluded (because they died or were inaccessible, declined or were unable to participate, or did not meet health-related study eligibility criteria). Potential to bias through exclusion of drinkers with poorer health caused or exacerbated by alcohol consumption (including those excluded due to dementia).	RoB due to confounding: **Serious.**<br>Multiple important domains were measured post-baseline and then adjusted for in the analysis.<br>(ii) No important concerns about timing, reliability and validity, such that we do not expect serious residual confounding.	RoB in classification of alcohol consumption:<br>**Serious** Use of a single assessment to categorise levels of drinking brings a risk of misclassifying consumption (i.e. variation in drinking patterns over time are missed). Misclassification is highly likely (e.g. no measure prior to midlife; and categorisation based on drinking patterns >30 years prior to outcome measurement).<br>RoB due to deviations from exposure as categorised through intervention: **Low**<br>Although plausible, intervention during alcohol measurement (e.g.an interviewer noting high alcohol intake), is unlikely to have an important effect on behaviour.	RoB in measurement of outcomes: **Low**<br>i) The methods of outcome assessment were comparable across alcohol consumption groups.<br>(ii) The outcome assessors were likely to be unaware of alcohol consumption status of study participants.<br>(iii) Any error in measuring the cognition is likely to be unrelated to alcohol consumption status.	RoB due to missing outcome data: **Critical.**<br>Very high amount of missing data (61%), not reported if balanced across exposure groups (but unlikely to be balanced), and the analysis is unlikely to have addressed the risk of bias arising from the missing data.<br>RoB in selection of the reported result: **No Information.**<br>No protocol (or statistical analysis plan) identified from which to determine if measures or analyses reported were selected on the basis of results. Hence there is too little information to make a judgement.

Heffernan 2016

Study ID<br>Country	Sample	Confounders measured (*adjusted for)	Ascertainment of alcohol consumption	Ascertainment of cognitive function outcome	Data & analysis (noting analysis to account for missing data)
**Heffernan 2016**<br>Australia<br>Cohort name: Sydney Memory and Ageing Study	Participants were 70-90 years of age at first alcohol measurement (T0). Those with incomplete alcohol data, MMSE <24 at the baseline measure of cognition (T1), and two or fewer valid scores for cognition measures were excluded.	**Age:** yes*. **Sex**: yes*. **SES**: education* (years). **Smoking:** as part of CVD risk. **Co-morbidities**: CVD risk score* (smoking status, blood pressure, diabetic status, cholesterol/ lipoprotein (or BMI), hypertension medication); depression*. **Baseline cognition**: yes. Other: APO-E allele*.	**Measurement**: Self-report data from interview, single assessment at baseline (T0). Current: asked "how frequently they drank (monthly, weekly, daily)" and "the amount of drinks per drinking session" with pictures of standard drinks by type. Recall: last 12 months. Lifetime: ever "drank more heavily than in the last 12 months"; or if no alcohol in last 12 months "had they ever consumed".<br>**Categories**: No alcohol (last 12 months; referent), 2 drinking categories defined (0-2, >2 drinks per day for women; 0-4, >4 drinks per day for men; mean intake per category reported).	Neuropsychological tests administered by trained research psychologists. Same method of assessment for all participants. No information about whether those who administered the test were aware of alcohol consumption status (blinding), but unlikely that this was the case, especially at the two follow-up assessments where alcohol was not measured.	
**Overall risk of bias**<br>**Serious** due to selection of participants into the study and classification of alcohol consumption	RoB in selection of participants into the study: **Serious**.<br>The lag time between initiating drinking and enrolment to the study means that those who previously experienced harmful outcomes associated with drinking may be excluded (because they died or were inaccessible, declined or were unable to participate, or did not meet health-related study eligibility criteria). Potential to bias through exclusion of drinkers with poorer health caused or exacerbated by alcohol consumption (including those excluded due to cognitive impairment at baseline).	RoB due to confounding: **Moderate**.<br>(i) Confounding expected. All known important confounding domains appropriately controlled for.<br>(ii) No important concerns about the timing, or validity and reliability, of measurement of confounding domains, such that we do not expect serious residual confounding.	RoB in classification of alcohol consumption: **Serious.**<br>Use of a single assessment to categorise levels of drinking brings a risk of misclassifying consumption (i.e. variation in drinking patterns over time are missed). Contamination of the non-drinking group with occasional/former drinkers is likely (e.g. categorisation is based on current drinking). Authors reported that sensitivity analyses using NIAAA categories did not change the results (no sensitivity analysis using Australian standard categories).<br>RoB due to deviations from exposure as categorised through intervention: **Low.**<br>Although plausible, intervention during alcohol measurement (e.g.an interviewer noting high alcohol intake), is unlikely to have an important effect on behaviour.	RoB in measurement of outcomes: **Low**<br>i) The methods of outcome assessment were comparable across alcohol consumption groups.<br>(ii) The outcome assessors were likely to be unaware of alcohol consumption status of study participants.<br>(iii) Any error in measuring cognition is likely to be unrelated to alcohol consumption status.	RoB due to missing outcome data: **Moderate.**<br>(i) Proportions of and reasons for missing participants differ slightly across groups; (ii) The analysis is unlikely to have addressed the risk of bias arising from the missing data.<br>RoB in selection of the reported result: **No Information**.<br>No protocol (or statistical analysis plan) identified from which to determine if measures or analyses reported were selected on the basis of results. Hence there is too little information to make a judgement.

Hogenkamp 2014

Study ID<br>Country	Sample	Confounders measured (*adjusted for)	Ascertainment of alcohol consumption	Ascertainment of cognitive function outcome	Data & analysis (noting analysis to account for missing data)
**Hogenkamp 2014**<br>Sweden<br>Cohort name: Uppsala Longitudinal Study of Adult Men (ULSAM)	Participants were all men, 70 years of age at first alcohol measurement (T0). Those with incomplete alcohol data were excluded, as were those with an MMSE <25 at the baseline measure of cognition (T0).	**Age:** yes. **Sex**: no (all men). **SES**: education* (primary, secondary, university). **Smoking:** % smokers (yes/no). **Co-morbidities**: history of diabetes*; history of hypertension* (BP or medication); BMI*; CVD risk factors (cholesterol*). **Baseline cognition**: yes. Other: physical activity level*, dietary energy intake*, APO-E allele*.	**Measurement**: Self-report questionnaire, single assessment at baseline (T0). Current: self-report of usual intake of types of alcohol per week, e.g. "How much medium-alcohol beer (number of bottles) do you usually drink per week? Recall: not reported. Lifetime: not measured. Authors report that measure was validated by a 7-day pre-coded dietary record (results not reported). Recall: not reported. Lifetime: not measured.<br>**Categories**: Non-drinkers (0 drinks per week), 3 drinking categories defined (1, 2 and >2 drinks per day, 12 g/drink; mean intake per category reported) and also reported in quintiles. No referent since analysed as continuous variable. Those drinking 0-1.0 g/day were excluded from analysis (n=39).	Neuropsychological tests administered by one of the authors and two trained occupational therapists. Same method of assessment for all participants. No information about whether those administered the test were aware of alcohol consumption status (blinding), but unlikely that this was the case at the follow-up assessment.	Linear regression model of the change between follow-up and baseline TMT-B. Alcohol was modelled as a continuous variable (grams/day). The model adjusted for the covariates: highest educational degree, current smoking, physical activity, total energy intake, BMI, hypertension prevalence, diabetes prevalence, HDL and LDL cholesterol, and APO-E genotype. Results extracted from Table 2. The linear trend coefficient was reported with a p-value. No measure of precision was reported (i.e. confidence interval, standard error).
**Overall risk of bias**<br>**Serious** due to selection of participants into the study and classification of alcohol consumption	RoB in selection of participants into the study: **Serious**<br>The lag time between initiating drinking and enrolment to the study means that those who previously experienced harmful outcomes associated with drinking may be excluded (because they died or were inaccessible, declined or were unable to participate, or did not meet health-related study eligibility criteria). Potential to bias through exclusion of drinkers with poorer health caused or exacerbated by alcohol consumption (especially those excluded due to cognitive impairment at baseline).	RoB due to confounding: **Moderate**.<br>(i) Confounding expected. All known important confounding domains appropriately controlled for.<br>(ii) No important concerns about the timing, or validity and reliability, of measurement of confounding domains, such that we do not expect serious residual confounding.	RoB in classification of alcohol consumption: **Serious**.<br>Use of a single assessment to categorise levels of drinking brings a risk of misclassifying consumption (i.e. variation in drinking patterns over time are missed). Non/low level drinking group excluded from analysis, lessening issues with misclassification of occasional/former drinkers. However, categorisation based on current drinking.<br>RoB due to deviations from exposure as categorised through intervention: **Low.**<br>Although plausible, intervention during alcohol measurement (e.g.an interviewer noting high alcohol intake), is unlikely to have an important effect on behaviour.	RoB in measurement of outcomes: **Low**.<br>(i) The methods of outcome assessment were comparable across alcohol consumption groups.<br>(ii) The outcome assessors were likely to be unaware of alcohol consumption status of study participants.<br>(iii) Any error in measuring cognition is likely to be unrelated to alcohol consumption status.	RoB due to missing outcome data: **Moderate**.<br>(i) Proportions of and reasons for missing participants are likely to have differed across groups; (ii) The analysis is unlikely to have addressed the risk of bias arising from the missing data.<br>RoB in selection of the reported result: **No Information**<br>No protocol (or statistical analysis plan) identified from which to determine if measures or analyses reported were selected on the basis of results. Hence there is too little information to make a judgement.

Horvat 2015

**Study ID**<br>**Country**	**Sample**	**Confounders measured (*adjusted for)**	**Ascertainment of alcohol consumption**	**Ascertainment of cognitive function outcome**	**Data & analysis** (noting analysis to account for missing data)
**Horvat 2015**[†]<br>Eastern Europe<br>Cohort name: HAPIEE (Health, Alcohol, and Psychosocial Factors in Eastern Europe) prospective cohort study	Participants were 45-69 years of age at first alcohol measurement (T0). No exclusion criteria were reported.	**Age:** yes*. **Sex**: yes*. **SES**: education* (primary or less, vocational, secondary, university); household assets index*. **Smoking:** yes* (never, former, current). **Co-morbidities**: self-reported CVD, hypertension, diabetes; high depressive symptoms (measured by CESD-10 scale). **Baseline cognition**: yes (if ≥60 years; 20% sample of those 45-59 years) Other: leisure-time physical activity*.	**Measurement**: Self-report graduated frequency questionnaire, single assessment at baseline (T1 measure not used in prospective analysis). Lifetime: not measured. Current: asked about frequency (6 categories: "never" to "almost every day"), and amount by type of alcohol (beer, wine, spirits; 6 amounts: >10 drinks, 7-9 drinks, 5-6 drinks, 3-4 drinks, 1-2 drinks, 0 drinks). Recall: last 12 months.<br>**Categories**: Non-drinkers (0 drinks in last 12 months), light (referent; women/men: <5/10 grams/day), moderate, heavy (women/men: ≥20/40 g/d). Authors note "baseline information was not available on long-term abstention" so 'non-drinkers' includes lifetime abstainers and former drinkers.	Neuropsychological tests administered by a trained nurse. Same method of assessment for all participants. No information about whether interviewer was aware of alcohol consumption status (blinding), but unlikely that this was the case.	
**Overall risk of bias**<br>**Serious** due to selection of participants into the study and classification of alcohol consumption	RoB in selection of participants into the study: **Serious.**<br>The lag time between initiating drinking and enrolment to the study means that those who previously experienced harmful outcomes associated with drinking may be excluded (because they died or were inaccessible, declined or were unable to participate, or did not meet health-related study eligibility criteria). Potential to bias through exclusion of drinkers with poorer health caused or exacerbated by alcohol consumption.	RoB due to confounding: **Moderate.**<br>(i) Confounding expected. All known important confounding domains appropriately controlled for. (ii) No important concerns about the timing, or validity and reliability, of measurement of confounding domains, such that we do not expect serious residual confounding.	RoB in classification of alcohol consumption: **Serious**<br>Use of a single assessment to categorise levels of drinking brings a risk of misclassifying consumption (i.e. variation in drinking patterns over time are missed). Contamination of the non-drinking group with occasional/former drinkers is likely (e.g. no lifetime measure; categorisation based on current drinking), but low intake was used as referent.<br>RoB due to deviations from exposure as categorised through intervention: **Low.**<br>Although plausible, intervention during alcohol measurement (e.g.an interviewer noting high alcohol intake), is unlikely to have an important effect on behaviour.	RoB in measurement of outcomes: **Low**.<br>(i) The methods of outcome assessment were comparable across alcohol consumption groups.<br>(ii) The outcome assessors were likely to be unaware of alcohol consumption status of study participants.<br>(iii) Any error in measuring cognition is likely to be unrelated to alcohol consumption status.	RoB due to missing outcome data: **Low**.<br>Data were reasonably complete.<br>RoB in selection of the reported result: **No Information**.<br>No protocol (or statistical analysis plan) identified from which to determine if measures or analyses reported were selected on the basis of results. Hence there is too little information to make a judgement.

Kesse-Guyot 2012

**Study ID**<br>**Country**	**Sample**	**<u>Confounders measured (*adjusted for)</u>**	**Ascertainment of alcohol consumption**	**Ascertainment of cognitive function outcome**	**Data & analysis** (noting analysis to account for missing data)
**Kesse-Guyot 2012**<br>France<br>Cohort name: SU.VI.MAX 2 cohort	Participants were 45-60 years of age at first alcohol measurement (T0). Those with incomplete alcohol data (<3/12 dietary records), covariate data, or incomplete cognitive tests were excluded.<br>Participants were initial selected for a randomised trial of dietary supplements for prevention of cancer and heart disease. Trial eligibility criteria were not reported, but likely that those with pre-existing heart disease were excluded (potentially associated with both alcohol and cognition).	**<u>Age:</u>** yes*. **<u>Sex</u>**: yes. **<u>SES</u>**: education* (primary, secondary, university / equivalent); occupation* (unemployed, manual labour, professional, self-employed/ farmer, managerial). **<u>Smoking:</u>** yes* (never, former, current). **<u>Co-morbidities</u>**: CV events (validated), CVD, measured diabetes*, BMI*, hypertension*, depression* (only at T1, CES-D). **<u>Baseline cognition</u>**: no (asked about 'memory troubles'*) Other: physical activity*.	**Measurement**: <u>Current</u>: 24 hour dietary record (bimonthly over 2 years, randomly assigned across 2 weekend days and 4 week days) asking about the number alcoholic drinks (by type) and portion size (validated photographs of 7 portion sizes, including 2 extreme). <u>Recall:</u> 24 hours. <u>Lifetime</u>: not measured. **Categories**: Non-drinkers. Other categories defined in grams per day (15-29.9 g/day used as referent). Authors note that they may not be able to "distinguish between abstainers and former drinkers" so 'non-drinkers' includes lifetime abstainers and former drinkers.	Neuropsychological tests administered by a trained neuropsychologists. Same method of assessment for all participants. No information about whether interviewer was aware of alcohol consumption status (blinding), but unlikely that this was the case.	
**Overall risk of bias**<br>**<u>Serious</u>** due to selection bias and classification of alcohol consumption	<u>RoB in selection of participants into the study</u>: **Serious**.<br>The lag time between initiating drinking and enrolment to the study means that those who previously experienced harmful outcomes associated with drinking may be excluded (because they died or were inaccessible, declined or were unable to participate, or did not meet health-related study eligibility criteria). Potential to bias through exclusion of drinkers with poorer health caused or exacerbated by alcohol consumption (especially cardiovascular health).	<u>RoB due to confounding</u>: **Moderate**.<br>i) Confounding expected. All known important confounding domains appropriately controlled for.<br>(ii) No important concerns about the timing, or validity and reliability, of measurement of confounding domains, such that we do not expect serious residual confounding.	<u>RoB in classification of alcohol consumption</u>: **Serious**.<br>Multiple assessments to categorise levels of drinking, but short recall for each, which brings a risk of misclassifying consumption (i.e. variation in drinking patterns over time are missed). Underestimation of intake (by modifying behaviour during measurement period) may amplify problems with misclassification. Contamination of the non-drinking group with occasional/former drinkers is likely (e.g. no lifetime measure; categorisation based on current drinking).<br><u>RoB due to deviations from exposure as categorised through intervention</u>: **Low**.<br>Unlikely to be any intervention during alcohol measurement that would lead to an important effect on behaviour.	<u>RoB in measurement of outcomes</u>: **Low**.<br>(i) The methods of outcome assessment were comparable across alcohol consumption groups.<br>(ii) The outcome assessors were likely to be unaware of alcohol consumption status of study participants.<br>(iii) Any error in measuring cognition is likely to be unrelated to alcohol consumption status.	<u>RoB due to missing outcome data:</u> **Moderate**.<br>(i) Proportions of and reasons for missing participants are likely to have differed across groups; (ii) The analysis is unlikely to have addressed the risk of bias arising from the missing data.<br><u>RoB in selection of the reported result:</u> **No Information.**<br>No protocol (or statistical analysis plan) identified from which to determine if measures or analyses reported were selected on the basis of results. Hence there is too little information to make a judgement.

Kitamura 2017

Study ID<br>Country	Sample	<u>Confounders measured (*adjusted for)</u>	Ascertainment of alcohol consumption	Ascertainment of cognitive function outcome	**Data & analysis** (noting analysis to account for missing data)
**Kitamura 2017**<br>Japan<br>Cohort name: Murakami Cohort Study	Participants were 44-79 years of age at first alcohol measurement (T0). No exclusion criteria were reported in this paper for this substudy or the original cohort.	<u>Age:</u> yes*. <u>Sex</u>: yes*. <u>SES</u>: education* (junior high, high school, university or above). <u>Smoking:</u> yes (as predictor variable). <u>Co-morbidities</u>: history of stroke*, history of diabetes*, BMI (as predictor variable). <u>Baseline cognition</u>: no. Other: physical activity (as predictor variable).	**Measurement**: Single assessment at baseline (T0). <u>Current</u>: limited information on how alcohol was measured "average frequency, amount, and types of drinks". <u>Recall:</u> 24 hours. <u>Lifetime</u>: no information except that "past drinkers" were initially classified as non-drinkers, suggesting participants may have been asked about lifetime drinking. **Categories**: Non-drinker or rare drinker (<1 gram per week; referent). Other categories defined in grams per week (1-149, 150-299, 300-449, ≥450). "Past drinkers" were included in the same group as other non-drinkers.	No information on who administered the MMSE, or whether they were aware of alcohol consumption status (blinding). Likely to be the same method of assessment for all participants. Since alcohol status was measured at a separate time point, it is likely the assessor was unaware of alcohol status.	
**Overall risk of bias**<br>**<u>Serious</u>** due to confounding, selection of participants into the study and classification of alcohol consumption	<u>RoB in selection of participants into the study</u>: **Serious**.<br>The lag time between initiating drinking and enrolment to the study means that those who previously experienced harmful outcomes associated with drinking may be excluded (because they died or were inaccessible, declined or were unable to participate). Potential to bias through exclusion of drinkers with poorer health caused or exacerbated by alcohol consumption. Unclear if any health-related study eligibility criteria that may also have led to disproportionate exclusion of sicker drinkers.	<u>RoB due to confounding</u>: **Serious.**<br>At least one known important domain was not controlled for.	<u>RoB in classification of alcohol consumption</u>: **Serious**.<br>Use of a single assessment, and short time frame, to categorise levels of drinking brings a risk of misclassifying consumption (i.e. variation in drinking patterns over time are missed). Underestimation (through recall) or conscious under-reporting may amplify problems with misclassification. Contamination of the non-drinking group with occasional/former drinkers is likely (e.g. unclear if there is a lifetime measure; categorisation based on current drinking).<br><u>RoB due to deviations from exposure as categorised through intervention</u>: **Low**.<br>Although plausible, intervention during alcohol measurement (e.g.an interviewer noting high alcohol intake), is unlikely to have an important effect on behaviour.	<u>RoB in measurement of outcomes</u>: **Low**.<br>(i) The methods of outcome assessment were comparable across alcohol consumption groups.<br>(ii) The outcome assessors were likely to be unaware of alcohol consumption status of study participants.<br>(iii) Any error in measuring cognition is likely to be unrelated to alcohol consumption status.	<u>RoB due to missing outcome data:</u> **No Information**.<br>Unclear if there is any missing data<br><u>RoB in selection of the reported result:</u> **No Information**.<br>No protocol (or statistical analysis plan) identified from which to determine if measures or analyses reported were selected on the basis of results. Hence there is too little information to make a judgement.

Lang 2007

Study ID<br>Country	Sample	<u>Confounders measured (*adjusted for)</u>	Ascertainment of alcohol consumption	Ascertainment of cognitive function outcome	**Data & analysis** (noting analysis to account for missing data)
**Lang 2007**<br>United States, United Kingdom<br>Cohort name: English Longitudinal Study of Ageing (ELSA); U.S Health and Retirement Study (HRS)	Participants were ≥65 years of age at first alcohol measurement (T0). No exclusion criteria were reported.	**<u>Age:</u>** yes*. **<u>Sex</u>**: yes* **<u>SES</u>**: education* (years), income*, wealth*. **<u>Smoking:</u>** yes* (never, ex, current). **<u>Co-morbidities</u>**: number of co-morbidities* (0, 1, >2 of heart condition, stroke, hypertension, diabetes, arthritis, dementia), depression*, BMI*. **<u>Baseline cognition</u>**: no. Other: exercise.	**<u>Measurement</u>**: Single assessment at baseline (T0). <u>Lifetime</u>: no information for HRS. For ELSA, non-drinkers asked if "they had quit for health reasons" (reported % never drank, quit for health reasons, quit for other reasons; not separated for analysis and no sensitivity analyses for cognition outcome). <u>Current</u>: asked about frequency ("Do you ever drink alcohol …", if 'yes' "how many days per week") and amount ("on average how much consumed" on drinking days). No information about whether response options for frequency or amount were provided. <u>Recall</u>: last 12 months (ELSA), last 3 months (HRS).<br>**<u>Categories</u>**: 0, >0-1 (referent), >1-2, >2 drinks per day.	No information about how or who administered cognitive tests, although it is likely that the same method of assessment was used for all participants. No information about whether person administering the test was aware of alcohol consumption status (blinding). No information about whether the tests used were valid measures of cognitive function.	Logistic regression model. Alcohol was modelled as a categorical variable. The model was adjusted multi-stage survey sampling and the covariates: age at baseline, sex, BMI, cigarette smoking, comorbidity (heart condition, stroke, high blood pressure, diabetes mellitus, arthritis, or dementia), income, wealth, exercise, depression (HRS data only). Models also fitted separately by sex. Results extracted from Figure 2 and text (pg 4, col 2). OR and CI reported for only one comparison in the text.
**Overall risk of bias**<br>**<u>Serious</u>** due to selection of participants into the study and classification of alcohol consumption	<u>RoB in selection of participants into the study</u>: **Serious**.<br>The lag time between initiating drinking and enrolment to the study means that those who previously experienced harmful outcomes associated with drinking may be excluded (because they died or were inaccessible, declined or were unable to participate) Potential to bias through exclusion of drinkers with poorer health caused or exacerbated by alcohol consumption. Unclear if any health-related study eligibility criteria that may also have led to disproportionate exclusion of sicker drinkers.	<u>RoB due to confounding</u>: **Moderate.**<br>(i) Confounding expected, all known important confounding domains appropriately controlled for;<br>(ii) No important concerns about the timing, or validity and reliability, of measurement of confounding domains, such that we do not expect serious residual confounding.	<u>RoB in classification of alcohol consumption</u>: **Serious**.<br>Use of a single assessment to categorise levels of drinking brings a risk of misclassifying consumption (i.e. variation in drinking patterns over time are missed). Contamination of the non-drinking group with occasional/former drinkers is likely (e.g. no lifetime measure; categorisation based on current drinking), and not examined for cognition outcome. Underestimation (through recall) or conscious under-reporting may amplify problems with misclassification.<br><u>RoB due to deviations from exposure as categorised through intervention</u>:<br>**Low.** Although plausible, intervention during alcohol measurement (e.g.an interviewer noting high alcohol intake), is unlikely to have an important effect on behaviour.	<u>RoB in measurement of outcomes</u>: **Low**.<br>(i) The methods of outcome assessment were comparable across alcohol consumption groups.<br>(ii) The outcome assessors were likely to be unaware of alcohol consumption status of study participants.<br>(iii) Any error in measuring cognition is likely to be unrelated to alcohol consumption status.	<u>RoB due to missing outcome data:</u> **Moderate**.<br>(i) Proportions of and reasons for missing participants differ slightly across groups. (ii) The analysis is unlikely to have addressed the risk of bias arising from the missing data.<br><u>RoB in selection of the reported result:</u> **No Information.**<br>No protocol (or statistical analysis plan) identified from which to determine if measures or analyses reported were selected on the basis of results. Hence there is too little information to make a judgement.

McGuire 2007

**Study ID**<br>**Country**	**Sample**	**<u>Confounders measured (*adjusted for)</u>**	**Ascertainment of alcohol consumption**	**Ascertainment of cognitive function outcome**	**Data & analysis** (noting analysis to account for missing data)
**McGuire 2007**<br>United States<br>Cohort name: Second Longitudinal Study of Aging (LSOA II)	Participants were ≥70 years of age at first alcohol measurement (T0). Those with cognitive impairment at baseline (1.5 SD units below the cohort mean), and missing measures of cognitive function were excluded.	**<u>Age:</u>** yes*. **<u>Sex</u>**: yes*. **<u>SES</u>**: education* (years); income (>$20K). **<u>Smoking:</u>** no (but adjusted for covariates associated with smoking). **<u>Co-morbidities</u>**: self-reported number of chronic conditions* (from diabetes, arthritis, heart disease, stroke, cancer, hypertension, asthma) and self-rate health. **<u>Baseline cognition</u>**: yes. Other: marital status*, ethnicity*.	**Measurement**: Self-report, two assessments. <u>Lifetime</u>: no information. <u>Current</u>: asked about frequency ("on how many days in the past year, on average, they drank alcoholic beverages (beer, wine, or liquor)"), and amount ("number of drinks consumed on those days"). No information about whether response options for frequency or amount were provided. <u>Recall</u>: last 12 months.<br>**Categories**: Non-drinkers (referent: 0 drinks in last 12 months), ≤1 drink/day, >1 drink/day. Grams per drink not reported.	Cognitive tests administered in an "adapted" telephone interview; unclear if items are a valid measure. No information on who administered. Appears that the same method of assessment was used for all participants. No information about whether interviewer was aware of alcohol consumption status (blinding), but unlikely that this was the case.	
**Overall risk of bias**<br>**<u>Critical</u>** due to missing data, also serious risk of bias due to selection into the study and classification of alcohol consumption	<u>RoB in selection of participants into the study</u>: **Serious**.<br>The lag time between initiating drinking and enrolment to the study means that those who previously experienced harmful outcomes associated with drinking may be excluded (because they died or were inaccessible, declined or were unable to participate, or did not meet health-related study eligibility criteria). Potential to bias through exclusion of drinkers with poorer health caused or exacerbated by alcohol consumption (including those excluded due to cognitive impairment).	<u>RoB due to confounding</u>: **Moderate**.<br>i) Confounding expected. All known important confounding domains appropriately controlled for.<br>(ii) No important concerns about the timing, or validity and reliability, of measurement of confounding domains, such that we do not expect serious residual confounding.	<u>RoB in classification of alcohol consumption</u>: **Serious**.<br>Use of two assessment (but only 2 years apart) to categorise levels of drinking brings a risk of misclassifying consumption (i.e. variation in drinking patterns over time are missed). Contamination of the non-drinking group with occasional/former drinkers is likely (e.g. no lifetime measure; categorisation based on current drinking). Underestimation (through recall) or conscious under-reporting may amplify problems with misclassification.<br><u>RoB due to deviations from exposure as categorised through intervention</u>: **Low**.<br>Although plausible, intervention during alcohol measurement (e.g.an interviewer noting high alcohol intake), is unlikely to have an important effect on behaviour.	<u>RoB in measurement of outcomes</u>: **Low.**<br>(i) The methods of outcome assessment were comparable across alcohol consumption groups.<br>(ii) The outcome assessors were likely to be unaware of alcohol consumption status of study participants.<br>(iii) Any error in measuring cognition is likely to be unrelated to alcohol consumption status.	<u>RoB due to missing outcome data:</u> **Critical**.<br>Very high amount of missing data (~50%), not reported if balanced across exposure groups (but unlikely to be balanced), and the analysis is unlikely to have addressed bias arising from the missing data.<br><u>RoB in selection of the reported result:</u> **No Information**.<br>No protocol (or statistical analysis plan) identified from which to determine if measures or analyses reported were selected on the basis of results. Hence there is too little information to make a judgement.

Piumatti 2018

**Study ID**<br>**Country**	**Sample**	**Confounders measured (*adjusted for)**	**Ascertainment of alcohol consumption**	**Ascertainment of cognitive function outcome**	**Data & analysis** (noting analysis to account for missing data)
**Piumatti 2018**<br>United Kingdom<br>Cohort name: UK Biobank prospective cohort	Participants were 40-73 years of age at first alcohol measurement (T0). Those who consumed alcohol infrequently (< one per week), had a history of neurological disorder (e.g. stroke, head trauma), and less than one valid score (from 7) on cognitive tests at baseline were excluded.	**Age:** yes*. **Sex**: yes*. **SES**: education* (no degree, degree); deprivation* (Townsend score). **Smoking:** yes* (non-smoker, previous, current). **Co-morbidities**: BMI*. No other co-morbidities measured or adjusted for. **Baseline cognition**: yes*. Other: physical activity* (walking days/week).	**Measurement**: Self-report questionnaire. Current: asked about frequency ("how often do you drink alcohol?" ['daily or almost daily', '3-4 times a week', 'once or twice a week', '1-3 times a month', 'special occasions only', 'never' 'prefer not to answer']), and amount (those who drank at least once per week asked: "how many alcoholic drinks consumed on average" [by type, volumes provided for standard drink)). Recall: no information. Lifetime: no information.<br>**Categories**: Alcohol consumption treated as a continuous variable in analyses (g/day). Analyses limited to 'weekly drinkers'.	Cognitive tests administered using computer based testing, so objective assessment (no concerns about blinding) The same method of assessment was used for all participants.	Restricted cubic splines of log transformed reaction time (milliseconds). Alcohol was modelled as a continuous variable (log transformed grams/day). A restricted cubic spline places a constraint in the relationship of linearity (between the predictor and outcome) up to a specified amount of alcohol. The model adjusted for the covariates: age, education, sex, and smoking (Table 2, footnote). It is noted that the model also adjusted for baseline cognition, however, the paper provides conflicting information as to whether this occurred (i.e. data presented in Tables 2 and 3 for the same results indicate contrary information re baseline adjustment). Interaction terms included to investigate if the relationship is modified by age and sex. Results extracted from Table 2 (pg 4) and study authors' interpretation (pg 4).
**Overall risk of bias**<br>**Serious** due to potential for residual confounding, selection of participants into the study and classification alcohol consumption	RoB in selection of participants into the study: **Serious**.<br>The lag time between initiating drinking and enrolment to the study means that those who previously experienced harmful outcomes associated with drinking may be excluded (because they died or were inaccessible, declined or were unable to participate, or did not meet health-related study eligibility criteria). Potential to bias through exclusion of drinkers with poorer health caused or exacerbated by alcohol consumption.	RoB due to confounding: **Serious.**<br>(i) Confounding expected. Most important confounding domains appropriately controlled for, but not diabetes. Some residual confounding possible.<br>(ii) No important concerns about the timing, or validity and reliability, of measurement of confounding domains.	RoB in classification of alcohol consumption: **Serious.**<br>Use of a single assessment to measure levels of drinking brings a risk of error in the measurement of consumption (i.e. variation in drinking patterns over time are missed). Underestimation (through recall) or conscious under-reporting may amplify this problem, as may measuring current intake only (i.e. no measure of lifetime drinking).<br>RoB due to deviations from exposure as categorised through intervention: **Low**.<br>Although plausible, intervention during alcohol measurement (e.g.an interviewer noting high alcohol intake), is unlikely to have an important effect on behaviour.	RoB in measurement of outcomes: **Low**.<br>(i) The methods of outcome assessment were comparable across alcohol consumption groups.<br>(ii) The outcome assessors were likely to be unaware of alcohol consumption status of study participants.<br>(iii) Any error in measuring cognition is likely to be unrelated to alcohol consumption status.	RoB due to missing outcome data: **Low.**<br>Data were reasonably complete.<br>RoB in selection of the reported result: **No Information**.<br>No protocol (or statistical analysis plan) identified from which to determine if measures or analyses reported were selected on the basis of results. Hence there is too little information to make a judgement.

Richard 2017

Study ID<br>Country	Sample	Confounders measured (*adjusted for)	Ascertainment of alcohol consumption	Ascertainment of cognitive function outcome	Data & analysis (noting analysis to account for missing data)
**Richard 2017**[†]<br>United States<br>Cohort name: The Rancho Bernardo Study	Participants were 55-84 years of age at first alcohol measurement (T0). The study outcome was cognitive function (intact or impaired) on or close to 85th birthday. Those who had cognitive impairment at any point prior to their 85th birthday were excluded, as were those unlikely to reach age 85 (at T0).	**Age**: yes*. **Sex**: yes*. **SES**: education (% some college), marital status. **Smoking**: yes* (% never, past, current). **Co-morbidities**: number of co-morbidities* (CVD, diabetes, stroke, TIA, hypertension, liver disease, cancer, metabolic syndrome); depression*, BMI* **Baseline cognition**: no. **Other**: exercise*, waist-hip ratio; self-perceived health compared to peers (better, same, worse), marital status*.	**Measurement**: Self-report questionnaire, single assessment at baseline. Lifetime: asked if "had ever drunk an alcoholic beverage". Current: asked if "had drunk an alcoholic beverage ... within the past 12 months". If 'yes', asked "how often" they "consumed alcohol in an average week" (daily/almost daily; 3–4 times/week, 1–2 times/week, 1–2 times/month, or once/month) and (2) "how many bottles or cans of beer, glasses of wine, mixed drinks, and liqueurs or other drinks they consume during an average week". Recall (current consumption): not stated.<br>**Categories**: Non-drinkers (referent: lifetime abstainers and former drinkers [no drinking last year]), moderate, heavy, excessive. Sensitivity analyses excluding those with self-rated health 'worse than peers' "yielded similar findings".	MMSE administered by a trained interviewer. Same method of assessment for all participants. No information about whether interviewer was aware of alcohol consumption status (blinding), but unlikely that this was the case.	
**Overall risk of bias**<br>**Serious** due to selection of participants into the study and classification of alcohol consumption	RoB in selection of participants into the study: **Serious**.<br>The lag time between initiating drinking and enrolment to the study means that those who previously experienced harmful outcomes associated with drinking may be excluded (because they died or were inaccessible, declined or were unable to participate, or did not meet health-related study eligibility criteria). Potential to bias through exclusion of drinkers with poorer health (including cognitive impairment) caused or exacerbated by alcohol consumption.	RoB due to confounding: **Moderate.**<br>(i) Confounding expected. All known important confounding domains appropriately controlled for.<br>(ii) No important concerns about the timing, or validity and reliability, of measurement of confounding domains, such that we do not expect serious residual confounding.	RoB due to deviations from exposure as categorised through intervention: **Serious.**<br>Use of a single assessment to categorise levels of drinking brings a risk of misclassifying consumption (i.e. variation in drinking patterns over time are missed). Contamination of the non-drinking group with occasional/former drinkers is likely (e.g. current and lifetime abstainers included).<br>RoB due to deviations from exposure as categorised: **Low**.<br>Although plausible, intervention during alcohol measurement (e.g.an interviewer noting high alcohol intake), is unlikely to have an important effect on behaviour.	RoB in measurement of outcomes: **Low**.<br>i) The methods of outcome assessment were comparable across alcohol consumption groups.<br>(ii) The outcome assessors were likely to be unaware of alcohol consumption status of study participants.<br>(iii) Any error in measuring cognition is likely to be unrelated to alcohol consumption status.	RoB due to missing outcome data: **Moderate**.<br>(i) Proportions of and reasons for missing participants differ slightly across intervention groups. (ii) The analysis is unlikely to have addressed the risk of bias arising from the missing data.<br>RoB in selection of the reported result: **No Information**.<br>No protocol (or statistical analysis plan) identified from which to determine if measures or analyses reported were selected on the basis of results. Hence there is too little information to make a judgement.

Sabia 2011

Study ID<br>Country	Sample	Confounders measured (*adjusted for)	Ascertainment of alcohol consumption	Ascertainment of cognitive function outcome	**Data & analysis** (noting analysis to account for missing data)
**Sabia 2011**<br>France<br>Cohort name: GAZEL cohort study	Participants were men, ~45-55 years of age at first alcohol measurement (T0), employed by France's national electricity and gas company. Those with <2 alcohol measures (one in each of T0-T4 and T5-T9), missing covariate data, or no cognitive testing were excluded.<br>The authors noted that 'non-drinkers were more likely to participate in the clinical examination, due perhaps to poor health' (p7).	**Age:** yes*. **Sex**: n/a. **SES**: education* (adjusted or stratified by: primary school, professional qualification, secondary school and more), occupational position (adjusted or stratified by: low, intermediate, high). **Smoking:** yes* (current, stopped ≤10years or >10 years ago, never). **Co-morbidities**: blood pressure, cholesterol, BMI (no diabetes or CVD). **Baseline cognition**: no. Other: marital/ cohabitation status*	**Measurement**: 10 annual assessments over 10 years (T0-T9) using a "validated" self-report quantity/ frequency questionnaire, Current: asked "about the frequency and the daily consumption of different alcoholic beverages (wine, beer, aperitif or spirits) … using drawings of standard alcoholic units. Recall: last 7 days. Lifetime: not measured; mean consumption over 10 years calculated from To-T9 data.<br>**Categories**: no alcohol (10 year), four groups for average weekly consumption in units of alcohol (1-3, 4-14 [referent], 15-21, >21).	Same method of assessment for all participants. No information about who administered the neurocognitive tests or whether they were aware of alcohol consumption status (blinding). However, this is a large cohort study and neurocognitive testing was done independently of alcohol measurement, so it is likely the interviewer was unaware of alcohol status.	**Missing data**: Weighted regression was used to account for missing data. Analyses showed similar results.
**Overall risk of bias**<br>**Serious** due to confounding, selection of participants into the study, classification of alcohol consumption and missing data	RoB in selection of participants into the study: **Serious.**<br>The lag time between initiating drinking and enrolment to the study means that those who previously experienced harmful outcomes associated with drinking may be excluded (declined to participate in alcohol measurement or cognitive testing). Potential to bias through exclusion of drinkers with poorer health caused or exacerbated by alcohol consumption. Since the study took place in a workplace, this may influence participation in measurement of alcohol intake.	RoB due to confounding: **Serious**.<br>At least one known important domain was not appropriately not controlled for (diabetes; smoking, BMI and other cardiovascular risk factors measured concurrently with cognition).	RoB in classification of alcohol consumption: **Serious**.<br>The use of up to 10 assessments over 10 years to categorise levels of drinking lessens misclassification of consumption (i.e. capturing variation in drinking patterns over time). Contamination of the non-drinking group with occasional/former drinkers may be less likely (e.g. no lifetime measure; but categorisation based last 10 years). However, the workplace setting may result in conscious under-reporting, and participants only required 2 alcohol measures to be eligible, amplifying problems with misclassification.<br>RoB due to deviations from exposure as categorised through intervention: **Low.**<br>Although plausible, intervention during alcohol measurement (e.g.an interviewer noting high alcohol intake), is unlikely to have an important effect on behaviour.	RoB in measurement of outcomes: **Low**.<br>i) The methods of outcome assessment were comparable across alcohol consumption groups.<br>(ii) The outcome assessors were likely to be unaware of alcohol consumption status of study participants.<br>(iii) Any error in measuring cognition is likely to be unrelated to alcohol consumption status.	RoB due to missing outcome data: **Serious.**<br>Very high amount of missing data (57%), not balanced across exposure groups, but the analysis accounted for the risk of bias arising from the missing data.<br>RoB in selection of the reported result: **No Information**.<br>No protocol (or statistical analysis plan) identified from which to determine if measures or analyses reported were selected on the basis of results. Hence there is too little information to make a judgement.

Sabia 2014

Study ID<br>Country	Sample	<u>Confounders measured (*adjusted for)</u>	Ascertainment of alcohol consumption	Ascertainment of cognitive function outcome	Data & analysis (noting analysis to account for missing data)
**Sabia 2014**<br>England<br>Whitehall II cohort study	Participants were British civil servants, 35-55 years of age at first alcohol measurement (T0). Those with missing alcohol or covariate data, and those who did not participate in the baseline or two follow-up assessments of cognition (T2-T4) were excluded.	**<u>Age:</u>** yes*. **<u>Sex</u>**: yes (stratification variable). **<u>SES</u>**: education* (university degree or higher), occupational position* (high, intermediate, low). **<u>Smoking:</u>** yes* (current, recent or long-term ex, never). **<u>Co-morbidities</u>**: prevalence of diabetes*, CVD*, hypertension*, depression*. **<u>Baseline cognition</u>**: yes. Other: ethnicity*, marital/ cohabitation status* physical activity*, fruit and vegetable consumption*.	**Measurement**: 3 assessments over 10 years (baseline – T0, T1, T2), self-report quantity/ frequency questionnaire. <u>Current</u>: item wording not reported. <u>Recall:</u> last 12 months (any consumption), last 7 days (quantity in 'drinks' by type of alcohol e.g. number of pints). <u>Lifetime</u>: not measured; mean consumption over 10 years calculated from T0, T1, T2 data.<br>**Categories**: abstainers (10 year), cessation (in last 10 years), occasional, and 3 groups for average daily consumption in grams (percentiles: 0-70 [referent], 70-90, >90).	Same method of assessment for all participants. No information about who administered the neurocognitive tests or whether they were aware of alcohol consumption status (blinding). However, this is a large cohort study and it is likely neurocognitive tests were administered independently of other measures and the interviewer was unaware of alcohol status.	Missing data: Linear mixed models were used to estimate the association between alcohol consumption and 10-year cognitive decline, adjusting for covariates that may predict missing data.
**Overall risk of bias**<br>**<u>Serious</u>** due to selection of participants into the study and classification of alcohol consumption	<u>RoB in selection of participants into the study</u>: **Serious**.<br>The lag time between initiating drinking and enrolment to the study means that those who previously experienced harmful outcomes associated with drinking may be excluded (because they died or were inaccessible, declined or were unable to participate). Potential to bias through exclusion of drinkers with poorer health caused or exacerbated by alcohol consumption.	<u>RoB due to confounding</u>: **Moderate**.<br>(i) Confounding expected. All known important confounding domains appropriately controlled for.<br>(ii) No important concerns about the timing, or validity and reliability, of measurement of confounding domains, such that we do not expect serious residual confounding.<br>The authors cautioned about interpretation of results for abstainers (10 year), noting they were likely to have different characteristics (e.g. higher proportion of women who weren't white compared to other groups).	<u>RoB in classification of alcohol consumption</u>: **Serious.**<br>The use of up to 3 assessments over 10 years to categorise levels of drinking lessens misclassification of consumption (i.e. variation in drinking patterns over time are more likely to be captured). Contamination of the non-drinking group with occasional/former drinkers may be less likely (e.g. no lifetime measure; but categorisation based last 10 years). However, the workplace setting may result in conscious under-reporting, amplifying problems with misclassification.<br><u>RoB due to deviations from exposure as categorised through intervention</u>:<br>**Low.** Although plausible, intervention during alcohol measurement (e.g.an interviewer noting high alcohol intake), is unlikely to have an important effect on behaviour.	<u>RoB in measurement of outcomes</u>: **Low**.<br>(i) The methods of outcome assessment were comparable across alcohol consumption groups.<br>(ii) The outcome assessors were likely to be unaware of alcohol consumption status of study participants.<br>(iii) Any error in measuring cognition is likely to be unrelated to alcohol consumption status.	<u>RoB due to missing outcome data:</u> **Moderate.**<br>(i) Proportions of and reasons for missing participants differ slightly across intervention groups; (ii) The analysis provides unbiased estimates under the assumption that the data are missing at random.<br><u>RoB in selection of the reported result:</u> **No Information.**<br>No protocol (or statistical analysis plan) identified from which to determine if measures or analyses reported were selected on the basis of results. Hence there is too little information to make a judgement.

**Study ID**<br>**Country**	**Sample**	**Confounders measured (*adjusted for)**	**Ascertainment of alcohol consumption**	**Ascertainment of cognitive function outcome**	**Data & analysis** (noting analysis to account for missing data)
**Samieri 2013**<br>United States<br>Women's Health Study	Participants were women, ≥60 years of age at first alcohol measurement (T0). Those with missing dietary data were excluded ('dietary data' not defined, but assumed to include alcohol).<br>Participants were initially recruited for a trial of aspirin and vitamin E for prevention of CVD and cancer. Trial eligibility criteria were not reported, but likely that those with pre-existing heart disease were excluded (potentially associated with both alcohol and cognition).	**Age:** yes*. **Sex**: n/a. **SES**: education* (bachelor's degree or higher), household income* (≥$50K/year). **Smoking:** yes* (current). **Co-morbidities**: history of diabetes*, hypertension*, hypercholesterolemia*, depression* (at baseline cognitive test), BMI*. **Baseline cognition**: yes. Other: physical activity*, diet*, energy intake*, ethnicity*, hormone use*.	**Measurement**: Current: single assessment (T0) using self-report food frequency questionnaire asking about frequency of consumption of foods and beverages, including alcohol ("never or less than once a month" to "six times per day"), and portion size (standard portion sizes were specified). Recall period: last 12 months. Lifetime: no information.<br>**Categories**: non-drinkers (0 last 12 months, referent), 1-14.9 g/day, ≥15 g/day. Possible that non-drinker category includes former drinkers and abstainers.	Telephone interview administered by a trained nurse (tool validated for telephone administration). Same method of assessment for all participants, but no information about whether interviewer was aware of alcohol consumption status (blinding). However, alcohol measures collected >5 years prior so it is likely the interviewer was unaware of alcohol status.	Linear regression model of the average of three measures of global cognitive function. Alcohol was modelled as a categorical variable. The model adjusted for the covariates: Models were adjusted for age at the start of cognitive testing, race, higher education, annual household income, energy intake, Women's Health Study randomized treatment assignment, regular vigorous exercise, body mass index, current smoking, history of type 2 diabetes, history of hypertension, history of hypercholesterolemia, post-menopausal hormone use, and history of depression. Results extracted from Table 2. No information on the scale range or standard deviation of the global cognitive function outcome (which is a linear combination of z-scores) is provided, precluding clinical interpretation.
**Overall risk of bias**<br>**Serious** due to selection of participants into the study and classification of alcohol consumption	RoB in selection of participants into the study: **Serious**<br>The lag time between initiating drinking and enrolment to the study means that those who previously experienced harmful outcomes associated with drinking may be excluded (because they died or were inaccessible, declined or were unable to participate). Potential to bias through exclusion of drinkers with poorer health caused or exacerbated by alcohol consumption.	RoB due to confounding: **Moderate**.<br>(i) Confounding expected. All known important confounding domains appropriately controlled for.<br>(ii) No important concerns about the timing, or validity and reliability, of measurement of confounding domains, such that we do not expect serious residual confounding.	RoB in classification of alcohol consumption: **Serious**.<br>Use of a single assessment to categorise levels of drinking brings a risk of misclassifying consumption (i.e. variation in drinking patterns over time are missed). Contamination of the non-drinking group with occasional/former drinkers is likely (e.g. no lifetime measure; categorisation based on current drinking). Underestimation (through recall) or conscious under-reporting may amplify problems with misclassification.<br>RoB due to deviations from exposure as categorised through intervention: **Low.**<br>Although plausible, intervention during alcohol measurement (e.g.an interviewer noting high alcohol intake), is unlikely to have an important effect on behaviour.	RoB in measurement of outcomes: **Low**.<br>(i) The methods of outcome assessment were comparable across alcohol consumption groups.<br>(ii) The outcome assessors were likely to be unaware of alcohol consumption status of study participants.<br>(iii) Any error in measuring cognition is likely to be unrelated to alcohol consumption status.	RoB due to missing outcome data: **Moderate**.<br>(i) Proportions of and reasons for missing participants differ slightly across intervention groups. (ii) The analysis is unlikely to have accounted for the risk of bias arising from the missing data.<br>RoB in selection of the reported result: **No Information**<br>No protocol (or statistical analysis plan) identified from which to determine if measures or analyses reported were selected on the basis of results. Hence there is too little information to make a judgement.

Solfrizzi 2007

Study ID<br>Country	Sample	<u>Confounders measured (*adjusted for)</u>	Ascertainment of alcohol consumption	Ascertainment of cognitive function outcome	**Data & analysis** (noting analysis to account for missing data)
**Solfrizzi 2007**<br>Italy<br>Cohort name: Italian Longitudinal Study on Aging (ILSA)	Participants were 65-84 years of age at first alcohol measurement (T0). Those with a confirmed diagnosis of dementia at baseline assessment of cognition (T0), with unknown level of education, or who refused cognitive testing were excluded.	**<u>Age:</u>** yes*. **<u>Sex</u>**: yes*. **<u>SES</u>**: education* (years). **<u>Smoking:</u>** yes* (cigarette pack-years). **<u>Co-morbidities</u>**: coronary artery disease* (CAD), stroke*, diabetes, hypertension*, (all confirmed through clinical exam), total cholesterol*, (BMI/obesity not mentioned). **<u>Baseline cognition</u>**: yes. Other: medications* (anxiolytics).	**Measurement**: Current: single assessment (T0) using self-report food frequency questionnaire asking about frequency of consumption of foods and beverages, including alcohol (times per day/month/year), and portion size (number of drinks by alcohol type; 3 portions sizes). Recall: last 12 months. Lifetime: asked 'when they had begun to drink' and 'how much beer or wine per day ever since' (to identify former drinkers, and changed patterns).<br>**Categories**: non-drinkers (0 last 12 months, referent). Drinking groups: >1, 1-2, ≥2 drinks/day (15 grams alcohol per drink). Sensitivity analyses removing former drinkers from abstainer group did not alter results	Clinical evaluation by a trained neurologist. It is not reported whether the neurologist was aware of the participants alcohol status (blinding). However the clinical exam and collection of alcohol data occurred >3 years apart, so it is unlikely the neurologist was aware of alcohol consumption status.	
**Overall risk of bias**<br>**<u>Serious</u>** due to selection of participants into the study, classification of alcohol consumption and missing data	<u>RoB in selection of participants into the study</u>: **Serious**<br>The lag time between initiating drinking and enrolment to the study means that those who previously experienced harmful outcomes associated with drinking may be excluded (because they died or were inaccessible, declined or were unable to participate, or did not meet health-related study eligibility criteria). Potential to bias through exclusion of drinkers with poorer health (including cognitive impairment) caused or exacerbated by alcohol consumption.	<u>RoB due to confounding</u>: **Moderate**<br>(i) Confounding expected. All known important confounding domains appropriately controlled for.<br>(ii) No important concerns about the timing, or validity and reliability, of measurement of confounding domains, such that we do not expect serious residual confounding.	<u>RoB in classification of alcohol consumption</u>: **Serious**<br>Use of a single assessment to categorise levels of drinking brings a risk of misclassifying consumption (i.e. variation in drinking patterns over time are missed). Contamination of the non-drinking group with occasional/former drinkers is likely (e.g. no lifetime measure; categorisation based on current drinking), but the authors reported that sensitivity analysis removing former drinkers did not alter results.<br><u>RoB due to deviations from exposure as categorised through intervention</u>: **Low**<br>Although plausible, intervention during alcohol measurement (e.g.an interviewer noting high alcohol intake), is unlikely to have an important effect on behaviour.	<u>RoB in measurement of outcomes</u>: **Low**<br>(i) The methods of outcome assessment were comparable across alcohol consumption groups.<br>(ii) The outcome assessors were likely to be unaware of alcohol consumption status of study participants.<br>(iii) Any error in measuring cognition is likely to be unrelated to alcohol consumption status.	<u>RoB due to missing outcome data:</u> **Serious**<br>(i) Proportions of missing participants differ substantially across interventions; or reasons for missing data differ substantially across groups; and<br>(ii) The analysis is unlikely to have accounted for the risk of bias arising from the missing data.<br><u>RoB in selection of the reported result:</u> **No Information**<br>No protocol (or statistical analysis plan) identified from which to determine if measures or analyses reported were selected on the basis of results. Hence there is too little information to make a judgement.

Stott 2007

Study ID<br>Country	Sample	Confounders measured (*adjusted for)	Ascertainment of alcohol consumption	Ascertainment of cognitive function outcome	Data & analysis (noting analysis to account for missing data)
**Stott 2007**<br>United Kingdom, Netherlands<br>Prospective Study of Pravastatin in the Elderly at Risk (PROSPER)	Participants were 70-82 years of age at first alcohol measurement (T0). Those with an MMSE≤24 at baseline assessment of cognition (T0), or a history of drug or alcohol abuse were excluded.	**Age:** yes*. **Sex**: yes. **SES**: education* (years). **Smoking:** yes* (current). **Co-morbidities**: prevalence of diabetes, history of vascular disease*, incident stroke*, blood pressure, BMI*. **Baseline cognition**: yes* (MMSE). Other: country* (Scotland, Ireland, Netherlands), body weight*.	**Measurement**: Single assessment (baseline – T0). No information about tools/items used. Current: item wording not reported "units per week". Recall: last month. Lifetime: no information.<br>**Categories**: non-drinker (not defined; referent), low and moderate intake for average daily consumption.	Same method of assessment for all participants. Trained nurses administered the neurocognitive tests. No information about whether they were aware of alcohol consumption status (blinding). However, cognitive data were collected as trial outcomes, at 12, 24, 36 months after baseline, so it is likely the interviewer was unaware of alcohol status.	
**Overall risk of bias**<br>**Serious** due to confounding, selection of participants into the study and classification of alcohol consumption	RoB in selection of participants into the study: **Serious**.<br>The lag time between initiating drinking and enrolment to the study means that those who previously experienced harmful outcomes associated with drinking may be excluded (because they died or were inaccessible, declined or were unable to participate, or did not meet health-related study eligibility criteria). Potential to bias through exclusion of drinkers with poorer health (including cognitive impairment) caused or exacerbated by alcohol consumption.	RoB due to confounding: **Serious**.<br>At least one known important domain was not appropriately measured, or not controlled for (diabetes)	RoB in classification of alcohol consumption: **Serious**.<br>Use of a single assessment to categorise levels of drinking brings a risk of misclassifying consumption (i.e. variation in drinking patterns over time are missed). Contamination of the non-drinking group with occasional/former drinkers is likely (e.g. no lifetime measure; categorisation based on current drinking). Underestimation (through recall) or conscious under-reporting may amplify problems with misclassification.<br>RoB due to deviations from exposure as categorised through intervention: **Low.**<br>Although plausible, intervention during alcohol measurement (e.g.an interviewer noting high alcohol intake), is unlikely to have an important effect on behaviour.	RoB in measurement of outcomes: **Low**<br>(i) The methods of outcome assessment were comparable across alcohol consumption groups.<br>(ii) The outcome assessors were likely to be unaware of alcohol consumption status of study participants.<br>(iii) Any error in measuring cognition is likely to be unrelated to alcohol consumption status.	RoB due to missing outcome data: **Low.**<br>No missing data, all participants included in the analysis<br>RoB in selection of the reported result: **No Information.**<br>No protocol (or statistical analysis plan) identified from which to determine if measures or analyses reported were selected on the basis of results. Hence there is too little information to make a judgement.

Wardzala 2018

**Study ID**<br>**Country**	**Sample**	**Confounders measured (*adjusted for)**	**Ascertainment of alcohol consumption**	**Ascertainment of cognitive function outcome**	**Data & analysis** (noting analysis to account for missing data)
**Wardzala 2018**<br>United States<br>Cohort name: Oregon Brain Aging Study (OBAS); Intelligent Systems for Assessing Aging Changes (ISAAC) study	Participants were ~80 years of age or older at first alcohol measurement (T0). Those with cognitive impairment (MMSE≤24, CDR of >0.5) at baseline assessment of cognition (T0), or missing alcohol or outcome data were excluded.	**Age:** yes*. **Sex**: yes. **SES**: education* (years). **Smoking:** no. **Co-morbidities**: Cumulative Illness Rating Scale* (CIRS; measures co-morbidities and includes cardiovascular disease but as part of an overall score among other conditions), diabetes, hypertension, BMI. **Baseline cognition**: yes.<br>Other: ApoE4 genotype*, Caucasian*.	**Measurement**: Single assessment at T0. Self-report questions administered at interview. Current: If "ever consumed >1 drink/week for >3 months", then asked about frequency ("average days per week" (1-2, 3-5, daily)), and amount ("average quantity in drinks per day" (1, 2-3, ≥4)), and how often they drank ≥4 per occasion. Lifetime: Quantity/ frequency questions asked for age '40-current', '19-39' and '0-18' years. Past drinkers: asked age when they quit. Recall: current to 80 years.<br>**Categories**: Rare/never (0 drinks/week for any 3 month period over lifetime), 2 drinking categories (<3 / <4 drinks/day for men/women; ≥3 / ≥4 drinks/day for men/women). Grams per drink not reported.	Cognitive tests administered in face-to-face interview by research personnel (Kaye 2011). The same method of assessment was used for all participants. No information about whether interviewer was aware of alcohol consumption status (blinding), but alcohol and cognitive measures seem to have been taken place at the same time.	Regression model of annual rates of change. The rates of change were taken from a linear mixed model that modelled log MMSE with subject-specific random effects and fixed effects rates. Alcohol was modelled as a categorical variable. The set of covariates adjusted for is not clear, but likely to have been: age, education, the Cumulative Illness Rating Scale, and expression of the apolipoprotein E, isoform 4 (Apo E4) genotype. An interaction was included between sex and alcohol consumption categories. Results extracted from Figure 2 (B).
**Overall risk of bias**<br>**Serious** due to confounding, selection of participants into the study, and classification of alcohol consumption	RoB in selection of participants into the study: **Serious.**<br>The lag time between initiating drinking and enrolment to the study means that those who previously experienced harmful outcomes associated with drinking may be excluded (because they died or were inaccessible, declined or were unable to participate, or did not meet health-related study eligibility criteria). Potential to bias through exclusion of drinkers with poorer health (including cognitive impairment) caused or exacerbated by alcohol consumption.	RoB due to confounding: **Serious.**<br>At least one known important domain (smoking) was not appropriately not controlled for. Co-morbidities were considered to be adjusted for based on adjustments for the CIRS.	RoB in classification of alcohol consumption: **Serious.**<br>Use of a single assessment to categorise levels of drinking brings a risk of misclassifying consumption (i.e. variation in drinking patterns over time are missed). Contamination of the non-drinking group with occasional/former drinkers is likely (e.g. lifetime measure based on long-term recall at age 80). Underestimation (through recall) or conscious under-reporting may amplify problems with misclassification.<br>RoB due to deviations from exposure as categorised through intervention: **Low**<br>Although plausible, intervention during alcohol measurement (e.g.an interviewer noting high alcohol intake), is unlikely to have an important effect on behaviour.	RoB in measurement of outcomes: **Low**<br>(i) The methods of outcome assessment were comparable across alcohol consumption groups.<br>(ii) The outcome assessors were likely to be unaware of alcohol consumption status of study participants.<br>(iii) Any error in measuring cognition is likely to be unrelated to alcohol consumption status.	RoB due to missing outcome data: **Low**. Data were reasonably complete<br>RoB in selection of the reported result: **No Information**<br>No protocol (or statistical analysis plan) identified from which to determine if measures or analyses reported were selected on the basis of results. Hence there is too little information to make a judgement.

Appendix 7. Data manipulation for each study

Study	Assumptions made in calculating the alcohol consumption (grams(g)/day)	Assumptions made in calculating the statistics used to compute the standardised mean difference
Arntzen 2010	Alcohol measured in glasses. Assumed 13.5g per glass. Alcohol content not specifically noted for Norway, so assumed an average of 12g and 15g (based on [34]). Dose calculated as the mid-point between categories. Upper bound of the largest dose category was assigned the lower bound of the largest dose category plus the width of the previous category.	Linear regression model with alcohol modelled as a categorical variable, adjusting for a set of covariates. Adjusted mean differences (and 95%CIs) compared with the referent category (0 to <10g/week) presented. Changed the reference category to non-drinker (current). Calculated pooled standard deviations from the confidence limits for each mean difference. These pooled standard deviations were used in the calculation of the SMD.
Downer 2015	Alcohol measured in drinks. Assumed 14g per drink. Dose was the reported mean dose within a category.	Linear regression model with alcohol modelled as a categorical variable, adjusting for a set of covariates. Adjusted mean differences (and 95% CIs) compared with the reference category (current non-drinker) presented. Calculated pooled standard deviations from the confidence limits for each mean difference. These pooled standard deviations were used in the calculation of the SMD.
Heffernan 2016	Alcohol measured in drinks. Assumed 10g per drink (based on Australian standard). The combined results for males and females were presented, but the alcohol intake within a category (e.g. 'low risk') varied by sex. Therefore, dose was calculated as a weighted average of mid-points for males and females, where the weights reflected the proportion of males and females in the category. Upper bound of the largest dose category was assigned the lower bound of the largest dose category plus the width of the previous category.	Logistic regression model with alcohol modelled as a categorical variable, adjusting for a set of covariates. Odds ratios (and 95% CIs) compared with the reference category (current non-drinker) presented. The reciprocal of the OR was computed so that the interpretation of the OR was in the same direction as the other studies (i.e. an OR > 1 means better cognition for exposure categories compared with the referent category). The ORs were converted to SMDs using the method of Chinn 2000. Standard deviations of 1 were assumed for each alcohol level. The variances of the SMDs were recalibrated (by reducing the sample sizes) so that when they were back transformed to the (log) OR scale, they yielded equivalent variances to the observed (log) OR variances.
Horvat 2015	Alcohol measured in grams. Dose calculated as the mid-point between categories. Upper bound of the largest dose category was assigned the lower bound of the largest dose category plus the width of the previous category.	Linear regression model with alcohol modelled as a categorical variable, adjusting for a set of covariates. Adjusted mean differences (95%CIs) compared with reference category (≥10 g/week and <10 g/day) presented. Changed the reference category to non-drinker (current). Calculated pooled standard deviations from the confidence limits for each mean difference. These pooled standard deviations were used in the calculation of the SMD.
Kesse-Guyot 2012	Alcohol measured in grams. Dose calculated as the mid-point between categories. Upper bound of the largest dose category was assigned the lower bound of the largest dose category plus the width of the previous category.	Analysis of covariance with alcohol modelled as a categorical variable, adjusting for a set of covariates. Adjusted mean differences (95%CIs) compared with reference category (≥ 20 g/day and <30 g/day) presented. Changed the reference category to non-drinker (current). Calculated pooled standard deviations from the confidence limits for each mean difference. These pooled standard deviations were used in the calculation of the SMD.

Kitamura 2017	Alcohol measured in grams. Dose calculated as the mid-point between categories. Upper bound of the largest dose category was assigned the lower bound of the largest dose category plus the width of the previous category.	Logistic regression model with alcohol modelled as a categorical variable, adjusting for a set of covariates. Odds ratios (and 95% CIs) compared with the reference category (current non-drinker) presented. The reciprocal of the OR was computed so that the interpretation of the OR was in the same direction as the other studies (i.e. an OR > 1 means better cognition for exposure categories compared with the referent category). The ORs were converted to SMDs using the method of Chinn 2000. Standard deviations of 1 were assumed for each alcohol level. The variances of the SMDs were recalibrated (by reducing the sample sizes) so that when they were back transformed to the (log) OR scale, they yielded equivalent variances to the observed (log) OR variances.
Sabia 2011	Alcohol measured in units. A standard unit is 10g – 12g, so assumed a mean of 11g. Dose calculated as the mid-point between categories. Upper bound of the largest dose category was assigned the lower bound of the largest dose category plus the width of the previous category.	Analysis of covariance with alcohol modelled as a categorical variable, adjusting for a set of covariates. Separate models fitted by education level (primary school, professional qualification, secondary school and more). Adjusted mean differences (95%CIs) compared with reference category (≥ 10 and <20 g/day) presented. Changed the reference category to non-drinker (current). Calculated pooled standard deviations from the confidence limits for each mean difference. Across the strata the mean differences and standard deviations were combined for each level of alcohol consumption. For a particular alcohol level, the mean difference was calculated as a weighted average of the three strata's mean differences, where the weights were the sample sizes. The standard deviations were calculated as the pooled standard deviation of the three standard deviations.
Sabia 2014	Alcohol measured in grams. Dose was the reported median dose within a category, or when not reported, calculated as the mid-point between categories.	Linear mixed model with alcohol modelled as a categorical variable, adjusting for a set of covariates. Adjusted mean differences (95%CIs) compared with reference category (≥10 g/week and <10 g/day) presented. Changed the reference category to non-drinker (current). Calculated pooled standard deviations from the confidence limits for each mean difference. These pooled standard deviations were used in the calculation of the SMD.
Stott 2008	Alcohol measured in units. Assumed 8g per unit (based on UK standard). Cut-offs in the paper are reported inconsistently. Have assumed the cut-offs outlined in the Statistical Analysis section. Dose calculated as the mid-point between categories. Upper bound of the largest dose category was assigned the lower bound of the largest dose category plus the width of the previous category.	Linear mixed model with alcohol modelled as a categorical variable, adjusting for a set of covariates. No information about how non-drinker was defined, so assumed zero for the dose-response analysis. Calculated pooled standard deviations from the confidence limits for each mean difference. These pooled standard deviations were used in the calculation of the SMD.
Richard 2017	Alcohol measured in drinks. Assumed 12g per drink. The combined results for males and females were presented, but the alcohol intake within a category (e.g. 'moderate') varied by sex and age ((i) men under 65, (ii) men over 65 and	Multinomial logistic regression model with alcohol modelled as a categorical variable, adjusting for a set of covariates. Sample size reduced to account for the fact that the results taken from a subset of the participants included in the multinomial logistic regression model (i.e. the fraction of the cohort that had an

	women). Assumed the categories for group (ii) since the mean age for each consumption group was approximately 70. Dose calculated as the mid-point between categories. Upper bound of the largest dose category was assigned the lower bound of the largest dose category plus the width of the previous category.	outcome of either cognitively healthy longevity or cognitively impaired longevity). The ORs were converted to SMDs using the method of Chinn 2000. Standard deviations of 1 were assumed for each alcohol level. The variances of the SMDs were recalibrated (by reducing the sample sizes) so that when they were back transformed to the (log) OR scale, they yielded equivalent variances to the observed (log) OR variances.

Appendix 8. Results from sensitivity analyses

Females

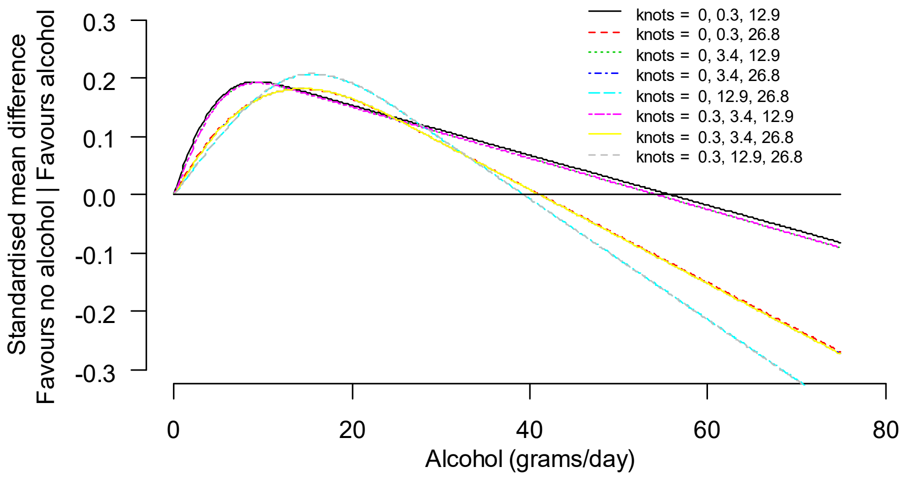

Figure 8.1 Sensitivity analysis of the pooled dose-response relationship between alcohol consumption (grams/day) and SMD using different locations of the three knots in the restricted cubic spline model. The current non-drinker served as the referent group.

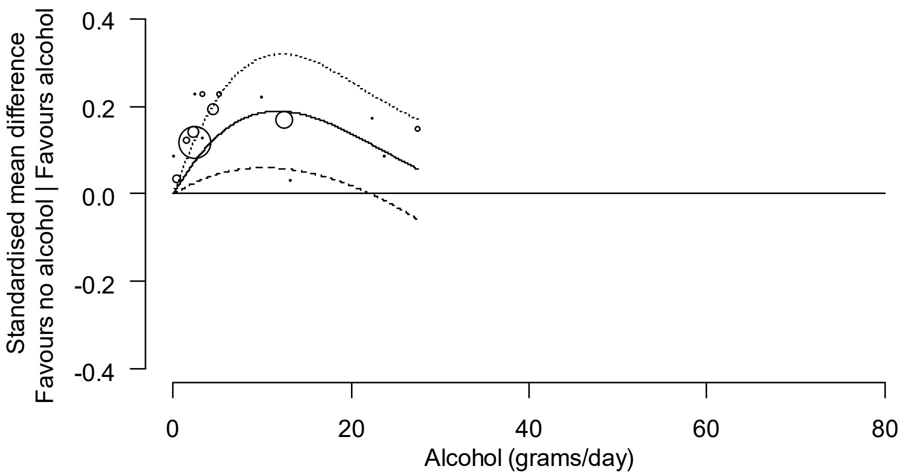

Figure 8.2 Sensitivity analysis of the pooled dose-response relationship between alcohol consumption (grams/day) and SMD with large alcohol consumption values from Kesse-Guyot 2012 (i.e. >30grams/day) removed. The current non-drinker served as the referent group.

Males

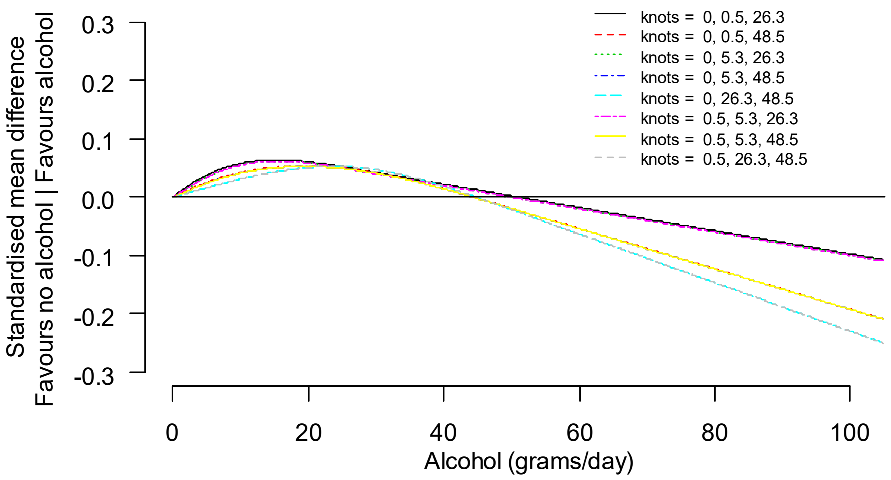

Figure 8.3 Sensitivity analysis of the pooled dose-response relationship between alcohol consumption (grams/day) and SMD using different locations of the three knots in the restricted cubic spline model. The current non-drinker served as the referent group.

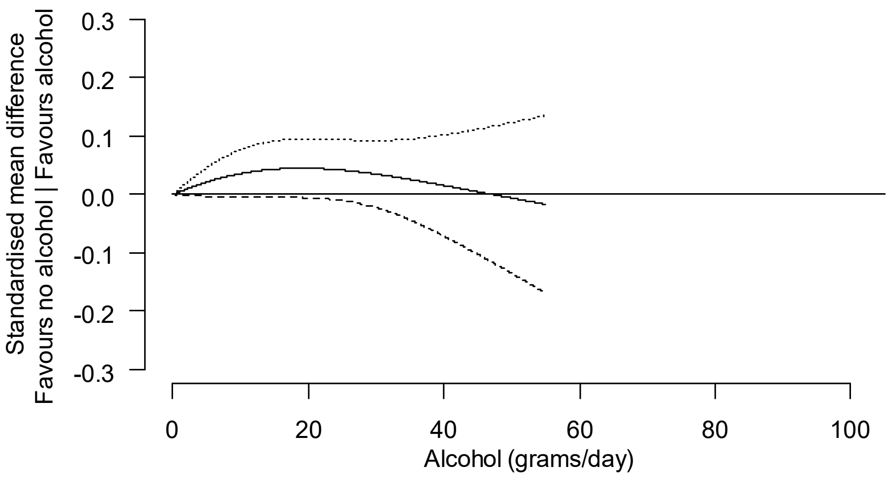

Figure 8.4. Sensitivity analysis of the pooled dose-response relationship between alcohol consumption (grams/day) and SMD with large alcohol consumption values from Kesse-Guyot 2012 (i.e. >70grams/day) removed. The current non-drinker served as the referent group.

Females and males

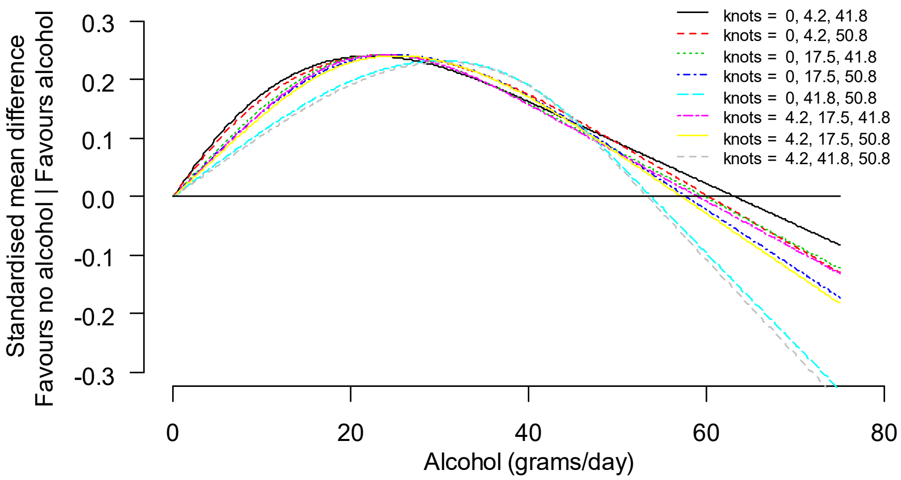

Figure 8.5 Sensitivity analysis of the pooled dose-response relationship between alcohol consumption (grams/day) and SMD using different locations of the three knots in the restricted cubic spline model. The current non-drinker served as the referent group.

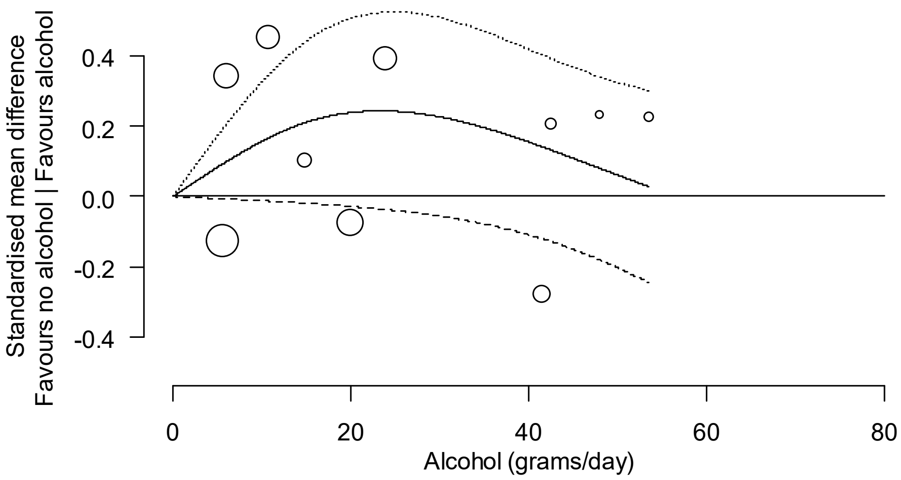

Figure 8.6 Sensitivity analysis of the pooled dose-response relationship between alcohol consumption (grams/day) and SMD with large alcohol consumption values from Kitamura 2017 (i.e. >55grams/day) removed. The current non-drinker served as the referent group.

Appendix 9. Reasons for exclusion of studies from the dose-response analyses

Study ID	Reason for exclusion from dose-response analysis
**Hogenkamp 2014**	Excluded because the data available for the dose-response analysis are unadjusted means and standard deviations. These statistics do adjust for baseline cognition (through the use of change scores) and age (by design - all participants are the same age at baseline), but do not adjust for other important potential confounders. There is some imbalance in potential confounders at baseline (Table 1).
**Lang 2007a**	Excluded because the data (ORs and confidence intervals) are only presented in a figure (2). If extracted, a dose-response analysis could be undertaken.
**Piumatti 2018**	Excluded because the data available for the dose-response are unadjusted means and standard deviations (Table 1), or results from a restricted cubic spline model. The former results are not re-analysed because they adjust for no confounders. The latter results cannot be included in any dose-response synthesis with the other studies because of the data presented. Further, the outcome - reaction time - while providing a measure of cognition differs to the other studies.
**Samieri 2013a**	Excluded because not all the data are available to undertake a dose-response analysis. Specifically, while mean differences and confidence intervals are provided (Table 2, pg 2), the breakdown of the total sample size (6174) is not provided by alcohol consumption level. This precludes the calculation of standardised mean differences unless some assumptions are made. A single global cognitive function score is the mean of five z-scores (mean 0, variance 1). Thus, the mean and variance of this variable is 0 and 1/5. For the analysis, three measures of cognitive function are averaged, yielding a mean and variance of 0 and 1/(3*5) = 1/15. There is the potential to use the standard deviation calculated from this variance (sqrt(var)) to undertake the dose-response meta-analysis, however we would have to assume the sample sizes were the same across the groups, which is unlikely to be true, and so, the standard errors would be incorrect.
**Solfrizzi 2007**	Excluded because the metrics presented (from Cox proportional hazards model) are hazard ratios (HRs). When the event is rare (in this case incident mild cognitive impairment), then it may be reasonable to assume that the HRs will yield similar values to Risk Ratios (RR) and in turn ORs (see Sutradhar. Annals of Epidemiology 28 (2018) 54e57) in which case, the data could be reanalysed for the dose-response analysis. The incident cases of MCI during the follow-up period was 105 or 1445 participants. However, we have chosen not to re-analyse this data for the dose-response analysis given the assumptions that would need to be made.
**Wardzala 2018**	Excluded since it is not clear what effects are being reported from the analysis that could potentially contribute to the dose-response. Specifically, in the online supplement 3, it is not clear whether in the columns 'Drinking-Gender Specific Interactions on Longitudinal Rates', the reported effects are (for example) the average difference in MMSE in the moderate drinkers versus rare/never drinkers at a particular point in time, or the difference in the slope over time between the moderate drinkers versus the rare/never drinkers for a unit of time. Further, the unit of time used in the model is not specified in the statistical analysis section. [JM has contact authors (29/10/2018) to ask some further questions regarding the timing of the alcohol measurement relative to cognition and how to interpret the results presented in the online supplement. No response received as at 23/11/2018.] Note that the results presented in the summary results table, pertain to the analyses that investigate whether the rate of change over time differs across the alcohol consumption groups (that is, those presented in section 'Decline profiles for moderate drinking men and women' (pg 5)).

Appendix 10. Alphabetical reference list of all studies excluded following full text review

1. (2012). "ALCOHOL intake in the elderly affects risk of cognitive decline and dementia." American Journal of Alzheimer's Disease & Other Dementias 27(5): 355-356.
2. (2016). ""Cognitive, emotion control, and motor performance of adolescents in the NCANDA study: Contributions from alcohol consumption, age, sex, ethnicity, and family history of addiction": Correction to Sullivan et al. (2016).[Erratum for Neuropsychology. 2016 May;30(4):449-73; PMID: 26752122]." Neuropsychology 30(7): 829.
3. Aguirre-Acevedo, D. C., F. Lopera, E. Henao, V. Tirado, C. Munoz, M. Giraldo, S. I. Bangdiwala, E. M. Reiman, P. N. Tariot, J. B. Langbaum, Y. T. Quiroz and F. Jaimes (2016). "Cognitive Decline in a Colombian Kindred With Autosomal Dominant Alzheimer Disease: A Retrospective Cohort Study." JAMA Neurology 73(4): 431-438.
4. Almeida, O. P., G. J. Hankey, B. B. Yeap, J. Golledge and L. Flicker (2014). "ALCOHOL consumption and cognitive impairment in older men: a mendelian randomization study." Neurology 82(12): 1038-1044.
5. Assmann, K. E., C. Lassale, V. A. Andreeva, C. Jeandel, S. Hercberg, P. Galan and E. Kesse-Guyot (2015). "A healthy dietary pattern at midlife, combined with a regulated energy intake, is related to increased odds for healthy aging." Journal of Nutrition 145(9): 2139-2145.
6. Au Yeung, S. L., C. Jiang, W. Zhang, T. H. Lam, K. K. Cheng, G. M. Leung and C. M. Schooling (2010). "Moderate alcohol use and cognitive function in the Guangzhou Biobank Cohort study." Annals of Epidemiology 20(12): 873-882.
7. Au Yeung, S. L., C. Q. Jiang, K. K. Cheng, B. Liu, W. S. Zhang, T. H. Lam, G. M. Leung and C. M. Schooling (2012). "Evaluation of moderate ALCOHOL use and cognitive function among men using a Mendelian randomization design in the Guangzhou biobank cohort study." American Journal of Epidemiology 175(10): 1021-1028.
8. Au Yeung, S. L., G. M. Leung, W. M. Chan, Y. F. Hui, T. H. Lam and C. M. Schooling (2011). "Moderate alcohol use and cognitive function in an elderly Chinese cohort." Journal of the American Geriatrics Society 59(1): 172-174.
9. Ballard, C. and I. Lang (2018). "Alcohol and dementia: a complex relationship with potential for dementia prevention." The Lancet Public Health 3(3): e103-e104.
10. Banz, B. C. (2015). "An evaluation of executive functions, cognitive control and a neurocognitive profile of college binge." Dissertation Abstracts International: Section B: The Sciences and Engineering 76(1-B(E)): No-Specified.
11. Barnes, D. E., J. A. Cauley, L.-Y. Lui, H. A. Fink, C. McCulloch, K. L. Stone and K. Yaffe (2007). "Women who maintain optimal cognitive function into old age." Journal of the American Geriatrics Society 55(2): 259-264.
12. Basta, M., A. Bertsias, E. Koutentaki, I. Zaganas, P. Simos, G. Duijker, S. Panagiotakis, D. Boumpas, C. Tziraki, C. Pionis and A. Vgontzas (2015). "Insomnia symptoms in a large elderly greek population are associated with cognitive decline." Sleep 38(SUPPL. 1): A398.
13. Bates, M. E., J. F. Buckman, G. T. Voelbel, D. Eddie and J. Freeman (2013). "The mean and the individual: Integrating variable-centered and person-centered analyses of cognitive recovery in patients with substance use disorders." Frontiers in Psychiatry 4.
14. Bell, C. L., R. Chen, K. Masaki, P. Yee, Q. He, J. Grove, T. Donlon, J. D. Curb, D. C. Willcox, L. W. Poon and B. J. Willcox (2014). "Late-life factors associated with healthy aging in older men." Journal of the American Geriatrics Society 62(5): 880-888.
15. Berntsen, S., J. Kragstrup, V. Siersma, G. Waldemar and F. B. Waldorff (2015). "Alcohol consumption and mortality in patients with mild Alzheimer's disease: a prospective cohort study." BMJ Open 5(12): e007851.
16. Beydoun, M. A., A. A. Gamaldo, H. A. Beydoun, T. Tanaka, K. L. Tucker, S. A. Talegawkar, L. Ferrucci and A. B. Zonderman (2014). "Caffeine and ALCOHOL intakes and overall nutrient adequacy are associated with longitudinal cognitive performance among U.S. adults." Journal of Nutrition 144(6): 890-901.

17. Binder, N., L. Manderscheid and M. Schumacher (2017). "The combined association of alcohol consumption with dementia risk is likely biased due to lacking account of death cases." European Journal of Epidemiology 32(7): 627-629.
18. Booker, A., L. E. Jacob, M. Rapp, J. Bohlken and K. Kostev (2016). "Risk factors for dementia diagnosis in German primary care practices." International Psychogeriatrics 28(7): 1059-1065.
19. Boot, B. P., C. F. Orr, J. E. Ahlskog, T. J. Ferman, R. Roberts, V. S. Pankratz, D. W. Dickson, J. Parisi, J. A. Aakre, Y. E. Geda, D. S. Knopman, R. C. Petersen and B. F. Boeve (2013). "Risk factors for dementia with Lewy bodies: a case-control study." Neurology 81(9): 833-840.
20. Bos, I., S. J. Vos, L. Frolich, J. Kornhuber, J. Wiltfang, W. Maier, O. Peters, E. Ruther, S. Engelborghs, E. Niemantsverdriet, E. E. De Roeck, M. Tsolaki, Y. Freund-Levi, P. Johannsen, R. Vandenberghe, A. Lleo, D. Alcolea, G. B. Frisoni, S. Galluzzi, F. Nobili, S. Morbelli, A. Drzezga, M. Didic, B. N. van Berckel, E. Salmon, C. Bastin, S. Dauby, I. Santana, I. Baldeiras, A. de Mendonca, D. Silva, A. Wallin, A. Nordlund, P. M. Coloma, A. Wientzek, M. Alexander, G. P. Novak, M. F. Gordon, I. Alzheimer's Disease Neuroimaging, A. K. Wallin, H. Hampel, H. Soininen, S.-K. Herukka, P. Scheltens, F. R. Verhey and P. J. Visser (2017). "The frequency and influence of dementia risk factors in prodromal Alzheimer's disease." Neurobiology of Aging 56: 33-40.
21. Braun, A. (2015). "Binge drinking's cognitive and emotional correlates: A multi-definitional investigation." Dissertation Abstracts International: Section B: The Sciences and Engineering 76(1-B(E)): No-Specified.
22. Brion, M., F. D'Hondt, A.-L. Pitel, B. Lecomte, M. Ferauge, P. de Timary and P. Maurage (2017). "Executive functions in alcohol-dependence: A theoretically grounded and integrative exploration." Drug & Alcohol Dependence 177: 39-47.
23. Britton, A., M. Shipley, A. Singh-Manoux and M. G. Marmot (2008). "Successful aging: the contribution of early-life and midlife risk factors." Journal of the American Geriatrics Society 56(6): 1098-1105.
24. Brown, S. A., T. Brumback, K. Tomlinson, K. Cummins, W. K. Thompson, B. J. Nagel, M. D. De Bellis, S. R. Hooper, D. B. Clark, T. Chung, B. P. Hasler, I. M. Colrain, F. C. Baker, D. Prouty, A. Pfefferbaum, E. V. Sullivan, K. M. Pohl, T. Rohlfing, B. N. Nichols, W. Chu and S. F. Tapert (2015). "The National Consortium on Alcohol and NeuroDevelopment in Adolescence (NCANDA): A Multisite Study of Adolescent Development and Substance Use." Journal of Studies on Alcohol & Drugs 76(6): 895-908.
25. Brumback, T., D. Cao, P. McNamara and A. King (2017). "Alcohol-induced performance impairment: a 5-year re-examination study in heavy and light drinkers." Psychopharmacology 234(11): 1749-1759.
26. Buttaro, M. A. (2008). "Vascular risk factors and cognitive functioning in normal elderly." Dissertation Abstracts International: Section B: The Sciences and Engineering 68(8-B): 5600.
27. Byeon, H., Y. Lee, S. Y. Lee, K. S. Lee, S. Y. Moon, H. Kim, C. H. Hong, S. J. Son and S. H. Choi (2015). "Association of alcohol drinking with verbal and visuospatial memory impairment in older adults: Clinical Research Center for Dementia of South Korea (CREDOS) study." International Psychogeriatrics 27(3): 455-461.
28. Cairney, S., A. Clough, M. Jaragba and P. Maruff (2007). "Cognitive impairment in Aboriginal people with heavy episodic patterns of alcohol use." Addiction 102(6): 909-915.
29. Cartier, J. L., S. C. Kukreja and E. Barengolts (2017). "LOWER SERUM 25-HYDROXYVITAMIN D IS ASSOCIATED WITH OBESITY BUT NOT COMMON CHRONIC CONDITIONS: AN OBSERVATIONAL STUDY OF AFRICAN AMERICAN AND CAUCASIAN MALE VETERANS." Endocrine Practice 23(3): 271-278.
30. Cations, M., B. Draper, L.-F. Low, K. Radford, J. Trollor, H. Brodaty, P. Sachdev, P. Gonski, G. A. Broe and A. Withall (2018). "Non-Genetic Risk Factors for Degenerative and Vascular Young Onset Dementia: Results from the INSPIRED and KGOW Studies." Journal of Alzheimer's Disease 62(4): 1747-1758.
31. Ceccanti, M., D. Hamilton, G. Coriale, V. Carito, L. Aloe, G. Chaldakov, M. Romeo, M. Ceccanti, A. Iannitelli and M. Fiore (2015). "Spatial learning in men undergoing alcohol detoxification." Physiology & Behavior 149: 324-330.
32. Chanraud, S. and C. Bernard (2015). "Neuroimaging and alcoholism." Annales Medico-Psychologiques 173(3): 249-254.
33. Chen, L. Y., Y. H. Wu, C. Y. Huang, L. K. Liu, A. C. Hwang, L. N. Peng, M. H. Lin and L. K. Chen (2017). "Predictive factors for dementia and cognitive impairment among residents living in the veterans' retirement

communities in Taiwan: Implications for cognitive health promotion activities." Geriatrics and Gerontology International 17(Supplement 1): 7-13.
34. Chen, S. Y. and S. T. Tsai (2010). "The epidemiology of Parkinson's disease." Tzu Chi Medical Journal 22(2): 73-81.
35. Chen, X., Y. Huang and H. G. Cheng (2012). "Lower intake of vegetables and legumes associated with cognitive decline among illiterate elderly Chinese: a 3-year cohort study." Journal of Nutrition, Health & Aging 16(6): 549-552.
36. Chen, Y., A. R. Sillaire, J. Dallongeville, E. Skrobala, D. Wallon, B. Dubois, D. Hannequin, F. Pasquier and Y. O. D. s. g. Lille (2017). "Low Prevalence and Clinical Effect of Vascular Risk Factors in Early-Onset Alzheimer's Disease." Journal of Alzheimer's Disease 60(3): 1045-1054.
37. Cherbuin, N., C. Reglade-Meslin, R. Kumar, P. Jacomb, S. Easteal, H. Christensen, P. Sachdev and K. J. Anstey (2009). "Risk factors of transition from normal cognition to mild cognitive disorder: the PATH through Life Study." Dementia & Geriatric Cognitive Disorders 28(1): 47-55.
38. Chiang, C.-J., P.-K. Yip, S.-C. Wu, C.-S. Lu, C.-W. Liou, H.-C. Liu, C.-K. Liu, C.-H. Chu, C.-S. Hwang, S.-F. Sung, Y.-D. Hsu, C.-C. Chen, S.-I. Liu, S.-H. Yan, C.-S. Fong, S.-F. Chang, S.-L. You and C.-J. Chen (2007). "Midlife risk factors for subtypes of dementia: a nested case-control study in Taiwan." American Journal of Geriatric Psychiatry 15(9): 762-771.
39. Choi, I.-G., S.-I. Woo, H. J. Kim, D.-J. Kim, B. L. Park, H. S. Cheong, C. F. A. Pasaje, T. J. Park, J. S. Bae, Y. G. Chai and H. D. Shin (2010). "Lack of association between PRNP M129V polymorphism and multiple sclerosis, mild cognitive impairment, alcoholism and schizophrenia in a Korean population." Disease Markers 28(5): 315-321.
40. Contador, I., F. Bermejo-Pareja, V. Puertas-Martin and J. Benito-Leon (2015). "Childhood and Adulthood Rural Residence Increases the Risk of Dementia: NEDICES Study." Current Alzheimer Research 12(4): 350-357.
41. Corley, J., X. Jia, C. E. Brett, A. J. Gow, J. M. Starr, J. A. M. Kyle, G. McNeill and I. J. Deary (2011). "Alcohol intake and cognitive abilities in old age: the Lothian Birth Cohort 1936 study." Neuropsychology 25(2): 166-175.
42. Czapla, M., J. J. Simon, B. Richter, M. Kluge, H. C. Friederich, S. Herpertz, K. Mann, S. C. Herpertz and S. Loeber (2015). "The impact of cognitive impairment and impulsivity on relapse of alcohol-dependent patients: Implications for psychotherapeutic treatment." Addiction Biology.
43. Davis, B. J. K., J.-S. Vidal, M. Garcia, T. Aspelund, M. A. van Buchem, M. K. Jonsdottir, S. Sigurdsson, T. B. Harris, V. Gudnason and L. J. Launer (2014). "The ALCOHOL paradox: light-to-moderate ALCOHOL consumption, cognitive function, and brain volume." Journals of Gerontology Series A-Biological Sciences & Medical Sciences 69(12): 1528-1535.
44. Deckers, K., S. Kohler, M. van Boxtel, F. Verhey, C. Brayne, J. Fleming and C. C. s. collaboration (2017). "Lack of associations between modifiable risk factors and dementia in the very old: findings from the Cambridge City over-75s cohort study." Aging & Mental Health: 1-7.
45. Dimitrov, I., I. Milanov, N. Deleva and B. Ivanov (2011). "Risk factors for dementia in a community-based sample of the Bulgarian urban population." Archives of the Balkan Medical Union 46(2): 147-149.
46. Dingwall, K. M., P. Maruff and S. Cairney (2011). "Similar profile of cognitive impairment and recovery for Aboriginal Australians in treatment for episodic or chronic alcohol use." Addiction 106(8): 1419-1426.
47. Durazzo, T. C., D. L. Pennington, T. P. Schmidt and D. J. Meyerhoff (2014). "Effects of cigarette smoking history on neurocognitive recovery over 8 months of abstinence in ALCOHOL-dependent individuals." Alcoholism: Clinical & Experimental Research 38(11): 2816-2825.
48. Eisenstein, A. R. (2012). "Individual and additive effects of lifestyle behaviors on cognition: A longitudinal study." Dissertation Abstracts International: Section B: The Sciences and Engineering 73(2-B): 914.
49. Ellingson, J. M., K. A. Fleming, A. Verges, B. D. Bartholow and K. J. Sher (2014). "Working memory as a moderator of impulsivity and ALCOHOL involvement: testing the cognitive-motivational theory of ALCOHOL use with prospective and working memory updating data." Addictive Behaviors 39(11): 1622-1631.

50. Elwood, P., J. Galante, J. Pickering, S. Palmer, A. Bayer, Y. Ben-Shlomo, M. Longley and J. Gallacher (2013). "Healthy lifestyles reduce the incidence of chronic diseases and dementia: evidence from the Caerphilly cohort study." PLoS ONE [Electronic Resource] 8(12): e81877.
51. Fama, R., E. V. Sullivan, S. A. Sassoon, A. Pfefferbaum and N. M. Zahr (2016). "Impairments in Component Processes of Executive Function and Episodic Memory in Alcoholism, HIV Infection, and HIV Infection with Alcoholism Comorbidity." Alcoholism: Clinical & Experimental Research 40(12): 2656-2666.
52. Fan, X., A. O'Donnell, S. P. Singh, R. Pungan and L. C. Perlmuter (2008). "Light to moderate alcohol drinking is associated with higher cognitive function in males with type 2 diabetes." Experimental Aging Research 34(2): 126-137.
53. Fein, G. and S. McGillivray (2007). "Cognitive performance in long-term abstinent elderly alcoholics." Alcoholism: Clinical & Experimental Research 31(11): 1788-1799.
54. Feng, Q., J. Son and Y. Zeng (2015). "Prevalence and correlates of successful ageing: a comparative study between China and South Korea." European Journal of Ageing 12(2): 83-94.
55. Fluharty, M. E., J. Heron and M. R. Munafo (2017). "Longitudinal associations of social cognition and substance use in childhood and early adolescence: findings from the Avon Longitudinal Study of Parents and Children." European Child & Adolescent Psychiatry: 20.
56. Franken, I. H. A., M. Luijten, F. M. van der Veen and J. W. van Strien (2017). "Cognitive control in young heavy drinkers: An ERP study." Drug & Alcohol Dependence 175: 77-83.
57. Fung, A. W. T., G. T. Y. Leung and L. C. W. Lam (2011). "Modulating factors that preserve cognitive function in healthy ageing." East Asian Archives of Psychiatry 21(4): 152-156.
58. Ganguli, M., B. Fu, B. E. Snitz, F. W. Unverzagt, D. A. Loewenstein, T. F. Hughes and C.-C. H. Chang (2014). "Vascular risk factors and cognitive decline in a population sample." Alzheimer Disease & Associated Disorders 28(1): 9-15.
59. Ganguli, M., B. Fu, B. E. Snitz, T. F. Hughes and C.-C. H. Chang (2013). "Mild cognitive impairment: incidence and vascular risk factors in a population-based cohort." Neurology 80(23): 2112-2120.
60. Ganguli, M., C.-W. Lee, B. E. Snitz, T. F. Hughes, E. McDade and C.-C. H. Chang (2015). "Rates and risk factors for progression to incident dementia vary by age in a population cohort." Neurology 84(1): 72-80.
61. Garcia, A. M., N. Ramon-Bou and M. Porta (2010). "Isolated and joint effects of tobacco and alcohol consumption on risk of Alzheimer's disease." Journal of Alzheimer's Disease 20(2): 577-586.
62. Gelber, R. P., H. Petrovitch, K. H. Masaki, R. D. Abbott, G. W. Ross, L. J. Launer and L. R. White (2012). "Lifestyle and the risk of dementia in Japanese-american men." Journal of the American Geriatrics Society 60(1): 118-123.
63. Gow, A. J., W. Johnson, A. Pattie, M. C. Whiteman, J. Starr and I. J. Deary (2008). "Mental ability in childhood and cognitive aging." Gerontology 54(3): 177-186.
64. Groot, R. H. M., M. L. van Dijk and P. A. Kirschner (2015). "Cohort profile of the GOALS study: A large-scale research of physical activity in Dutch students." British Journal of Educational Technology 46(5): 947-952.
65. Gvozdenovic, L. and A. Antanaskovic (2015). "History of alcohol abuse after major non-cardiac surgery and postoperative cognitive dysfunction." European Journal of Internal Medicine 26(9): e51.
66. Hagger-Johnson, G., S. Sabia, E. J. Brunner, M. Shipley, M. Bobak, M. Marmot, M. Kivimaki and A. Singh-Manoux (2013). "Combined impact of smoking and heavy ALCOHOL use on cognitive decline in early old age: Whitehall II prospective cohort study." British Journal of Psychiatry 203(2): 120-125.
67. Hai, S., B. Dong, Y. Liu and Y. Zou (2012). "Occurrence and risk factors of mild cognitive impairment in the older Chinese population: a 3-year follow-up study." International Journal of Geriatric Psychiatry 27(7): 703-708.
68. Hajek, A. and H.-H. Konig (2016). "Longitudinal Predictors of Functional Impairment in Older Adults in Europe--Evidence from the Survey of Health, Ageing and Retirement in Europe." PLoS ONE [Electronic Resource] 11(1): e0146967.
69. Handing, E. P., R. Andel, P. Kadlecova, M. Gatz and N. L. Pedersen (2015). "Midlife Alcohol Consumption and Risk of Dementia Over 43 Years of Follow-Up: A Population-Based Study From the Swedish Twin Registry." Journals of Gerontology Series A-Biological Sciences & Medical Sciences 70(10): 1248-1254.

70. Hao, L., X. Wang, L. Zhang, Y. Xing, Q. Guo, X. Hu, B. Mu, Y. Chen, G. Chen, J. Cao, X. Zhi, J. Liu, X. Li, L. Yang, J. Li, W. Du, Y. Sun, T. Wang, Z. Liu, Z. Liu, X. Zhao, H. Li, Y. Yu, X. Wang, J. Jia and Y. Han (2017). "Prevalence, Risk Factors, and Complaints Screening Tool Exploration of Subjective Cognitive Decline in a Large Cohort of the Chinese Population." Journal of Alzheimer's Disease 60(2): 371-388.
71. Harper, J., S. M. Malone and W. G. Iacono (2017). "Testing the effects of adolescent alcohol use on adult conflict-related theta dynamics." Clinical Neurophysiology 128(11): 2358-2368.
72. Harvanko, A. M., B. L. Odlaug, L. R. N. Schreiber and J. E. Grant (2012). "Cognitive task performance and frequency of ALCOHOL usage in young adults." Journal of Addiction Medicine 6(2): 106-111.
73. Harwood, D. G., A. Kalechstein, W. W. Barker, S. Strauman, P. St George-Hyslop, C. Iglesias, D. Loewenstein and R. Duara (2010). "The effect of alcohol and tobacco consumption, and apolipoprotein E genotype, on the age of onset in Alzheimer's disease." International Journal of Geriatric Psychiatry 25(5): 511-518.
74. Hatchard, T., A. M. Smith, R. E. Halchuk, C. A. Longo, P. A. Fried, M. J. Hogan and I. Cameron (2015). "Effects of low-level alcohol use on cognitive interference: an fMRI study in young adults." Alcohol 49(1): 7-13.
75. Hawkins, L. A., S. Kilian, A. Firek, T. M. Kashner, C. J. Firek and H. Silvet (2012). "Cognitive impairment and medication adherence in outpatients with heart failure." Heart & Lung 41(6): 572-582.
76. Heikkinen, N., E. Niskanen, M. Kononen, T. Tolmunen, V. Kekkonen, P. Kivimaki, H. Tanila, E. Laukkanen and R. Vanninen (2017). "Alcohol consumption during adolescence is associated with reduced grey matter volumes." Addiction 112(4): 604-613.
77. Heward, J., L. Stone, S.-M. Paddick, S. Mkenda, W. K. Gray, C. L. Dotchin, J. Kissima, C. Collingwood, B. Swai and R. W. Walker (2018). "A longitudinal study of cognitive decline in rural Tanzania: rates and potentially modifiable risk factors." International Psychogeriatrics: 1-11.
78. Heymann, D., Y. Stern, S. Cosentino, O. Tatarina-Nulman, J. N. Dorrejo and Y. Gu (2016). "The Association Between Alcohol Use and the Progression of Alzheimer's Disease." Current Alzheimer Research 13(12): 1356-1362.
79. Holst, C., J. S. Tolstrup, H. J. Sorensen and U. Becker (2017). "Alcohol dependence and risk of somatic diseases and mortality: a cohort study in 19002 men and women attending alcohol treatment." Addiction 112(8): 1358-1366.
80. Houston, R. J., J. L. Derrick, K. E. Leonard, M. Testa, B. M. Quigley and A. Kubiak (2014). "Effects of heavy drinking on executive cognitive functioning in a community sample." Addictive Behaviors 39(1): 345-349.
81. Hsu, W.-C., A. C. Tsai, Y.-C. Chen and J.-Y. Wang (2017). "Predicted factors for older Taiwanese to be healthy octogenarians: Results of an 18-year national cohort study." Geriatrics & gerontology international 17(12): 2579-2585.
82. Huang, C.-C., J.-D. Lee, D.-C. Yang, H.-I. Shih, C.-Y. Sun and C.-M. Chang (2017). "Associations Between Geriatric Syndromes and Mortality in Community-Dwelling Elderly: Results of a National Longitudinal Study in Taiwan." Journal of the American Medical Directors Association 18(3): 246-251.
83. Huntley, J., A. Corbett, K. Wesnes, H. Brooker, R. Stenton, A. Hampshire and C. Ballard (2018). "Online assessment of risk factors for dementia and cognitive function in healthy adults." International Journal of Geriatric Psychiatry 33(2): e286-e293.
84. Hurstak, E., J. K. Johnson, L. Tieu, D. Guzman, C. Ponath, C. T. Lee, C. W. Jamora and M. Kushel (2017). "Factors associated with cognitive impairment in a cohort of older homeless adults: Results from the HOPE HOME study." Drug & Alcohol Dependence 178: 562-570.
85. Jacob, L., J. Bohlken and K. Kostev (2017). "Risk Factors for Mild Cognitive Impairment in German Primary Care Practices." Journal of Alzheimer's Disease 56(1): 379-384.
86. Jones, S. B. (2016). "Association of mid-life alcohol consumption with stroke and cognitive decline in the Atherosclerosis Risk in Communities Study." Dissertation Abstracts International: Section B: The Sciences and Engineering 77(1-B(E)): No-Specified.
87. Jurk, S., E. Mennigen, T. Goschke and M. N. Smolka (2016). "Low-level alcohol consumption during adolescence and its impact on cognitive control development." Addiction Biology: No-Specified.

88. Kalapatapu, R. K., K. L. Delucchi, S. Wang, J. D. Harbison, E. E. Nelson and J. H. Kramer (2016). "Substance use history in behavioral-variant frontotemporal dementia versus primary progressive aphasia." Journal of Addictive Diseases 35(1): 36-41.
89. Kim, M. and J.-M. Park (2017). "Factors affecting cognitive function according to gender in community-dwelling elderly individuals." Epidemiology and health 39: e2017054.
90. Kim, S., Y. Kim and S. M. Park (2016). "Association between alcohol drinking behaviour and cognitive function: results from a nationwide longitudinal study of South Korea." BMJ Open 6(4): e010494.
91. Kimm, H., P. H. Lee, Y. J. Shin, K. S. Park, J. Jo, Y. Lee, H. C. Kang and S. H. Jee (2011). "Mid-life and late-life vascular risk factors and dementia in Korean men and women." Archives of Gerontology & Geriatrics 52(3): e117-122.
92. Kimura, S., T. Ogata, J. Watanabe, T. Inoue and Y. Tsuboi (2017). "Does cerebral large-artery disease contribute to cognitive impairment?" eNeurologicalSci 8: 5-8.
93. Kitamura, K., Y. Watanabe, K. Nakamura, K. Sanpei, M. Wakasugi, A. Yokoseki, O. Onodera, T. Ikeuchi, R. Kuwano, T. Momotsu, I. Narita and N. Endo (2016). "Modifiable Factors Associated with Cognitive Impairment in 1,143 Japanese Outpatients: The Project in Sado for Total Health (PROST)." Dementia and Geriatric Cognitive Disorders Extra 6(2): 341-349.
94. Klaming, R., J. Annese, D. J. Veltman and H. C. Comijs (2017). "Episodic memory function is affected by lifestyle factors: a 14-year follow-up study in an elderly population." Aging Neuropsychology & Cognition 24(5): 528-542.
95. Kuzma, E., D. J. Llewellyn, K. M. Langa, R. B. Wallace and I. A. Lang (2014). "History of ALCOHOL use disorders and risk of severe cognitive impairment: a 19-year prospective cohort study." American Journal of Geriatric Psychiatry 22(10): 1047-1054.
96. Lambert, M. E. (2016). "Differences in neurocognitive functioning associated with alcohol consumption in a multiethnic rural cohort: A Project FRONTIER study." Applied Neuropsychology Adult 23(5): 372-378.
97. Langballe, E. M., H. Ask, J. Holmen, E. Stordal, I. Saltvedt, G. Selbaek, A. Fikseaunet, S. Bergh, P. Nafstad and K. Tambs (2015). "Alcohol consumption and risk of dementia up to 27 years later in a large, population-based sample: the HUNT study, Norway." European Journal of Epidemiology 30(9): 1049-1056.
98. Langberg, J. M., M. R. Dvorsky, K. L. Kipperman, S. J. Molitor and L. D. Eddy (2015). "Alcohol Use Longitudinally Predicts Adjustment and Impairment in College Students with ADHD: The Role of Executive Functions." Psychology of Addictive Behaviors 29(2): 444-454.
99. Larsson, S. C., M. Traylor, R. Malik, M. Dichgans, S. Burgess, H. S. Markus and o. b. o. t. I. G. o. A. s. P. CoStream Consortium (2017). "Modifiable pathways in Alzheimer's disease: Mendelian randomisation analysis." BMJ 359: j5375.
100. Latvala, A., A. E. Castaneda, J. Perala, S. I. Saarni, T. Aalto-Setala, J. Lonnqvist, J. Kaprio, J. Suvisaari and A. Tuulio-Henriksson (2009). "Cognitive functioning in substance abuse and dependence: a population-based study of young adults." Addiction 104(9): 1558-1568.
101. Latvala, A., A. Tuulio-Henriksson, D. M. Dick, E. Vuoksimaa, J. Suvisaari, R. J. Viken, J. Kaprio and R. J. Rose (2009). "Cognitive functioning and alcohol dependence symptoms in young adulthood: Investigating the association in finnish twins." Behavior Genetics 39(6): 666.
102. Lee, H., S. Park, K. Lim, K. Lim, Y. Park and J. Jang (2016). "Association between lifestyle and cognitive impairment among women aged 65 years and over in the Republic of Korea." Educational Gerontology 42(3): 198-208.
103. Levy, B., E. Manove and R. D. Weiss (2012). "Recovery of cognitive functioning in patients with co-occurring bipolar disorder and ALCOHOL dependence during early remission from an acute mood episode." Annals of Clinical Psychiatry 24(2): 143-154.
104. Lo, A. H. Y., R. J. Woodman, N. A. Pachana, G. J. Byrne and P. S. Sachdev (2014). "Associations between lifestyle and cognitive function over time in women aged 40-79 years." Journal of Alzheimer's Disease 39(2): 371-383.

105. Lobo, E., C. Dufouil, G. Marcos, B. Quetglas, P. Saz, E. Guallar, A. Lobo and Z. Workgroup (2010). "Is there an association between low-to-moderate alcohol consumption and risk of cognitive decline?" American Journal of Epidemiology 172(6): 708-716.
106. Lopez-Caneda, E., F. Cadaveira, A. Crego, A. Gomez-Suarez, M. Corral, M. Parada, F. Caamano-Isorna and S. Rodriguez Holguin (2012). "Hyperactivation of right inferior frontal cortex in young binge drinkers during response inhibition: a follow-up study." Addiction 107(10): 1796-1808.
107. Lu, D., S. Ren, J. Zhang and D. Sun (2016). "Vascular risk factors aggravate cognitive impairment in first-ever young ischaemic stroke patients." European Journal of Neurology 23(5): 940-947.
108. Luck, T., M. Luppa, S. Briel, H. Matschinger, H.-H. Konig, S. Bleich, A. Villringer, M. C. Angermeyer and S. G. Riedel-Heller (2010). "Mild cognitive impairment: incidence and risk factors: results of the leipzig longitudinal study of the aged." Journal of the American Geriatrics Society 58(10): 1903-1910.
109. Lyu, J. and S. H. Lee (2014). "ALCOHOL consumption and cognitive impairment among Korean older adults: does gender matter?" International Psychogeriatrics 26(2): 335-340.
110. Lyu, J., S. H. Lee and H.-Y. Kim (2016). "Associations between healthy lifestyles and health outcomes among older Koreans." Geriatrics & gerontology international 16(6): 663-669.
111. Marceau, E. M., J. Lunn, J. Berry, P. J. Kelly and N. Solowij (2016). "The Montreal Cognitive Assessment (MoCA) is Sensitive to Head Injury and Cognitive Impairment in a Residential Alcohol and Other Drug Therapeutic Community." Journal of Substance Abuse Treatment 66: 30-36.
112. Maurage, F., P. de Timary, J. M. Tecco, S. Lechantre and D. Samson (2015). "Theory of mind difficulties in patients with alcohol dependence: beyond the prefrontal cortex dysfunction hypothesis." Alcoholism: Clinical & Experimental Research 39(6): 980-988.
113. McCallum, J., L. A. Simons, J. Simons and Y. Friedlander (2007). "Delaying dementia and nursing home placement: the Dubbo study of elderly Australians over a 14-year follow-up." Annals of the New York Academy of Sciences 1114: 121-129.
114. Mehlig, K., I. Skoog, X. Guo, M. Schutze, D. Gustafson, M. Waern, S. Ostling, C. Bjorkelund and L. Lissner (2008). "Alcoholic beverages and incidence of dementia: 34-year follow-up of the prospective population study of women in Goteborg." American Journal of Epidemiology 167(6): 684-691.
115. Miguez-Burbano, M. J., M. Nair, J. E. Lewis and J. Fishman (2009). "The role of alcohol on platelets, thymus and cognitive performance among HIV-infected subjects: are they related?" Platelets 20(4): 260-267.
116. Mlinarics, R., O. Kelemen, T. Sefcsik and D. Nemeth (2009). "[Cognitive impairment in patients with alcoholism after long-term abstinence]." Neuropsychopharmacologia Hungarica 11(3): 135-139.
117. Muller-Oehring, E. M., D. Kwon, B. J. Nagel, E. V. Sullivan, W. Chu, T. Rohlfing, D. Prouty, B. N. Nichols, J.-B. Poline, S. F. Tapert, S. A. Brown, K. Cummins, T. Brumback, I. M. Colrain, F. C. Baker, M. D. De Bellis, J. T. Voyvodic, D. B. Clark, A. Pfefferbaum and K. M. Pohl (2018). "Influences of Age, Sex, and Moderate Alcohol Drinking on the Intrinsic Functional Architecture of Adolescent Brains." Cerebral Cortex 28(3): 1049-1063.
118. Muller-Oehring, E. M., Y.-C. Jung, A. Pfefferbaum, E. V. Sullivan and T. Schulte (2015). "The Resting Brain of Alcoholics." Cerebral Cortex 25(11): 4155-4168.
119. Nemoto, Y., T. Saito, S. Kanamori, T. Tsuji, K. Shirai, H. Kikuchi, K. Maruo, T. Arao and K. Kondo (2017). "An additive effect of leading role in the organization between social participation and dementia onset among Japanese older adults: the AGES cohort study." BMC Geriatrics 17(1): 297.
120. Nguyen-Louie, T. T., A. N. Simmons, L. M. Squeglia, M. Alejandra Infante, J. P. Schacht and S. F. Tapert (2018). "Earlier alcohol use onset prospectively predicts changes in functional connectivity." Psychopharmacology 235(4): 1041-1054.
121. Nguyen-Louie, T. T., A. Tracas, L. M. Squeglia, G. E. Matt, S. Eberson-Shumate and S. F. Tapert (2016). "Learning and Memory in Adolescent Moderate, Binge, and Extreme-Binge Drinkers." Alcoholism: Clinical & Experimental Research 40(9): 1895-1904.
122. Nguyen-Louie, T. T., N. Castro, G. E. Matt, L. M. Squeglia, T. Brumback and S. F. Tapert (2015). "Effects of Emerging Alcohol and Marijuana Use Behaviors on Adolescents' Neuropsychological Functioning Over Four Years." Journal of Studies on Alcohol & Drugs 76(5): 738-748.

123. Niu, M.-J., F.-Z. Yin, L.-X. Liu, Y. Fang, X.-M. Xuan and G.-F. Wu (2013). "Non-high-density lipoprotein cholesterol and other risk factors of mild cognitive impairment among Chinese type 2 diabetic patients." Journal of Diabetes & its Complications 27(5): 443-446.
124. Nooyens, A. C. J., H. B. Bueno-de-Mesquita, B. M. van Gelder, M. P. J. van Boxtel and W. M. M. Verschuren (2014). "Consumption of alcoholic beverages and cognitive decline at middle age: the Doetinchem Cohort Study." British Journal of Nutrition 111(4): 715-723.
125. Nordstrom, P., A. Nordstrom, M. Eriksson, L. O. Wahlund and Y. Gustafson (2013). "Risk factors in late adolescence for young-onset dementia in men: A nationwide cohort study." JAMA Internal Medicine 173(17): 1612-1618.
126. Norton, M. C., J. Dew, H. Smith, E. Fauth, K. W. Piercy, J. C. S. Breitner, J. Tschanz, H. Wengreen, K. Welsh-Bohmer and I. Cache County (2012). "Lifestyle behavior pattern is associated with different levels of risk for incident dementia and Alzheimer's disease: the Cache County study." Journal of the American Geriatrics Society 60(3): 405-412.
127. Nowakowska, K., K. Jablkowska and A. Borkowska (2007). "[Cognitive dysfunctions in patients with alcohol dependence]." Psychiatria Polska 41(5): 693-702.
128. Ormstad, H., T. A. Rosness, A. L. M. Bergem, E. Bjertness and B. H. Strand (2016). "Alcohol consumption in the elderly and risk of dementia related death - A Norwegian prospective study with a 17-year follow-up." International Journal of Neuroscience 126(2): 135-144.
129. Paganini-Hill, A., C. H. Kawas and M. M. Corrada (2016). "Lifestyle Factors and Dementia in the Oldest-old: The 90+ Study." Alzheimer Disease & Associated Disorders 30(1): 21-26.
130. Park, B., J. Park, J. K. Jun, K. S. Choi and M. Suh (2013). "Gender differences in the association of smoking and drinking with the development of cognitive impairment." PLoS ONE [Electronic Resource] 8(10): e75095.
131. Park, K.-Y., H.-S. Hwang, Y.-P. Kim and H.-K. Park (2017). "Risk factors for cognitive decline associated with gait speed in community-dwelling elderly Koreans with MMSE scores of 30." Aging-Clinical & Experimental Research 29(2): 183-189.
132. Parrish, K. H., O. E. Atherton, A. Quintana, R. D. Conger and R. W. Robins (2016). "Reciprocal relations between internalizing symptoms and frequency of alcohol use: Findings from a longitudinal study of Mexican-origin youth." Psychology of Addictive Behaviors 30(2): 203-208.
133. Pearson, K. E., V. G. Wadley, L. A. McClure, J. M. Shikany, F. W. Unverzagt and S. E. Judd (2016). "Dietary patterns are associated with cognitive function in the REasons for Geographic And Racial Differences in Stroke (REGARDS) cohort." Journal of Nutritional Science 5: e38.
134. Pelletier, S., B. Nalpas, R. Alarcon, H. Rigole and P. Perney (2016). "Investigation of Cognitive Improvement in Alcohol-Dependent Inpatients Using the Montreal Cognitive Assessment (MoCA) Score." Journal of Addiction Print 2016: 1539096.
135. Peres, K., F. Matharan, M. Allard, H. Amieva, I. Baldi, P. Barberger-Gateau, V. Bergua, I. Bourdel-Marchasson, C. Delcourt, A. Foubert-Samier, A. Fourrier-Reglat, M. Gaimard, S. Laberon, C. Maubaret, V. Postal, C. Chantal, M. Rainfray, N. Rascle and J.-F. Dartigues (2012). "Health and aging in elderly farmers: the AMI cohort." BMC Public Health 12: 558.
136. Peters, R., N. Beckett, M. Geneva, M. Tzekova, F. H. Lu, R. Poulter, N. Gainsborough, B. Williams, M.-C. de Vernejoul, A. Fletcher and C. Bulpitt (2009). "Sociodemographic and lifestyle risk factors for incident dementia and cognitive decline in the HYVET." Age & Ageing 38(5): 521-527.
137. Pfefferbaum, A., T. Rohlfing, K. M. Pohl, B. Lane, W. Chu, D. Kwon, B. Nolan Nichols, S. A. Brown, S. F. Tapert, K. Cummins, W. K. Thompson, T. Brumback, M. J. Meloy, T. L. Jernigan, A. Dale, I. M. Colrain, F. C. Baker, D. Prouty, M. D. De Bellis, J. T. Voyvodic, D. B. Clark, B. Luna, T. Chung, B. J. Nagel and E. V. Sullivan (2016). "Adolescent Development of Cortical and White Matter Structure in the NCANDA Sample: Role of Sex, Ethnicity, Puberty, and Alcohol Drinking." Cerebral Cortex 26(10): 4101-4121.
138. Pitel, A. L., J. Rivier, H. Beaunieux, F. Vabret, B. Desgranges and F. Eustache (2009). "Changes in the episodic memory and executive functions of abstinent and relapsed alcoholics over a 6-month period." Alcoholism: Clinical & Experimental Research 33(3): 490-498.

139. Piumatti, G., S. Moore, D. Berridge, C. Sarkar and J. Gallacher (2018). "The relationship between alcohol use and long-term cognitive decline in middle and late life: a longitudinal analysis using UK Biobank." Journal of Public Health: 16.
140. Postuma, R. B., A. Iranzo, B. Hogl, I. Arnulf, L. Ferini-Strambi, R. Manni, T. Miyamoto, W. Oertel, Y. Dauvilliers, Y.-E. Ju, M. Puligheddu, K. Sonka, A. Pelletier, J. Santamaria, B. Frauscher, S. Leu-Semenescu, M. Zucconi, M. Terzaghi, M. Miyamoto, M. M. Unger, B. Carlander, M.-L. Fantini and J. Y. Montplaisir (2015). "Risk factors for neurodegeneration in idiopathic rapid eye movement sleep behavior disorder: a multicenter study." Annals of Neurology 77(5): 830-839.
141. Quaglino, V., E. De Wever and P. Maurage (2015). "Relations Between Cognitive Abilities, Drinking Characteristics, and Emotional Recognition in Alcohol Dependence: A Preliminary Exploration." Alcoholism: Clinical & Experimental Research 39(10): 2032-2038.
142. Reas, E. T., G. A. Laughlin, D. Kritz-Silverstein, E. Barrett-Connor and L. K. McEvoy (2016). "Moderate, Regular Alcohol Consumption is Associated with Higher Cognitive Function in Older Community-Dwelling Adults." Jpad 3(2): 105-113.
143. Reijs, B. L. R., S. J. B. Vos, H. Soininen, J. Lotjonen, J. Koikkalainen, M. Pikkarainen, A. Hall, R. Vanninen, Y. Liu, S.-K. Herukka, Y. Freund-Levi, G. B. Frisoni, L. Frolich, F. Nobili, M. O. Rikkert, L. Spiru, M. Tsolaki, A. K. Wallin, P. Scheltens, F. Verhey and P. J. Visser (2017). "Association Between Later Life Lifestyle Factors and Alzheimer's Disease Biomarkers in Non-Demented Individuals: A Longitudinal Descriptive Cohort Study." Journal of Alzheimer's Disease 60(4): 1387-1395.
144. Rist, P. M., J. R. Marden, B. D. Capistrant, Q. Wu and M. M. Glymour (2015). "Do physical activity, smoking, drinking, or depression modify transitions from cognitive impairment to functional disability?" Journal of Alzheimer's Disease 44(4): 1171-1180.
145. Ritchie, S. J., T. C. Bates, J. Corley, G. McNeill, G. Davies, D. C. Liewald, J. M. Starr and I. J. Deary (2014). "ALCOHOL consumption and lifetime change in cognitive ability: a gene x environment interaction study." Age 36(3): 9638.
146. Ritz, L., L. Coulbault, C. Lannuzel, C. Boudehent, S. Segobin, F. Eustache, F. Vabret, A. L. Pitel and H. Beaunieux (2016). "Clinical and Biological Risk Factors for Neuropsychological Impairment in Alcohol Use Disorder." PLoS ONE [Electronic Resource] 11(9): e0159616.
147. Roberts, R. O., Y. E. Geda, J. R. Cerhan, D. S. Knopman, R. H. Cha, T. J. H. Christianson, V. S. Pankratz, R. J. Ivnik, B. F. Boeve, H. M. O'Connor and R. C. Petersen (2010). "Vegetables, unsaturated fats, moderate alcohol intake, and mild cognitive impairment." Dementia & Geriatric Cognitive Disorders 29(5): 413-423.
148. Ros-Cucurull, E., R. F. Palma-Alvarez, C. Cardona-Rubira, E. Garcia-Raboso, C. Jacas, L. Grau-Lopez, A. C. Abad, L. Rodriguez-Cintas, S. Ros-Montalban, M. Casas, J. A. Ramos-Quiroga and C. Roncero (2018). "Alcohol use disorder and cognitive impairment in old age patients: A 6 months follow-up study in an outpatient unit in Barcelona." Psychiatry Research 261: 361-366.
149. Sabia, S., A. Singh-Manoux, G. Hagger-Johnson, E. Cambois, E. J. Brunner and M. Kivimaki (2012). "Influence of individual and combined healthy behaviours on successful aging." CMAJ Canadian Medical Association Journal 184(18): 1985-1992.
150. Samieri, C., O. I. Okereke, E. E Devore and F. Grodstein (2013). "Long-term adherence to the Mediterranean diet is associated with overall cognitive status, but not cognitive decline, in women." Journal of Nutrition 143(4): 493-499.
151. Sawyer, K. S., M. Oscar-Berman, S. Mosher Ruiz, D. A. Galvez, N. Makris, G. J. Harris and E. M. Valera (2016). "Associations Between Cerebellar Subregional Morphometry and Alcoholism History in Men and Women." Alcoholism: Clinical & Experimental Research 40(6): 1262-1272.
152. Schwarzinger, M., B. G. Pollock, O. S. M. Hasan, C. Dufouil, J. Rehm and G. QalyDays Study (2018). "Contribution of alcohol use disorders to the burden of dementia in France 2008-13: a nationwide retrospective cohort study." The lancet Public Health 3(3): e124-e132.
153. Schwarzinger, M., S. P. Thiebaut, S. Baillot, V. Mallet and J. Rehm (2017). "Alcohol use disorders and associated chronic disease - a national retrospective cohort study from France.[Erratum appears in BMC Public Health. 2017 Sep 22;17 (1):736; PMID: 28938882]." BMC Public Health 18(1): 43.

154. Silverberg, N. D., W. Panenka, G. L. Iverson, J. R. Brubacher, J. R. Shewchuk, M. K. S. Heran, G. C. S. Oh, W. G. Honer and R. T. Lange (2016). "Alcohol Consumption Does not Impede Recovery from Mild to Moderate Traumatic Brain Injury." Journal of the International Neuropsychological Society 22(8): 816-827.
155. Smith, K., L. Flicker, A. Dwyer, D. Atkinson, O. P. Almeida, N. T. Lautenschlager and D. LoGiudice (2010). "Factors associated with dementia in Aboriginal Australians." Australian & New Zealand Journal of Psychiatry 44(10): 888-893.
156. Son, S. J., K. S. Lee, B. H. Oh and C. H. Hong (2012). "The effects of head circumference (HC) and lifetime ALCOHOL consumption (AC) on cognitive function in the elderly." Archives of Gerontology & Geriatrics 54(2): 343-347.
157. Squeglia, L. M., A. D. Spadoni, M. A. Infante, M. G. Myers and S. F. Tapert (2009). "Initiating moderate to heavy alcohol use predicts changes in neuropsychological functioning for adolescent girls and boys.[Erratum appears in Psychol Addict Behav. 2010 Mar;24(1):118]." Psychology of Addictive Behaviors 23(4): 715-722.
158. Srinivasa, R. N., H. C. Rossetti, M. K. Gupta, R. N. Rosenberg, M. F. Weiner, R. M. Peshock, R. W. McColl, L. S. Hynan, R. T. Lucarelli and K. S. King (2016). "Cardiovascular Risk Factors Associated with Smaller Brain Volumes in Regions Identified as Early Predictors of Cognitive Decline." Radiology 278(1): 198-204.
159. Steinberg, S. I., M. D. Sammel, B. T. Harel, A. Schembri, C. Policastro, H. R. Bogner, S. Negash and S. E. Arnold (2015). "Exercise, sedentary pastimes, and cognitive performance in healthy older adults." American Journal of Alzheimer's Disease and Other Dementias 30(3): 290-298.
160. Stephan, B. C. M., C. Tzourio, S. Auriacombe, H. Amieva, C. Dufouil, A. Alperovitch and T. Kurth (2015). "Usefulness of data from magnetic resonance imaging to improve prediction of dementia: population based cohort study." BMJ 350: h2863.
161. Stephens, C., J. Spicer, C. Budge, B. Stevenson and F. Alpass (2015). "Accounting for differences in cognitive health between older adults in New Zealand and the USA." International Psychogeriatrics 27(4): 591-600.
162. Su, P., C.-C. Hsu, H.-C. Lin, W.-S. Huang, T.-L. Yang, W.-T. Hsu, C.-L. Lin, C.-Y. Hsu, K.-H. Chang and Y.-C. Hsu (2017). "Age-related hearing loss and dementia: a 10-year national population-based study." European Archives of Oto-Rhino-Laryngology 274(5): 2327-2334.
163. Subramaniam, M., E. Abdin, J. A. Vaingankar and S. A. Chong (2013). "Gender differences in disability in a multiethnic Asian population: the Singapore Mental Health Study." Comprehensive Psychiatry 54(4): 381-387.
164. Sullivan, E. V., T. Brumback, S. F. Tapert, R. Fama, D. Prouty, S. A. Brown, K. Cummins, W. K. Thompson, I. M. Colrain, F. C. Baker, M. D. De Bellis, S. R. Hooper, D. B. Clark, T. Chung, B. J. Nagel, B. N. Nichols, T. Rohlfing, W. Chu, K. M. Pohl and A. Pfefferbaum (2016). "Cognitive, emotion control, and motor performance of adolescents in the NCANDA study: Contributions from alcohol consumption, age, sex, ethnicity, and family history of addiction." Neuropsychology 30(4): 449-473.
165. Sun, Q., M. K. Townsend, O. I. Okereke, E. B. Rimm, F. B. Hu, M. J. Stampfer and F. Grodstein (2011). "Alcohol consumption at midlife and successful ageing in women: a prospective cohort analysis in the nurses' health study." PLoS Medicine / Public Library of Science 8(9): e1001090.
166. Takahashi, P. Y., C. R. Caldwell and P. V. Targonski (2011). "Effect of alcohol and tobacco use on vascular dementia: a matched case control study." Vascular Health & Risk Management 7: 685-691.
167. Tang, H. D., Y. H. Yao, R. F. Xu, S. D. Chen and Q. Cheng (2008). "Analysis of cognitive impairment and associated factors of the elderly in Shanghai suburbs." Chinese Journal of Contemporary Neurology and Neurosurgery 8(4): 318-322.
168. Theadom, A., V. Parag, T. Dowell, K. McPherson, N. Starkey, S. Barker-Collo, K. Jones, S. Ameratunga, V. L. Feigin and B. R. Group (2016). "Persistent problems 1 year after mild traumatic brain injury: a longitudinal population study in New Zealand." British Journal of General Practice 66(642): e16-23.
169. Toda, A., Y. Tagata, T. Nakada, M. Komatsu, N. Shibata and H. Arai (2013). "Changes in Mini-Mental State Examination score in Alzheimer's disease patients after stopping habitual drinking." Psychogeriatrics:The Official Journal of the Japanese Psychogeriatric Society 13(2): 94-98.

170. Townsend, M. K., E. Devore, J. H. Kang and F. Grodstein (2009). "The relation between moderate alcohol consumption and cognitive function in older women with type 2 diabetes." Diabetes Research & Clinical Practice 85(3): 322-327.
171. Tremolizzo, L., E. Bianchi, E. Susani, E. Pupillo, P. Messina, A. Aliprandi, A. Salmaggi, M. Cosseddu, A. Pilotto, B. Borroni, A. Padovani, C. Bonomini, O. Zanetti, I. Appollonio, E. Beghi and C. Ferrarese (2017). "Voluptuary Habits and Risk of Frontotemporal Dementia: A Case Control Retrospective Study." Journal of Alzheimer's Disease 60(2): 335-340.
172. Unverzagt, F. W., L. T. Guey, R. N. Jones, M. Marsiske, J. W. King, V. G. Wadley, M. Crowe, G. W. Rebok and S. L. Tennstedt (2012). "ACTIVE cognitive training and rates of incident dementia." Journal of the International Neuropsychological Society 18(4): 669-677.
173. Vachon, D. D., R. F. Krueger, D. E. Irons, W. G. Iacono and M. McGue (2017). "Are Alcohol Trajectories a Useful Way of Identifying At-Risk Youth? A Multiwave Longitudinal-Epidemiologic Study." Journal of the American Academy of Child & Adolescent Psychiatry 56(6): 498-505.
174. Vaillant, G. E., O. I. Okereke, K. Mukamal and R. J. Waldinger (2014). "Antecedents of intact cognition and dementia at age 90 years: a prospective study." International Journal of Geriatric Psychiatry 29(12): 1278-1285.
175. Valls-Serrano, C., A. Verdejo-Garcia and A. Caracuel (2016). "Planning deficits in polysubstance dependent users: Differential associations with severity of drug use and intelligence." Drug & Alcohol Dependence 162: 72-78.
176. van der Heide, I., U. Gehring, G. H. Koppelman and A. H. Wijga (2016). "Health-Related Factors Associated with Discrepancies between Children's Potential and Attained Secondary School Level: A Longitudinal Study." PLoS ONE [Electronic Resource] 11(12): e0168110.
177. Velasquez-Perez, L., J. Guerrero-Camacho, Y. Rodriguez-Agudelo, M. E. Alonso-Vilatela and P. Yescas-Gomez (2008). "Conversion from slight cognitive deterioration to dementia." Revista Ecuatoriana de Neurologia 17(1-3).
178. Vincze, G., P. Almos, K. Boda, P. Dome, N. Bodi, G. Szlavik, E. Magloczki, M. Pakaski, Z. Janka and J. Kalman (2007). "Risk factors of cognitive decline in residential care in Hungary." International Journal of Geriatric Psychiatry 22(12): 1208-1216.
179. Virag, M., K. Janacsek, A. Horvath, Z. Bujdoso, D. Fabo and D. Nemeth (2015). "Competition between frontal lobe functions and implicit sequence learning: evidence from the long-term effects of alcohol." Experimental Brain Research 233(7): 2081-2089.
180. Virta, J. J., T. Jarvenpaa, K. Heikkila, M. Perola, M. Koskenvuo, I. Raiha, J. O. Rinne and J. Kaprio (2010). "Midlife alcohol consumption and later risk of cognitive impairment: A twin followup study." Journal of Alzheimer's Disease 22(3): 939-948.
181. Voortman, T., J. C. Kiefte-de Jong, M. A. Ikram, B. H. Stricker, F. J. A. van Rooij, L. Lahousse, H. Tiemeier, G. G. Brusselle, O. H. Franco and J. D. Schoufour (2017). "Adherence to the 2015 Dutch dietary guidelines and risk of non-communicable diseases and mortality in the Rotterdam Study." European Journal of Epidemiology 32(11): 993-1005.
182. Vos, S. J. B., M. P. J. van Boxtel, O. J. G. Schiepers, K. Deckers, M. de Vugt, I. Carriere, J.-F. Dartigues, K. Peres, S. Artero, K. Ritchie, L. Galluzzo, E. Scafato, G. B. Frisoni, M. Huisman, H. C. Comijs, S. F. Sacuiu, I. Skoog, K. Irving, C. A. O'Donnell, F. R. J. Verhey, P. J. Visser and S. Kohler (2017). "Modifiable Risk Factors for Prevention of Dementia in Midlife, Late Life and the Oldest-Old: Validation of the LIBRA Index." Journal of Alzheimer's Disease 58(2): 537-547.
183. Wadley, V. G., L. A. McClure, V. J. Howard, F. W. Unverzagt, R. C. Go, C. S. Moy, M. R. Crowther, C. R. Gomez and G. Howard (2007). "Cognitive status, stroke symptom reports, and modifiable risk factors among individuals with no diagnosis of stroke or transient ischemic attack in the REasons for Geographic and Racial Differences in Stroke (REGARDS) Study." Stroke 38(4): 1143-1147.
184. Wang, R., L. Fratiglioni, E. J. Laukka, M. Lovden, G. Kalpouzos, L. Keller, C. Graff, A. Salami, L. Backman and C. Qiu (2015). "Effects of vascular risk factors and APOE epsilon4 on white matter integrity and cognitive decline." Neurology 84(11): 1128-1135.

185. Wang, T., S. Xiao, K. Chen, C. Yang, S. Dong, Y. Cheng, X. Li, J. Wang, M. Zhu, F. Yang, G. Li, N. Su, Y. Liu, J. Dai and M. Zhang (2017). "Prevalence, Incidence, Risk and Protective Factors of Amnestic Mild Cognitive Impairment in the Elderly in Shanghai." Current Alzheimer Research 14(4): 460-466.
186. Weber, E., E. E. Morgan, J. E. Iudicello, K. Blackstone, I. Grant, R. J. Ellis, S. L. Letendre, S. Little, S. Morris, D. M. Smith, D. J. Moore, S. P. Woods and T. Group (2013). "Substance use is a risk factor for neurocognitive deficits and neuropsychiatric distress in acute and early HIV infection." Journal of Neurovirology 19(1): 65-74.
187. Weyerer, S., M. Schaufele, B. Wiese, W. Maier, F. Tebarth, H. van den Bussche, M. Pentzek, H. Bickel, M. Luppa, S. G. Riedel-Heller and g. German AgeCoDe Study (2011). "Current alcohol consumption and its relationship to incident dementia: results from a 3-year follow-up study among primary care attenders aged 75 years and older." Age & Ageing 40(4): 456-463.
188. Xu, G., X. Liu, Q. Yin, W. Zhu, R. Zhang and X. Fan (2009). "Alcohol consumption and transition of mild cognitive impairment to dementia." Psychiatry & Clinical Neurosciences 63(1): 43-49.
189. Xue, H., Q. Sun, L. Liu, L. Zhou, R. Liang, R. He and H. Yu (2017). "Risk factors of transition from mild cognitive impairment to Alzheimer's disease and death: A cohort study." Comprehensive Psychiatry 78: 91-97.
190. Yamamoto, N., G. Yamanaka, E. Takasugi, M. Ishikawa, T. Yamanaka, S. Murakami, T. Hanafusa, K. Matsubayashi and K. Otsuka (2009). "Lifestyle intervention reversed cognitive function in aged people with diabetes mellitus: two-year follow up." Diabetes Research & Clinical Practice 85(3): 343-346.
191. Yamawaki, M., K. Wada-Isoe, M. Yamamoto, S. Nakashita, Y. Uemura, Y. Takahashi, T. Nakayama and K. Nakashima (2015). "Association of cerebral white matter lesions with cognitive function and mood in Japanese elderly people: a population-based study." Brain and Behavior 5(3): e00315.
192. Yen, C.-H., C.-J. Yeh, C.-C. Wang, W.-C. Liao, S.-C. Chen, C.-C. Chen, J. Liang, T.-J. Lai, H.-S. Lin, S.-H. Lee and M.-C. Lee (2010). "Determinants of cognitive impairment over time among the elderly in Taiwan: results of the national longitudinal study." Archives of Gerontology & Geriatrics 50 Suppl 1: S53-57.
193. Yen, C.-H., Y.-W. Yeh, C.-S. Liang, P.-S. Ho, S.-C. Kuo, C.-C. Huang, C.-Y. Chen, M.-C. Shih, K.-H. Ma, G.-S. Peng, R.-B. Lu and S.-Y. Huang (2015). "Reduced Dopamine Transporter Availability and Neurocognitive Deficits in Male Patients with Alcohol Dependence." PLoS ONE [Electronic Resource] 10(6): e0131017.
194. Zanjani, F., B. G. Downer, T. M. Kruger, S. L. Willis and K. W. Schaie (2013). "ALCOHOL effects on cognitive change in middle-aged and older adults." Aging & Mental Health 17(1): 12-23.
195. Zhou, S., R. Zhou, T. Zhong, R. Li, J. Tan and H. Zhou (2014). "Association of smoking and ALCOHOL drinking with dementia risk among elderly men in China." Current Alzheimer Research 11(9): 899-907.

Appendix 11. Abbreviations

95% CI	95% confidence interval
ACE-R	Addenbrooke's Cognitive Examination - Revised
AWC	Alcohol Working Committee
CI	Cognitive impairment
COWAT	Controlled Oral Word Association Test
CVD	Cardiovascular disease
DSCT	Digit symbol coding test
DSST	Digit symbol substitution test
g	grams
GCF	Global cognitive function
GFQ	Graduated frequency questionnaire
GRADE	Grading of Recommendations Assessment, Development and Evaluation
HR	Hazard ratio
HVLT-R	Hopkins verbal learning test
MCI	Mild cognitive impairment
MD	Mean difference (usually based on a scale score or test)
MMSE	Mini Mental State Examination
MOCA	Montreal Cognitive Assessment
ms	milliseconds
NHMRC	National Health and Medical Research Council
NIAAA	National Institute on Alcohol Abuse and Alcoholism (United States)
NIA-AA	National Institute on Aging and the Alzheimer's Association (United States)
ONHMRC	Office of the National Health and Medical Research Council
OR	Odds ratio
PECO	Population, Exposure, Comparator, Outcome
PRISMA	Preferred Reporting Items for Systematic Reviews and Meta-Analyses
PRISMA-P	Preferred Reporting Items for Systematic Reviews and Meta-Analyses - protocols
RoB	Risk of bias
ROBINS-I	Risk Of Bias In Non-randomized Studies of Interventions
SCD	Specific cognitive domain
SD	Standard deviation
SE	Standard error
SMD	Standardised mean difference
SR	systematic review
STROBE	Strengthening the Reporting of Observational Studies in Epidemiology
T0, T1, etc.	Time 0: 1st measurement point; time 1: 2nd measurement point
TICS	Telephone Interview for Cognitive Status
TMT-A & TMT-B	Trail making test part A; Trail making test part B

References

1. Higgins J, Green S (Eds.): **Cochrane Handbook for Systematic Reviews of Interventions Version 5.1.0 [updated March 2011].** The Cochrane Collaboration; 2011.
2. Schunemann HJ, Brozek J, Guyatt G, Oxman AD (Eds.): **Handbook for grading the quality of evidence and the strength of recommendations using the GRADE approach. Accessed 5 July 2016.** Hamilton, Canada: McMaster University; 2013.
3. Liberati A, Altman DG, Tetzlaff J, Mulrow C, Gotzsche PC, Ioannidis JPA, Clarke M, Devereaux PJ, Kleijnen J, Moher D: **The PRISMA statement for reporting systematic reviews and meta-analyses of studies that evaluate healthcare interventions: explanation and elaboration.** *BMJ* 2009, **339:**b2700-.
4. Moher D, Liberati A, Tetzlaff J, Altman DG, for the PRISMA Group: **Preferred reporting items for systematic reviews and meta-analyses: the PRISMA statement.** *BMJ* 2009, **339:**b2535-.
5. Moher D, Shamseer L, Clarke M, Ghersi D, Liberati A, Petticrew M, Shekelle P, Stewart LA, Group P-P: **Preferred reporting items for systematic review and meta-analysis protocols (PRISMA-P) 2015 statement.** *Syst Rev* 2015, **4:**1.
6. Shamseer L, Moher D, Clarke M, Ghersi D, Liberati A, Petticrew M, Shekelle P, Stewart LA, Group P-P: **Preferred reporting items for systematic review and meta-analysis protocols (PRISMA-P) 2015: elaboration and explanation.** *BMJ* 2015, **349:**g7647.
7. Naimi TS, Stockwell T, Zhao J, Xuan Z, Dangardt F, Saitz R, Liang W, Chikritzhs T: **Selection biases in observational studies affect associations between 'moderate' alcohol consumption and mortality.** *Addiction* 2017, **112:**207-214.
8. Knott CS, Coombs N, Stamatakis E, Biddulph JP: **All cause mortality and the case for age specific alcohol consumption guidelines: pooled analyses of up to 10 population based cohorts.** *BMJ* 2015, **350:**h384.
9. Topiwala A, Allan CL, Valkanova V, Zsoldos E, Filippini N, Sexton C, Mahmood A, Fooks P, Singh-Manoux A, Mackay CE, et al: **Moderate alcohol consumption as risk factor for adverse brain outcomes and cognitive decline: Longitudinal cohort study.** *BMJ (Online)* 2017, **357**.
10. Australian Institute of Health and Welfare: **National Drug Strategy Household Survey 2016: detailed findings. Drug Statistics series no. 31. Cat. no. PHE 214.** Canberra: AIHW; 2017.
11. Livingston G, Sommerlad A, Orgeta V, Costafreda SG, Huntley J, Ames D, Ballard C, Banerjee S, Burns A, Cohen-Mansfield J, et al: **Dementia prevention, intervention, and care.** *Lancet* 2017, **390:**2673-2734.
12. Black DW: **DSM-5 guidebook : Chapter 17 Neurocognitive Disorders.** In *DSM-5 guidebook : the essential companion to the Diagnostic and statistical manual of mental disorders, fifth edition.* First edition. edition. Edited by Grant JE. Arlington, VA: American Psychiatric Publishing; 2014
13. Winblad B, Palmer K, Kivipelto M, Jelic V, Fratiglioni L, Wahlund LO, Nordberg A, Backman L, Albert M, Almkvist O, et al: **Mild cognitive impairment--beyond controversies, towards a consensus: report of the International Working Group on Mild Cognitive Impairment.** *J Intern Med* 2004, **256:**240-246.
14. Albert MS, DeKosky ST, Dickson D, Dubois B, Feldman HH, Fox NC, Gamst A, Holtzman DM, Jagust WJ, Petersen RC, et al: **The diagnosis of mild cognitive impairment due to Alzheimer's disease: recommendations from the National Institute on Aging-Alzheimer's Association workgroups on diagnostic guidelines for Alzheimer's disease.** *Alzheimers Dement* 2011, **7:**270-279.
15. Matthews FE, Stephan BC, McKeith IG, Bond J, Brayne C, Medical Research Council Cognitive Function and Ageing Study: **Two-year progression from mild cognitive impairment to dementia: to what extent do different definitions agree?** *J Am Geriatr Soc* 2008, **56:**1424-1433.
16. Davis DH, Creavin ST, Noel-Storr A, Quinn TJ, Smailagic N, Hyde C, Brayne C, McShane R, Cullum S: **Neuropsychological tests for the diagnosis of Alzheimer's disease dementia and other dementias: a generic protocol for cross-sectional and delayed-verification studies.** *Cochrane Database Syst Rev* 2013.
17. Anstey KJ, Mack HA, Cherbuin N: **Alcohol consumption as a risk factor for dementia and cognitive decline: meta-analysis of prospective studies.** *Am J Geriatr Psychiatry* 2009, **17:**542-555.
18. Harrison JK, Noel-Storr AH, Demeyere N, Reynish EL, Quinn TJ: **Outcomes measures in a decade of dementia and mild cognitive impairment trials.** *Alzheimers Res Ther* 2016, **8:**48.
19. Reeves B, Deeks J, Higgins J, Wells G: **Chapter 13: Including non-randomized studies. .** In *Cochrane Handbook for Systematic Reviews of Interventions Version 510 [updated March 2011] Available from wwwcochrane-handbookorg.* Edited by Higgins J, Green S: The Cochrane Collaboration; 2011

20. Hartling L, Featherstone R, Nuspl M, Shave K, Dryden DM, Vandermeer B: **Grey literature in systematic reviews: a cross-sectional study of the contribution of non-English reports, unpublished studies and dissertations to the results of meta-analyses in child-relevant reviews.** *BMC Medical Research Methodology* 2017, **17:**64.
21. Morrison A, Polisena J, Husereau D, Moulton K, Clark M, Fiander M, Mierzwinski-Urban M, Clifford T, Hutton B, Rabb D: **The effect of English-language restriction on systematic review-based meta-analyses: a systematic review of empirical studies.** *Int J Technol Assess Health Care* 2012, **28:**138-144.
22. NHMRC Clinical Trials Centre: **Evaluating the evidence on the health effects of alcohol consumption: evidence evaluation report commission by the Office of the National Health and Medical Research Council** Sydney: The University of Sydney; 2017.
23. Xu W, Wang H, Wan Y, Tan C, Li J, Tan L, Yu J-T: **Alcohol consumption and dementia risk: a dose-response meta-analysis of prospective studies.** *European journal of epidemiology* 2017, **32:**31-42.
24. **Risk Of Bias In Non-randomized Studies of Interventions (ROBINS-I): detailed guidance, updated 12 October 2016. Available from http://www.riskofbias.info Accessed 25 March 2018**
25. Sterne JA, Hernan MA, Reeves BC, Savovic J, Berkman ND, Viswanathan M, Henry D, Altman DG, Ansari MT, Boutron I, et al: **ROBINS-I: a tool for assessing risk of bias in non-randomised studies of interventions.** *BMJ* 2016, **355:**i4919.
26. Schunemann HJ, Cuello C, Akl EA, Mustafa RA, Meerpohl JJ, Thayer K, Morgan RL, Gartlehner G, Kunz R, Katikireddi SV, et al: **GRADE Guidelines: 18. How ROBINS-I and other tools to assess risk of bias in non-randomized studies should be used to rate the certainty of a body of evidence.** *J Clin Epidemiol* 2018.
27. Chinn S: **A simple method for converting an odds ratio to effect size for use in meta-analysis.** *Stat Med* 2000, **19:**3127-3131.
28. Karahalios A, English DR, Simpson JA: **Change in body size and mortality: a systematic review and meta-analysis.** *Int J Epidemiol* 2017, **46:**526-546.
29. Crippa A, Orsini N: **Dose-response meta-analysis of differences in means.** *BMC Med Res Methodol* 2016, **16:**91.
30. Stockwell T, Chikritzhs T: **International Guide for Monitoring Alcohol Consumption and Related Harm.** Geneva, Switzerland: Department of Mental Health and Substance Dependence, Noncommunicable Diseases and Mental Health Cluster, World Health Organization; 2000.
31. Il'yasova D, Hertz-Picciotto I, Peters U, Berlin JA, Poole C: **Choice of exposure scores for categorical regression in meta-analysis: a case study of a common problem.** *Cancer Causes Control* 2005, **16:**383-388.
32. Crippa A, Orsini N: **Multivariate Dose-Response Meta-Analysis: The dosresmeta R Package.** *2016* 2016, **72:**15.
33. Hernan MA: **The C-Word: Scientific Euphemisms Do Not Improve Causal Inference From Observational Data.** *Am J Public Health* 2018, **108:**616-619.
34. Mongan D, Long J: **Standard drink measures throughout Europe; peoples' understanding of standard drinks and their use in drinking guidelines, alcohol surveys and labelling.** Dublin: RARHA (Reducing Alcohol Related Harma); 2015.
35. Lazarus C, Haneef R, Ravaud P, Boutron I: **Classification and prevalence of spin in abstracts of non-randomized studies evaluating an intervention.** *BMC Med Res Methodol* 2015, **15:**85.
